# The Spermatophore in *Glossina morsitans morsitans*: Insights into Male Contributions to Reproduction

**DOI:** 10.1038/srep20334

**Published:** 2016-02-05

**Authors:** Francesca Scolari, Joshua B. Benoit, Veronika Michalkova, Emre Aksoy, Peter Takac, Adly M. M. Abd-Alla, Anna R. Malacrida, Serap Aksoy, Geoffrey M. Attardo

**Affiliations:** 1 University of Pavia, Dept of Biology and Biotechnology, 27100 Pavia, Italy; 2 University of Cincinnati, McMicken School of Arts and Sciences, Dept of Biological Sciences, 45221, Cincinnati, OH, USA; 3 Yale School of Public Health, Dept of Epidemiology of Microbial Diseases, 06520, New Haven, CT, USA; 4 Section of Molecular and Applied Zoology, Institute of Zoology, Slovak Academy of Sciences, 845 06 SR, Bratislava, Slovakia; 5 International Atomic Energy Agency, Joint FAO/IAEA Division of Nuclear Techniques in Food and Agriculture, IPC Laboratory, A-1400, Vienna, Austria

## Abstract

Male Seminal Fluid Proteins (SFPs) transferred during copulation modulate female reproductive physiology and behavior, impacting sperm storage/use, ovulation, oviposition, and remating receptivity. These capabilities make them ideal targets for developing novel methods of insect disease vector control. Little is known about the nature of SFPs in the viviparous tsetse flies (Diptera: Glossinidae), vectors of Human and Animal African trypanosomiasis. In tsetse, male ejaculate is assembled into a capsule-like spermatophore structure visible post-copulation in the female uterus. We applied high-throughput approaches to uncover the composition of the spermatophore in *Glossina morsitans morsitans*. We found that both male accessory glands and testes contribute to its formation. The male accessory glands produce a small number of abundant novel proteins with yet unknown functions, in addition to enzyme inhibitors and peptidase regulators. The testes contribute sperm in addition to a diverse array of less abundant proteins associated with binding, oxidoreductase/transferase activities, cytoskeletal and lipid/carbohydrate transporter functions. Proteins encoded by female-biased genes are also found in the spermatophore. About half of the proteins display sequence conservation relative to other Diptera, and low similarity to SFPs from other studied species, possibly reflecting both their fast evolutionary pace and the divergent nature of tsetse’s viviparous biology.

Vector-borne diseases account for more than 17% of all infectious diseases, causing more than 1 million deaths each year^1^. Among these vectors are insects, including mosquitoes, reduviid bugs, sand flies, black flies and tsetse flies that transmit devastating diseases to humans, such as malaria, Dengue fever, Chagas disease, Leishmaniases, Onchocerciasis and African trypanosomiasis. Effective control tools, such as vaccines to prevent disease emergence, or effective and affordable drugs for treatment of infected mammalian hosts, are lacking. In contrast, traditional vector control methods, such as adult spraying, larvicides, traps and targets, which decrease vector populations, are effective in reducing disease transmission^2^. Many of these methods rely on insecticides, which are expensive, toxic and drive the evolution of resistance in target insects. Thus, novel vector control strategies are needed.

Tsetse (Diptera: Glossinidae) transmitted Human African Trypanosomiasis (HAT, or sleeping sickness) is one of the most neglected tropical diseases in Africa. HAT is caused by the flagellate protozoa *Trypanosoma brucei rhodesiense* in East and Southern Africa, and by *T. b. gambiense* in West and Central Africa^3^. In addition to HAT, nagana (or Animal African Trypanosomiasis, AAT), caused by *T. b. brucei* and the related trypanosomatids *T. congolense* and *T. vivax*, impacts land use, and results in agricultural losses equivalent to US$ 4.75 billion per year^4^. In the absence of vaccines and efficacious therapeutic agents, control of sleeping sickness and nagana primarily involves reducing tsetse populations using traps and targets^5^. The success of these methods is dependent on tsetse’s already low reproductive capacity due to its unusual viviparous reproductive physiology.

Tsetse females produce a single offspring at each reproductive cycle through adenotrophic viviparity (development and feeding of intrauterine offspring). This biology requires high levels of maternal investment^6^. To accommodate viviparity, tsetse females have undergone extreme reproductive adaptations, such as ovarian follicle reduction, uterine expansion and development of accessory glands (milk glands) to accommodate and feed a developing larva that increases over 100-fold in dry mass over a 6-day period^6^.

Less is known about the tsetse male reproductive biology, which also displays unique features. Insects have developed multiple strategies to ensure successful sperm transfer and long-term evolutionary success of mating outcomes. Males from many Dipteran species transfer free sperm in seminal fluid (SF) by means of sperm pumps, using an elongate penis (or aedeagus) specialized for direct transfer to the openings of the spermathecal duct^7^. In several other species, the SF components form a ‘mating plug’ within the female reproductive tract during or shortly after mating^8^,^9^. These plugs can affect sperm storage and release^10^. While the male tsetse reproductive tract is morphologically similar to other Cyclorrhaphous Diptera, their ejaculate is transferred in the form of a spermatophore, a rare feature among Diptera. Tsetse’s spermatophore is a capsule-like structure that is thought to be assembled within the uterus of the female^11^ from male accessory gland (MAG) products^12^. However, the role of secretions from other male reproductive tissues (such as testes) and/or female body compartments in its formation remains unknown. In addition, knowledge on the molecular composition of the spermatophore and the function of its individual components on fertilization and female post-mating response is almost completely lacking. Male seminal fluid proteins (SFPs) are documented as playing multiple post-copulatory roles in female insects, including facilitating sperm storage and provision, initiating changes to female physiology/behavior and interference with ejaculate from competing males^13^. Identifying these functions in tsetse is fundamental to achieving a better understanding of their reproduction, which in turn can lead to the development/improvement of new methods for tsetse population control.

We have addressed these questions by investigating the temporal events leading up to the formation of the tsetse spermatophore, and identifying the composition and origin of its protein components, using proteomic and transcriptomic approaches, respectively. We describe the extent of the paternal and maternal contributions to the spermatophore proteins, as well as the respective contributions made by the MAGs and testes. Beyond the conserved functions already associated with insect SFPs and sperm in characterized systems, we discovered a number of abundant novel proteins that form the foundation to advance our understanding of male reproductive physiology in this viviparous vector species.

## Results

### Timing of Spermatophore Assembly in the Female Uterus

**Figure 1:**
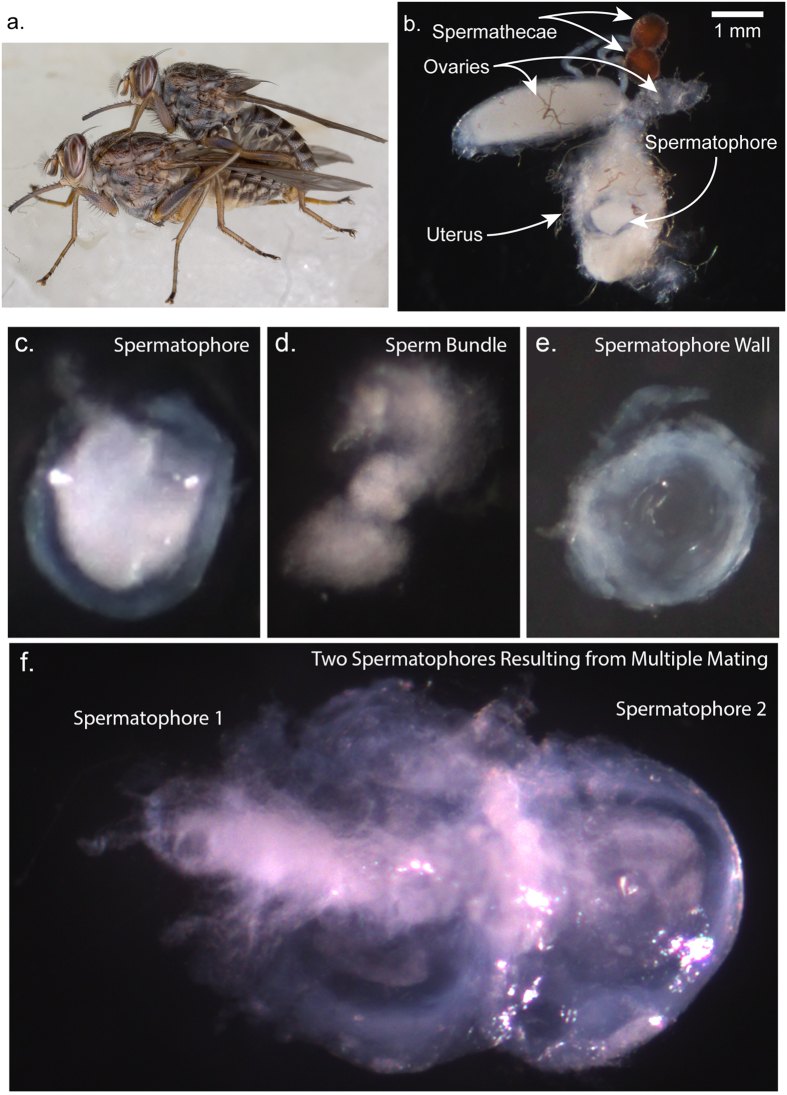
Tsetse mating and spermatophore production. (**a**) Mating pair. (**b**) Female reproductive tract dissected immediately after mating: a spermatophore is evident in the uterus. Scale bar = 1 mm. (**c**) Spermatophore filled with sperm isolated from the uterus of a mated female. (**d**) Sperm bundle isolated from the spermatophore capsule. (**e**) Spermatophore wall. (**f**) Two spermatophores resulting from double mating.

Individual pairs of virgin males and females were allowed to mate under controlled conditions to obtain information on the timing of spermatophore assembly in the female uterus. In agreement with previous studies^14^, the individual mating pairs remained *in copula* for an average duration of 122 min (±25.18) (Fig. 1a). After the completion of mating, a fully formed spermatophore was evident in the female uterus (Fig. 1b). To determine the time at which the spermatophore was formed during copulation, mating couples were interrupted at specified time points after the initiation of mating (5, 30, 60, 75 and 100 min, respectively) and the reproductive tracts of the females were dissected. No free sperm were detected in the uterus prior to the presence of the whole spermatophore, which was observed only at around 100 min, close to the end of copulation (Supplementary Fig. S1). This suggests that the seminal components, i.e. spermatozoa and seminal fluid (SF), are assembled into the spermatophore in a short time window before the end of coitus. The spermatophore was found adhering to the antero-dorsal wall of the uterus. It appeared as a double-layered capsule containing the sperm bundle and the SF (Fig. 1c–e). Its opening oriented towards the spermathecal ducts allows a direct sperm flow to the two spermathecae. Indeed the presence of sperm in spermathecae was detected close to the end of coitus. After copulation, female remating receptivity declines rapidly, and it is almost absent at ~48 hours^15^. However, this time window can provide the opportunity for other males to inseminate the same female as females presented with new partners immediately after the first mating remain receptive. In females that mated immediately after the first *copula*, we observed two fully formed spermatophores (Fig. 1f) that had both their openings oriented towards the spermathecal ducts. It is possible that the spermatophore resulting from the first mating acts as a physical barrier for the sperm enclosed in the second one, possibly impeding proper sperm movement to the spermathecae.

### Protein Composition of the Spermatophore

**Figure 2:**
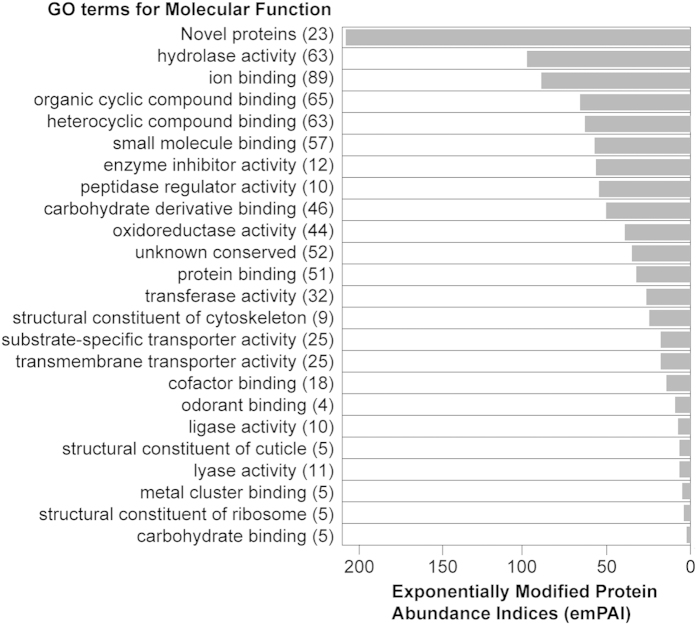
Distribution of the 287 *G. m. morsitans* spermatophore proteins in functional classes. The number of total detected proteins associated with corresponding Gene Ontology term (Molecular Function Level III) is shown in brackets. Of the total spermatophore proteins, 18% (n = 52) yielded significant hits to proteins identified in other insect species but with still uncharacterized function, and were thus classified as ‘unknown conserved’. Among the novel protein class, in addition to 20 newly identified sequences, are three that had previously been identified and are currently limited to *Glossina*: *Gmfb8* (GMOY005771 and GMOY009539) and *immune responsive product Fb49* (GMOY000899). For each class, protein abundances were assessed using emPAI calculation.

To understand the composition of the tsetse spermatophore, we dissected the uterus of mated females immediately post copulation, and analyzed the protein components of the spermatophores using mass spectrometric (MS) proteomic analyses (Supplementary Data S1). The resulting data were compared against the *Glossina m. morsitans* predicted proteome database (n = 12,448 predicted proteins, version GmorY1.4^16^) and a total of 287 spermatophore proteins were identified (Supplementary Data S2). Of these proteins, 92% (n = 264) produced best hits against the NCBI nr database with an expectation value (e) of at least <10^−10^ using BLASTp, while the remaining 8% (n = 23) shared no similarity to sequences present in the GenBank database and were grouped as novel proteins. The 264 spermatophore proteins that resulted in significant hits to the nr database were assigned to different Gene Ontology (GO) functional classes (Supplementary Data S1). We then correlated each GO class (Molecular Function, Level III) with the sum of the emPAI (Exponentially Modified Protein Abundance Index) values of its respective proteins as derived from the MS analysis of the spermatophore. In the absence of any similarity to known sequences, we considered the novel proteins as a separate class, which is also the most abundant in the spermatophore proteome, followed by sequences with putative hydrolase and ion binding activities, proteins with binding functions, enzyme inhibitors, and peptidase regulators, respectively (Fig. 2). Interestingly, six proteins account for 50% of the spermatophore proteome (Supplementary Data S1), and they include the novel proteins GMOY005875 (the most abundant: 27% relative emPAI), GMOY005874, GMOY004506, GMOY004319, and GMOY005876, and Serpin 1.

### Gender-Bias in Spermatophore Component Gene Expression

**Figure 3:**
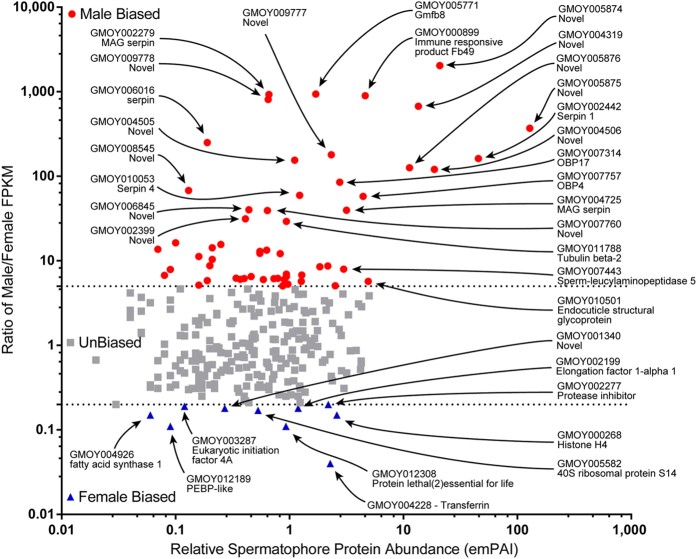
Spermatophore proteins are mostly encoded by male-biased genes. Relative protein abundance in the spermatophore (emPAI) as compared to the ratio of male/female whole body normalized expression (FPKM). Both axes are labeled with logarithmic values. Two genes, *serpin* GMOY004724 and the novel GMOY012285, displayed no expression in females (normalized expression values = 0) and thus could not be included in the expression fold change ratio calculations.

Considering that the tsetse spermatophore is assembled inside the female uterus, we evaluated the potential female contributions to its content. We mapped previously generated whole body transcriptomes from whole adult males and females^16^ against the representative genes coding for constituents of the spermatophore proteome to obtain a qualitative overview of the sex-bias of spermatophore protein gene expression. This analysis shows that 20% of the spermatophore protein-coding genes are enriched in their expression in the male transcriptome (n = 57, >5 fold higher normalized expression in males), whereas only 4% are enriched in the female transcriptome (n = 10; >5 fold higher normalized expression in females; <100 unique reads in combined male libraries). The remaining 76% are unbiased in their expression levels between the sexes (n = 217, <5 fold expression difference between males and females) (Fig. 3).

About one third of the genes with male-enriched profiles encode the majority of the novel proteins (n = 18), including the most abundant spermatophore protein GMOY005875 (Table 1). Male-biased genes include *Obp4, Obp17*, flagellar axoneme components (tubulin and dynein), several serpins, three predicted cuticular proteins, and aminopeptidase-coding genes, such as sperm leucylaminopeptidases. This group also contains the *Male-specific RNA 98Cb*-like gene, which in *Drosophila melanogaster* is suggested to play a structural role in the sperm tail.

Female-biased spermatophore-associated genes are limited not only in number, but also in their relative expression level. These genes exhibit a mean female/male expression ratio of 7.7, while the male-biased genes display a mean male/female expression ratio of 147.14 (Supplementary Data S1). One exception is *transferrin*, which is expressed 25 fold higher in females than in males (Fig. 3; Table 2). Other highly expressed female-biased genes include *phosphatidylethanolamine-binding protein* (PEBP), *protein lethal(2)essential for life-like* and a protease inhibitor (GMOY002277) (Table 2).

### MAG versus Testes Bias in Spermatophore Component Gene Expression

**Figure 4:**
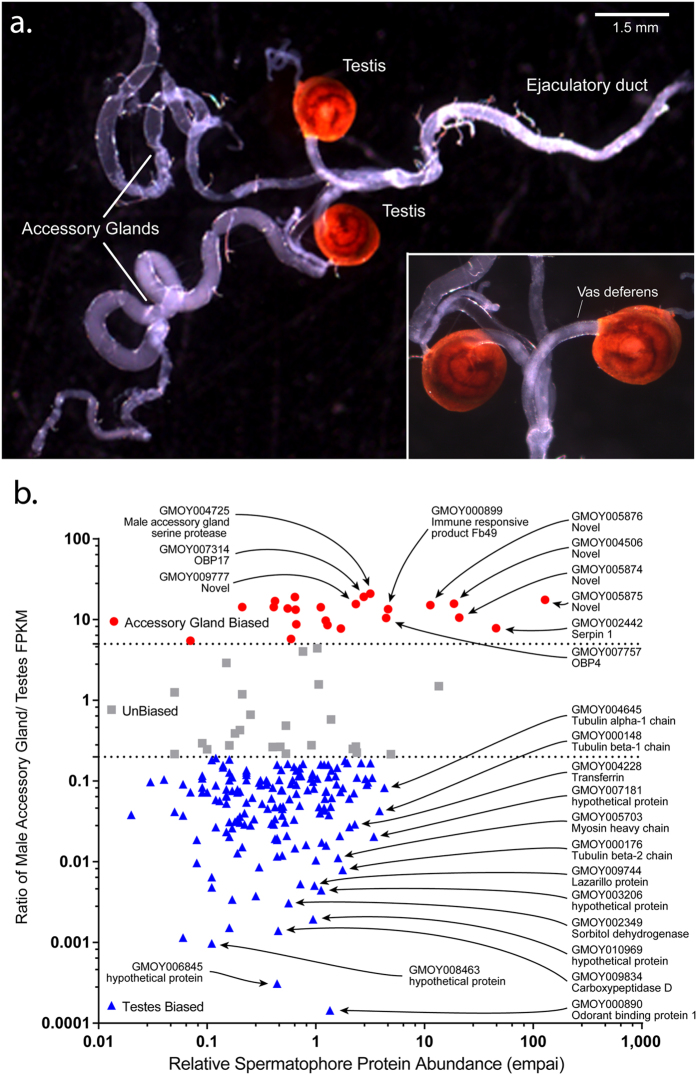
Spermatophore proteins are encoded by both testes- and MAGs-biased genes. (**a**) Male reproductive tract. Scale bar = 1.5 mm. A closer view of the testes and their respective deferent ducts are shown in the box. (**b**) Relative protein abundance in the spermatophore (emPAI) as compared to the ratio of MAG/Testes normalized expression (FPKM). Both axes are labeled with logarithmic values.

Considering that the tsetse spermatophore is thought to be primarily composed of SFPs produced by the MAGs^12^, we queried the role the testes play in spermatophore protein production. We developed testes- and MAG-specific RNAseq datasets covering multiple male physiological states (Fig. 4a). Sequencing of these libraries generated 131 million and 128 million reads from the testes and MAGs, respectively (Supplementary Table S1). These datasets were then cross-referenced with genes corresponding to the identified 287 spermatophore proteins. Of the transcripts associated with spermatophore proteins 55% (n = 151) were testes-biased (>5 fold higher in the testes), 6% (n = 17) were testes-specific (Table 3), 8% (n = 23) were MAG-biased (>5 fold higher in the MAGs) (Table 4) and none of them displayed a MAG-specific expression profile. In addition, 9% of the spermatophore-associated transcripts (n = 24) were unbiased in their expression between testes and MAGs (<5 fold difference between testes and MAG libraries) (Fig. 4b) and 22% (n = 62) display very low expression levels in MAGs and/or testes (<100 total unique reads between MAGs and testes). The ten genes identified as having female-biased expression were analyzed separately. Of these genes, three were unbiased between testes and MAGs, whereas seven displayed testes-biased expression (Supplementary Data S1).

**Figure 5:**
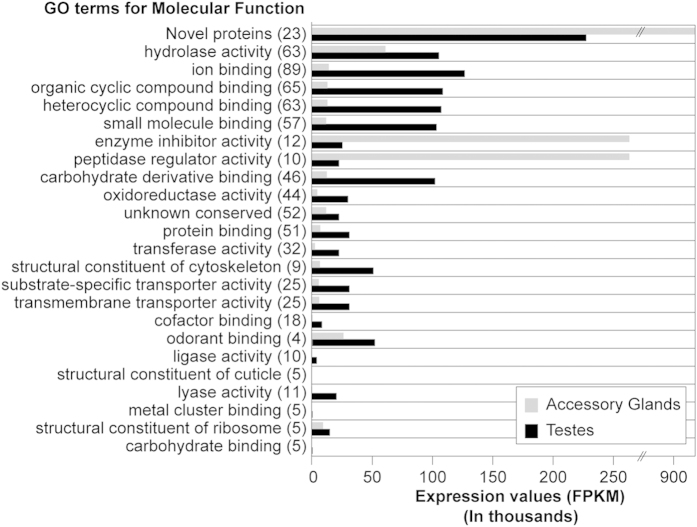
Relative expression of MAGs- and testes-biased genes encoding spermatophore protein classes. For each protein class (GO Molecular Function Level III, with descending emPAI values from the top of the graph), gene expression (FPKM) in MAGs and testes is shown, respectively.

We found that a small number of highly expressed MAG-biased genes encode the most abundant spermatophore proteins, including the novel proteins, enzyme inhibitors, and peptidase regulators. By contrast, a great number of testes-specific or testes-enriched genes with relatively lower expression levels encode a more diverse array of low abundance spermatophore proteins associated with binding, oxidoreductase, transferase and lyase activities, and also include cytoskeletal and transporter proteins (Fig. 5).

The testes-specific genes encode a number of proteins predicted to be associated with sperm, based on characterized orthologs from other insect species. These include proteins with structural (beta-2 tubulins, tektin), energy metabolism (NADH dehydrogenase, cytochrome-c oxidase, and sorbitol dehydrogenase), and sperm capacitation (apolipoprotein D) functions. Testes-specific genes also encode one novel protein (GMOY008545) and four sequences similar to uncharacterized *Musca domestica* (GMOY002531, GMOY009073, GMOY009618) and *Ceratitis capitata* (GMOY011501) predicted proteins (Table 3). Among the testes genes, we found that only the testes-specific apolipoprotein D and 26 testes-biased genes were predicted to encode proteins carrying classical secretory signals. These included for example gelsolin, one apolipophorin (GMOY005442), three novel proteins, OBP1, OBP99b-like, and the putative ortholog of the *M. domestica* ejaculatory bulb-specific protein 3-like (Supplementary Data S1). The MAG-biased genes encode novel proteins, five serpins, Obp4, Obp17 and a putative regulator of spermatid differentiation angiotensin-converting enzyme (ANCE) (Table 4; Supplementary Data S1).

### Comparison of Spermatophore and Semen Proteins from Insects with Different Mating Systems

**Figure 6:**
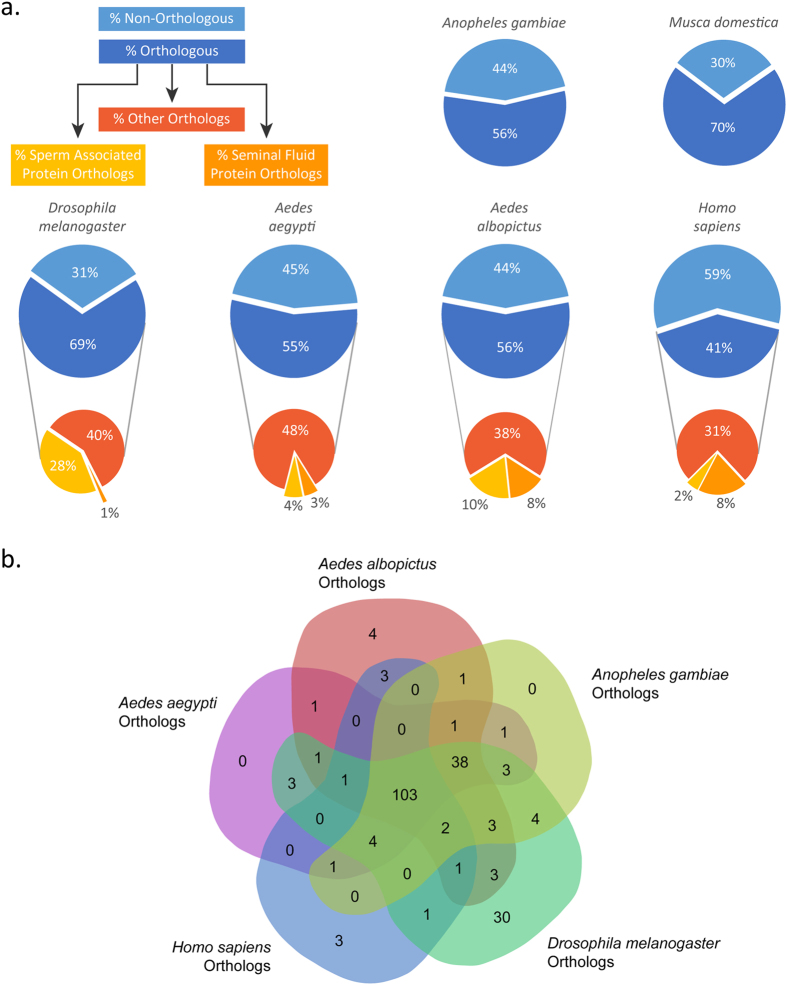
Orthology analysis with other characterized species. Comparative orthology analysis of the tsetse spermatophore proteins against *D. melanogaster, M. domestica, Ae. aegypti, Ae. albopictus, An. gambiae* and *H. sapiens*. (**a**) Pie charts represent the percent of spermatophore genes with positive reciprocal BLAST hits between *Glossina* and the respective compared species. (**b**) Venn diagram illustrating the number of common orthologs found between the compared species.

The level of conservation of tsetse spermatophore proteins was estimated with respect to the predicted proteomes and semen components of *D. melanogaster, Musca domestica, Anopheles gambiae, Aedes aegypti, Aedes albopictus* and *Homo sapiens*. These species display different levels of relatedness and modalities of ejaculate delivery, assembly and use to maximize their reproductive success^17^. Each tsetse spermatophore protein was thus first compared against the full proteomes of the above-mentioned species and then their respective SF and sperm proteomes, when available (Fig. 6; Supplementary Data 3). The reciprocal best hit approach was used as a proxy for the identification of putative orthologies^18^.

A large proportion of the 287 tsetse spermatophore proteins displayed reciprocal best BLAST hits against the full proteomes of the closely related *D. melanogaster* (n = 197; 69%) and *M. domestica*, for which the genome was recently released^19^. As expected, in *Musca* 70% (n = 201) of tsetse spermatophore proteins had reciprocal hits, but interestingly none of the novel proteins were conserved. In *Drosophila*, almost half of the orthologs generated reciprocal hits to sperm proteins (n = 80; 28%), whereas only 4 (1%) had reciprocal hits to SFPs.

Conversely, despite the high level of putative orthology of tsetse spermatophore proteins to the *An. gambiae* full proteome (n = 161; 56%), there were no reciprocal hits to characterized mating plug components^20^. The unavailability of a sperm proteome for this species did not allow further comparative analyses. In *Ae. aegypti*, 55% (n = 157) of tsetse spermatophore proteins had reciprocal hits, but only a few matched to SF and sperm proteins (3% and 4% of all spermatophore proteins, respectively). We also compared the tsetse proteins against the transcriptomes and recently identified semen components of the Asian tiger mosquito *Aedes albopictus*^21^. This species shares physiological features with *Aedes aegypti* including the kinetics of sperm migration to the spermathecae and coagulation of seminal secretions within the female *bursa inseminalis*^22^. This comparison revealed that 56% of the tsetse spermatophore proteins (n = 162) had reciprocal hits, with a slightly higher number of proteins that matched to SF and sperm proteins (8% and 10% of all spermatophore proteins, respectively) (Fig. 6a and Supplementary Data S3).

In addition, we explored whether any similarities were present between the spermatophore proteins of the viviparous tsetse and mammalian ejaculate. Of the tsetse spermatophore proteins, 41% (n = 119) had putative orthologs in the human (*Homo sapiens*) full proteome, 8% (n = 23) had human SFP orthologs, whereas only 2% (n = 7) were orthologs to human sperm proteins (Fig. 6a).

A global comparison across all the species shows that more than one third (n = 103) of *Glossina* spermatophore proteins have common orthologs not only across the considered insect species, but also in humans (Fig. 6b). However, none of the common proteins were unanimously classified as SF in all five species or in the Diptera alone. Conversely, one of the common proteins was unanimously classified as sperm protein in *Ae. aegypti, Ae. albopictus, D. melanogaster* and *H. sapiens* (i.e. the ATP synthase GMOY008764) and two (alpha-tubulin GMOY004645, and male-specific RNA 98cb-like GMOY006985) found in the three Dipteran alone (Supplementary Data S3).

## Discussion

Here we tried to reconstruct the sequential events that occur during copulation in *Glossina* to understand the modality in which the male assembles its ejaculate into the female uterus and uses it to maximize its reproductive success. Only towards the end of the mating process do sperm become visible in the uterus. They are found as an encapsulated mass (together with male seminal fluid) in the spermatophore, a structure that appears to be formed entirely within the female. A hypothesis from early researchers^15^ is that the MAG material, which enters the uterus first, forms a lining within which the spermatophore is later assembled. The spermatophore thus functions as a protective container for the ejaculate, ensuring that i) the spermatozoa reach the female spermathecae in a rapid, safe and efficient manner, and ii) seminal fluid components act as rapid modulators of many aspects of female post-mating physiology/behavior. Indeed, it has been shown that male secretions from the spermatophore rapidly enter the hemolymph of *G. morsitans* females^15^. The transfer of sperm via a closed system directly into the spermathecal ducts prevents the diffusion of sperm throughout the female reproductive tract and facilitates fertilization. Insemination in *G. morsitans* only occurs with the agency of a spermatophore. Its absence or incomplete presence in the uterus results in failure to fertilize^14^. This is also the case when males infected with the Salivary Gland Hypertrophy (SGH) virus mate with uninfected females. In these females, the spermatophore was found to be either incomplete, or absent from the uterus. No sperm were found in incomplete spermatophore, and the females remained un-inseminated^23^. This virus, in addition to its association with salivary gland enlargement, also induces testicular degeneration and appears to damage the MAG epithelium. As such, viral infection could possibly impair MAG protein synthesis, secretion, spermatophore formation and sperm transfer^24^.

In addition to its key role in fertilization, the tsetse spermatophore may impact sperm competitiveness. In *Glossina*, matings occur near the blood meal sources, creating a high probability for males to find virgin females. However, they are also likely to encounter mated females within the 48 hour window prior to complete attainment of their refractory state. This increases the chances for female remating. In this case, the transfer of free sperm into the uterus would be associated with a high risk of sperm mixing, even before the spermatozoa reach the spermathecae^25^. This risk is reduced in *Glossina* because in case of sequential female remating within the 48 hours non-refractory period, each male forms its own spermatophore. The spermatophore resulting from the first mating may act as a physical barrier for the sperm enclosed in the second one, possibly impeding proper sperm movement to the spermathecae. Whether this results in a preferential use of first male sperm in *G. m. morsitans*, as observed in the closely related tsetse species *G. austeni*^26^, and in other spermatophore-forming Dipteran species^25^, remains unknown. Female remating in *G. morsitans* can occur^27^, once the refractory state is over, allowing sperm mixing within the spermathecae^28^. However it is uncertain how polyandrous females utilize sperm from multiple males over time.

Given that the tsetse spermatophore is assembled inside the female uterus, it is conceivable that both sexes could contribute to its content. Indeed, our proteomics analysis coupled with tissue- and sex-specific transcriptomics data showed that spermatophore formation involves a bulk of proteins encoded by genes with unbiased expression between the sexes. However, a relevant proportion of spermatophore proteins are encoded by genes with male-biased expression, with limited female-biased products present. The male contribution comprises of not only MAG products, as previously assessed^29^, but also by testes-specific proteins that include both proteins predicted to be sperm structural components based on information from other insect species, and secreted proteins with likely important roles in ejaculate functions.

A small number of highly expressed MAG-biased genes encode the most abundant spermatophore proteins, including: novel proteins, enzyme inhibitors, and peptidase regulators. The novel proteins account for 8% of the genes from which spermatophore proteins are derived, however they represent the majority of the protein constituting the spermatophore. They may be involved in sexual selection and speciation, and may represent ideal subjects for future evolutionary analysis of tsetse species. This may be the case in the novel protein gene family consisting of the paralogs GMOY005874-5-6, which localizes to a single genomic locus on *G. m. morsitans* scaffold scf7180000648778^16^. They appear to have arisen as a result of gene duplication events, in accordance with the evolutionary patterns observed in other SFPs^30^.

The corresponding proteins contain six conserved cysteine residues, which is a common feature of Odorant Binding Proteins (OBPs). The presence of OBPs in male seminal fluid and their transfer to the female upon mating is common in insects^31^,^32^. We found two highly abundant OBPs within the spermatophore proteins which are represented by GmmOBP4, which was previously identified in the antennae, and GmmOBP17 the expression of which was previously undefined^33^. The identification of OBPs in non-olfactory tissues suggests that they may play roles other than chemoreception, for example transporting pheromones^34^ or other hydrophobic ligands in the female hemolymph. The high emPAI value of GMOY005874-5-6 and OBP4 may be alternatively explained if they are utilized as structural components of the spermatophore wall, as OBPs are found in the eggshells of *An. gambiae*^35^.

Other MAG-biased genes encode ANCE, serpins, and the evolutionary conserved nucleolar pre-rRNA processing ESF1. The tsetse *ance* gene (GMOY009723) may be involved in peptide processing, as its *Drosophila* counterpart is proposed to cleave the C terminus of reproduction-associated peptides to alter their biological activity^36^. In *Drosophila*, ANCE plays a relevant role in spermiogenesis, possibly processing a regulatory peptide required for spermatid differentiation. The processing of peptides in seminal fluid has also been proposed for the human prostate Angiotensin I-converting enzyme (ACE), which shares many of ANCE’s enzymatic properties. Serine protease inhibitors (serpins) and serine proteases are also found in the tsetse spermatophore. This suggests their potential involvement in sperm activation, regulation of proteolysis and other post-coital functions^37^. As proposed for other species, the transfer of serpins by tsetse males may provide a molecular component of an arms race between the sexes in which male-derived serpins inhibit female-derived proteases to obtain a reproductive advantage^38^.

Differing from the contribution by the MAG-biased proteins, we see a great number and diversity of testes-specific or testes-biased spermatophore proteins. This is likely due to the features of the sperm cell, which is rich in mitochondria and has a microtubule-based axoneme, containing a diverse repertoire of proteins with a wide spectrum of functional categories^39^. In the tsetse spermatophore, we indeed found abundant levels of the sperm axoneme-related proteins Beta-2 tubulin and Tektin^40^. Potentially related to sperm motility is Sorbitol dehydrogenase, which utilizes sorbitol to provide sperm with fructose for energy^41^. We also found Apolipoprotein D, which is a lipocalin that belongs to a class of proteins known to affect the acrosome reaction in mammals and involved in sperm capacitation^42^. Also important for spermatozoa function is gelsolin, which is proposed to be involved in the disassembly of F-actin cytoskeletons during capacitation in mammals, thereby promoting the acrosomal reaction^43^. Tsetse also transfers through the spermatophore a protein (GMOY012164) whose *Drosophila* putative ortholog, PEBIII, is a component of the posterior region of the mating plug^13^. PEBIII is induced by viral infection and may function as odor/pheromone binding protein pherokine^44^.

The mating process offers the opportunity for microorganisms from the male genitalia to be introduced into the female reproductive tract, causing infection. To protect the female and/or the sperm from this risk, antibacterial proteins, including glycine-rich peptides, are transferred from the male to the female in the seminal fluid of *Drosophila*^8^. Similar antibacterial functions may be played by the highly glycine-rich protein GMOY011501 detected in tsetse spermatophore.

Insect females secrete molecules from their reproductive epithelia that may influence fertilization, provide immune defense, or function in sperm maintenance, storage and release^45^. Nevertheless, only a few proteins were identified as clearly female-derived in the spermatophore. Among these, transferrin is the most abundant. Insect transferrins, in addition to their proposed roles as iron transporters, antibiotic agents, antioxidants and vitellogenic proteins^46^, are known to function as immune proteins^47^. Considering the relative abundance of transferrin in the spermatophore, we can speculate it contributes to the molecular machinery controlling the immune response induced by mating in the female. Indeed, mating results in the up-regulation of *Drosophila* females’ genes involved in immune/defense response^48^. Whether this is the case in tsetse is currently unknown and requires further investigation. The putative phosphatidylethanolamine-binding protein (PEBP) GMOY012189 may also be involved in the immune response. The *Drosophila* homolog of this protein (CG17919) is related to the innate immune response and has been detected in a proteomic analysis of the hemolymph after microbial infection^49^. Additional experiments are required to determine whether these putative female-derived proteins are directly included in the spermatophore content or they are structurally incorporated in the spermatophore wall during its formation.

Male reproductive fitness is directly related to the ability of the ejaculate to efficiently fertilize the female partner and prevent her from being fertilized by competing males. Different insects have developed varying strategies for ejaculate delivery and assembly. In *D. melanogaster*, mating plug components are assembled within the female uterus before and after sperm transfer to facilitate sperm movement into female reproductive tract^8^. In *An. gambiae*, the male transfers a structured fully coagulated gelatinous plug to the female *atrium* after sperm delivery^20^. *Aedes* mosquitoes do not produce mating plugs, but their MAG secretions contain granular materials that gradually cluster and solidify within the female *bursa inseminalis* at around 40 min post-insemination^50^. It remains to be seen if the use of a spermatophore is an adaptation associated with the development of viviparity in female tsetse. Components of the ejaculate also contribute to male reproductive fitness, and are subject to rapid change driven by the co-evolutionary dynamics between the two sexes^51^. The sex-specific costs and benefits associated with ejaculate composition plays a role in shaping this plasticity. Generally, the 287 proteins identified within the tsetse spermatophore follow the expected phylogenetic relationships among the considered insect taxa. In the case of *M. domestica*, it is noteworthy that none of the identified novel proteins are conserved. In *Musca*, ejaculate delivery indeed follows a different path, with complete sperm transfer being usually achieved within 10 minutes followed by the separate transfer of accessory seminal products^52^.

Although both sperm and SFPs participate in complementary reproductive functions, sperm-associated proteins are typically more conserved among the different insect taxa than SFPs, and did not show evidence of positive selection^39^,^53^. The high sequence conservation noted for sperm associated proteins may reflect essential sperm-specific processes they participate in. In contrast, SFPs may act as key factors in species-specific male reproductive success and as such, SFP-encoding genes may be subject to rapid evolution resulting from sexual conflict and competition^54^. Indeed, we found that tsetse predicted sperm proteins were also more conserved than SFPs, in particular when compared to the related *Drosophila*. Interestingly, SF components of the *An. gambiae* mating plug do not display any similarity to those of the tsetse spermatophore, and hence may reflect two divergent mating systems.

Insect SFPs have been studied as a model for seminal protein function in animals, including mammals^55^. The identification of a number of tsetse spermatophore proteins with reciprocal homology to human SFPs may be interesting, as it could potentially reveal functional similarities between such distant organisms.

## Conclusions

In this paper we attempted to touch one of the most interesting aspects of tsetse reproductive physiology, i.e. the formation and use of a spermatophore for ejaculate transfer and insemination. This modality of ejaculate delivery is part of the process leading to the maximization of reproductive success in this species. One would expect that such a peculiarity of this ejaculate delivery system would also be reflected at the molecular level. Indeed when we examined the protein components of spermatophore, we found that a great portion of it is represented by highly abundant novel proteins. Whether this species-specificity is related to the viviparous reproductive strategy of tsetse fly is an open question. The identification of these *G. m. morsitans*-specific proteins has evolutionary and applicative outcomes. Comparative genomics analyses of the identified novel proteins among the *Glossina* species for which genomic and transcriptomic resources are becoming available (i.e. *G. fuscipes fuscipes, G. palpalis gambiensis, G. brevipalpis, G. austeni*, and *G. pallidipes*) will allow for the measurement of the presence and extent of evolutionary pressures on these sequences. This knowledge will also favor the development of improved *Glossina*-specific population control strategies aimed at population reduction in the field. Indeed insect SFPs are being studied as potential agents for use in controlling the reproduction of insect vectors of diseases^56^ and tsetse’s low reproductive capacity and viviparous biology make it an ideal target for vector control. The identification of SFPs with a role in inducing female refractoriness to remating is of particular interest for the development of improved Sterile Insect Technique (SIT) applications^57^.

## Methods

### Tsetse fly samples

The *G. m. morsitans* flies used here are from a colony maintained in the Yale insectary since 1993 and established with puparia from wild samples from Zimbabwe. Newly emerged individuals were sexed at emergence and mating assays were performed at three to four days post-eclosion. Flies were reared at 24 ± 1 °C with 50–55% relative humidity, and fed with defibrinated bovine blood every 48 h using an artificial membrane system^58^.

### Mating and Spermatophore Formation

Spermatophore transfer to females was observed by interruption of copulation. We allowed four day-old males and females to engage in copulation and then interrupted the matings at 5, 30, 60, 75, or 100 min (3 mating pairs per time) while others were allowed to mate uninterrupted (15 pairs). Mating couples were transferred to ice, separated, and females were dissected to determine the status of the spermatophore. In addition, ten singly-mated females were exposed to a second mating immediately after the end of the first uninterrupted *copula* and flies were monitored to detect female remating. Twice-mated females were dissected as described above.

### Spermatophore collection and proteomic analyses

Tsetse individuals were sexed at emergence, fed and used for mating experiments on their fourth day. After CO^2^ anesthetization, one male and one female were transferred to a single mating cage. Cages were checked for coupled pairs approximately every 15 min. Coupled flies were left undisturbed and those that paired for at least 1 hour were processed for spermatophore collection^14^. After coupling, females were dissected in 1x PBS to isolate the spermatophore. In absence of any molecular information related to ejaculate composition in tsetse, we decided to derive a first comprehensive picture and thus we included in our proteomic analysis both the double-layered spermatophore wall^59^ and its contents, which comprises sperm and other male secretions. Seven whole spermatophores were pooled and stored at −20 °C until analysis by liquid chromatography tandem mass-spectrometry (LC/MS/MS) at the Yale Keck Biotechnology Resource Laboratory. After homogenization in 8M urea, peptides were separated with a Waters nanoACQUITY ultra high-pressure liquid chromatographs (UPLC) system (75 μm x 150 mm Ethylene Bridged Hybrid (BEH) C18 column eluted at 500 nl/min at 35 ºC) with Buffer A (100% water, 0.1% formic acid) and Buffer B (100% CH_3_CN, 0.075% formic acid). A linear gradient was established with 5% Buffer B at initial conditions, increasing to 50% Buffer B at 50 min and finally to 85% Buffer B at 51 min. Mass spectral data were acquired with an AB Sciex 5600 Triple Time-of-Flight mass spectrometer using 1 microscan followed by four MS/MS acquisitions. Neutral loss scans (MS^3^) were also obtained for 98.0, 49.0 and 32.7 atomic mass units (amu). Seven separate 1 μl injections at an estimated 0.351 μg/μl concentration for a total of 2.457 μg on the column were used for analysis. The Mascot algorithm was used to analyze un-interrupted MS/MS spectra^60^ after using the Mascot Distiller program to generate Mascot compatible files (http://www.matrixscience.com/home.html). The Mascot Distiller program combined sequential MS/MS scans from profile data that have the same precursor ion. A charge state of +2 and +3 were preferentially located with a signal to noise ratio of 1.2 or greater and a peak list was generated for searching the *G. m. morsitans* predicted peptide database^16^. Mascot scores were based on MOlecular Weight SEarch (MOWSE) relying on multiple matches of more than one peptide to the same predicted protein^61^. The MOWSE based ions score is equal to (−10)*(Log_10_P), where P is the absolute probability that a match is random. Matches were considered significant when the probability of a random match fell below 5% (e-value < 0.05). Thus, Mascot scores greater than 54 were above the significance threshold and they were considered in our analyses. The exponentially modified protein abundance index (emPAI) was employed to estimate the relative amounts of individual proteins^62^.

### Functional annotation of spermatophore proteins

Functional classification of the spermatophore proteins was performed using the Blast2GO software v.2.8 (https://www.blast2go.com/b2ghome)^63^. We performed genes searches using the transcripts encoding spermatophore proteins via BLASTx against the NCBI non-redundant (nr) database (e-value < 10^−10^). For Gene Ontology mapping (GO; http://www.geneontology.org) we used Blast2GO to extract GO terms associated with homologies identified by NCBI’s BLAST. We retained annotations with a minimum e-value 1 × 10^−10^. We then performed InterPro (InterProScan, EBI^64^) searches remotely from Blast2GO via the InterPro EBI web server and merged InterProScan GOs with the original GO annotations. For each of the identified Molecular Function (Level III) categories, the sum of the emPAI values in the spermatophore was derived. To derive information on the secretion mechanisms of the spermatophore proteins, putative secretory signal motifs were analyzed using SignalP 4.0 (http://www.cbs.dtu.dk/services/SignalP-4.0/)^65^.

### Transcriptomic resources utilized for determining sex-biased/specific expression

To assess sex-biased or sex-specific expression of the genes encoding spermatophore proteins, we used transcriptomic resources previously generated for gene prediction in the framework of the *G. m. morsitans* genome project^16^. Whole body female Illumina sequencing data were obtained from two libraries created from lactating and non-lactating (post parturition) females^66^. Whole body male transcriptome data were derived from two male libraries^16^. Library construction and sequencing were performed as described in Benoit *et al*.^66^. Sex specificity of spermatophore proteins was based on normalized gene expression calculated as the Fragments Per Kilobase of Exon per Million reads mapped (FPKM), analysed using the Baggerley’s test^67^ and filtered with the False Discovery Rate (FDR) P-value correction of 0.0001. We derived the fold change ratio between the male and female normalized expression means and defined a gene as male-biased when displaying a fold change ratio >2; we defined a gene as female-biased when displaying a fold change ratio <0.5. The intermediate ratios were considered unbiased. In addition, for a protein to be defined as male-biased, the corresponding gene had to have at least a combined number of 100 unique reads in the male whole body library. If this was not the case, the corresponding protein was defined as female-biased.

### Transcriptomic resources utilized for determining male tissue-biased/specific expression

Total RNA was separately extracted from testes and male accessory glands of i) teneral, ii) 3 days old virgin, and iii) 6–8 h post mating adult individuals. For each of the three time points, tissues were dissected from pools of 20 flies in 1X PBS, transferred immediately to cold Trizol Reagent (Ambion - Life Technologies, Carlsbad, CA). RNA isolation, Illumina library construction and sequencing were performed as described previously^66^. The Bioproject ID for these samples is PRJNA295435. The Sequence Read Archive numbers for the individual libraries at NCBI are SAMN04054396, SAMN04054395, SAMN04054394, SAMN04054393, SAMN04054392 and SAMN04054391. To determine Illumina read quality, we performed FastQC analyses on the transcriptomes derived from teneral, three day-old and mated adult flies. The sequences were then trimmed in CLC Genomics Workbench (CLC bio, Cambridge, MA) to remove ambiguous nucleotides. Reads were mapped to the predicted transcripts dataset from the *Glossina* genome with an algorithm allowing only two mismatches and a maximum of 10 hits per read. Transcript expression levels were analyzed using CLC Genomics Workbench (CLC bio, Cambridge, MA). As a measure of relative gene expression we used the Fragments Per Kilobase of Exon per Million reads mapped (FPKM) statistic^68^. We then combined the values for the three replicates corresponding to the testes and the MAG libraries, respectively, and derived for each transcript an average expression value for each of the two tissues. This allowed us to identify the tissue of origin of each spermatophore protein. For each individual sample, the proportion of read counts for each sequence relative to total read counts was determined to calculate the P-value differences in proportions by Z-tests following FDR correction. Fold changes were determined as the ratio of FPKM of testes vs. MAGs. In all comparisons, we considered one gene to be tissue-biased when fold change in one tissue was at least 5 fold higher in expression than in the other. One gene was considered to be tissue-specifically expressed when fold change in one tissue was at least 5 folds higher in expression than in the other, and the number of unique reads in the other tissue was less than 50.

### Determining sequence similarity between tsetse spermatophore proteins and SFPs from other Diptera

Putative orthologs of the *G. m. morsitans* spermatophore proteins were identified by comparing their sequence similarity to proteins from full predicted protein sets from five species: *Ae. aegypti* (AaegL3.3., Vectorbase), *An. gambiae* (AgamP4.2, Vectorbase), *D. melanogaster* (GCF 000001215.4 Release 6 plus ISO1 MT, NCBI), *H. sapiens* (UP000005640, Uniprot), *M. domestica* (MdomA1.1, Vectorbase), and *Ae. albopictus*. For this last species, we compared tsetse spermatophore proteins sequence similarity to proteins from the *Ae. albopictus* predicted protein list derived from a merged reference dataset including multiple transcriptomic resources^69^. BLASTp analyses (e-value < 10^−10^) were performed using CLC Genomics Workbench (CLC bio, Cambridge, MA). Protein sequences were defined as orthologs if they were reciprocal-best BLASTp hits having an e-value < 10^−10^.

To determine whether the predicted orthologs were previously reported as SFPs or sperm proteins, the orthologs were cross-referenced with published lists of SFPs and sperm proteins in their respective species: *Ae. aegypti* (93 SFPs and 52 predicted sperm proteins^70^); *Ae. albopictus* (198 SFPs and 116 sperm proteins^21^); *An. gambiae* (SFPs, i.e. 27 proteins identified in the mating plug, of which 15 were male-derived, 6 female-derived, and 6 with corresponding genes expressed in both male and female^20^; no sperm proteins have been published so far); *H. sapiens* (923 SFPs^71^; 98 sperm proteins^72^); *D. melanogaster* (157 SFPs^73^; 1108 sperm proteins^74^,^75^). The intersections among the five groups of tsetse orthologs in the different species were visualized by a venn diagram (http://bioinformatics.psb.ugent.be/webtools/Venn/).

## Additional Information

**Accession codes:** Sequence Read Archive numbers at NCBI: SAMN04054396, SAMN04054395, SAMN04054394, SAMN04054393, SAMN04054392 and SAMN04054391. 

**How to cite this article**: Scolari, F. *et al*. The Spermatophore in *Glossina morsitans morsitans*: Insights into Male Contributions to Reproduction. *Sci. Rep.* **6**, 20334; doi: 10.1038/srep20334 (2016).

## Supplementary Material

Supplementary Information

Supplementary Dataset 1

Supplementary Dataset 2

Supplementary Dataset 3

## Figures and Tables

**Table 1:** Fifty-seven transcripts encoding spermatophore proteins displaying male-biased expression.

Proposed Functional Class	Transcript Name – Description	Normalized expression	Protein emPAI
*Novel proteins (18)*			
	GMOY002399	491	0.41
	GMOY004319	1481	13.53
	GMOY004505	336	1.11
	GMOY004506	2352	18.68
	GMOY005874	6321	20.84
	GMOY005875	1300	128.43
	GMOY005876	578	11.33
	GMOY006845	1528	0.44
	GMOY006927	107	0.21
	GMOY006928	301	1.28
	GMOY007760	527	0.64
	GMOY008545	223	0.13
	GMOY008910	100	0.34
	GMOY009777	1231	2.33
	GMOY009778	1019	0.65
	GMOY000899* - *immune responsive product Fb49*	3721	4.62
	GMOY005771* - *Gmfb8*	685	1.7
Unknown Conserved Proteins (11)**			
	GMOY002531 - uncharacterized protein	91	0.9
	GMOY005107 - protein dj-1-like	30	0.37
	GMOY006985 - male-specific RNA 98Cb-like	71	0.07
	GMOY007027 - myosin heavy clone 203-like	27	2.17
	GMOY008628 - uncharacterized protein LOC101900116-like	117	0.55
	GMOY009348 - short spindle 5	34	0.08
	GMOY011142 - uncharacterized protein	147	0.21
	GMOY011236 - reduction in cnn dots isoform b	58	0.46
	GMOY011898 - glycine-rich cell wall structural protein 2-like	1978	0.94
	GMOY012011 - arylphorin subunit c223-like isoform x2	358	0.59
	GMOY012319 - glutamine-rich protein	76	0.16
*Odorant Binding Proteins (1)*			
	GMOY007314 - *Obp17*	436	2.76
	GMOY007757 - *Obp4*	764	4.43
*Transporter/Oxidoreductase activity (1)*			
	GMOY010969 - cytochrome c oxidase subunit 4 isoform mitochondrial-like	23	0.94
*Structural Constituent of Cytoskeleton/Binding/Hydrolase activity (1)*			
	GMOY011788 - *beta-2 tubulin*	524	0.94
*Structural Constituent of Cuticle (3)*			
	GMOY008996 - endocuticle structural glycoprotein abd-4-like	10536	0.63
	GMOY009277 - adult cuticle protein 65aa	2145	0.1
	GMOY010501 - cuticle protein	3337	4.91
*Protein Binding/Hydrolase activity/Ion Binding (5)*			
	GMOY008018 - dynein intermediate chain 2	93	0.09
	GMOY004744 - alpha-actinin sarcomeric-like isoform x1	6198	0.19
	GMOY000830 - wd repeat-containing protein 16-like	55	0.25
	GMOY004957 - tropomyosin isoform p	31402	1.85
	GMOY009671 - tektin isoform a	76	0.55
*Peptidase Regulator/Enzyme Inhibitor/Hydrolase activity (5)*			
	GMOY002442 - serpin 4	5094	45.56
	GMOY002279 - male accessory gland serine protease inhibitor-like	4673	0.66
	GMOY004725 - male accessory gland serine protease inhibitor-like	423	3.17
	GMOY006016 - collagen alpha-4 chain	2200	0.19
	GMOY010053 - serpin 4	216	1.23
*Oxidoreductase Activity (3)*			
	GMOY006971 - alcohol dehydrogenase	32	0.4
	GMOY007615 - malate mitochondrial-like	36	0.79
	GMOY010728 - phenoloxidase subunit a3-like	84	0.73
*Metal Cluster Binding/Oxidoreductase Activity/Protein Binding (2)*			
	GMOY007601 - NADH dehydrogenase	102	0.2
	GMOY011708 - succinate dehydrogenase	127	1.27
*Hydrolase activity/Ion Binding (6)*			
	GMOY007443 - sperm-leucylaminopeptidase 5	108	2.99
	GMOY007957 - sperm-leucylaminopeptidase 2	52	0.83
	GMOY008793 - cytosol aminopeptidase-like	23	2.52
	GMOY011554 - myosin light chain 2	27469	0.94
	GMOY011904 - cytosol aminopeptidase-like	23	0.97
	GMOY003835 - putative inner dynein arm light chain, axonemal	44	0.87
*Cofactor Binding/Oxidoreductase Activity/Ion Binding (1)*			
	GMOY002007 - NADP-dependent malic enzyme-like	5768	0.16

*Gmfb8 and *immune responsive product Fb49* have been previously identified in *Glossina*; **displayed similarity (BLASTp e-value < 10^−10^) to proteins of unknown function.

Normalized expression values in male whole body and emPAI of corresponding spermatophore protein are reported.

**Table 2:** Ten transcripts encoding spermatophore proteins displaying female-biased expression.

Proposed Functional Class	Transcript Name - Description	Normalized expression	Protein emPAI
*Amide Binding/Lyase/Transferase/Oxidoreductase/Binding/Hydrolase activity*			
	GMOY004926 - fatty acid synthase includes	12083	0.06
*Binding/Hydrolase activity*			
	GMOY002199 - elongation factor 1-alpha	18883	1.19
*Ion Binding*			
	GMOY012189 - PEBP homolog-like	2295	0.09
*Peptidase Regulator/Enzyme Inhibitor/Hydrolase activity*			
	GMOY002277 - protease inhibitor-like	9081	2.19
*Protein Binding/Compound binding*			
	GMOY000268 - histone partial	2839	2.61
*Structural Constituent of Ribosome*			
	GMOY005582 - ribosomal protein isoform b	11712	0.53
*Transporter/Ion Binding*			
	GMOY004228 - transferrin	57917	2.28
*Transcription Factor Binding/Hydrolase activity/Binding*			
	GMOY003287 - dead box ATP-dependent RNA helicase	6275	0.12
*Novel Protein*			
	GMOY001340	3753	0.27
Unknown Conserved Protein*			
	GMOY012308 - protein lethal essential for life-like isoform x1	25204	0.93

*displayed similarity (BLASTp e-value < 10^−10^) to proteins of unknown function.

The gene encoding the *WD repeat-containing protein* GMOY002068 was excluded from this list and from further analyses given its extremely low expression in both sexes (0 and 1 normalized expression values in male and female whole body, respectively).

Normalized expression values in female whole body and emPAI of corresponding spermatophore protein are reported.

**Table 3:** Seventeen transcripts encoding spermatophore proteins displaying testes-specific expression.

Proposed Functional Class	Transcript Name - Description	Normalized expression	Protein emPAI
*Novel Proteins (1)*			
	GMOY008545*	11.81	0.13
Unknown Conserved Proteins (4)**			
	GMOY002531 - uncharacterized protein*	1.77	0.9
	GMOY009073 - outer dense fiber protein 3-like protein 2	5.82	1
	GMOY009618 - uncharacterized protein	6.46	0.24
	GMOY011501 - gly-rich protein	3.26	0.04
*Structural Constituent of Cytoskeleton/Binding/Hydrolase activity (2)*			
	GMOY011788 - beta-2 tubulin*	5.16	0.94
	GMOY000176 - beta-2 tubulin	21.66	1.76
*Metal Cluster Binding/Oxidoreductase Activity/Protein Binding (1)*			
	GMOY007601 - NADH dehydrogenase*	12.58	0.2
*Pigment Binding (1)*			
	GMOY009744 - apolipoprotein d-like	200.06	0.97
*Transporter/Oxidoreductase Activity (2)*			
	GMOY007874 - mitochondrial uncoupling protein 4	15.23	0.49
	GMOY010969 - cytochrome c oxidase sub. 4 isoform mitochondrial-like*	67.69	0.94
*Carbohydrate & Substrate-Specific Transporter/Transmembrane Transporter Activity (1)*			
	GMOY012318 - facilitated trehalose transporter tret1-like	13.11	0.16
*Transferase/Oxidoreductase Activity/Ion Binding (2)*			
	GMOY002349 - sorbitol dehydrogenase	11.39	0.56
	GMOY003513 - citrate synthase	21.48	0.41
*Protein binding/Compound binding (2)*			
	GMOY009985 - histone h2b	53.50	1.32
	GMOY004552 - tektin isoform a	6.89	0.39
*Cofactor Binding/Oxidoreductase Activity/Ion and Compound Binding (1)*			
	GMOY006295 - succinate dehydrogenase	8.82	1

*displayed male-biased expression (see Table 1); **displayed similarity (BLASTp e-value < 10^−10^) to proteins of unknown function.

Normalized expression values in male testes and emPAI of corresponding spermatophore protein are reported.

**Table 4:** Twenty-three transcripts encoding spermatophore proteins display MAG-biased expression.

Proposed Functional Class	Transcript Name - Description	Normalized expression	Protein emPAI
*Novel Proteins (14)*			
	GMOY002399*	5750.25	0.41
	GMOY004505*	4166.84	1.11
	GMOY004506*	104795.17	18.68
	GMOY005874*	170459.66	20.84
	GMOY005875*	169512.53	128.43
	GMOY005876*	18367.88	11.33
	GMOY006927*	1162.76	0.21
	GMOY006928*	2195.32	1.28
	GMOY007760*	22726.50	0.64
	GMOY009777*	58246.99	2.33
	GMOY009778*	11312.84	0.65
	GMOY005771 - Gmfb8*	12248.56	1.7
	GMOY000899 - immune responsive product Fb49*	107047.76	4.62
Unknown Conserved Proteins (1)**			
	GMOY008628 - uncharacterized prot. LOC101900116*	500.68	0.55
*Protein Binding/Hydrolase activity (1)*			
	GMOY002550 - ESF1 homolog	2247.16	0.59
*Peptidase Regulator/Enzyme Inhibitor/Hydrolase Activity (5)*			
	GMOY002442 - serpin 4*	36267.19	45.56
	GMOY002279 - male accessory gland serpin -like*	113208.38	0.66
	GMOY004724 - n.a.	10930,17	0.42
	GMOY004725 - male accessory gland serpin -like*	15253.09	3.17
	GMOY010053 - serpin 4*	1982.44	1.23
*Odorant Binding Protein (1)*			
	GMOY007314 - Obp17*	20196.45	2.76
	GMOY007757 - Obp4*	19601.29	4.43
*Oxidoreductase/Hydrolase/Compound & Ion Binding (1)*			
	GMOY009723 - b chain crystal structure of *Drosophila* ance	296.82	0.07

*displayed male-biased expression (see Table 1); **displayed similarity (BLASTp e-value < 10^−10^) to proteins of unknown function.

Normalized expression values in the MAGs and emPAI of corresponding spermatophore protein are reported.
