# Supplementary material for: The Spermatophore in Glossina morsitans morsitans: Insights into Male Contributions to Reproduction
Source: Sci Rep. 2016 Feb 5;6:20334. doi: 10.1038/srep20334 (PMC4742874; doi:10.1038/srep20334)
Supplement: Supplementary Information [file srep20334-s1.pdf]

Supplemental Information

The Spermatophore in *Glossina morsitans morsitans*: Insights into Male Contributions to Reproduction

Francesca Scolari[1], Joshua B. Benoit[2,3], Veronika Michalkova[3,4], Emre Aksoy[3], Peter Takac[4], Adly Abd-Alla[5], Anna R. Malacrida[1], Serap Aksoy[3], Geoffrey M. Attardo[3]*

[1] University of Pavia, Dept of Biology and Biotechnology, 27100 Pavia, Italy

[2] University of Cincinnati, McMicken School of Arts and Sciences, Dept of Biological Sciences, 45221, Cincinnati, OH, USA

[3] Yale School of Public Health, Dept of Epidemiology of Microbial Diseases, 06520, New Haven, CT, USA

[4] Section of Molecular and Applied Zoology, Institute of Zoology, Slovak Academy of Sciences, 845 06 SR, Bratislava, Slovakia

[5] International Atomic Energy Agency, Joint FAO/IAEA Division of Nuclear Techniques in Food and Agriculture, IPC Laboratory, A-1400, Vienna, Austria

*geoffrey.attardo@yale.edu

Supplemental Fig. 1

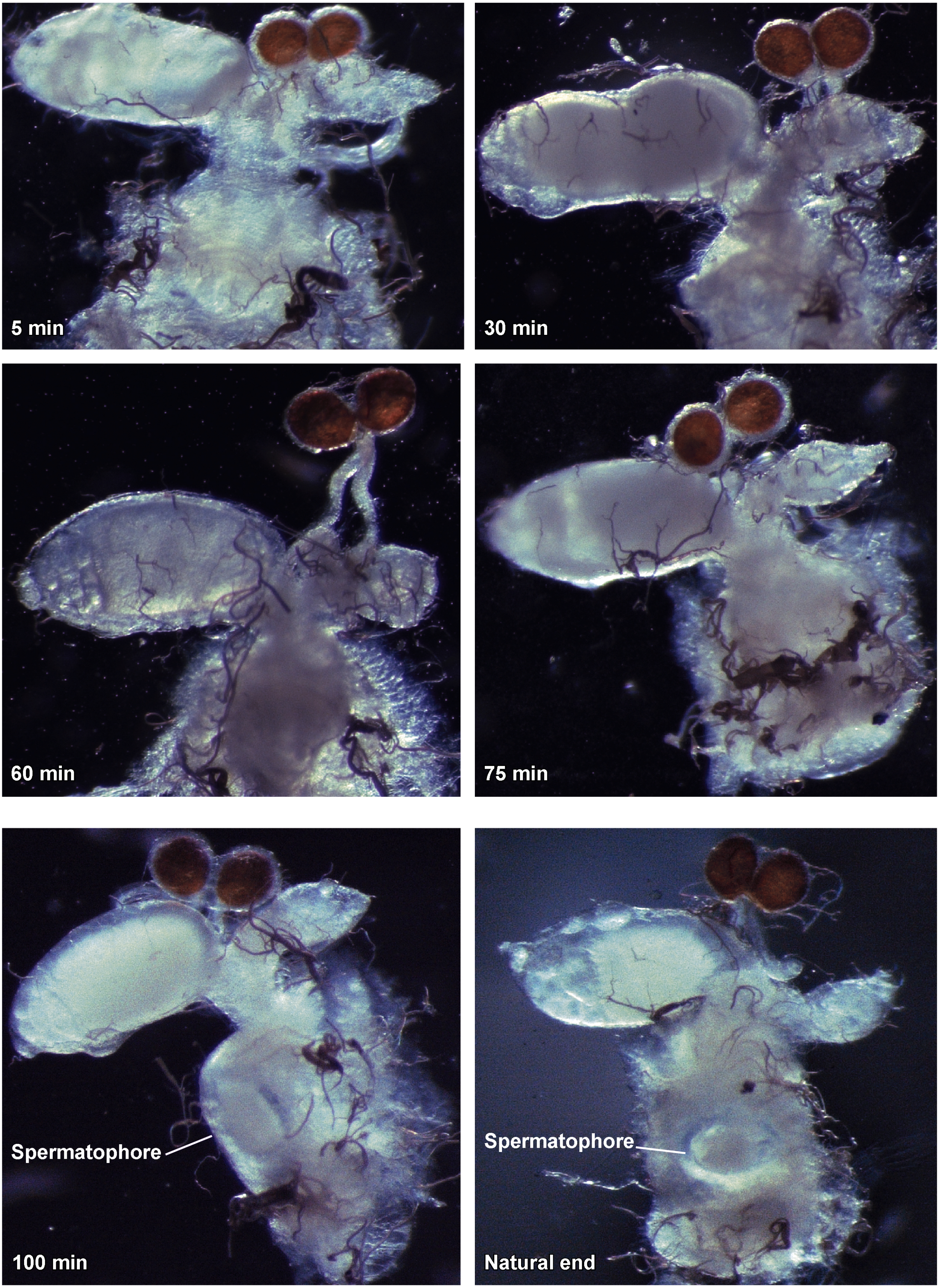

Table S1. Overview of testes and MAGs transcriptomes. The number of RNA-seq reads is shown, before and after quality control.

Tissue	Total RNA-seq reads	Reads after trimming	Percentage trimmed	Average lengh after trimming (bp)
Average MAG	128.565.696,67	126.765.703,33	98,60%	86,63
Average T	131.973.344,00	129.097.917,67	97,82%	85,63

Average read lenght before trimming was 101 bp.
