# Supplementary material for: The Spermatophore in Glossina morsitans morsitans: Insights into Male Contributions to Reproduction
Source: Sci Rep. 2016 Feb 5;6:20334. doi: 10.1038/srep20334 (PMC4742874; doi:10.1038/srep20334)
Supplement: Supplementary Dataset 2 [file srep20334-s3.doc]

>GMOY000023-PA hypothetical protein|protein_coding|scf7180000638894:91900-92229:-1|gene:GMOY000023

MVFRTIIAQVADKRVPLIKFRKGGGQLLPSSSSRHSQTHQAGPNSTLVESQPTGAIEDWE

LPSRYKRLPITADEMEYINRGGPA

>GMOY000024-PA hypothetical protein|protein_coding|scf7180000638905:15789-25683:1|gene:GMOY000024

MANKTFYIPALIGSIFFLTFVTAQEKPVTDICLGCICEAISGCNQTAICSGGVCGLFRIT

WAYWADAGKLTLGNDSVERELDYSNCVNDPYCAANTIQNYMTRFGQDCNDDKVVDCYDYA

AIHKLGAYGCRGDLPHQYYQVLDTCLKYHQSGNHLDVRSDAK

>GMOY000148-PA Tubulin beta-1 chain |protein_coding|scf7180000639097:26770-31615:-1|gene:GMOY000148

MREIVHIQAGQCGNQIGAKFWEIISDEHGIDATGAYHGDSDLQLERINVYYNEASGGKYV

PRAVLVDLEPGTMDSVRSGPFGQIFRPDNFVFGQSGAGNNWAKGHYTEGAELVDSVLDVV

RKEAESCDCLQGFQLTHSLGGGTGSGMGTLLISKIREEYPDRIMNTYSVVPSPKVSDTVV

EPYNATLSVHQLVENTDETYCIDNEALYDICFRTLKLTTPTYGDLNHLVSLTMSGVTTCL

RFPGQLNADLRKLAVNMVPFPRLHFFMPGFAPLTSRGSQQYRALTVPELTQQMFDAKNMM

AACDPRHGRYLTVAAIFRGRMSMKEVDEQMLNIQNKNSSYFVEWIPNNVKTAVCDIPPRG

LKMSATFIGNSTAIQELFKRISEQFTAMFRRKAFLHWYTGEGMDEMEFTEAESNMNDLVS

EYQQYQEATADEDAEFEEEQEAEVEEN

>GMOY000176-PA hypothetical protein|protein_coding|scf7180000639142:1109-6491:1|gene:GMOY000176

MREIVHMQAGQCGNQIGGKFWEVISDEHCIDATGTYYGDSDLQLERINIYYNEATGAKYV

PRAILVDLEPGTMDSVRSGAFGQIFKPDNFVFGQSGAGNNWAKGHYTEGAELVDSVLDVI

RKEAEGCDCLQGFQLTHSLGGGTGSGMGTLLLSKIREEYPDRIMCTFSVVPSPKVSDTVV

EPYNATLSVYQLMENTDETFCIDNEALYDICFRTLKLTTPTYGDLNHLVSATMSGVTTCL

RFPGQLNADLRKLAVNMVPFPRLHFFMPGFAPLTSRGSQQYRALTVPELTQQMFDAKNMM

AACDPRHGRYLTVAAIFRGRMSMKEVDEQMLNIQQKNSSFFVEWIPNNCKTAVCDIPPRG

LKMSATFLGNSTAIQELFKRVSEQFTAMFRRKAFLHWYTGEGMDEMEFTEAESSMNDLVS

EYQQYQEATAEEEGFDKTKKKMREIVHVQAGQCGNQIGDATGTYYGDSDLQLERINVYYN

EASGSKYVPRAVLVDLEPGTMDSVRSSTFGQIFRPDNFVFGQSGAGNNWAKGHYTEGAEL

VDSVLDVVRKETEGCDCLQGFQLTHSLGGGTGSGMGTLLISKIREEFPDRIINTFSVVPS

PKVSDTVVEPYNATLSVHQLVENTDETYCIDNEALYDICFRTLKLTTPTYGDLNHLVSAT

MSGVTTCLRFPGQLNADLRKLAVNMVPFPRLHFFMPGFAPLTSRGSQQYRALTVPELTQQ

MFDAKNMMAACDPRHGRYLTVAAIFRGRMSMKEVDEQMLNIQNKNSSFFVEWIPNNCKTA

VCDIPPRGLKMSATFIGNSTAIQELFKRVSEQFTAMFRRKAFLHWYTGEGMDEMEFTEAE

SNMNDLVSEYQQYQEATAEEEGEF

>GMOY000267-PA hypothetical protein|protein_coding|scf7180000639349:12-385:1|gene:GMOY000267

MSGRGGKAKGKAKSRSNRAGLQFPVGRIHRLLRKGNYAERVGAGAPVYLAAVMEYLAAEV

LELAGNAARDNKKTRIIPRHLQLAIRNDEELNKLLSGVTIAQGGVLPNIQAVLLPKKTEK

KA

>GMOY000268-PA hypothetical protein|protein_coding|scf7180000639349:889-3203:-1|gene:GMOY000268

MTATINLLKIFFKNNKIKTTGRGNGGKGLGKGGAKRHRKVLRDNIQGITKPAIRRLARRG

GVKRISGLIYEETRGVLKVFLENVIRDAVTYTEHAKRKTVTAMDVVYALKRQGRTLYGFG

G

>GMOY000340-PA mitochondrial chaperonin|protein_coding|scf7180000639576:24426-25057:1|gene:GMOY000340

MAAQAIKKIMPMLDRILVQRAESLTTTKGGIVLPEKSQAKMMQGTVVAVGPGARNNQTGA

HIAPSVKEGDRVLLPEYGGTKVELEDKKEYLLFRESDILAKYE

>GMOY000430-PA elongation factor 2|protein_coding|scf7180000639782:15281-18767:1|gene:GMOY000430

MVNFTVDEIRGLMDKKRNIRNMSVIAHVDHGKSTLTDSLVSKAGIIAGAKAGETRFTDTR

KDEQERCITIKSTAISMYFEVEDKDLVFITNADQREKDCKGFLINLIDSPGHVDFSSEVT

AALRVTDGALVVVDCVSGVCVQTETVLRQAIAERIKPILFMNKMDRALLELQLDAEELYQ

TFQRIVENVNVIIATYNDDGGPMGEVRVDPSKGSVGFGSGLHGWAFTLKQFAEMYAEKFK

IDVVKLMNRLWGENFFNAKTKKWQKQKEADNKRSFCMYILDPIYKVFDAIMNYKKEEIPT

LLEKIGVALKHEDKDKDGKALLKVVMRTWLPAGEALLQMIAIHLPSPVVAQKYRMEMLYE

GPLDDEAAVAVKNCDPEGPLMMYISKMVPTSDKGRFYAFGRVFSGKVATGQKCRIMGPNY

VPGKKEDLYEKAIQRTILMMGRYVEAIEDVPSGNICGLVGVDQFLVKTGTITTFKDAHNM

KVMKFSVSPVVRVAVEPKNPADLPKLVEGLKRLAKSDPMVQCIIEESGEHIIAGAGELHL

EICLKDLEEDHACIPLKKSDPVVSYRETVFEESNQQCLSKSPNKHNRLIMKAMPMPDGLP

EDIDNGDVSSKDDFKARARYLAEKYDYDVTEARKIWCFGPDGTGPNFILDCTKSVQYLNE

IKDSVVAGFQWATKEGIMAEENMRGVRFNIYDVTLHADAIHRGGGQIIPTTRRCLYASAI

TASPRLMEPVYLCEIQCPEVAVGGIYGVLNRRRGHVFEESQVVGTPMFVVKAYLPVNESF

GFTADLRSNTGGQAFPQCVFDHWQVLPGDPCEPNSKPYQIVQDTRKRKGLKEGLPDLSQY

LDKL

>GMOY000442-PA hypothetical protein|protein_coding|scf7180000639810:1817-2356:1|gene:GMOY000442

MAESGDSSEIGQLDKDFQELAKKLETEFLPKLSYREKLLATEWLVKLRNTKGDIKERKLR

NRFTKHFLETPKVFSGAKFKDLPANFQDPLEQLRQLLPKTPDEALNPTNEEKLTYISELF

ANLPDRGQFLASLPVPRAGSFYILLTSPTQETNKEEKKN

>GMOY000497-PA hypothetical protein|protein_coding|scf7180000639963:26952-40695:1|gene:GMOY000497

MADATKRNIPIKLGDFSVIDTEFSSIRERFDAEMRKMEEEMAKFRHELMNRESNFFESTS

ATNASRPKQNYISDINSPLIQEDGDNKVLKLRFDVSQYAPEEIVVKTVDQKLLVHAKHEE

KSDTKSVYREYNREFLLPKGVNPETIRSSLSKDGVLTVDAPLPAITSGETMIPITHK

>GMOY000508-PA mitochondrial phosphate carrier protein|protein_coding|scf7180000639967:55644-56965:1|gene:GMOY000508

MFSLLEVAKNSPFRTPFTKVQCDVPSPGLQGPVPGRHIQAAAAATESCEFGSSKYFALCS

IGGILSCGTTHTFVVPLDLVKCRLQVDQAKYKNLFTGFKITVAEEGYKGLAKGWAPTLFG

YSAQGAFKFGLYEVFKVYYAKALGEENAYLYRTTLYLAASASAEFFADMALAPFEASKVK

IQTTPGFANTLREAMPKIMNEEGLNGFYKGLVPLWMRQIPYTMMKFACFERTLELLYKYV

VPKPRAECSKGEQLVVTFAAGYIAGVFCAIVSHPADVVVSKLNQAKGSSALDVTRSLGFM

GMWQGLMPRIVMIGTLTALQWFIYDGVKVALAIPRPPPPEMPASLKEKLAK

>GMOY000582-PA hypothetical protein|protein_coding|scf7180000640123:100992-102814:1|gene:GMOY000582

MMLPMIKIVLRFSHIKEFGLFSRFVKNCQLPGTIKPFSSMMMQRVTAFLAPMAVEATLQI

QQQRGMATLKAISIRLKSVKNIQKITQSMKMVSAAKYSRAERELKAARPYGVGAQQFFEK

TDIKADEKEAPKKLLIGITSDRGLCGAVHTGVARFIRGELGKDDTNTKIFCVGDKSRSIL

ARLYGKNILMVANEVGRLPPTFLDASRIAHEVLKCGYDYTEGKIVYNRFKSVVSYQCTSL

PIYGAPTVEKSDKLVLYDSLDSEVIKSYLEFSLASLIFYTMKEGACSEQSSRMTAMDNAS

KNAGEMIEKLTLTFNRTRQAVITRELIEIISGAAALD

>GMOY000776-PA hypothetical protein|protein_coding|scf7180000640438:78259-83267:1|gene:GMOY000776

MTTTTKNTSSPLNDDELSDITKKEIAFWSKKLNLSPSELFTLSNEDLEKRLNKAQGREVN

DEERKRQTRRWERLNEMKSKLIERQEGRRLKGGANPNIWDIMIRAAAELDEVVGGGSYDY

EDDYDDSSSCPSPGDISYDENYEENFNADAVLAASTPVESPSGAGNVAYNADAARNIRPN

DTYTASPGATVRSATFTRNATYASPRGAVRSTSRTYVQCPPRAANQTYKVSTPEAKALAE

SPAQSNQTYAVPSDRTYTAPSDRTYSVPRERTFNVAKDRTYAVPSDRTYNVNYDRNVPLG

QTYVHCIDRTYPRPYPCPGGQRTYNVTPGSGSGSDSMYEPSSPYSPSLSPEAVTPPRATP

PPQFGSPGGSGSQGFIYQSTPVQRYRNYTEYCMRFGSDGCNETGNSISVENYDANSGMVQ

AASRTYNIPRAADATYPAPRAGDATYTAARAGDGVCRCPTNPQSRDAAYNVGANPSPNVQ

GYAGGAAAQGSPANNPPCPHAGDIEDCIVTPIIQGQFTPLHEAICDVIMDLTTVGHSATI

ENVRNHLEVSFPHMTTPSVEVVYDTLAQLMQEQKIYQTAKGYFILTPERRRSRSRPRSHH

NGCTDLDESLNSGPQEVRTILMTNVEALHSLYGEISTERDGDLTHQCIQTNLADVICGGN

SNDKILYPRTSKRRSSSFPTPRSLERRHSLRLFGSSKRLQRCSSTRSLSKAYAQTLHNTD

SSSSEYQSTDSSSPKKGSLLSRLFRRSGRNKSRQLETYSAQFPPVEWFNSKAVHLHSVGT

QTADHDLPTIRTLSNSTFYDGLELSQRSLTLPRRHRRQLSSESTFLISSRDCSPIRRRSP

VYSSSSLPRSTQSIPATANNSTKGAITNTTNSFSARLSNPYKCPNSQKSGQISRHILEHS

PSRSTNSSKMVISSNKYQDNKCEQRNLASGPSSLESGKTSTFNSGPSSIESSKASFNNKT

SGHSSLDSQRSNSSTTIQPKHQTRSIIMLNGSPRGTPRHQIMRARNAEQASSFNHQPAKP

ATTPTISNLSANASTTSFTLTPDRTHTLENYESSSAPISNSTSNNSITLKVTTSNDTQNS

LPNTKIVVQNTPAQSVITFENSQLTENSSVFIINNETTTNERGEVLRKTLSKHPNPLDTT

ASTKTSQSVIDQFADNERIQSTNYKTRSSILKENYLKDQYNYNVPDTFDISAVTSSNRST

VQQKLATTKSLYNETTMNNSRKLSVPSTLLTNNSNRLVYKNNVLRGDGSQTVSSNDEKSH

DDKPNVEISGSMGNLRFFEKNSRDGLTKAINSNCELNNLRYESAIPSLPAKPTKLNSNSS

LSGNNLVHILQKNSIGIGSEPNLSQKDNNCVLTADEKSIKKESLNRRFSITKEPSTEEGT

DELCNFPSLTDLSFNFTSLAAQKILQGVSLNSIDTLVELNMAAAAAAASVNTTMEKSQNN

QNTVCTDYGLV

>GMOY000781-PA hypothetical protein|protein_coding|scf7180000640453:50222-111408:1|gene:GMOY000781

MAQNFLYMFEVVVDDLLVTRPNYCAPEEYPTCCEVSFRNSVFLSICDREFGQCVDPTSPK

CGKCCLFSLDAPINDTDKLLIHIYKKKTNKCKFLVGCTDMPIKGLFDKVMESFNIENPKW

EDTAKKHITSIPNPHEPKPSEIIDNDCDDDSMGRREQLCPTSELTKRLLPLFNLKGSQTG

NVVLIIRLVANGPAIVSSFPFARICQAGCGAKPPNCSPKQCPKSCPDSTTNKCNGADDIL

PNIGRCCGGNDTAFGASGTSKTGATSSAKESSKDDKCDAPSSSTATYGNKSACGGRKVYK

NHKDPCNGCPALEDPCKKEEKKLRCMRYFACNADKGCPCDNVEDDCERGKQKQQACKPPK

CNCPESTKKSSGGSKDKDYNKSQACSCDECMDECEAVSEDDRFDPCKVADSSRTNARFVD

PTEPDPGPDAYEEFEACLNGSGLIIRVLKDTHQVQNICDGTENFDNAESASDCECPQTNS

QNSQCNLNELLQRSDFARNQVKRRTGGHIINHPKLPKIRANIKYSGNDYCCPDSYHVPFS

RLKQFCDTQENKVDAYRRRNPCDRGARAPLDSDDNRNCCVQVDRDDIKNALQGVNADTRK

KGIEVCYRTCEETDSDVFLVKLGSKHKSQKKKNNIEIELKTPKQPLVLPKQKVTTQTQIN

EAELDAALTGVCKGKGKGKGKGKESAFELDPTEIARQNQITQQILTHYIERRLFPFSNVK

QKVPSIAEILPKAITSTTSPRAISIATSPTYKRGLASSNNTRRKTYGQKLKNSTRRKYNA

NKIETHDKEEQEIEQRPSQQRLTENINEDEDATEQPATITYSNKYKNQDNHTFYYNNEWS

GGRGFLLPYDIEDDVEHDVQEALQSVESEQYDQYYNNDIFQRDPKENEIDGGGDCPNCVD

EVHYMNKWTMPLLKLGEKRYYLGIFFKANWFKATQYCRYHGMHLASISSQEENDRLEKYI

RDYGLGHEHFWISGTDLADEGNFFWMSTGRPITFTNWNAGEPNNFRYENGEEENCLELWN

RDGKGLKWNDSPCSFETYFVCEVQPN

>GMOY000806-PA hypothetical protein|protein_coding|scf7180000640485:19656-21308:1|gene:GMOY000806

MLRLVRLNTSRDIVKRAIQMRKYASAALNQMLQLQNTELCADPPLRGLVLGVYADEGDKS

DPGILTPTAWKYNVQKTGGRLINVLRMSGPMPKTGQARIFFCPESEKIPYYTAVAIVGLG

RECLGYNPYEVIDEQKEVIRRSVAKACMDLALIDSVRIDVEHCGHTESAAEGAALGIWAY

QELRAKKNRITVPVIDLYTVKDEVCDIEGWRIGLQKAAAQNLTRQLQEMPSNILTPTAFA

QHVVEVLCKSGVNVEVKVEGWAESQVMNAFLSVGKASCEPPIFLELSYYGTSADERPIVL

IGQGITYDCGGLCLKPLEKLQWMRGDMTGAAVVVATCRAIAGLRLPVNMRGLIPLCENVI

GCNSFRSGDTVKCMNGKHIKIQGTNHEDVLVLADTLLYAQNFCPKLIVDVGTPSWYMRDL

LGEAACGVFTNSEILWQQINHASMHTGDRVWRLPLWNYYTEQVTTDMSADVQNYGIGSGG

RPCKAAAFLREFVPCGQWMHIDATNVMYTTGKRFEYLREGMAGRPTRTLVEFIAQTICKD

TAPKLPKKGQ

>GMOY000825-PA hypothetical protein|protein_coding|scf7180000640488:120250-135556:-1|gene:GMOY000825

MDFQITNDVVEIEPKTKYLRGSELILLRNKDVGIPSAGLKKTIPKDAEEEVKELEKLYDC

LEQQLSRSHIERLAGRTLAEWDHEAQTVRVIRKDGKFEHFGFNKHGQHYLEYYEALFLLE

KNRLQLEYNSCVVSIEQSYLLLIGEKRSQRCDEYLVYSIMTRSGYILNKYENRPKAHSHR

KQEIRTREDCIWHILEMEIEQEKKSQKEQIVPDYVRKYIDYTKIKQQFQQIKESIKTPEK

RTQNIGYKDCENDLDFRKKMQTNLKRPAQDDNDCNYWHKIKRNCTLSTPGTATANTSNES

LIDFLKTDVEYERFRETFHKLNIIPLKSYDTENEDAKDDSSAEILPINFDVYLHNEGYRK

SSPGSPQFRLFMCHQMAMQKEANDKQKAEELRRQQSCFKVPIIWVLGGPGSGKGTQCEKI

VQKYGFVHLSTGDLLRAEVASGSPKGKDLAIIMKEGRLVSNDAVLDYPREKNQGVAFEQK

IAPVDLIIYFECEDETLINRIMGRAAASTEKRADDNEQTVKARILTFRGNTNAILSQYTD

RTLTLNAERTVDEIFCDVTCALDCLLQKKNLKASDR

>GMOY000830-PA hypothetical protein|protein_coding|scf7180000640494:189076-192680:-1|gene:GMOY000830

MTEAVQNDVEVEKLNCRAIFGINGKVNFGLHLHPDGRHMIFPLGMKIGIDDIKTSTQEFI

AGHTNNLSCLDLSRSGKYMASGQINHMGFPAYVILWDFDKRRELARHDLHKVRAQSVCFT

ARDKYVISIGGRDDGSVIVFDIETRTPICRSVASRGINGNPEVVNALNNNPSFFVTAGDR

HLRLWSIQREMKKVNVQDIFMGKQQRRMCCVGIYRGDEYAYMGTYSGDLMKVALNCCDVV

NVTQVGQTSGLVGAYGVHNPRKPFGKDCNRYVHGVRVLRIVDDGLLLVGAGDGVVELVEE

RKDINEIVFKNYPNPTCPMLKMLKRTKVNGAVSSLVEINPLEYYIATDLNEIYLLNIKTF

SLKLLKTSHKKAVYAIVFPRNLSNVFATAGYETIRVWSTKRLQELLRIMVYNFNCAALAF

SFDGTSIVSAWNDGVIRSFTPITGRLIYAIPNAHNKGCSALAISSTGRVLVTGGIEGQVR

VWKIEPLRQSLLGVLKDHSAPITTLDFNKFDTEVISASSDGSCVIWDIKRLTRKTVVAAN

TQFMTAKYFPTGVQFLTCGTDGCISYWMVYNGSLIRELQASKKSSVNHVAINATGDYFAS

VGSDQMVKLWDYNRGNVVAEGHEHASAVISVAYSPSRKFFVTGCTDGAIIIWDVPEKYWG

QENPPPVEEPIVEEKPKSKPETPSHPQAKSSRGNTSKGENIEGLVASTPKNNVCCVECPP

ITAISKKVANENCNIVPNVKTC

>GMOY000849-PA hypothetical protein|protein_coding|scf7180000640584:6921-11049:1|gene:GMOY000849

MEGETKDSEAKDSEAKDSKKRRKPKPKPKPKPPPEPIVYLGKTAKINAKLLKAKQRVPPQ

KKPEKKEEKKEGEEEPPPEKKEKEEKKVQKSQKKVAKAKAEAKKKSEDAELARLVKRQEL

KAKYKEIIKVREKRKKKEEPEKKLCTITKGKAKAVVEEDKKPGSGSGENLKDTIYHDMSM

DIDPQEHRPKTEPELLLVPKYYKFEEIKSKVEAKEEPLTELFKKTFQMYKAQEVMLAFNG

GKDCTVILHMLDLFFQKNHCLKHLKIPTLFITDPDGFPEVEEFVNDCAKLYNIELIKRPG

TIKEALDEICKEKPLIRAIFMGSRRTDPHCQDLKVMQPTDPGWPPLMRINPILDWTCRDV

WQYMYVYNVPYCILYQRGFTSIGNKKNSKPNPYLRVIESTTGRVLDYRPGHELLDNDNLE

RAGRGMSRAGQDVLGVLRGIEMVVEAVLQERSKSCKHIWINSSLREVIEKNFKTGSEYMQ

TATGNPMAELKKLQEFIGETGERTYVVTEGLRQLVGTKVSQELRMRQMPDAPSETKESGK

VLNASDNISLHRNVDAANLDISSITLEELEDILSKRHKDREISLRTTATKSKQIINLQKP

TETSNRSDQLVQVRNSETVEAAKTPGDETLRADTKYVKNVLSFVAGAPAHLGSTSQIELP

ILSKVAKQRRVPASRLGRMASFGGLFAGLGFGTINELTKGALGLGGSKNMKEALLSPANA

ERIVDTLCKVRGAALKIGQILSLQDSNVVSPQLAKAFERVRQAADYMPDWQVERVMSTQL

GPEWRNLLQKFDEKPFAAASIGQVHRAILQNGMQVAIKIQYPGVAQSIESDIDNLVGMLK

VWDVFPHGFFIDNVVKVAKRELTWEVDYTREAEYTEKFKEMIAAYPEYYVPIVIKQMTTA

NVLTTELVPGVPLDKCFDLRYEYRKHIGESILKLCLRELFELQCMQTDPNWSNFLYDVKS

KRLMLIDFGSTRFYPKKFIKNYRNVIISAVNNDRQNVLKISREMGFLTGYESKQMEEAHV

DAVMILGEMFRCESEFDFGRQNITERIAHLVPTMVAHRLCPPPEEIYSIHRKLSGIFLLC

ARLNIRLNCRPLYDEIVISKFKE

>GMOY000890-PA Odorant binding protein 1|protein_coding|scf7180000640662:1165-1692:1|gene:GMOY000890

MKTTAVILLALFALVSADYKLRNQEDLNKARKECMEAKKVTPELVEKYKKFDFPDDEITR

CYIECIFDKFQLFDSQTGFKNDNLIAQLGQSKDNKDEVKADIEKCADKNTEKSDSCTWAF

RGFKCFISKNLPLVMESLKKN

>GMOY000899-PA hypothetical protein|protein_coding|scf7180000640665:19025-29929:1|gene:GMOY000899

MNFYFVHSKIAIKGTSSFNSSFKSFKAMVNYQVRVAVFLVALLGAHEVRGVSMWSPETAI

TSFQIGVFLSGINYYCYAIRSAREENAVEEESAVNKSTVHELEKDNEKISKINTDYSSNL

TASVNAVENTVRSSKLENADIDKKEMQTIEATMVNYQVQVGILLVALLGGHEVAGLSMWS

SKTAISSFHIGVFLSGVNYYCYAIRLAREENAVEDETVSNKSTIMELERDNEKIAKTNTD

YSSNLTANVNAVENSIRSSKLENADIDRKENQKLEIEETIGPDGRTLISNSDAATQRKTI

RNSKELKRYKKYLLRYLFTTKRFKAMVNYQVQVGILLVALLGGHEVAGLSMWSSKTAISS

FHIGVFLSGVNYYCYAIRLAREENAVEDETVSNKSTVMELERDNEKIAKTNTDYSSNLTA

NINAVENSIRSSKLENADIDRKENQKLEIEETIGPDGRTLISNSDAATQRKTIRNSKELK

RYKK

>GMOY001043-PA hypothetical protein|protein_coding|scf7180000640844:6029-8160:-1|gene:GMOY001043

MPKYNKICFVRQCGTLKRLNNMRYSVVFSLLLLILGLSWAQNKASPTGSTTSIGPQKHLV

CYYDSSSFVKEGLGKMVIDDLEPALQFCTCLVYGYAGIERDSYKAVSMNQNLDLDLGKGL

YRSVTKLKRKYPHLKVLLSVGGDKDIETGEDAKDLPNKYLELLENPTGRMRFINTAYALV

KTYGFDGLDVAWQFNKNKPKKVHSGLGSLWKGFKKTFTGDHIVDENAETHKEQYTALMRE

LKNEFRPENLLLSTTVLPNVNSSLFYDVPSVMNYVDFVNLAAFDFYTPERNPELADLPAP

LYPLPERNPEFSVDSQVQYWLRQGCPANKLNVGVPTYGRPWKMTDDSGLTGVPPVPKVEN

EAPQGPNTLIPGLYSWQEVCSLLPNTNNMYSKGADAPLAKVLDPQKKSGVYAYRVADKKG

KNGIWVGYEDPDTGAEKAGYVKTLNLGGIALYDLSLDDFRGLCTGDKYPILRAIKYRLVN

>GMOY001219-PA translation elongation factor EF-1 gamma|protein_coding|scf7180000641051:1827-3369:1|gene:GMOY001219

MATGTLYTYPDNFRAFKALIAAQYSGASIKLAENFKFGETNKTPEFLKKFPSGKVPAFET

PNGQYLSESNAIAYFVANEHLRGGKCPFAQAQVHQWMSFADNEIVPASCALVFPLLGIMP

QKRGSTARQDVEEVLALLNEKLLNCTYLVSERITLADIVVFCSLLHVYQYVMEPSARKPY

GNTNRWFMTLLNQPQFKAVLKTFKVCEHALVFDPKKYAEFLAKTGQAGGDAKQQKQEKKP

KEEKKKPVKEAEYEEKEEMDATEEALASEPKSKDPFESLPKGKFNFDDFKKVYSNEDEAV

SIPYFWDKFDPENCSIWFGEYKYNEELTKTFMSCNLISGMFQRLDKMRKQAFASMCLFGG

DNDSTISGIWVWRGQDLAFTLSPDWQVDYEVYDWKKLDPNSEETKKLVQQYFSWTGTDKD

GRKFNQGKIFK

>GMOY001238-PA hypothetical protein|protein_coding|scf7180000641097:11067-21257:-1|gene:GMOY001238

MQVDPVICAFIALFLATLCSTGTIPNRPALPTQTNQIQPSNADSKSPGRRIMHPAFANAG

RSPGLEIWRIENFEPVAYPKNNYGKFYTGDSFIVLNTVQTKDKKLSWDVHFWLGSETSVD

EAGAAAILTVQLDDLLNGGPVQHREVQDHESQLFLSYFKNGVRYEQGGIASSFKHVEINA

QGEKRLFQVKGKRNVRVRQVNLSVSSMNKGDCFILDAGNEIYVYVGAQAKRVEKLKAISA

ANQIRDQDHNGRARVHIIGNSDADKGSFFDILGSGSPGQVPDESTAEEDGAFETTDANSV

TLYKVSDASGSLKIDPISQKPLRQEMLNSQDCFILDTGSGIYVWVGRGATQKEKTDSLSK

AQEFLRSKKYPAWTQIHRIVEGAESAPFKQYFATWRDVGMSHTRLVRSALGYDSDNSEFD

VDDVDSVLKTLKEKGGRAIGFMPDNGRNELSDITVYSSVPGTNDVQKEKTSYTETLPLKS

HNAYVIPYNYHDKNDETGTLVYVWEGVKAANANEYAFEDALTLAVGENAILVRTVQNHEP

RHFLKMFKGKLFTIVNNTPTVPQLFHIRGTDADDVHAYETKADSSSLSSSDVYVLFVHNE

DKAFIWIGLGASEFEKNAAEDLFKSVWPSVALSTVEEGAEPDEFWEHLNGEGIYDRSLSE

KSAPILEPRLFHCRLINNKIKVEEIMHFEQADLDFEDVMLMDTGDEIYLWVGSGASAEEN

GRILDIAKKYIAFEPTDRTIDTVTVVRISQDYEPHVFKRMFPSWEKGYWENIPTYEDVRK

KVVEDNERIEDNDV

>GMOY001277-PA |protein_coding|scf7180000641192:60782-74961:-1|gene:GMOY001277

MAANGVILLLTLTLFSVSSINTQYYSDFMDHALMLPFQQQQQQQQQQLPSPQSYPPILTR

YFARTAPQALQPQLQVPQQQLQQQYSLTWPQRPNNNIFNSYEPEAEFVPLEEQQRHQQNN

QQTGRHNEEKPFDVEVVSGDINGGSSHLPGIFFHQAFPFLGNSFFNSFGGFGIGTQQEPW

WKGPNVCTEKEEDEKVMDENDGNEETEVADGTDTTVQVKQPYFGQFHFSMNSCVEKPSKY

ICKRVVNKNGRKKTLTITRQCCHGYGRPRNAAYATPCDKIEIKDVESTAADVGAKEFVIK

ARNSGLNDMITSRKNLTIFLPVDEAFSSYSDLLQSESNLVEKQNDDGMKKLFLRHVVDGE

VSMDEVRNEQVFKTQVDGQNIRINSYQVPLSLSPEPYRYTANCVPILKHDKLTEQGMIHT

LDGIMKPVDKNLMDIIRDRRDMSIMRTVLEKTKLSDLLEGEKPLTIFVPTDDAFDKLEPH

LRRVLKDGKGCASNILKNHMLDLTFCSIASVPGAKTTAYNLLGEPMRFNRSTHGSETEQP

QPIVINGAAKIVETDIMGTNGVLHVIDTIMPTETALPLSSLMQEKNVTIFRRLLEISGLD

NAFDDMDNLTMFAPTDKALEGTEWAKMLEENPEQLINNRNLNEFLSYHVTKPMTKTCDLK

EQLMPTIGGGNVRINLFSTHSLFTNVMNRATANCARLVHFDDESCGSVLHQVDKPLIPPK

MNLLEMLQNNPNYSKFLELVQAANLTDLLVNPEEDYTLLVPKNDVFEELDENITKDPAEL

ESLIKTHIVNDVICCAGIIPTNWPFVRSIESLNGHHLRITRDRRPKIQNAGITKCDAMAT

NGIIHEMNDIVVPSSRQSQQPQRPHQHFSHHPQEDIFSDLFF

>GMOY001323-PA hypothetical protein|protein_coding|scf7180000641282:5518-6419:1|gene:GMOY001323

MLAARILLKNSSLKATAIRTFQTTFPVMAKVGDTLPSVELFEGSPANKINISELTAKKKV

VIFGVPGAFTPGCSKTHLPGYVDSADELKKELNVNEIICISVNDPFVMSAWGKEHGADGK

VRMLADPSAAFVKAMDLTIDLPPLGGIRSKRFSMIVEDAKVLALNVEPDGTGLSCSLAQN

IKNK

>GMOY001340-PA hypothetical protein|protein_coding|scf7180000641283:15512-23364:-1|gene:GMOY001340

MICFGRGLKVTGVFSHIDEINSGQTHNSYNPSAPPAPNSGGGGNSAGYGGGFSAGGHSLY

PTLPTDSNSGGVSGGAGGYRPYGGGSSGGYQPGGYQPGGYQPGGYQPGGGYAQPAPGYGG

RGGYSGGNGGGAGSGGYTPSKPKEKDGFFSSFFSNPAVSQAVTGIIAGQIASTLRGGGAG

GNQGNQGMQQPGGGYGGGAPSSSSPGSNFLGGLLTNVLTGAGGSGHGTPSSSGSSASNFL

GSLLSGGGTGGNRGSSSGTSGGLGDIFSSRNFGGLFSENPSSRSGSSGSSQPASSSGPKS

YPTQPPVNGNYYNMG

>GMOY001525-PA hypothetical protein|protein_coding|scf7180000641491:115440-116963:-1|gene:GMOY001525

MFTAVRSALTRSDKKVISLLAQSQREYAKTAAKAGAAGAGKVVAVIGAVVDVQFEDDLPP

ILNALEVDKRTPRLVLEVAQHLGENVVRTIAMDGTEGLVRGQKVLDTGFPIRIPVGAETL

GRIINVIGEPIDERGPVPTDKRAPIHAEAPEFVEMSVEQEILVTGIKVVDLLAPYAKGGK

IGLFGGAGVGKTVLIMELINNVAKAHGGYSVFAGVGERTREGNDLYNEMIESGVISLKDK

TSKVALVYGQMNEPPGARARVALTGLTVAEYFRDQEGQDVLLFIDNIFRFTQAGSEVSAL

LGRIPSAVGYQPTLATDMGSMQERITTTKKGSITSVQAIYVPADDLTDPAPATTFAHLDA

TTVLSRAIAELGIYPAVDPLDSTSRIMDPNIIGHEHYNVARGVQKILQDYKSLQDIIAIL

GMDELSEEDKLTVARARKIQRFLSQPFQVAEVFTGHAGKLVPLEQTIKGFTQILAGEYDH

LPEVAFYMVGPIEEVVEKADRLAKEAA

>GMOY001589-PA enoyl-CoA hydratase 2|protein_coding|scf7180000641554:79886-85019:1|gene:GMOY001589

MVEGELPDPGDKQLLIKIALAPINPYDLYNILGKFHTGPKKFPYTGGSEFVGEVVKTGPA

YSAYARGDIVIPLTLDSGAWTTYKLVNESDVFKVPNEVGLQEAASTAITACTAYRLLRDF

VRLSEGDSVILSGANSAVGQMILQLCKLWKLNSIGIVRDRDNVVNLKEDLKSLGATEILT

DKEIDRTDLFETYPLLKPKLALDCIGGTIGSMIAQRLVKDGFMISYGEMSNEPVYAESNQ

FVMQENVFMVRHSCSLTINMANIAKIFTNRAQYMLQVAAKQSQIVPRFYSAAANFEFIKT

ELAGANKNVAVITLNRPKALNALCNGLMKELSSVLDDYEKDKNVAAIIITGSEKAFAAGA

DIKEMQPNTYPHCILSNFLNDWTRVAKCQKPIIAAVNGYALGGGCELAMMCDIIYAGDKA

KFGQPEIALGTIPGAGGTQRLTRVVGKSKAMEMCLTGNMISAEEAEKMGLVSKVVPADKL

VSEAIKLGEKIGSHSNLIVQLCKESVNTAYETTLQEGLKFERRTFHATFSTNDRKEGMTA

FVEKRPAKFSNN

>GMOY001776-PA actin|protein_coding|scf7180000642081:269290-271349:-1|gene:GMOY001776

MCDDEVAALVVDNGSGMCKAGFAGDDAPRAVFPSIVGRPRHQGVMVGMGQKDSYVGDEAQ

SKRGILTLKYPIEHGIITNWDDMEKIWHHTFYNELRVAPEEHPVLLTEAPLNPKANREKM

TQIMFETFNSPAMYVAIQAVLSLYASGRTTGIVLDSGDGVSHTVPIYEGYALPHAILRLD

LAGRDLTDYLMKILTERGYSFTTTAEREIVRDIKEKLCYVALDFEQEMATAAASTSLEKS

YELPDGQVITIGNERFRCPESLFQPSFLGMESCGIHETVYNSIMKCDVDIRKDLYANIVM

SGGTTMYPGIADRMQKEITALAPSTIKIKIIAPPERKYSVWIGGSILASLSTFQQMWISK

EEYDESGPGIVHRKCF

>GMOY002000-PA hypothetical protein|protein_coding|scf7180000642602:70579-76500:-1|gene:GMOY002000

MKEIENIIHIEIWDPLHMYCAWCKKVKYTYNEASRTACTFLTMDLYYLPGSAPCRSVIMT

AKALGLQLNKKLLNLMAGEQMKPEFLKLNPQHTIPTLVDGDFSIWESRAIMVYLVEKYGK

TDSLFPKCPKKRAIINQRLYFDMGTLYKSFADYYYPQIFSKAPADPEMHKKIETAFDFLN

TFLEGQQYAAGDTLTVADIALLATVSTFEVAGFDFSKYPNVAKWYANAKTVTPGFDENWQ

GCLEFKKFFN

>GMOY002007-PA hypothetical protein|protein_coding|scf7180000642602:107813-113373:1|gene:GMOY002007

MESNTGDSTPLKFIAIMNVTAITKGRLRVESLDQSVCLYGNLTKICGNMRTTALVNPSSV

TAAAASSPTPCSNASVRQYHEVVGDIVCPSQVRGIDHIRDPRLNKGLAFTLEERQILGIH

GLQPARFKTMEEQLELCKIAVNRYMEPLNKYLYLADLADRNEHLYFRFLYDNIEDLMPIV

YTPTVGLACQRFGLIYRRPRGLFITVNDRGHVFDILRNWPEPDVRAICVTDGERILGLGD

LGANGMGIPVGKLALYTALAGIKPHQCLPVLIDVGTNSYDLLEDPLYVGLRQKRVVGKEY

DDFIDEFMEAVVKRYGQNTLIQFEDFGNQNAFRFLDKFRNTYCTFNDDIQGTAAVAVAGL

YASKRITNRNFADSTFVFAGAGEAAIGIASLCVKAMVDEGVDEKTALSKIWMVDIDGLLT

TTRKKGTISTHQTKFAKDVEPLDKLEDIVTKYKPNVLIGASAAAGIFTPKILQTMAANNE

RPIVFALSNPTSKAECTAEQAYQNTDGRVVFSSGSPFPPVTINNKTFTPGQGNNAYIFPG

VALGVIATGTHHIPEDMFLISAKELANFVEQSDLDRGSLYPPLKSIHEVSMRIASAITSY

AYKEGLASTYPEPECKREWLKEQLYNFNYESSMPVTWAWPRMPYFKTRELQPQKLYGKTL

H

>GMOY002029-PA hypothetical protein|protein_coding|scf7180000642607:3510-5697:-1|gene:GMOY002029

MNSASKEHVLAVTRDFISQPRLTYKTVSGVNGPLVILDEVKFPKFAEIVQLRLADGTVRS

GQVLEVSGSKAVVQVFEGTSGIDAKNTLCEFTGDILRTPVSEDMLGRVFNGSGKPIDKGP

PILAEDFLDIQGQPINPWSRIYPEEMIQTGISAIDVMNSIARGQKIPIFSAAGLPHNEIA

AQICRQAGLVKIPGKSVLDDQEDNFAIVFAAMGVNMETARFFKQDFEENGSMENVCLFLN

LANDPTIERIITPRLALTAAEFLAYQCEKHVLVILTDMSSYAEALREVSAAREEVPGRRG

FPGYMYTDLATIYERAGRVEGRNGSITQIPILTMPNDDITHPIPDLTGYITEGQIYVDRQ

LHNRQIYPPVNVLPSLSRLMKSAIGEGMTRKDHSDVSNQLYACYAIGKDVQAMKAVVGEE

ALTPDDLLYLEFLTKFEKNFIAQGNYENRTVFDSLDIGWQLLRIFPKEMLKRIPASTLAE

FYPRDARH

>GMOY002068-PA hypothetical protein|protein_coding|scf7180000642647:12454-13865:1|gene:GMOY002068

MTEAVQNDVEVEKLNCRAIFGINGKVNFGLHLHPDGRHMIFPLGMKIGIDDIKTSTQEFI

AGHTNNLSCLDLSRSGKYMASGQINHMGFPAYVILWDFDKRRELARHDLHKVRAQSVCFT

ARDKYVISIGGRDDGSVIVFDIETRTPICRSVASRGINGNPEVVNALNNNPSFFVTAGDR

HLRLWSIQREMKKVNVQDIFMGKQQRRMCCVGIYRGDEYAYM

>GMOY002199-PA hypothetical protein|protein_coding|scf7180000642974:49446-50837:1|gene:GMOY002199

MGKEKTHINIVVIGHVDSGKSTTTGHLIYKCGGIDKRTIEKFEKEAQEMGKGSFKYAWVL

DKLKAERERGITIDIALWKFETSKYYVTIIDAPGHRDFIKNMITGTSQADCAVLIVAAGT

GEFEAGISKNGQTREHALLAFTLGVKQLIVGVNKMDSSEPPYSEARYEEIKKEVSSYIKK

IGYNPAAVAFVPISGWHGDNMLEPSSNMSWFKGWKIERKEGNAEGKTLIDALDAILPPSR

PTEKPLRLPLQDVYKIGGIGTVPVGRVETGVLKPGCVVVFAPANITTEVKSVEMHHEALP

EAVPGDNVGFNVKNVSVKELRRGYVAGDSKNNPPKGAADFTAQVIVLNHPGQIANGYTPV

LDCHTAHIACKFAEIKEKVDRRSGKTTETDPKFIKSGDAAIVNLVPSKPLCVESFQEFPP

LGRFAVRDMRQTVAVGVIKSVNFKDATGGKVTKAAEKATKGKK

>GMOY002243-PA hypothetical protein|protein_coding|scf7180000643132:24882-38261:1|gene:GMOY002243

MSDRKAVIKNADMSEEMQQDAVDCATQALEKYNIEKDIAAFIKKEFDKKYNPTWHCIVGR

NFGSYVTHETRHFIYFYLGQVAILLFKSG

>GMOY002277-PA hypothetical protein|protein_coding|scf7180000643204:6768-7063:-1|gene:GMOY002277

MKFLLFLLSVVIVLCAVVQAADKSADCSLPKEVGPCRGSKLNFYYDSDAKTCKEFFYGGC

QGNANRFDTQEECQQLCH

>GMOY002279-PA hypothetical protein|protein_coding|scf7180000643204:19975-20830:1|gene:GMOY002279

MKFLLLSFLSIVALVASQDGPAMCYMEHSANGRDASQCAGGNFFWSYNAGANSCVKFYYF

GCWGNENRFLTQAQCELLCKKTEEKDNESNKANEEVSKSNEEVSKPNEEIGEPNEELEAD

PE

>GMOY002349-PA hypothetical protein|protein_coding|scf7180000643447:31807-41300:-1|gene:GMOY002349

MLTRFSAKNFGNCVKLFSNFQRNFSANDKKKSNKKGKNNKKSDKGKGDKPENLAVVLHAK

QDLKMETKPIAKIEPKEVLLAMDCVGICGTDIQLWQKGKLGPDVVKGPLVLGHEASGVVC

EIGKDVKDLKVGDRVAIEPGTFCRNCQVCRQGRYNLCPFMKFPSYPPTDGLLQRYFKQNS

DMCHKLPDHLTMEAGALCELLAVGVAGARRAKVKLNSKVLIVGSGPTGLATSIVCQAIGA

SKVMVIDKKEDRLEMAKGFGNMIMPLDDSDEKDLNKTAKKIHDCMGCIPDKVFDCHGSQE

TFKLAIRSTGWGGTCCLTGMASGNFTDFPLMDDVMRELLITANFRYCNDFPAAVAILANS

KYDLLKMITHHIEFDDALCAFDTALKQEPGTMKVMVHIVPQGTNNPKEKKSEPKPVPIVN

AKGKQFMIFQRKYPEKLFLLEVVLAIDCAGICGTDVHFWQEGRIGSNIVTEPAVLGHEAS

GVVWEVGFKVKHVRVGDRVVIEPGKCCRNCKACRLRRYNLCPFMRFPPFPSVDGLLQRYI

QQDADMCYKLPDHLTMEEGVLCAPLAVGVAAVRRAKVGSNSKVLIVSSAPIGLATSIVCQ

AIGVTKSHSNWRFAYRAALGIVSQGKYDLLKMITHHFTMENASCAFDKAYKQEPGTMKVI

VHMVQRNTNNKNVKKNSKINEKRTGSAENMSVVLCAEQDLKLEPRPMPEIGPRDVLLAMD

CVGICGTDIHLWKEGKIGADVLNSPVVLGHEASGVVCDLGRKVKNLQAGDRVVIEPGKFC

RDCEMCRRGRYNLCPFMQFHSCPPTDGLLQNYITHDANMCHKLPDHLTMEEGALCEPLTV

GVAAARRAKVKLSSNVLIVGAGPIGIATAIVCQTFGATKVMVIDKKQDRLEKAAGFGNMT

MALGDRDGREYNKTAQKIHERMGCIPDKVFDCHGSQETFKLAIRSTSWGGTCCLVGMASG

NFTNFPLMDDVMREVEITANFRYCNDFPSAINIAAHCKYDLSNLVSHRYDLAEALCAFET

ACKQEKGTMKVMIHMLPQEAYNEKE

>GMOY002377-PA hypothetical protein|protein_coding|scf7180000643566:75824-106403:1|gene:GMOY002377

MSSSQAIRSSKYSYRATSTGPGAADINIEYVADLSALSRLEDKIRLLQDDLEVERELRQR

IEREKADLSVQVIQMSERLEEAEGGAEHQFEANRKRDAELIKLRKMLEDVHLESEETAML

LKKKHNEIISDFQEQVEILTKSKSRAEKEKSKFQAEIYELLSQIESYNKEKLMSSKTIER

LEITITELNIKIEELNRTVIDITSHKTRLSQENIELIKDVQDLKSQLDTVSFSKSQVISQ

LEDARRRLEDEDRRRSMLESSLHQVESELESIRIQLEEESEARIDLERQLVKANADATSW

QNKYLAEAAARAEECEEIRRKYQVRITELEEHVEVLIVKINNLEKQKTRLQSEVEVLIID

LEKSNNSCRELQKSVTILEKHNIELKTRLDETIILFENSQRDLKNKQADLQRAIHELEKV

KDANNQLARENKKMGDDLHDAKAALNEMNRRLHEMELELRRLENEREELTAAYKEAEAGR

KAEEQRAQRVSADFNQYRHDAERRLTEKDEEIECIRERGLKQYRPSNVIRQYKTDDLLRL

SRAAAARADEILLDFRVKKRSPFSVQKLVDAARVTKHIAPDTVVERQRERRRRRQRELED

QIQQDITKLLFRIKKMELESGEMTKEFKRQIRGKSATAIAQALLTESERNIKNSKREEEE

YMSQAMIRSSRAASRLRSVSPEGGRVSTHTLHIELMDDRLVDNLDHRVSSSLHNVKRQLS

SLNQKTVEFYADSSKQTALEIEQLNARVIEAETRLKTEVQRIKKKLQIQITELEMSLDVA

NKTNIDLQKVIKKQSLQLTELQAHYEDVQRQLQTTLDQYGVAQRRLASMNGELEEVRSQL

DSASRAKRTVELQFEEAQTRINELHVTNVNLVSMKSKLEQELSVVATDYEEVTKELRISD

ERYQKVSVELKHTIEQIHEEQERIVKLETIKKSLEVEVKNLSVRLEEVELNAVAGSKRII

SKLEARVRDLELELEEEKRRHAETIKILRKKERTVKEVMVQCEEDQRNIALLQEALDKSV

AKEGMSQQSVTRVRRFQRELEAAEERAEVAETNLNMVRAKHRTFVTTSTVPGSQVFIQET

TRTITE

>GMOY002399-PA hypothetical protein|protein_coding|scf7180000643593:155471-157613:-1|gene:GMOY002399

MRENSLKTVAVSVLLIILFLHPNDCMPDDEETTTPPADTLEDTTSTPIEPEAEEGTTLPS

ADTLDDTTPMPVEPEAEVATTVTPTEDISPNEHMDHPQPPEEEATSAKPKEHVKPNPNQN

QPKEHKHQPKEEATSAKPKEHVKPKANKRSMRDSQSSKGDMDKDKVASPILSEGVDALTG

LLSEEWEHPISAPTGEHHTGRGKRDGSGEQPAQPQDGQPAPAPVGPPAVMSHPPAPVGPP

AVGPPAAQFQPPPFQPSAVGPPGFGQPPFQPNAVGPPGFGQPPFQPNAVGPPGFGQPPFQ

PNAVGPPGLGQPPFQPNAVGPPGAGQPPSQPNAVGPPGAGQPPFQPNTVGPPGAGQPPFQ

PNAVGPPGFGQPPVGPPGLMQSAPPPGGANYPMMPPQGKSVNIYL

>GMOY002421-PA hypothetical protein|protein_coding|scf7180000643634:45290-47080:1|gene:GMOY002421

MLRLSATLARSGIRHQLAVRGYAKDVKFGPEVRAMMLQGVDVLADAVAVTMGPKGRNVII

EQSWGSPKITKDGVTVAKSIELKDKFQNIGAKLVQDVANNTNEEAGDGTTTATVLARAIA

KEGFEKISKGANPVEIRRGVMMAVDTVKDHLKTMSRPVSTPEEIAQVATISANGDHNIGN

LISEAMKKVGRDGVITVKDGKTLSDELEVIEGMKFDRGYISPYFINSSKGAKVEFQDALV

LFSEKKISSVQSIIPALELANQQRKPLVIVAEDIDGEALSTLVVNRLKIGLQVAAVKAPG

FGDNRKSTLTDMAIATGGIVFGDDANLVKLEDVNINDLGKIGEVVITKDDTLLLKGKGKK

EDVQRRVEQIKDQIAETTSDYEKEKLQERLARLAAGVALLRVGGSSEVEVNEKKDRVNDA

LNATRAAVEEGIVPGGGTALLRCITKLDTLKGQNEDQNMGIEIVRRALRMPCMTIAKNAG

VDGAMVVAKIETKEGDYGYDALKGEYCNLIEKGIIDPTKVVRTAITDASGVASLLTTAEA

VVTELPKEESGPAGMGGMGGMGGMGGMGGMM

>GMOY002434-PA hypothetical protein|protein_coding|scf7180000643697:21736-22413:1|gene:GMOY002434

MKNYYIICLGLILLIQYCVASPIPDDEQIDEHNKLYIEVLKDLTEFALKTGDELREFVTK

VTDEIEQNNDKYFPNHRQEKLVKNYEKVKNSESNPNIMDLYELTGDIIDFATADFAAKDE

EAKKFVEKYKLVEFSEKIRGEVTKFYDHISEEFETYAHELDETQKKEQQKLFDWHKDFTG

TNDIKDKFNEIVSFFELFKPTLVNE

>GMOY002442-PA Serpin 1|protein_coding|scf7180000643732:31516-32946:-1|gene:GMOY002442

MSVNEYFKRQWKNPFVLLLLSGFALFAKNGTYIYTEASGICPCYEQFADDFFTEQSQRNN

DTNFVFSPFAMQSFEDLVKLGFSDKPELPDDNFSEIMEGLKDSEAVKLEYKIHLPEGREL

APSTLELLQEKFPSTFEHIDSSNAWMMKAVEQHLRNLKELEKFMGNKQAPSEALVEINTE

VNTDWLHPFDEQATKLAEFHTDDNRKVDMPFMHQVSNLRTAEMTDLQAKVIEMPFKNENV

SMWLYLPDEEDGLEELEEKLKLKSLNSIEALMSEREVDVLLPKFEDTHEMDLDGEISRSG

LDYPLSQTDEDVTIQRPASKLQTKLAMYESVEAHQAADNVGEDHVVAKRHAINLHSVDAD

TEVNESEEEATKDADAEVDVSCATDDCLKFYAAHPFIYQVVYKHVDEVVPIVMGCFRNLN

LNINI

>GMOY002487-PA hypothetical protein|protein_coding|scf7180000643833:93494-94919:1|gene:GMOY002487

MREIVHLQAGQCGNQIGGKFWEVISDEHCIDCTGSYYGDSDLQLERINVYYNEATGGKYV

PRAVLIDLEPGTMDSVRSSSFGQVFRPDNFVFGQSGAGNNWAKGHYTEGAELVDSVLDVV

RKEAEGCDCLQGFQITHSLGGGTGSGMGTLLLSKIREEYPDRIISSFSVVPSPKVSDTVV

EPYNATLSVYQLMENTDETYCIDNEALYDICFRTLKLTTPTYGDLNHLVSATMSGVTTCL

RFPGQLNADLRKLAVNMVPFPRLHFFIPGFAPLTSRGSQQYRALTVPELTQQMFDAKNMM

AACDPRHGRYLTVAAIFRGRMSMKEVDEQMLNIQNKNSSFFVEWIPSNCKTAVCDIPPRG

LKMSATFIGNSTAIQELFKRVSEQFTAMFRRKAFLHWYTGEGMDEMEFTESESSMNDLVS

EYQQYQDATAEEEGEFDEDEEAHE

>GMOY002513-PA aldehyde dehydrogenase|protein_coding|scf7180000643912:14481-28115:-1|gene:GMOY002513

GTCITEIERSGVEEVDLAVQAAKCAFRFGSPWRRLDASRRGALLNRLANLMERDRVYLAS

LETFDNGKPYFMSYTVDLPMSIKCLRYFAGWADKNHGKTIPMDGQFFAYTRYEPVGVCAQ

IIPWNFPLVMASWKLGPALATGNTVVLKPSEQTCLSALYLAQLIEEAGFPSGVVNVLPGF

GDVGEALVNHCDVQKVAFTGSTEVGKLIQQSSANANLKRVSLELGGKNPVIVFADTDMDY

AVETAHIGTFFNMGQNCCAASRIFVEEKIYDEFVERSVERATKRCLGDPFDLKVDHGPQI

DEKQMKKILDLIESGKKDGAKLLTGGARQEGMSGYFVQPTVFADVKDDMKIARDEIFGPV

QQIMRFKKLDEVITRANDTHYGLGAGIFTRDIDKVNYIVQGLQAGTVWVNTYNNFGSQVA

FGGFKMSGHGRENGEEALHKLKKMLRFIKSINVVQNTNKILASSLQQQRTANYSAMPEPQ

TTPDILYTGLFINNEWHKSATGKTFPSINPTTEKAIAEVQRAGKEDVDLAVQAARDAFRS

TCSHKKFLFINTFRLGSRWRRMDASDRGDLIRRLADLIERDRVYLASLETLDNGKPYSMS

YNVDLPMSIKNFRYFAGWADKNHGKTIPMDGDFFAYTRHEPVGVCGQIIPWNFPILMMAW

KLGPALAAGNTVVLKPAEQTPLTALYIAQLIKEAGFPEGVVNVLPGYGDAGAALANHFNV

DKVAFTGSTEVGKLIQQASGNTNLKRVTLELGGKSPNIVLADTDMDYAVETSHFGLFFNM

GQCCCAGSRTFVEEKIYNEFVERSAERARKRRFGNPFDLNVEQGPQVDQEQLQKILQLID

SGKQQGAKLIAGGARPEEMPGYFVQPTVFADVQDDMRIAREEIFGPVQQIIRFKKLDEVI

ERANNTDYGLAAAVFTKDIDKANYIVQGLRAGTVWVNTYNSLAAQVPFGGFKMSGHGREN

GEYALRNYTEVKSVIVKLAQKNS

>GMOY002531-PA hypothetical protein|protein_coding|scf7180000643919:113081-126728:-1|gene:GMOY002531

MSGQRCRSLDRAQSKPWSPVNAEYIPFSYQIREVQSNEYLSQSLEALRDDDGDQRIEANT

DVYPAPLEYLEQTLPPDFQDIPANASFEELVCIYASPPPPTPQIEPQIARPNLEQLEASF

RAEEVTRPPSPKKAPCPMALSEDPYYIPPPPKDKTDIQGMIKRARAIERLRKIKEEEAAK

ANARSRKQLGQPPRASAPVSRARGAPSKPGGQLPRAGRPPPPGAGRVPPRVSAPAQRVRG

PPAKPAAKVSASTSRTGVQQAKPSDAAYKTRDQSRKISAPPSKTRGPPPKAAPPKPGGPP

RAGAPASRVGSLPPRTRGPPPKAAPIKPGVLRRASSQPSRPPPPKIRGPPVKVAPGGPRR

PSAPASRGGPPLPKTHGAPPKQAPSRAEVPLARRRPGPETGAIPKRLAPAKQTADRRPGE

KKKGERPKTKPQDARYVEALPPVKLPTPTKPKTPSPPPGPTYESIVHRISAIRAKKLPPQ

ECVGEMADVARDLDILEEVTAEEEAAAEEDAVAMQAACEKMWLSPKGRPWKTRPKFRRDL

LDVSPDLIQEYIEPAEETIDEFLALEEAVYGGEKVVAPEYMTQLEMPYMPELIEQIRQLG

MYDMPATPPQECPGSPPAQMEGEIMEAIGVEELAMMEALAPDQGLSAEAASESPVYEMHP

VLKYADYKFRQEQMAEAAAAAAMGAAATDVESEDEECPLPALAEELQKLQAAADWELAQM

KTPPRVPQLQSPEIEYLDDAFFQGIACPAFDIEDVSEFAFSDEAAVGAGFGEYEIKELDG

EEWLVPKESGEAADVAEGSECPEELEEPLWEAAAPDVEFGTSYIPSEMAEAGFDEGLQIA

DVSVPSYYEENVSIDEEGLEDVTSPEIVDVSMCPVQTTDEEEQVCEPIEETPKRSFLASV

VETVVDKVKNIFGKSPRIEETEEEDQPQCISLDSSLPNMFGLSEALEPETVEESETVEGP

VCPAPEQVQVQPTKPKSPPKPKKETPIKIPPPPKDPKDRKAFVARARAIQKLKERQLAAA

GALPPPPKPKKKGRMPISRWVDKPKPQMKRKKVLMRKKGYKTPPPPVEGEYDEPPPFPPG

YCEAVARARALQQLRRKRPDLSVKEADKIIAAQRARREAAEAEAARFAALPEHEKIKERM

ERVRKAPRRRRRKVPLDVIAAISAFKESAAAQPCPPSPRAVSPTQGRTSQEPQDEEFIEF

SPLRAQERMRRLQYAREQAAARQLALVCPEMEPEAPVEVSPVRLESPKTEEIRRERITKQ

VGTYGGEECTKQTVIETSTVQNGEVHSTSTKITTKIDEFGNTTCHVEEGTCSTDKENIME

DLEAIEEPDFLDPSNIPAPLDTSMPTPSELIELIRSKVQEKLNIAISPAAEPVEVSRISP

VRLQDQMQIFEGLPEEELLDITSPDIELSPTGPEPPATREPLLDTSLPKIFIEPQPTPPR

RTPSPVRARSSPRAPVQAPPPQPEVFPESQVPSPIVIPPPPKDPKDYRAFVARAVAIQKL

KEREIAEAARAPPPKPKKKIRVPISKWVDKPKPIAKRKKIVMRKKGYKTPPPPVEGEYDE

PPPFPPGHCEAVARARALQQLRRKRPGLSLKEADKIIAAQRAQREAAEAEAARFAALPEY

EKIKERMERVRKAPRRRRRKVPLDVIAAISAYKELMPSEPCPPAAAPLQRAASPQREVSD

TRKSPVQVPLPERPEETPRARPTVARQKARGRIILPSEERRQREAQETYDMQKRMQKLEE

ELEAPPKWRPASRHRVSRRKPPGESPFRKKFPRVFAIARKKRSRPLDVLDVLREEEERED

SFEGEDDDDEFEILERQIRAALELGVGEEDVSIEASLEEEEQAAGMVKPSTPSFEPRYSS

YPEWLVKHYQEEVEEFACPSEDVEASAMLMLDEATGPIDRFRRIREMHVSASTTTSETSV

EPSISVDVSYSAITLTSPTQQPAQASLAEVVEDQPVDQLLVDIEAPPVHVVRQLADLEDL

PFDDLEYEDVPDMPDVPLDVPVTPPPRPEAIIQRIRAQVEQMLTEQAVQRSPVTTPPMRP

IPEDLPEEDLLDVSAVSEEEIPTTSPLVRQDILDISLTTIFEEPRPPTPPQRVPSPARAR

SPAPALPAPPEVCPPSQVPSQPQRPSPIVIPPPPKDPKDYRAFVARAVAIQKLKEREMAE

AARAPSPKPKKKARVPLSRWVDKPKPKTKRKRIVMRKKGYKTPPPPVEGEYDEPPPFPPG

YLEQIARARELRELRKKRPNLTLRELDQIIAARKANREAAEAVKRAKREAAEAEAARFAA

LPEYEKIHERMSRLRTKVGSRRKRRKPLRISPSILGVLEAIKASMPSSRHPSPQREGVVV

TRIEAAAEYQEDEEGAIGKQSEISLHEFLAVEGAAHQSRVPELPGYQQQIDILSMPEVVE

QLRELGLQDQGPPLPPEGGQIDLPGVCPPTSEVQLGAHERYRRIVERRNVPMTPEAPPSP

VEQPVIEYSPIRISTPTPTKAQKQTTQITKLGDNKIVEQVIETSQIRHGELQQVVTQVTT

TSDLYGNVTCQINQNKEISAQDPTASEDVFAIEEPDFADVSMGQIEERPRPAEIIEKIRK

EVREGLRPEGRPEAPTGRLVEVETPPRSLPIPEDIPEEKLVELICPPLAQTPPSSSPKTP

PRQVSPVRSLDVSLAEIFAPTPPPPTPPLPPTPDYRRRLSSPPGPIVIPPPPKDPTDYKA

ICRRARKIMELKAQEEAKAKAEAEAAAAAAAAAAAAAAEEAKKPKKRVPISRWTAPPQAT

RPKKELKRKRMIIPKKGAAKLPPPAKVLPREAAKRKELEDMAKRQEAEEAAAAAEAARIA

ALPPPPPLPLSYTTIMTRLQGLRERHGGEVQPVPVTAETKAYLHALRNWLPLPCPPAEGQ

VLPPPRLTPTAADRYRVLRARTPGLLPKSSSGLPPSARYKQILERREQEPATPQPCPASP

VQTQEVEVHYRAIPFEPLPAERPEFAIEIEEEGELEEVEEPSLLQEVCEIIHGSPQTPVQ

SPSKIISRIREQVRTQRATAAAAAEARPRTSPPQIDFPANLPDEDLLGYETPMFEITPPR

TTSPIRSPDISLPRLFETTPSRARTPSPQRSPVVMPQPEHQVAAICPQPAPICPPQKVPT

PPVVIPPPPKDPKDRAAFVRRAQAIQKLQQQQTAKRAAEHPKQPEPRGRVPISRRPGGPP

PVRRGVGKSRGPPPSKKVCPPSKKSDQAPKKGGPPPRKGGPPPRKAAPPPRKGGPPPRKG

ALPPRKGGPPSKKGEIPAKQDMPGPSRKAPGAAPPSKEYVDAVMRARKLAGIQQRSPTPP

PAKPVTPPRPPTPPRGSPTYEHLMARAAALTAGRPSRPSPEIKIPEVIGGREQTLAQYRI

NQQQREERLRHLRELQEQREQQGDIEIDFGEGTPPRPILERLEAIRERQSIGVATAAYSP

VQTPPTPELEYQFCPYIYESSPREYGEPIELIPMPELPEDYLTPCRQYESDACLAEVANI

NQEILAAHEKIIQAPKGRRVRRSIRERFGRSHIATPPASRRRLDFSPLKTPLSAESAALA

EYERMEGRPSAFPARATPEESQHYPIERQIQLLQRRLFSSPPEAAFQDPSQPISLLSPEA

AAQVPPQPISLLTPEAAAQVPAQIISLLTPKAADQVPSQPISLFTPEAAAQVPAQIISLL

TPKAADQVPSQPIPLLTPENMQRIEQNIESIKDQLAALPPQQPITTTLTPEEIQRYRMRL

EVERLQDQLASSPPPRRVSPGFTSEDIRRFRIEEELRGLEEQLNTPPLSPFSPRVQRQLF

PSPSEAAAQTPSELQQPQICPEEQQVRKSPLRQLSPEEEVIPEGRAAPFYKQIPRILSGV

RSFEIMETLVEVLTPEEQLPVYGPSLPPGGLPQLKPKQRAGQKTKPPSQQQRAKSLAEIS

KKPSKPQIQPRPATRLPKQPKTQTTSLPSMPAQAQAQARSRTGQVRPGARQQPQISTRPP

QQVPTRGQSQVARRPPQQVPTRAQPQVPTRPPQRVPTRAQSQLPKQPPQQVPARAQPQVS

RRPPQIPTRAQPQAPRRLPPQLPARPQPKATERPQAKVLTRTQTKPLACPKAQGSAQPQA

TRSVAQIASKPTTRPAGQTQITKRVLRSAKPPQAMLKAQSGIPTGSRIAKPSKPSKILPP

KAHSAVAAVQSSAQICPKIDYLRVPSRPKARELPKCPVEESPYKSIPGLPKIVVSSPGTS

SFQAAKEQEEAEFQQMQHMAWQRITGQVEEAEKEDFVYDEGDLAEDDSFLLMEQMLRTRH

GIQEQEVGDAGSQEMEEFLGISQAAQERPQICPQMDYLQVPGRRKQPILPKIPIEASLLL

RSPSIMPHTSNIPQIVVSTPTMSDLQAQQAQEAIQRRSAWVEAVRVGRQTPEEEEDEFAA

ELENLDEFLGQTEQDQFAAELEDLEEFLEEPKQDEFADELENFEEFLPEPEQNEFADELQ

NLEEFLEQPEQDEFAAELGNLMVFLGEDQAERETELDDLYEDFEDLNISRMEDLGVSRIE

DFEEMCQ

>GMOY002550-PA deoxyribonuclease II|protein_coding|scf7180000643942:25679-34179:1|gene:GMOY002550

MNLEKKKGENVSKGNVVGGVLQDERFQHLVSDPRFKILTKVERKVKIDERFQEMFTNNKF

KVKCHVDKYGRKINKSSSEDLRRYYELDSDEDDEENFETKERLKEEQAILQEEKNDKIED

SVKDSEEIAVTLSDRLLDPNIDYARGEGDLLTDSSSDESESSKEEPILSMDNVRQEWEEL

NNDAETIQHSTHRLALCNMDWDRIRAIDLMMLFSSFLSPGGSILSVKIYPSEFGKARMAE

EDLHGPPELVNLNGKTNRDIQEKDEEDSESGEDLLQEQDSDAEEGDDYHIEKLRQYQLSR

LRYYYAIVECDSAATADKIYKECDGLEYESSATLIDLRFVPDDTNFDEDEPTDVCYELPD

ISNYKPRQFTTTALQQAKVDLTWDETAIDRKELGEKLSSGKLDDISEKELRKIVACSSED

EDKDNYEREPETQHKESEKPDRKLDKKEEIINKYKALLAEITEKELQEKEGKKYDMEFTW

QVSKDDEVDNKELDLSETDKKDLTPIEKIMQKRAAKNKKRKEERKKKKLEFKYNGEGIDN

SDAGSDSIPEDIDMNDPYFAEEFANGDFIEPVGKVREKKKKRKNLKSEDSEEDEKQLKEL

ELLLDDDEADEKYKQHFSLAKIIKTEQEMKSKKKRHKYAKKSKDSALKDNLVEDTFEMNV

NDERFQAVFNSHEFNIDPTNPHFKKTNGMEKLISEKMKRKCIDDDVQKPATTNKKQIENV

LLFSLVARKMHLFYFHICLLVVLSLNFIECALNRNLKVACKTEQGYYLYKLPKHYTSGNK

RNSEPKGYDYMFATSDAHENWQFSEQKIKMTTSMPGKTMVDLFDDPSVLLIAYNDEFPDG

TVKSGGAHAKGVLATDGVTGFWLIHSVPKFPSIPHYLYPATAAIYGQSFLCITFSASEVE

KIATQLLVNAPNIYFDRVPPKLVGKFPLLELVIDKEWNRTEPYYNILDINSLENVHFKSF

AKSSKYNQELYEDLMAPELASNLLVESWRNGGGNIASNCSLGQKVYNVKEIKGNQRSLAF

RSSQDHSKWAVSQSTGFKVFHWRIGGTSPNWICVGDINRQQGQLNRGGGQLCQKNKIVSG

LYSSLIKDYESC

>GMOY002563-PA hypothetical protein|protein_coding|scf7180000643976:1728-2972:-1|gene:GMOY002563

MVRVLFLHPDLGIGGAERLVVDSAIALKEYGHQVGFLTNHHDKNHCFEETADGSLRVEVV

GDWLPRKLFGRFYAFCAYFRMIYAAVYATFCIIPKERVDVIFCDQVSVAIPVLRFARPRP

KIIFYCHFPDQLLSKKEGYLKSLYRWPINIVEELTINMADKILVNSKFTLKVFQETFRRI

HHCPDVLYPSLNTKYFDEMEKKHNLETPTYFRNLADCYVYLDINRFERKKNHQLAIESFH

ELSSSLSDNKPRLIITGGYDERVNENVEHFNELQDLIKETDKENVILLRSSSDDEKFQLL

RRAQCLLYTPIDEHFGIVPLEGMYMSKPIIAINSGGPTETIVHNETGFLCEPNKASFVEA

MKAVLGNKALCERMGAKGRKRVQQKFSFEAFCKKLDGIVRDIAECDNRTTKKTE

>GMOY002825-PA odorant binding protein 2|protein_coding|scf7180000644614:60099-60640:-1|gene:GMOY002825

MKTIIVIVFLVTLATVWGHHHHEHHDDDDYVVKTREDLFKYRDECSNKLNVPADLLEKYK

KWQYPDDEVTKCYMKCMFEHFGFFNEKQGFDVHKIHKQLMGAHGTVDHSDETHEKIAKCA

DKKPEDTDPCAWAYRGGVCFINSNLQLVKSSVN

>GMOY002884-PA hypothetical protein|protein_coding|scf7180000644733:67286-75667:1|gene:GMOY002884

MHKINLLAKNIVKSLSNIGIVYPGLIPRRGVRNIRTEIPQITMNNGCKMPMIGLGTWGAT

DDFAQIESLGLGEKREKGIEPNEMVEITKKAIDCGYRHFDTALMYGNEKQIGDAIQQKIK

DGTVTRNELFISSKLWHTYANPNRVILGCKRSLKNLGLDYVDLYLIHSPLAAEANDNTLY

PKTKDGKPAYAKVHYVCTWRAMEDLQKEGLCKSIGISNFNLKQVKHILEYGTIVPQNLQI

EHHPYLTQTALIGFCKAHNITITAYAPLGSPNRPWAFKDTPKALLSDRKASEHVLAIAKK

HDKSPAQILIRYQIEMHFATVPNPGRSEKFLIENINVFDFCLRPEDMAVLNSLNANVRYF

KFTGLAFRKTKQKIYIRNFLTISYQFSLFTAPLRLACIHTGSILSAKMHDTRIKIPDIVL

NNELRMPVLGIGTWGIKPSEMVNIIKNAIDHGYRHIDTASLHKNEKQIGEAIKKKINEHS

VKRDELFIVTKLWNTCHDPQNVKCACRKSLCNLRLDYIDLYLMHTPMGVPAPHDENEFAP

TKNGKPIFTNVDYVCTWQAMENLVKEGFCKSIGISNFNIKQIKRLLENCTIVPQVLQIEH

HPYLRQKELTDFCEAEKIAITAYAPLGSPNRPWVGKDTSDVLMCDPQASKCLLLTIFTHK

KSPAQVLIRYQIDLGHATIPNPGRSEDNMIENISVFNFKLRPQDMITLANLDSDIRYFNF

TGVRKTTAKMPNIVMNNGHKMLALALGTCWCNISSMDPGGRRRMIGKATGYDYRHIDTPF

VIVKPLNTAIDVNTYLACRRGFSAQDELSPVPCITMNNGCKMPALGLGTWGANDGYLEKV

GKGKGKNGPVQSDEWVNIVKRAIDCGYRHIDTALLYRNEKQIGQAIKEKIKEKVIKRKDI

FLVTKLWNTFHDPENVMCGCKKSLENLGLDYVDMYLMHTPMGAPFQKDDKISSPTKNGKP

LFTDTDYVCTWQAMEELVKKGLCKNIGVCNFNIKQLERLLKYAKIAPQTLQMEHHPYLTQ

KELKGFCRALKIIITCYSPLGSPNRPWAGKEKILLKDPQASEYLNCND

>GMOY002885-PA hypothetical protein|protein_coding|scf7180000644733:75669-76030:1|gene:GMOY002885

MFRFIAHTQVIKIANNYEKTPAQILLKYPIDLGHAAITNPGKGEKNMADNICIFDFCLTP

KDIEILDALNANLRYFKFTGNSGHPHHPFETPKS

>GMOY002966-PA putative dynamitin|protein_coding|scf7180000644939:227696-260522:1|gene:GMOY002966

MFIEIHVTYGRSNNVLTYRKLAIRLNPYRDPKHCKEFVPLAEENQLTHMSPLGLSKQWAY

INMALDVLGNDLYRSIYDRYGEAGLIQGVLLPNGYFPPYNYHGDHMKVYHEVFGTYSPYA

NIMDPITHPPPLYTNKNNGIGVRTKEPPVEKTVALSLEDVYFGCVKLMHVWRQEFIDKEE

LTTEKRKKTLTLNIPPGITAGTRFSFKEEGDRFPTKIPSDIIFIVEDKPHDVFKRRNLHN

IVYTHEINLCQALTGFRFVIETLDKRKIKVSISDVVTPDYVKILPNEGLPKYKSVDDISK

SRGVPSYEYGDLIIEFKTPRKQLATKAARKSAPATGGVKKPHRYRPGTVALREIRRYQKS

TELLIRKLPFQRLVREIAQDFKTDLRFQSSAVMALQEASEAYLVGLFEDTNLCAIHAKRV

TIMPKDIQLARRGKAKGKAKSRSNRAGLQFPVGRIHRLLRKGNYAERVGAGAPVYLAAVM

EYLAAEVLELAGNAARDNKKTRIIPRHLQLAIRNDEELNKLLSGVTIAQGGVLPNIQATY

DQPDVYETPDNPEQETSDYYEEEPENEAIERLHISTQNSYQRFRHARLEGPVDFSERINK

RACRGYGAAWGELEIAGVGEKETPVQKCRRLQWEMNELMDEVSALQNDRKLKEEEKASYN

AVASVIATAKKVLDSLRLEQVLGKEQTPDMADKEVKALINQVDEYKKSGILTAIEPAGND

LAATARVAKLEYRLHQLEQAVGAQPEKLSRLASLTQHTNIVDAVRQLSTKSAMLQAEKVE

CIESRLGSLITKMDAIAEKSSGSSEDAKRDQKIIELYNIAKRTEPVADVLADIIERMQAL

EALHKYAMNFAKIIFEIEEKQTTISTTLVNNRELLHSVEETFAENLNTINKEMAKVEERV

NGLVDKV

>GMOY002979-PA hypothetical protein|protein_coding|scf7180000644968:39688-56665:1|gene:GMOY002979

MCKQVWRSTIFLVLYGLSHIRACDLPPEIEKCKAGDHICVTQRINDVIRLYPEGNPLFKM

PNLKLITVDRLAGTKTGSNTQLQLKFQLFDVKITGWDKATALSAKGFEKDTQYHEIELQV

PIMKIVGRYEIDGKLLLLPIKGSGNEEFIFKDVHITVTHKFNLEKRDGKNYIKTVSFLTS

IEPGSVTFKFENLFGGNKELTDATNKVLSDNSLDVWNAMSGQFNNALGQLYRKLLSPGTE

CLSYDDFFATEIVEEDNTNIRGIQRPIPQIILREWQQRWNDGTKAPEIQKCKAGDDTCIA

ERINEIIRLYPKGNPTFGLPDFSLVKLDKATASRKSSNSPVKLNFILSNLEARGIEKLKV

FKTKGFVKDVKNTEIDFTVPLLRINALYELDGKLLLLPIKGKGNVEIIFKDAHYFENLFG

GNKELTDATNKVLTDSWQDVWNELGRDVNQAFSDALHFNLIPVADAISYDDYFAD

>GMOY003011-PA hypothetical protein|protein_coding|scf7180000645045:17658-21933:1|gene:GMOY003011

MAMQYLEYTMTNRLKIVWNLDPPMRDIEIGIELSRGVYSWSYEKFLARRRFKHVWIDLLM

WEFGEALLERVKLHFDLNEVSRLVDVIFKAMYALVDGKSIDFDKYLWGPFIDVQRYLTVA

EDLCAEVHANKTKEFEYVNFLMVAWGPAVLPVINETNERLFDAIEIRYQLNWYRSKVNQM

QLKSDILQESYVKSTSQALQASKTEEDVGFNIVDVIYNETQRFRDLIIHFQKLYDVELEK

IETSIRVNKKQITKLVDHQKFLSEQIVRFAEEIAARKALEIKRFERATVVYQLVEMARIR

RDDTPAEGDSAKIDFSEFANKIMDEFKKTLNNEDLQVFVA

>GMOY003054-PA tissue inhibitor of metalloproteases|protein_coding|scf7180000645221:11192-12117:1|gene:GMOY003054

METRRNCLGIFAFIWFAVITFYCKSATACSCMPSHPQTLYCEADYAVVVRVLRMSYRSFE

NNMIYKIQIKKSFKTTPEGDMILRHHRLLTPSHDATCGVKLGIGKLYVIAGRGRHLNSCS

YIQEYQKMTVVERMGFSRLYRKGCDKCKIKACFHKYCSPNDTDKTVCQWSPFDECEKNFS

ACLKRTSYKERVDRDQYGRCYWKKSPVYNKCKAEQT

>GMOY003176-PA hypothetical protein|protein_coding|scf7180000645539:55852-74333:-1|gene:GMOY003176

MFKSHLEMIGRNETPSKKAKFWTSYIRSLKGSEDIRAHETPRLGRPYSSYLDAPATYRSI

FDEPVTAQDRVLSHGYRYLPVSRDTYGYSPRAIYDHHYSRTIPATYEAERAWNEHLRRMQ

EIERRYPSRYGLYLKDKPYTPHSLVPLEFEPEDKMLHDLNRARRSPSPMVGPNRVKRAGS

EPYVPPPTCWSRGPSPPRGRSMTRGPSVFDRATSLAPFTRPLFRASSLEPLDELFARKLE

RPSVFERAGSPSPTPVKAGRWGPRPTEVAFDADGLPIFHPRNRFRDMFDPNPAMPISAFT

RDPFWWDIEDLAPFRAGSPFRGLSLPPRGSSVARDSFLSPVKNRYLWAKHPARPLTRKY

>GMOY003206-PA hypothetical protein|protein_coding|scf7180000645622:4233-5241:-1|gene:GMOY003206

MKVFILMSCLAMAAARPEAGYAYNRPGGSGGHGGGGFGGGSGGFGGGGFGGSGGFGGGSG

GFGGGSGGFGGGGSGGFGGGGFGGGTGFGGGSGGGFGGGGAGGGGFGGGAVGGGFGGGGG

TLVQKHIYVHVPPPEQEDIRPRPNIPVGQAQKHYKIIFIKAPSPPSYQTPVIPVQPQNEE

KTLVYVLVKKPEEQQDIVIPTPAPTQPSKPEVYFIKYKTQKDGGGAGGIGGGDLGGGSGG

GLGGGLGGGLGGGLGGGLGGGGGGGLDGGFGGGAGGLGGGSGGGFGGGSGGIGGGSGGGG

GGLSTSYGPPGKSGPY

>GMOY003216-PA hypothetical protein|protein_coding|scf7180000645625:136161-138241:1|gene:GMOY003216

MRLLLILAITAFVGFSYGEEKKEKEKDIGTVIGIDLGTTYSCVGVYKNGRVEIIANDQGN

RITPSYVAFTADGERLIGDAAKNQLTTNPENTVFDAKRLIGREWSDINVQHDIKFFPFKV

IEKNSKPHINVATSQGNKVFAPEEISAMVLGKMKETAEAYLGKKVTHAVVTVPAYFNDAQ

RQATKDAGVIAGLNVMRIINEPTAAAIAYGLDKKEGEKNVLVFDLGGGTFDVSLLTIDNG

VFEVVATNGDTHLGGEDFDQRVMDHFIKLYKKKKGKDIRKDNRAVQKLRREVEKAKRALS

SAHQVRLEIESFFEGEDFSETLTRAKFEELNMDLFRSTLKPVQKVLEDADMNKKDVHEIV

LVGGSTRIPKVQQLVKEFFGGKEPSRGINPDEAVAYGAAVQAGVLSGEQDTDAIVLLDVN

PLTLGIETVGGVMTKLIPRNTVIPTKKSQIFSTASDNQHTVTIQVYEGERPMTKDNHLLG

KFDLTGIPPAPRGIPQIEVSFEIDANGILQVSAEDKGTGNKEKIVITNDQNRLTPEDIER

MIHDAEKFADEDKKLKEKVESRNELESYAYSLKNQIGDKDKLGAKLSEDDKTKMEAAIED

TIKWLDQNSDADPEEFKKQKKELETIVQPIIAKLYQGAGGVPPTDGGEESDDLKDEL

>GMOY003226-PA hypothetical protein|protein_coding|scf7180000645661:179073-184752:1|gene:GMOY003226

MNKNIVACFSLLILCLMHGANAIKCYQCKSLTDPNCGKNVIDVNSNIRQVDCDYAAKPNS

MEQFMPVTKCNKVVTSDKAGIIISRDCHFQIVGQRDDICSVSHSRDVQSCYTCTGDLCNA

SSAGRLMAYGILAIFSLVAINIAF

>GMOY003229-PA hypothetical protein|protein_coding|scf7180000645661:189044-189852:-1|gene:GMOY003229

MADSEKVKDEDAAEPVAADDTIVAIPPEEPKVEEPPAPVIEIDDADSPKPPTPPKADEID

DAGKAKATTPPKADEINDVVKSKAPKPAKVDEGPKVDKAMSDEINFLSEQFENYSRFAKV

LMKTLKRPKDVESCNELLRKVERLNDSRQVEVKRNVNRFMRYCLKIIHWTSQNQPTKIYE

AWYCRADEKAKAGKLSETSTWIGDRKSYIAVKSLPDGATLLYAAVTNNPESGWEEGGLKL

LEQRGACKRA

>GMOY003287-PA hypothetical protein|protein_coding|scf7180000645803:105949-114126:-1|gene:GMOY003287

MRNRHLKQSECPMSNSAERNIRRHFISLKMPKSKKEESSESDSDSGPEDRLPPPSKKSKE

SNANSKSKGSTETKWILEKQRHVTISEFRGRKLIDIREYYEENGESLPGKKGISLSITRW

KKLLEVAEEINKSLREDDQSDIAHEGPAGMEPEGVIESTWHEVYDNFDDMNLREELLRGI

YGYGFEKPSAIQQRAIIPCVKGRDVIAQAQSGTGKTATFSIAILQQIDTSIRDCQALILA

PTRELATQIQRVVMALGEYMKVHSHACIGGTNVREDARNLEAGCHVVVGTPGRVYDMINR

KVLRTSRIRLFVLDEADEMLSRGFKDQIQDVFKMLPNDVQVILLSATMPPDVLEVSRCFM

RNPVSILVKKEELTLEGIKQFYINVKQENWKLGTLCDLYDTLSITQSVIFCNTRRKVDQL

TEEMTTHNFTVSAMHGDMEQRDRELIMKQFRSGSSRVLITTDLLARGIDVQQVSLVINYD

LPSNRENYIHRIGRGGRFGRKGVAINFITDEDRRILKDIEQFYHTTIEEMPANIADLI

>GMOY003314-PA yellow-e|protein_coding|scf7180000645812:30002-47300:-1|gene:GMOY003314

MLSAQLTRAGMEARSRMLKFFVWHKMSALHTFVTIKHGSSCRGASQNSFISGLIALFSLL

NLSTATFQQSQYSSGFQIAKQWKTFTYNFLPQAPVHDTNFFNPSNILGTGLAVTRDRIFL

STPKLFSGVPSTVSWMSRKDFGDSPVLQAYPDWSFSTTGRTDFNCSDMVLVSSYRLRIDS

CNRLWILDAGISRSLEDYEKTCPPKILVVDLSSDQVVRRVDFPSGILRGETLFTNLVIDE

TTSKSGTCDDVHVYISDTVEPGIIVYDSEHDTTWRLSHPAMYPDPDFSQAEILGDRFVLM

DGIVGMTYDDKKAVLYFQPFATDRVFAITREVLRAGPIPINKVLPVKLLGKKSSQGIGLA

LSPLDRSLIFSPVSETAVASWNPATNEQHILAQDQQAIQFVGDMFVSVDDPGYVYVLSSK

FHRFFLKTLNTAEVNNRILRIPLPGSEKLDSALPSYPIKSLDTPDLNFYYQISNDFPHKS

SARITNISPVQNYFTRPTSVSKHPYRFESSGIVNYGVKNPFNNLNQGESFPPTKEQRSKS

SYSWLDDLGGPPTRYKNIPHYQQQFPEVLYQWNQLDYAFPNENVLQAALGDQTFVPGAGV

PVDVQPYYNQAGDLKVFLTTPRFGMGVPYTLSSIRDNSSIEAYPSYEWHNRGDGKWDCDK

ISSAFRTAITECEQLWVLDSGMAADEFKCPPQLLLFDLRNDELVHRFRFNDSLFTASASL

FVTNVVQVNDPPPAGNCQDAILYVADTDHHGLVVYDYGKDEAWRVENKFMYPDPDFGTHS

VAGQRFNLMDGIIGLSFDEQYLYFHPMASITEYAVPLQIINNSTLFAENREAAGDEFKPI

AKRSSPCVPSAIDSKGIWYCVTFNPIELLAWDIKKNMIEKLAIRREHLEFVGGLKVIKNK

DNQDELWMLSNRFQMLLCIVIAFQVLLVYGTTFTSTFSQQVDVLENEPVLEGIFHRPPYK

NLVRVYEACNLEFAFPSEEERLSALATGKYNPDVPVPIDVDVYFPNEFAEPYIFMTIPRF

GSGVPYSLALLTNVQRPNGTEVVAFPNYYWHNSHGQDCDGITSVYRIQIDSCNRMWVLDS

GEINFVQHCAPQIVVLDLPTGKLVHRYRLPADVFRISTSRFVTPFVDIADNPPFGECKQA

FVYMADALGYGLVVYDVQRDKSWRIENKYTLPDPDFGTLTLAGESFELTDGIFGLTATPH

GLGLRRMLFFHSLSNDAQIAVPLDVVNNDTLWHDGVASSIEEFILVGKRGVQCAASAMTS

HGFLLCGFLNPIAIVGWNIRTPYVSPNRIVWAENPHTLQFVAGLKVRRNPAGKEEVWMLS

NRLQKVFGGTINYHEINYRIQRCGVDELLFGSECKP

>GMOY003315-PA actin 87e|protein_coding|scf7180000645812:74006-75136:1|gene:GMOY003315

MCDDEVAALVVDNGSGMCKAGFAGDDAPRAVFPSIVGRPRHQGVMVGMGQKDSYVGDEAQ

SKRGILTLKYPIEHGIITNWDDMEKIWHHTFYNELRVAPEEHPVLLTEAPLNPKANREKM

TQIMFETFNTPAMYVAIQAVLSLYASGRTTGIVLDSGDGVSHTVPIYEGYALPHAILRLD

LAGRDLTDYLMKILTERGYSFTTTAEREIVRDIKEKLCYVALDFEQEMATAAASTSLEKS

YELPDGQVITIGNERFRCPESLFQPSFLGMESCGIHETVYNSIMKCDVDIRKDLYANIVM

SGGTTMYPGIADRMQKEITALAPSTIKIKIIAPPERKYSVWIGGSILASLSTFQQMWISK

QEYDESGPGIVHRKCF

>GMOY003481-PA mitochondrial processing peptidase beta subunit|protein_coding|scf7180000646258:347269-349066:1|gene:GMOY003481

MATRVLSVSQNLRPLSRGVDMLKRFKASKAEVMRKTLLNIPATQVTALDNCLRVASEDSG

ASTATVGLWIDAGSRSETPQNNGVAHFLEHMAFKTDLELEVENMGAHLNAYTSREQTVFY

AKCLSKDVSKAIEILADIIQNSKLGESEIERERSVILREMQEVESNLQEVVFDHLHATAY

QGTPLGQTILGPTKNIKSIGKNDLQAYISTHYKASRIVLSGAGGVKHNELVTMAQQHLGK

LENTFDGKPPSVAPCRFTGSEVRVRDDSLPLAHVAIAVEGCGWTDQDNIPLMVANTLIGA

WDRSQGGGVNNASNLARASAEDNLCHSFQSFNTCYKDTGLWGIYYVCDPLECENMLFNVQ

TEWMRLCTMVTEAEVERAKNLLKTNMLLQLDGTTPICEDIGRQMLCYGRRIPLHELEQRI

EAVDVKNIRDVAMKYIYDRCPAVAAVGPVENLPDYNRIRSSMYWLRV

>GMOY003513-PA hypothetical protein|protein_coding|scf7180000646337:39232-40644:1|gene:GMOY003513

MSIYRISTTRKMPEVQKLSALLASYVRFISADSSLRDVLAEKIPAEQEKVKNFRKQYGSF

KTGETTVDMIYGGMRGIKALVTETSVLDADEGIRFRGLSIPECQKVLPAADGGEEPLPEG

LFWLLLTGEVPSKAQVKQVSREWAARAALPQHVVTMLKNFPTSLHPMSQFSAAITALNHD

SKFAKAYSDGVHKSKYWEHVYEDSMDLIAKLPVIAATIYRNTYRNGKGSKSIDSSLDWSA

NFVKMVGYDDPKFTELMRLYLTIHSDHEGGNVSAHTVHLVGSALSDPYLSFAAGMNGLAG

PLHGLANQEVLVWLLKLQKEAGSNPSEEQLKEYIWKTLKSGQVVPGYGHAVLRKTDPRYT

CQREFALKQLPNDELFQLVSKIYKVVPPILQETGKVKNPWPNVDAHSGVLLQHYGMKEMN

YYTVLFGVSRALGVLASLVWDRALCLPIEWPKSLSTDVLMKMVQKETRFE

>GMOY003541-PA hypothetical protein|protein_coding|scf7180000646489:3473-3919:-1|gene:GMOY003541

MFRLAANQIANVMRSSVSSASRVSAVRALHGHDIPAEEFDKRYEDYFNRSDIDGWEVRKG

MNDLLGMDLVPAPKIVEAGLKACRRVNDIALAIRWLEGVKDKCGDKTDEIFPYIMDSVRP

TLAELGIPTLEELNYDKPELALKSVYDM

>GMOY003590-PA Scavenger Receptor Class A, Member 5|protein_coding|scf7180000646704:2284-21224:1|gene:GMOY003590

MEPRWKRLLFAAILAEVLIGSDAQFWKIADTGATSISERHYRPEYPQPLPNPIDDSYAVV

DSPNALPPKNCTSGPVGCIPKCFAEKGNRGPHGPIGVPGPKGHQGYPGIEGPPGDKGQKG

DPGPMGPRGLKGERGTSGIPGMPGVPGIQGVSGNPGAPGVPGKDGCDGEKGLPGIAGLGG

MTGPRGYPGVPGVKGEKGEPAKENGDYAKGEKGEPGFAGRSGNPGPDGPQGSKGDRGDTG

PYGVPGPRGDRGQKGDKGAPCFAPPQPGKKGQKGEKGEPARNAAPVHGTETISGEKGDRG

EKGDNGPPGEKGEVGYPGEPGRDGMKGEKGLPGSSGDRLAKPGLKGEPGRPGKAGERGLL

GPPGPPGGGRGSPGAPGPKGPRGYTGPPGPKGLDGFDGPPGPQGLPGAKGGPGVPGSNGV

EGPAGEKGERGNPGRQGSVGPIGPQGHTGPPGPEGQKGEPGLPGYGERGLKGDDGIPGIP

GLKGQKGERGFKGNVGAPGDAQLGRPGTPGRIGVPGQKGDPGRSGVPGQKGEMGPKGDIG

GRCSSCPPGFKGDKGERGLDGAPGSPGLRGPPGERGYPGERGSDGIAGSPGAPGDKGFDG

LPGLPGPEGPAGKDALLDLDLINVEKGEKGERGYKGALGPKGDRGEAGFPGIPGQKGEVG

EKGDKGYSGPGGTDGNPGSPGRDGRDGVPGQSIKGDAGKPGLDGEKGDKGFSGPQGSKGE

PGSCDIDNIKMPLKGNKGDRGDQGNPGPMGPMGEKGDIGRPGHKGEQGAVGPTGPVGPRG

LAGPRGEKGNIGPMGAPGNPGKDGLRGPPGRGGVPGQKGEQGIAVAGPPGPPGRIGMPGE

KGDRGPTGPYGPPGLDGAPGYPGDKGETGFPGTPGFSGRDGEKGDMGPLGPQGPPGPPGK

PGVDGVQGRDGAKGEQGSPGMVGMPGLKGERGAPGNDGPKGYPGITGHAGRRGPPGPAGI

PGVKGDKGERGLTGKDGLPGPRGPQGPTGLMGIKGDTGPVGPPGADGIPGLEGEKGEYGP

PGPEGLRGEPGDVSEKGQKGEPGPPGLRGESGRPGTPGLPGEKGVPGLALHGRPGAPGEK

GDRGRDGIDGREGTPGQKGDQGYPGPPGAKGDKGAIGLPGVPGHPGMDGKPGEIGLPGPM

GHTGIKGDKGERGFPGANGPKGDKGETGLPGFNGTPGAKGDRGLPGAAGLNAVPITIKGE

KGDMGEPGLIGMPGPAGEKGNQGTPGFSGPKGDRGLTGPHGTPGLNGAPGQKGEMGPMGD

IGVPGLTVKGEKGLPGRTGKNGREGAPGPAGQKGEKGLPGLPGNPGPEGQPGPIGPPGPK

GERGSMGSPGRDGGDGMPGATGVKGDMGYPGPKGEMGSPGLQGQKGEKGDYGLMGAPGMP

GSPGQKGDMGLPGLDGRPGPVGAPGEMGFTGPKGRDGRDGLSGQKGAKGEPGMVPPPGPK

GEPGYPGRNGQKGETGPPGPRGLVGLKGERGEKGEQGLIGLTGPMGRPGPKGDQGLPGPV

GRDGPPGLTGGKGDAGLPCSAAQDYLTGILLVKHSQSEEIPRCQPGHIELWNGYSMLYID

GNDYAHNQDLGSPGSCVRRFSTLPVLSCGPNNVCNYASRNDKSFWLTTSAPLPMMPVQVQ

DVSRYISRCTVCEAPSNVIAVHSQSIEIPNCPAGWEGLWIGYSFLMHTAVGNGGGGQALA

SPGSCLEDFRTTPFIECNGGKGQCHFYETMSSFWLVTLEDYQQFQRPAMQTIKAGELLTR

ISRCQRLVLYIHDRWINPKNVRLLKEAAESAKSAKLGPDEYQGCQVKRSRKRGYVLNSSS

SNALGGNEYCRFCANTPQGYCRHHFHLQELHMHKLQQQKEYNNSLWCSLGRSFERTADLR

QLRRIKRELKRNLQQQEQQKIQPIPLLSQYNNVEGHFLAQDEQNSPTDSGYQSSPLTSPS

SVTSTIQSEFISLLSSDDAGLISPSSFEDRQSAFGANSCGSASQSFVPYNHRTGLLHLLK

AQKQNYNMTSSESSTSSSRNSTPRHRQRSNARSANNSPTTSILKRNAMSRKALVPLEHGC

EDNWTWSRRHRSKEVVLSGPNDRTVHFHPNWSKGTAGVQGKRPLNNGRFYWELHVSQRIF

GTSIMFGIGTNKARLHANSFRNMLGENVHGWGLSHKGVLWHDGIALLYTKRFKENQATVI

GILFDGIEGTLTYYKDGKCLGVAFHGLDKIKEPLYPVVCSTAAKTEMTLKCARRDFVNLQ

DRCRAVIMKHIKTRQDLIQLKLPAIISNYLAEVLGDYTPLRQVDNYDLLFDF

>GMOY003684-PA hypothetical protein|protein_coding|scf7180000646915:29320-31609:-1|gene:GMOY003684

MLRNIPTMRYLPLSVGKESLLSTHLTGLYQYFQFIRNKSNMPIKSIHARQIFDSRGNPTV

EVDLSTELGLFRAAVPSGASTGVHEALELRDKEKNNYHGKSVLKAVCHVNEIIGPELIKA

NLEVTEQEKIDEFMIKLDGTENKSKYGANAILGISLAVCKAGAAKNGLPLYKHIANLASI

KDIILPVPAFNVINGGSHAGNKLAMQEFMILPTGASSFTEAMKMGSEVYHHLKKVINDKF

GLDATAVGDEGGFAPNIQSNKEALCLIKDAIELGGYTGKIEIGMDVAASEFFKDGQYDLD

FKNPSSDKSKWLSPEKLLGLYQEFINEFPIVSIEDAFDQDHWDAWSTITANTKIQIVGDD

LTVTNPKRIQTAVEKKACNCLLLKVNQIGTVTESIKAHLLARENGWGTMVSHRSGETEDS

FIADLVVGLSTGQIKTGAPCRSERLAKYNQILRIEEELGSTAKYAGKNFRSPLSPK

>GMOY003770-PA glutamine synthetase|protein_coding|scf7180000647149:3900-5102:-1|gene:GMOY003770

MALRCAGLFLGKELASSGKQVRAISTTAAVGQGNFLKNSPNAALDKTILDRYRKLETPVD

RYQATYLWIDGTGENVRLKDRVLDKIPSSAADLPNWQYDGSSTYQASGENSDTTLVPRAI

YKDPFKPGKNDIIVMCDTYKADGTATESNKRASLQAAIDKVTAHEPWFGIEQEYTLLDVD

GHPFGWPANGFPAPQGPYYCGVGADRVYARDLVEAHALACLYAGVDFAGTNAEVMPAQWE

YQVGPSIGMKAADDLWMSRYILQRIAEEFGVVVTFDPKPMEGPWNGAGAHTNFSTKAMRS

DGGMKPIEEAIQKLSKRHDRHIKAYDPKEGKDNERRLVGRLETSSIDKFSWGIANRGVSV

RVPRGVANAGKGYLEDRRPSSNCDPYAVCDALIRTCLLDE

>GMOY003774-PA hypothetical protein|protein_coding|scf7180000647199:83482-83931:1|gene:GMOY003774

MKTFLLLTIICAIFAFGFAAESTGFYKDPRHPGKCYINDNLVLSAGEQRRHTDYCARVIC

GGDGYTTIQTCGVQGAAPPCKLGDYENPDGDYPDCCKRNVEC

>GMOY003835-PA hypothetical protein|protein_coding|scf7180000647377:84978-85849:1|gene:GMOY003835

MENAEATVGQFQTLVRYNNPVLVVKHPDKKTAPTELELKRPQTAGALLDTKKETEEILNS

ILPPRCWEEDGQLWQQTVSSTPATRQDVINLQEMLDTRLQQTQARETGICPIRRELYLQC

FDEIIRQVTINCSERGLLLLRIRDEIAMSMEAYETLYCSSVAFGMRKALQAHEEKEMLRE

RVKILEVDKESLEDIINDMKIKTEQTDRRNAELRAAEEKKYQEEIAFLKKTNTQLKAQLE

GITAPKK

>GMOY003929-PA hypothetical protein|protein_coding|scf7180000647827:1210-44929:1|gene:GMOY003929

MVQNVLSESNRVNTNVWQTVNHRRTSKKELSYANASKKSLTYPPKENFALNKTSDRLCGQ

KTIRSTVINADTTPNKVATLQLPSSKKLLNNYNTNTIAALQKEHNLLNKKKKYRNNTISS

VSTANKIHNAGTSGSSNKSQIANGSAPLPRRKRLNQVRQIKRAHRHLLTSGGNDKQQPTL

QSQRAVNKINYHANSCSAKKTLQKNAHSSGRGRESGALSTSGVNKNKNNKGGNYNRCHEN

IQSSSQYHTYNRQTIKKKRVKSINQTSSESVTSSRWCSIEKQLDLLEKLLRCCDEEENIV

VQDWHRQLNANYWSNQATSSLQSENEEADLASVNDWSWSDIMSNQSISSSEECRNDYICT

TQLNMSLVPSTLKRRGHQHHPRFSGTRRSNVAPNVQEILDALNKREVTPFHVQSEVSETE

ASLSTLDDSYSAQSTDEPDDDPAAAQRIKAALNLPLTDSATTSLGSPTPDESSMVDEGVI

SAQMPQSILAMPKEKKLKIKRDKNASEQTKDKKCKKLSNSTKKDKQKRSSGCLETATEIS

NDTGSAATDEGIAIDDDDMQTAEWAKLRCTSEASEIVAEREARRSKNRCSDYPGLAFGRS

IFSSDTMMKFNIIRNELHNIMNTQLKRAESEVAALNRRIQLLEEDLERSEERLGSATAKL

SEASQAADESERARKILENRALADEERMDALENQLKEARFLAEEADKKYDEVQINKNLLT

PIKVTKITTTTATISSNVDAGCSSSTNKQNNSVINENNITSTATNTMPNSTYNLPIFSSG

IIANINEVARKLAMVEADLERAEERAEQGEKSPLSHFLSFVARKLVLMEQDLERSEEKVE

LSESKIVELEEELRVVGNNLKSLEVSEEKANQREEEYKNQIKTLNTRLKEAEARAEFAER

SVQKLQKEVDRLEDELILEKQRFAIIGDSLDLTFVELMGMPPVYNDRHPKPPTPQLPTPV

HEEEKVVATEQVIEEQEITDIIAQEEVMVGNNLQIDFIENVPMEAVKLPTPPPPPPFEYA

LDLPPEGAEVPYVKNFEKPTIPDEHLKKETILDLAFMDLIPGLEPFYTPRNPKPPTPKVP

TPTPEELAAMEAAKAEAEAAAAAAAEAAVAAAAAGAAGEAGAEGAPKVTAEGVEGATTEG

GEAAAAPAVKEPTPPPPPPPPFEYAIDLPPEGAEVPFVKNYEPPPPGAEPAAPADGAAPP

ADGAAPPAEGAAPPAEGAAPPAEGAAPPAEGAAPPPAEGATPAPTEGAAPAPAPAPVESS

TTAAAPAPTEKIIRGKEKLACKQMIVNVCDEYETVLNVASS

>GMOY003934-PA hypothetical protein|protein_coding|scf7180000647834:2837-7518:-1|gene:GMOY003934

MAGGRESYRIKDAEKKRVLDEAARQRRARKALEALEQDNFHEDPHADLVMSKKLPKFQDG

LKNTKEKKSKRKGPEYYRAKYRKNFQQLLEEEKQLHPDPPNYSSALAPPPKTPQRHFCAV

CVPFFQIRKAGYYGVEGQLRQFHASGYAASKVAISKYDPDVCMPYEKLWENLECIRSRIN

RPLTLSEKILYSHLDEPKTQQILRGTSYLRLRPDRVAMQDATAQMALLQFISSGLKRVAV

PSTVHCDHLIEAQIGGKNDLARAKDMNKEVYDFLESACAKYGLGFWKPGSGIIHQILLEN

YCFPGLLMIGTDSHTPNGGGLGGLCIGVGGADAVDVMADLPWELKCPKVIGVRLNGKISG

WTSPKDVILKVADILTVKGGTGAIVEYHGDGVDSISCTGMGTICNMGAEIGATTSVFPFN

ERMASYLKATGRKAIADEARKYQTRLLSPDEKCAYDKLIEIDLNKLEPHINGPFTPDLAH

PISKLKQNAEKNGYPMDIKVGLIGSCTNSSYEDMGRCASIANDAMQHGLKSKIPFNVTPG

SEQIRATIERDGLSQVFDKFGGTVLANACGPCIGQWDRKDVKKGEKNTIVTSYNRNFTGR

NDANPATHAFVTSPELVTALSIAGTLAFNPLKDELTGADGKKFKLKAPFGEELPAKGFDP

GVDTYTSPPADGSNLTVRVDPQSQRLQILEPFDKWDGKDLTDMTVLIKVKGKCTTDHISA

AGPWLKFRGHLDNISNNMFIGATNIENNEMNKIKNQRTGEWGAVPDVARDYKANGVKWVA

VGDDNYGEGSSREHAALEPRHLGGRAIIVKSFARIHETNLKKQGLLPLTFANPSDYDKIQ

PTSKISLLDLAGLAPGKPVDCEVKTDNKVEKIKLNHTLNEQQIAWFKAGSALNRMKEISG

>GMOY003968-PA ATP citrate synthase|protein_coding|scf7180000647850:122981-129402:-1|gene:GMOY003968

MTRSFLTGNWVCIKLNALLASYVRFISADSSLRDVLAAKIPAEQERVKNFRKQHGSFKMG

ETTVDMMYGGMRGIKALVTETSVLDADEGIRFRGLSIPECQKVLPAADGGEEPLPEGLFW

LLLTGEVPSKAQVKQVSREWAARAALPQHVVTMLNNFPTSLHPMSQFSAAITALNHDSKF

AKAYSDGVHKSKYWEHVYEDSMDLIAKLPVVAATIYCNTYRNGKGSKSIDSSLDWSANFV

KMLGYDDPKFTELMRLYLTIHSDHEGGNVSAHTVHLVGSALSDPYLSFAAGMNGLAGPLH

GLANQEVLVWLRKLQKEAGSNPSEEQLKEYIWKTLKSGQVVPGYGHAVLRKTDPRYTCQR

EFALKHLPNDELFQLVSKIYKVVPPILQETGKVKNPWPNVDAHSGVLLQYYGMKEMNYYT

VLFGVSRALGVLASLVWDRALGLPIERPKSLSTDLLMKMVQK

>GMOY004070-PA 60s ribosomal protein L14|protein_coding|scf7180000647940:20449-21524:-1|gene:GMOY004070

MAPFQRFVQTGRIAKCSAGPLKGRLVAIVDVVDQNRVLVDGPLTGVPRQEYRLNNLHLTK

YRIKFPYTAPTRIVRKAWQESDLKSQWKVSSWSQKAQNICKRSQLNDFDRFKLRYAKRQR

NKLLTIAFNALKKRTKADGSIRKLKKDKREAIRQLKSQGVKKAALKK

>GMOY004118-PA hypothetical protein|protein_coding|scf7180000647965:1347-24684:-1|gene:GMOY004118

MNTSNKSSEKFSMTDKNYKPISISPVNTKARFYICKQNLKSSTSADLLESISISRVQYQE

NCDRNQFSGTASNLGKSKQEKDIYSTTHSTFETVQNHNLATSNFKKSVRFGAHQNSEEIV

AETYEYPKCPSEHCSCSTRSSSSNSQQGASPQCICNLTSFKYASTTNNHIPSNLLASKTF

DKEGEIHVIRDYKNTVGHKLEGTLLEDLWQDENLKITDCQKLEIPLYLSKHSDLNTTLEK

PQKPNNLEEVGPLHETYVLKMDNNLNSIDISNNSNNHLKVVASTSSIIKKKENNKTLSDA

TRKAANGRSPRSESDIQKRDIQKVENEDSNLLEFNIDKVDTWMSSYESLPQKLSLKTSML

DESFQKLTKETLEAMKTLDPDLENGPSAKEEDGKDSSSSHDNSQDDSTYDEIVSVIKEIE

DDKKKDEIYAKTPDNKLQDILSYLDNVEDNCEKTLLETRLAIPTNHSEIGFVVEPDIVED

VPKLSDLLMLPNHQLARKIVALSLRANELANALLLSKEHVAKIRTEKQKILRSEKSNAAL

RMREQKKHYETIVCRHQGFIEQLLKDKGCLCEKVAALTRRLESQTQAWEHKLETEIARVK

ENILASEKIRRERWVKENTKKIKELTVKGLEAEINKMSTCHQKELTELKSQHQQQLLTAL

EEARVKHEQIENSIRESCTQDRESVIAKERAVLRERFEKQIEEEREVFNKQKQKFIEDFH

QEKEKLLKDLKTREEDMQSRKLEWQKEKEIEFQRTTKEIQEKMLKHEEKYQNRLNTIEKQ

YEADFEVWKKEYENNCKMQQVEKENAIRQYYRAERDRQIDSIVQRMDAESLKNNEEFQVK

TSRLKQKYEKDLQEAEVLEKSVREKYIETRNKLAESDAQLKNLQADIKRMKMELEHSKKM

CKDFLNEREQLRENLKSELQGEMEGLKIERDQEIRQIHKRWAFRIVFNIYFTASLKGLGD

KTTNLFSKKKDEAEKLAKEKVENAQKLAEEQAKKVGEAVQHTKSEAEDLAVGTGKKDCIV

KNSEKNVDVPFFKAREAVGLAAAEAQKAGEVVGQGVNKATSVVDNTKNTLNNTVHQANVV

AANTKHMAANIADASQQAAVNAVDQTKKAANEAINKSVKAAEQVVDQKMKDVEGAVDGAV

KTIDQAVDHKMQEANQFVDQKRENLEKTIHGAASHAQESASQQATNLLGKLHLGK

>GMOY004147-PA hypothetical protein|protein_coding|scf7180000647981:33738-37893:1|gene:GMOY004147

MNKLFFVIGLFIVSFASARVVREAPTKTEFSFEHFFKATHERFKEVEKAFLETIGASNPE

DVKKIVEQQGETLAKQFDVWADHIKKDTEDLKNLSVVKELRETVENRVEEFKGEHPQTAA

DIEQYIGKAKQDVEDIITKIKTLSESKEAENIKNLSQKLVDTAKDAFKDLAETIKENVNK

TE

>GMOY004149-PA catalase|protein_coding|scf7180000647982:3201-19384:1|gene:GMOY004149

MSRNSATSQLIDYRNNLTVSPGAITTGSGNPVGIKDASMTVGPKGPLLLQDVHFLDEMAH

FDRERIPERVVHAKGGGAFGYFEVTHDITKYCAASLFEKVKKRTPIAIRFSTVGGESGSA

DTARDPRGFAVKFYTEDGIWDLVANNTPIFFIRDPVLFPSFIHTQKRNPQTHLKDADMFW

DFLTLRPESTHQVCFLFSDRGIPDGFRHMHGYGSHTFKMINGKGEPIYCKFHMRTEQGIQ

NLDVKRAEALTATDPDYSIRDLYNSIKNGNYPSWIMSIQVMTFDQAKKFKYNPFDVTKVW

SHRDFPLIQVGKLTLDRNPTNYFAEIEQLAFSPAHLVPGIEPSPDKMLQGRLFSYSDTHR

HRLGPNYLQIPVNCPYRVAVKNYQRDGPMTVTDNQGGAPNYYPNSFAGPETDARARSLQS

CCPVSGDVYRFSSGDTEDNFSQVTDFWVHVLDECARKRLVNNIANHLSNASQFIQERAVK

NFTMVHSDFGRALTEALSLVQSSKM

>GMOY004193-PA hypothetical protein|protein_coding|scf7180000647995:193645-212073:1|gene:GMOY004193

MPIKIEEPTQQQINELRNLFIKKYETNPPIVAFHPVDINRILKEDIWCRKFLEMYDLDMN

LAFDKLWFTCTWRQEFGANDLNESDLRMDYLNDGFIFCHNHDIDGKPLLIFQTKNHIRGS

KNMDDVVKILVYYIERLHRQTNMDKITIFFDMNGCGLSNMDLEFIKCIIETFKQYYPSAL

NYILVYEMAWILNAAFKIVKGLMPEKAVAILKMINKKSISTYIDKNNCLVSWGGNDNYEF

TFEPEKKQTTPTPPSIAETNGAVHFANGSSIRVPAEFAQMTDVPERYTPEELQSDDMLRI

VPNDYIQFPRGDPSESLLQLNNTSKQPVTFKIQTTSPEKFRVRPRCGLINPGETTTVNIL

LKAEHSLSDNARDKFLIMCLPAREISNAQVGEFWKKQNNNGPNVEQHRLCCIYKDAPLPT

PSSQPMTTATQKGAGEASPRSAPASSTSALERQLRFTQVLQVITLIFLLMLSGAVIYLLM

LQLCGDAGCFSRDVASDTSEGAPCGKAKPATATNCHQWQQNLDSLTRRCNEEEKNRFDNS

DSSADNDDGNNCFGDCKKGGVLGGGYLLGLIAFARKLARCILTVSILCCIVFLTFYFCSM

SREQVYSPNALVTTQSATEFGYYAVTPYNWDTN

>GMOY004228-PA hypothetical protein|protein_coding|scf7180000648003:44929-47549:1|gene:GMOY004228

MISTKLLCLAAVMGVLAVSQAAEERICDDLIAFIDRMCVPRKYYEDCLTLLKDPSEAGIN

MQCVAGRDRIDCLDMINERKADVLASEPEDMYVAYHTKNEDFRVISEIRTKDDKDVTFCI

QPITYKVTSSSSSSSFVLFFKIFTLADFRYEGIILVKKNSNIHSLKDLRGAKSCHTGYGR

NVGYKIPITKLKNSNILKVSMDPEITATERELKALSEFFTQSCLVGTYSPHHETDRLLKK

KYSNLCALCENPEQCNYPDKFSGYDGAIRCLDKGKGDVAFTKVQFIKKYFGMIPGTTAEG

DASEFEYLCEDGTRRPITGPACSWAQRPWTGYVSNYDAVKGQEKLHKLQQRLEKFFDNGL

HAQNKESAAHLLIKEDGVYHNKPEAVDPKVYLERAGYKDVIERDGSAIRKMKMCVQTETE

YNKCETLRRAAYSRDIRPELECVQEKDCVIAVKEGKADLAAIHANNYKVARNDKLKPVVY

ESYGENDVYVAVVEPTLTHQNLQTMPIHYDGQDERAHQAAAYLNKLRNINTCQTTPSSEK

NIMIVNAKDLEQWKNKQLLCANLEKKAVTEWRSCNLEAYLPVGIFIRESMTPVEQDTIKH

LFVSLSEKFGHNGRFEDVFNLFGEYKTHEKNILFNDRAVKFVTELTNEHTNEEIYHSLRC

EANLIKKH

>GMOY004319-PA hypothetical protein|protein_coding|scf7180000648041:87954-97318:1|gene:GMOY004319

MKTMIIVIRSTSGYNLTADSKMFLNSSKDFITQSIHHMQSYVSYGHQYHWNIKTEISWGA

ISKSSSMKAMEAIRTVVVHYGQQERSTHRDLSKQGKLRMSLKLLYLFCLEVILIVFVSAI

TTNKHPDGVETYFAPSKQPKEMIVEYSWVRGFPLDQETSKIRQSFLYWIQQANRVFDWLR

KLDNGEHGDRDVQPPTIFVQGQNLGRFCSTDEQKGGSLEETKALNKPRQIVCWLKDFLDK

NNQIKQTPALHLDYKSDANTEAAPKTEAKHDMKRPLTVGKAKEILKYLKEFLKDTEKPEN

VTPNLDVSRQNQDISRGLGQSGLSEQPKHSHNDVPKAVHKRSPVSHQPAAVSYDPQMFIP

KYIEPANKLLFWLEELIKAKKNPEPLIPNAYKRAPEGMKNDSLYRNDHRTDNYNDEEDEA

KHKRSASCSFSLLVDVNLLIINSDVINLWFPVGRMKMKNEVLLEIMPAKNLYTLNFSFHH

KGLNSISILLNK

>GMOY004375-PA hypothetical protein|protein_coding|scf7180000648077:25349-27499:1|gene:GMOY004375

MTEEIETFAFQAEIAQLMSLIINTFYSNKEIFLRELISNASDALDKIRYESLTDPSKLET

GKELYIKLIPNKTAGTLTIIDTGIGMTKPDLVNNLGTIAKSGTKAFMEALQAGADISMIG

QFGVGFYSAYLIADKVTVTSKNNDDEQYIWESSAGGSFTVKPDNSEPLGRGTKIVLYVKE

DQTEYLEENKIKEIVNKHSQFIGYPIKLVVEKERDQEVSDDEAEDDKKEEEKKEMETDEP

KIEDVGEDEDADKKEQENKKKKTVKVKYTEDEELNKTKPIWTRNPDDISQAEYGEFYKSL

TNDWEDHLAVKHFSVEGQLEFRALLFIPRRTPFDLFENQKKRNNIKLYVRRVFIMDNCED

LIPEYLNFIKGVVDSEDLPLNISREMLQQNKVLKVIRKNLVKKTMELIEELTEDKDLYKK

FYNQFNKNLKLGVHEDSNNRAKLADFLRFHTSASGDDFCSLADYVSRMKENQKHIYFITG

ESKEQVANSAFVERVKARGFEVVYMTEPIDEYVIQHLKEYKSKQLVSVTKEGLELPEDEA

EKKKREEDKAKFENLCKLMKSILDNKVDKVVVSNRLVESPCCIVTSQYGWSANMERIMKA

QALRDTSTMGYMSGKKHLEINPDHPIIETLRQKAEADKNDKAVKDLVILLFETSLLSSGF

SLQSPQTHASRIYRMIKLGLGIDEDEPMATEDTQSAGDAPPLVDDTEDASHMEEVD

>GMOY004379-PA ATP synthase subunit b|protein_coding|scf7180000648077:42903-43953:-1|gene:GMOY004379

MLSRAALLSVQRPLSVLAARTAVSSSAERPVRLEQPGKVRLGFIPDEWFQFFYNKTGVTG

PYTFGVGLITYLCSKEIYVMEHEYYSGISLAIMAVVAVKKLGPPIAKWADSEIDRIENEW

NEGREAELKSLKDSIEAEKKEQWRAEGSLMLMDAKKENVALQLEAAFRERVMNVYNEVKR

RLDYQVECRHIERRLSQKHMVNWIVNNVLSSITPQQEKDTLNKCIADLNALAARAK

>GMOY004505-PA hypothetical protein|protein_coding|scf7180000648123:147125-152527:1|gene:GMOY004505

MKLTVFCVLLVFSCLVANVEGASWQDQFSNDNLKKAFNTVAKSFHEVGAKVSKALSGDSK

KKPFKSVIQSPQNPGNKTTETPNKEESQKESNSSNQPLQNSTSNTTLAPTVAHLWRNSTS

SVEILNNTDVSSADVVNEDDLVKNANSLAEIPTDAATSAAETPNKEESEKKSNSSNQPLQ

NSTSNTTLSPTIANLRAYLISLFDIFRNTDVSSVDVVNEEDLVKNSTSLAEIPTDAATSA

AEAENLKKTSSSLAEDLNNSTASAAKVLKENSTSLPSAAETFTGDNLNKNSSSLAEILKN

EAISGENLEKNGQSLDASIKVSEVVSAQNLQTIINSLAEILQNTSASIGEAISSDKENSN

SLGDVLTDAGSNIAGDSNSENLTNTINSLTDPCTVINVRALEPLAGIEIDIATDSEEDET

NDRIADGVENLKVDLDEVMTYWDKTSSKLSKVANDISKLSKVSGDYDGSGSSTMEKDLKY

LAEALNDADNKFREAKFY

>GMOY004506-PA hypothetical protein|protein_coding|scf7180000648123:156183-156960:1|gene:GMOY004506

MKLTALCTLSKAVVVGAADYGNETSPQLSKNPMQNEVDSDTGPQMKIMLGGTVPIAADAV

DALSAARQSMDSLKDALESENFFKAGDKVVANTVVTLNGLKSGLDDLTISLEKQLKKKNK

NFNPKKKPKKATPVPTNDFQDDNLLRGLEAAKAASSEKLLKQARAVVGEKFIQRLDDARV

ATGDKLTKQLQNARDQALDAQLNFADVKKMRNNLTPPLRFGVDGPDALHLF

>GMOY004552-PA hypothetical protein|protein_coding|scf7180000648143:17964-21645:-1|gene:GMOY004552

MDSKIGLNCNLNTLDDVPVENEYDLLPCQKKFRIKPEDEISVDSIISALLSRIEPSEMTF

RKYRFDVASKELVNEKQAIIFNENLYISKLKNSECRLEEEVRLLLEKDLEEIGEFNMNVE

ESFDGFVVFSKSKMRKGKNNIECGHWVRGKYNDHLSLICEKRSEYDYIDNSRIVRSELFN

QNDLMCISKTTFQEKSLDARIVDGKLIAKRDVSINKDTQRFKAVINMKGRDDVLVLDGGI

LLLMRYLVVNNFVGDFFYFSMNLFGKILHCKLTINATRKRIKVFHRTFQNAIHVTNVQYF

NNYPSEVSESYYTPNGKLILHFWHDFQYIIHETKGFIRPVERIVPKLELMWRSHHLLLDK

YRTRKQLTYDTAMAYFKEHPELTDFLHDYLLNVIKFKPLNVLDFSVRFFQKFKKVPATQN

PAPPSDGQNGPLIEKTGNAGGPVEEIDFKIPRKEDAGESNIPMSINPHADDSNNPPCYLP

QPEDELPSKDNLQPMGPIGPWATGKVDWSPMAGLTGTRPVVDRYSITRFSSNEWRAKNLE

TVKNTNKVFDKAIKNQYNSKTTHMRISSLVEKTQTETTASMRQRAQLVGKWKTTLEVAIK

AMADEISTLEEERVRLKKSLVILGVPESIAKECIDKRTGRPDTELVRDVPEEELVNELAL

LAEIRQLLKKTLEDFEQQQVENRTARERLEYDWSDKKEAYEIDSINVGLDNNSKIIMFRP

GAVRQPPEQASEQYWEHFSKETLEECEKCRQKSVRFLQQCAELKNHNMHLQQVSLRQTLN

SILLNAARDIRGQADTVEKAFVSRINCTQENLQRFENDLRDCLQKLADTENRIGHLHRCV

RSFDAAMKVSQTRMDNRSFRPNVENCRDSSQQDLIDEVATIQSSVSAMLVELDEAENVKT

DLMQLRSKLEREIMLKRRTLWLDRDRCMLLRSHYPSANALSGFASV

>GMOY004638-PA hypothetical protein|protein_coding|scf7180000648171:50279-54825:-1|gene:GMOY004638

MCAAVVCNGPEEGTQNNANAAYNPCDSTPQIECGDYCPPRCDPCVAPADYTPCIQEAEQP

CECRCDCGDYSGCCYQQPRRTLPILPQGCLMRSSAPMESDTIYRRSYIGNCGDGRSKPVL

PCNHLATVKEPMEKCTIQKLSYMPHWCAARTQPIKPHENGLHFKGPLYAVTSQKHDFVPK

GFCKREPIVPPTGICTPSSPLERCTVNRLSYMPIDVCTNPPPKPIVQTATYIRPTGPGEK

CTVQKLSYLPICIPPKEPMPWAEKKRLCPPKYENLCTTYNLSYIPNCSPQRTAAILPNTG

LKFLGTDRTDNHTVYKLSYVGSDARYTRPPAILPSNGLALPTGPMESCTVQKLSYQPFCT

VGRTTPIKPKENCIKPSGPLYCITTQKHDFVPKPDCRRAAIKPMSLICRPTGGMEKCTIN

RLSYLPVDVCEYRRPDAVVPAVGVERNSGPMEKCTTYKLSYLPNCIQPKENLPWANQSSY

QKPTGPIEKCTIQKLSYGPPGTFSRCGSCYSKKRDPLEKVNACVQIM

>GMOY004645-PA hypothetical protein|protein_coding|scf7180000648171:142395-144031:-1|gene:GMOY004645

MRECISIHVGQAGVQIGNACWELYCLEHGIQPDGQMPSDKTVGGGDDSFNTFFSETGAGK

HVPRAVFVDLEPTVVDEVRTGTYRQLFHPEQLITGKEDAANNYARGHYTIGKEIVDLVLD

RIRKLADQCTGLQGFLIFHSFGGGTGSGFTSLLMERLSVDYGKKSKLEFAIYPAPQVSTA

VVEPYNSILTTHTTLEHSDCAFMVDNEAIYDICRRNLDIERPTYTNLNRLIGQIVSSITA

SLRFDGALNVDLTEFQTNLVPYPRIHFPLVTYAPVISAEKAYHEQLSVAEITNACFEPAN

QMVKCDPRHGKYMACCMLYRGDVVPKDVNAAIATIKTKRTIQFVDWCPTGFKVGINYQPP

TVVPGGDLAKVQRAVCMLSNTTAIAEAWARLDHKFDLMYAKRAFVHWYVGEGMEEGEFSE

AREDLAALEKDYEEVGMDSGDGEGEGAEEY

>GMOY004724-PA hypothetical protein|protein_coding|scf7180000648209:24185-24511:-1|gene:GMOY004724

MKLFKVCIVVIAALIPLFNVVTSYNEACFAPPGSRASSLSKCYPKLRLWTYREDINKCIR

FRGCGDSENQFYRKRECVKECKQ

>GMOY004725-PA hypothetical protein|protein_coding|scf7180000648209:27667-27999:-1|gene:GMOY004725

MKLFKIVLVAIAALISLTKAVAGARDAACFLPDSVNGFGALQCRAHSVMWSYRATANRCV

RFIYGGCGGNRNQFSTQRECENICKK

>GMOY004727-PA hypothetical protein|protein_coding|scf7180000648209:33535-33866:-1|gene:GMOY004727

MKFLNFQFLIIVILISLTANVIAVKDAVCSLPDSADGDGKIACVAYIPSWTYRSATNECI

QFIYGGCGGNKNRFRTQEDCENKCKE

>GMOY004732-PA hypothetical protein|protein_coding|scf7180000648211:41887-46294:1|gene:GMOY004732

MLFAFANSAVKSLRQTTPRVRVFLISKNAYSSQVEYKPIRSVLVANRGEIAIRVFRACTE

LGIKSVAIYSEQDKMHMHRQKADESYLVGKGLPPVEAYLNIPEIIRVCKENDVDAVHPGY

GFLSERSDFAQAVIDAGLRFIGPSPKVVQQMGDKVAARTAAIEAGVPIVPGTDGPVTTKD

EAVGFCKKHGLPVIFKAAYGGGGRGMRVVRKMEEVGEMFERASSEAKAAFGNGAMFIEKF

IERPRHIEVQLLGDKAGNVVHLYERDCSVQRRHQKVVEIAPAPRLPVEVRNKMTDAAVRL

AKHVGYENAGTVEFLCDETGNFYFIEVNARLQVEHTVTEEITDIDLVQSQIRVAEGMTLP

ELGYTQDKIHPRGFAIQCRVTTEDPANDFQPSTGRLEVFRSGEGMGIRLDSASAFAGAII

SPYYDSLLVKIISHASDLQSSASKMNRALREFRIRGVKTNIPFLLNVLENQKFLHGVLDT

YFIDEHPQLFQFRISQNRAQKLLGYLGEVLVNGPQTPLATPLKPAEISPHVPTVPLDLSP

EAVEREERGEAKVTEPPKGLRQLLKSQGPEAFAKEVRSRKNLMLMDTTFRDAHQSLLATR

VRSHDLLKISPYVANKFHNLYALENWGGATFDVALRFLHECPWERLEEMRKLIPNIPFQM

LLRGANAVGYTNYPDNVVYKFCELAVQTGMDIFRVFDSLNYLPNLILGMEAAGKAGGVVE

AAISYTGDVSDPNRT

>GMOY004740-PA hypothetical protein|protein_coding|scf7180000648216:23043-26855:-1|gene:GMOY004740

MQSNQDPPCCVKRFLVVPGKQCIHTSAGRWQRQTVNVPPFPESVAEGEAKLAAQVGQQVS

VDQVIIEVETDKTAVPIPAPCNGIIRELLVKDGDIVRPGQPVFVIEQTAAGAAPAPKPAP

TAAPGQSPPKPAVAAAPTAPAAPFPPQPPITAKAPPPPVAPDPTIPKATLPPPEGMTQLQ

VKIPPAQYSREITGTRTEQRVKMNRMRQRIALRLKESQNTNAMLTTFNEIDMSAAMEFRK

TNLDAFTKKYGIKLGYVSIFCKAASYALQDQPVVNAVIEDKNFAEIEIDLAALANKARRN

IISVEDMAGGTFTVSNGGVYGSLISMPIINPPQSAILGMHGVFERPVAIKGKVVIRPMMY

VALSYDHRLIDGREAIMFLRKIKDAVEDPKIMLAGL

>GMOY004743-PA enoyl coA hydratase_ long chain 3-hydroxacyl coA dehydrogenase|protein_coding|scf7180000648218:36720-39514:1|gene:GMOY004743

MSAVRFLSVIGQLPRRNLVHQNGCNRVFMQNVRLMSAASEKHIRTKIVDDVMVITLDSPG

AKVNSLGDAVMREFEEILKDVETNSQVKSAVLISAKPGCFVAGADISMLEKCQTAAEATR

ISQEGQYMFERMERGRKPFVAAINGVCLGGGLELAMACHYRIATKDKKTKLGLPEVMLGL

LPGGGGTVRLPKLAGVPTALDMELTGKQLPAERAKKAGFVDLLVNPLGPGLAPADQTTME

YLERVAVQAAKDLASGKLKVNREKAGLINKITSFVMDTEFVKNKIFDTARKQVMKMTNGL

YPAPLKILDVIRTGVDKGSEAGYVAEREGFGQLAATPESKGLIALFRGQTECKKNRFGAP

EHKVKTLGAGIVQVSVDKGVSVVMKDAKDAGLARGIGQVQNGLETAVKRKRITALERDQI

LANLLPTLDYNDFKKADMVIEAVFEDIKVKHGVIKELEAVVPKHCVIATNTSAIPITKIA

AGSSRPDKVVGMHYFSPVDKMQLLEIITHPGTSKDTIASAVAMGLKQGKVVIIVGDGPGF

YTTRILSTMLSEAIRLLQEGVDPKDLDSMSKKFGFPVGAATLADEVGIDVGSHIAVDLAK

AFGDRFSGGNLAVMQDMVTSGFLGRKSGKGIFIYEGKTKGARPVNLQALEILKQKYALTP

KGANTTEDMTLRMVSRFINEAVLCLEEKILHNPLEGDVGAVFGLGFPPFTGGPFRFVDQF

TARRLVDKMLMYADLYGAPFKPAQTLLDMAKDPSKKFYPDTNVTSNL

>GMOY004744-PA hypothetical protein|protein_coding|scf7180000648219:20036-33985:1|gene:GMOY004744

MKILACRTLAGKLFRLPDMATFTAWCNSHLRKAGTSIDNIEEDFRNGLKLMLLLEVISGE

TLPKPDRGKMRFHKIANVNKALDFIASKGVHLVSIGAEEIVDGNLKMTLGMIWTIILRFA

IQDISVEEMTAKEGLLLWCQRKTAPYKNVNVQNFHLSFKDGLAFCALIHRHRPDLIDYSK

LSKDNPLENLNTAFDVAEKYLDIPRMLDPDDLINTPKPDERAIMTYVSCYYHAFQGAQQV

GNVTHVPEPTRQYTYVPNYNNTALPDERAVMTYVSSYYHCFSGAQKAETAANRICKVLKV

NQENERLMEEYERLASDLLEWIRRTMPWLNSRQADNSLAGVQKKLEEYRTYRRKHKPPRV

EQKAKLETNFNTLQTKLRLSNRPAYLPTEGKTVADIANAWKGLELAEKAFEDWLLAETMR

LERLEHLAQKFKHKADAHEDWTRGKEEMLQSQDFRQCKLNELKALKKKHEAFESDLAAHQ

DRVEQIAAIAQELNTLEYHDCISVNARCQRICDQWDRLGALTQRRRTALDEAERILEKID

ILHLEFAKRAAPFNNWLDGTREDLVDMFIVHTMEEIQGLIQAHDQFKATLGEADKEFNLI

VNLVREVESIVKQHQIPGGLENPYTTLTASDMTRKWSDVRQLVPQRDQTLANELRKQQNN

EMLRRQFAEKANVVGPWIERQMDAVTAIGMGLQGSLEDQLHRLKEYEQAVYAYKPNIEEL

EKIHQAVQESMIFENRYTNYTMETLRVGWEQLLTSINRNINEVENQILTRDSKGISQEQL

NEFRSSFNHFDKNRTGRLTPEEFKSCLVSLGYSIGKDRQGEMDFQRILAVVDPNSTGYVH

FDAFLDFMTPEYCIQRMPPYKGPNAIPGALDYMSFSTALYGETDL

>GMOY004756-PA hypothetical protein|protein_coding|scf7180000648225:5152-8897:1|gene:GMOY004756

MSNNLIKIKPQTNRTDIDEGGGGDNAYNLSMTLAPSELQRILHQSHYTNASSFLSNSFKA

NMLIDSTTICERSGALDSIEYPKEKADILFSQFLEVWKIRANGSDVFDTVQDLIFACAGV

LESAKQEVSRYGEEKGRSNSWKWLEQELKTWKLLYALYKDRTLVQCGDEAMGFDGATLGG

SEKEVISQLYNCNSTLREYQLIIDWLEACYECRDYGPLIGHTTDRSVSWENTLFQLKKKK

QLAFGTGAEIVKSIDPDAPIREKRPLHALDEEDSMRLSRCLFQEVRQGHIDEATSMCKYY

GQSWRAAIFEGWRLHEDPNYDTIGANKSEKLPIEGNPRRDIWKKCAWLMADSKKFDEYTR

AIAGAFSGHLESLKSLLGNSWEDLLWAYLKVQVDIRVESEIRACCLKPYHPLPEEYWNSK

MSLEQIFDELLVHNDAEVRDYAQSKIGITQKYFILDNISELLQQMRHWIDNEDAECETEC

IPITPHMLRFLTHIVLFMRQIGRVDIEDAAYQQNHIIAAYVECLITMGDAQLVAFYTATL

PSKTSISLYSKFLEKIHKKEARSIALEEAINVGLNVEEITRHAVETIRHAMPTDSDMTGS

LQAGEITEFDQRKIKALEWLTFLPAQRGELLWQANAMTRTYLAENKIECVRAISNMIPSD

SLAQIIKLYSTKDDIPYREDCSIKEYLSYKVYLAAIDSFDEWTRLYHNRPKEPEPAMVGA

NFTERVACEHREQVYRAELNHWHITLQEHVKVCRDSLYNVLVFPEMGWLIDPDPPKTFNS

NNVAWETRLTQLEKLRSICIPEIVLLLYKVLHVSKDFQGCIKLADDIASETRQLYKVYTK

HKLADLLSKLADSSLELLNNKLDPWAIVLLTSPLIRLLHMFRTVYQKGCFSILYSVGGNP

LKLWTIHTKNGYVKRVYDEDIKSLVLEIMGTNVSTMYISTPRGNNQQLGIKLPFLVLLIK

NMHKYFTFEVKIVDDQRFMRRFRVSNFQSKTSVKPFCTSMPMGMSPGWNQIHFNLADFTR

RAYGTNYMETVRLQIHANVRIRRIYFTDRLYQESELPNEYRLIGQPKTKKPISWRVPAAR

PPSPQTIRGATARSAKTATPAPEPKKEELTEEAQPPPPPQPPPEKKIE

>GMOY004791-PA Chd64|protein_coding|scf7180000648252:151471-171909:-1|gene:GMOY004791

MAPRNKEQEQEVLNWIFEVLGEKVPAGQYEDILKDGIVLCKLINKMAPGSVKKIQERGTN

FQLMENVQRFQAAIKKYGVPEEEIFQTADLFERRNIPQVTLCLYSLGRTTQHHPEYNGPT

LGPKMAEKNVREFTEEQLRAHNAELNLQMGYNKGASQSGHGGFGNTRHM

>GMOY004878-PA hypothetical protein|protein_coding|scf7180000648308:67727-90182:1|gene:GMOY004878

MDEILRASSPGDRGSSFAGRASERRSERNSKFMQPRGMDPEKNVRKLRKLFGVSHTIQKQ

TTRRLSVTDQEREEIERKRGQQLKELRQQRISRLGANHRYVLEIVADLMGTDTEEIVTGV

ADEQSFVDLLSGMFDKQGPRACMISYATMEGYPADSGRYVESMKRQLVKRTVIHRSDTVE

LFGKWVIVYRNNNKKALENRTVSDDLAMFCWNADKKDLCLYVIKNFMDRVLTKSLEAVSE

FGVAEMDQKRKFFHTLDMFNIFLRSSEATVSSRVNFDVSHELYKGFLLVKYQIETSSKDM

NRVRSVERYFGQWMRQIQGILVEGKQILKDGPDVGPLQMLVNWRRMLARYTSIKEFVSSK

AFNNHKICLTLSRSSKLLKKWAEIDNQVTLVLNEAKDNVRYITSLQKFWDPLYRSSPDGI

ISSLPGLMVAIRNVSKTAHYFNTPSNVTGLFVKISNQLTITNKNYLTDNGRIKLWTMSPN

DIIAKIEKCRDVMQSYKEQYYETCKEMSEAAEKPWIVSSVYIFTCLEKFLDRLDKIKVIF

NTEITYSILDRIRISGMEKFSAMIKGARATISSKPYDPLNYRIETFDMDFQKFIKEVERA

EVGMQQFVKQQIADVPIAESVILILKRFERLNLECLCLDRRYLEVAEMLEQEMFLLKDVY

NEERGNPFIARNMPARGRPDYVDTFRTVRYYNYMNGIICHSEMTYHKAWFDYAEEVRCLL

NSPILVCNKESAEYTINLDPAIEQLIKETEWMWKLKLEVPNIAAVVTYCKLRILSPAWKL

RIALKKFDRLRSSITPVYINIMRFRLQEISVLLKPALSTVTWLSENLEDFVEDAEKHIDE

IDLFYRMVLNIDEIRISQEMLSLQNYLYVYKPSAPVTSEKFYESCNDLLQEIQKELEKKS

VCMERAVIDLCNMFVELLNFGPVDSKGRRVFQLAPEKITDTNWRVEGNMPIDKWDWIQFQ

KIYRTIYLVPDDVLKTLQFKNYENIRYELHHLRNDCMDLFSYYNSRMISSLVGCSKRSLD

FVRYSILRTNWRTDPQPPILYSAMDVDLENGCSIDPNLENMQANFHRAILCCTEINYFVT

TWGKQAKTFARKLRRVTVDENRYERNYYRFVIEHKDIIRAVQNLATGLMMYKPDIDDFLK

EQFEKYKYLWSAERENMIQAFVNTSPLTVDIRDKFIYYDNITTDLETKHARHTIGPIEIR

MEKAFTKFVEESKKWKTALGQLLSAQYKKQLDEMVDFISEQENILGKPINDLDDVRLAMM

CLEKVRDNFIEMDMELTLITDTYALFAQYNIYIPQDDYDKVDSLQLSFNKMLDMAKKVSQ

RISEMEGPLLQELNDGIATFVTELDKFNDDFAANGPMVEGISAKEASDRVFLFQNRFDEL

WRKYEMYSSGEKLFGLPITEYPILQQRKREFGYLNRLYSLYIQVLKTISDYYEMPWAEVD

IERISTELADFQVRCRKLPKGMQSWPAFIDLKTKIDDFNETCPLLELMTNKAMKERHWVR

LNALLKSDFDPTSSKFTLGKLLEAPILKHKEDVEDICVGASKELDIEAKLKQVMTDWSVV

NVQLGMFKNRGELVLKGGETLEIIASLEDSIMVMNSLSSNRYNAPFKKEIQLWLGKLVGT

GEILEKWLMVQNLWIYLEAVFVGGDISKQLPMEAKRFSNIDKNYVKIMYRAREIPNAVEC

CTGDETLATSLTWLLDQLETCQKSLTGYLESKRLLFPRFFFVSDPVLLEILGQASDPTSI

QPHLLSIFDAVATVDFQEKTNDVITIMNSANNEKVPLENPVQCSGSVELWLNRLLKEMQD

TMRTVLAGMAVGLNDPEFNFAEEFPSFCGQAGVIGVQLLWTRDAEYALRKCRTDKTIMKR

TNAKFLALLNHFIDLTVKDLTKLDRIRFETMVTIHVHQRDIFDDLCQLRIRSAGDFEWQK

QARFYYNEDNDDIIVGITDVNFIYQNEYLGVTERLAITPLTDRCYITLAQAIGMSMGGAP

AGPAGTGKTETTKDMGRALGKLVVVFNCSDQMDFRGLGRIYKGLAQSGSWGCFDEFNRIE

LPVLSVAAQQIYIVLTARKEKRTTFIFSDGDIVSLNPEFGLFITMNPGYAGRQELPENLK

IMFRTVAMMVPDRAIIIRVKLASCGFKENLILSRKFFTLYKLCEEQLSKQVHYDFGLRNI

LSVLRTLGAQKRSNPSDTEETIVMRVLRDMNVSKLIDEDEGLFLSLIEDMFPGIKLTTAV

YKDLQKAISNVVEECGYVNNPEWNLKVVQLYETSLVRHGLMLMGPTGSGKTSCTLAMLKC

FTEMGRPHKEMRMNPKAITAPQMFGRLDVATNDWTDGIFSTLWRRSLKVPKNHNTWIVLD

GPVDAVWIENLNSVLDDNKTLTLANGDRIKMADNSKLVFEPDNVDNASPATVSRVGMVFM

SSSVLKWVVYKDAWVKKRSPTEQDVFQKCYDAIYDDAHVYLQTKLIAKMKILEAIYIKQM

LEILDGLLADTSNRSDRYLERTFLFALMWTLGAVLEIGERDKLEEFFLKHPSKLKWPKKQ

PGETVFEYYVDDSGIWQHWNNRVERFIYPEDSIPEFSAILVPNVDNVRTAFLMHNISKQR

KQVLLIGEQGTAKTVMIKGYMLTYDPEVHLFKSFNFSSATTPNMFQRIIESYVEKRVGTT

YGPPGQRSMTVFIDDINMPIINAWGDQVTNEIVRQMIEQHGFYSLEKPGDFSTIMDIQML

AAMIHPGGGRNDIPNRLKRHMAIFNCTLPSNNSMDQIFSQIGCGYFCLARFEEEVVNVVP

SLVPLTRIFWQHIKGKMLPTPSNFHYVFNLRDLSRIWEGILKVAGDECKTVSDILKLWRH

ECTRVIADRFTDYKDTTWFLERMKKDAATLLGEYFEEYPEEETFFVDFLRDPPDVGEDDE

DVSLEPPKIYEEIPSLERVKERVTWYMGQFNEYVRGYHMDLVFFRDALVHLMIVSRILSM

PRGNALLVGVGGSGKQSLTRLSSFVAGYKFFQITLTRAYSVNNLTDDLKYLYRTAGLEGR

GITFIFTDNEIKDEAFLEFINNILSSGEIANLFAKDEMDEIYNEIIPIMKKIQPRRPPTQ

DNLYDFFLSRARSNLHVALCFSPIGEKFRARALKFPGLISGCVIDWFQKWPEDARVAVSR

HYLKDFEIICDPAVKEQVIDIMSWIHEVVSDSCLSYFERFRRTTFVTPKSLISFLESYKK

LYKEKQNNIVVMAERMSSGLYKLDEAGASVSVLKKELVEMNKVITVATEEAELVLETVAE

STAAAEIIKTQVAEKKSQATELVKAISADKEVAEAKLEKARPALEEAEAALKTIKGSDIA

TVRKLGKPPYLITLIMDCVCILFRKRVKPVRPDMEKTFLQSSWEESLKVMSDTAFLKKIV

EYPTDLINAEMVDLMVPYFNYSLYTFEAAKVACGNVAGLLSWTIAMAKYYEVNKEVLPLK

ANLAVQAAKYAKAASDLQEAEDLFAAKERELAKVQQQFEEAIGKKNAVLEEAQKCQDKMD

AATALISGLAGEKIRWTEQIAQFKSETERLVGDVILLTAFLSYTGPFNQEYRNDLQAQWL

KQIVERRIPISSNINIIDSLTDRTQIGEWNLQGLPSDELSIQNGIIATKAVRFPLLIDPQ

SQGKAWIKNKEKENNLIVTNLAHKYFRTHIEDAVSMGLPVIIEDVGEELDPCLDNVLDRN

LLKVGTTYKIKLGDKEIDYNADFRCYITTKLPNPAYTPEVSARTSIIDFTVTMKGLEDQL

LGRVILTERKELEEERTNLVETVTGNMKKMKELEANLLHKLSTTQGSLLDDVTVIEVLNT

SKNTAIEVKEKLDIAKVTEAKINAAREEYRPVATRGSVLYFLVCTMAKVNVMYQTSLVQF

LDRFDASMHNSAKTHITRKRIKRIIAYLTFEVYRYKSRGLYEKDKFLFVLLMALNIDRQL

DLVGYEEFQNFIKGGAALNLNDCPAVPFRWITDETWLNLVQLSHLGPFSNILTKVSNNER

AWLAWYKKECPENEVIPDGYNALDPFRRLLLIRSWCTDRTLSQCRKYIANSLGDRFAEPV

ILQYDALLEESKPLMPIICFLSMGSDPSSNIEALAKRNELKCYPISMGQGQEIHARKLVA

NCLEDGGWVLLQNCHLGLEYMNELTLLILDLERGDESAVSKNFRVWITTEPHDSFSITLL

QMSLKFTNEPPAGIRAGLKRTYSNLSQDFLDYSQSVFYHPLVFAISFLHSVVQERRKFGP

LGWNIPYEFNSADWYASCLFVQNHLDDLETGKGISWTTVRYMLGEVQYGGRVTDDYDKRL

LNTFGRAWFHDHLFDETFEFYKGYKILSFKEQEAYLAAIEELPNIDPPQVYGFHSNAEIT

YQTNTTRSILDTIVAIQPKESSGGGGETREDKVARMVKEMQSKAPMPYDLFEVKERLKYM

GALGSMNIFLRQEIDRMQRIIILVRATLKDLLLAIEGTIIMSEQLRDALDNIFNARVPGI

WQRGSWASSTLGFWFTELLERNQQFHNWCFNARPVVFWMSGFFNPQGFLTAMKQEVARAH

QGWALDQVSMTNEVLKISLDEAKKPPKEGIYVHGLFLDGAGWDRKTSRLVESANKVLYVL

MPVVHISAINSVAPKSPKLYQCPVYKKINRTDLNYISPLWLNTARSADHWIIRGVAILCD

IK

>GMOY004926-PA fatty acid synthase 1|protein_coding|scf7180000648340:4314-11699:1|gene:GMOY004926

MPARFVENSEPSVPMNLENRYDHPSSNGVNSDIVITGISGRLPESTNIEEFRKNLFEGVD

MVNDEPRRWSKGLYGLPERNGKIKEEDLENFDQTFFGIHQKQADGMDPQMRMLLECTYEA

IIDAGLNPAEIRGTRTGVYVGASSADSENYQLSDPDRVDGYGLIGCARAMFPNRISFSFD

LKGPSYCVDTACSSSLYALSQAFADMNSGKCDAAIVAGSNLCLRPTMSLQFKRLSMLSAE

GKCKAFDESGSGYVRSDAVVVILLQRVSVAKRVYATILNARTNVDGHKEQGITFPNGQMQ

NQLMRETYGEINLDPAEVNYVEAHGTGTKVGDPQEVNSITDFFCKNRKSPLLVGSVKSNM

GHSEPSSGLCSIAKVLLAMEQNVLPPNLHYKNPNPDLYGLLDGRLKVVDKPTPWEGGIVG

INSFGFGGANAHVILKSNPKPKVITPTVGPPKLILCSGRTSDAVQEFLEDADQHKDDDEY

LTLINDIHSRPITGHYYRGYSVMDSKGALQKDVAEISPEPRPIWFVYSGMGSQWASMAKD

LMVFDTFKKTIHRCCEILRPEGLDLMEILTRSTEKTFENILNSFAGIAAMQVALTDLLTS

LNIVPDGIVGHSVGEVACAYADGCLSMEQTILAAYWRGKSILDTKLTKGKMAAVGLSWEE

AHKRLPADCFPACHNSAENCTISGPEESIDAVCQKLTAEGVFARAVKSSGYAFHSKYIAD

AGPKLRKSLEKIIPNAKNRSPRWISSSIPEVAWNTAIAQQASAAYHVNNLLSPVLFHQAL

QHVPKNAICIEIAPTGLLQAILKRSLGNETTNLSLIKRDYENNPEFFLASIGKLYAAGAQ

PQIMTLSKPITYPVGRGTPMLGCKVGWDHSQKWHVPKFDKMTSSGETVIEVDLSKEEDAF

LAGHTIDGRILFPATGYMTLAWMTYAKMRGHEFQNTPVVMENVVFHRATILNKEGVVKFG

INFFDGTGAFEICEGGSLAVSGKISIPEHVDNEQLPLDPLPVSTVAKELSTSDVYKELRL

RGYDYAGIFRGITASDSLSSTGKLQWVDNWISFMDTMLQFSILSKNLRELYLPTRIEKAI

IDPNHHLEEIAKLTPEQQTKEGVPVYMYDDINVIKSGGIELRGMKASLAQKRPSNQNPPT

LERYAFIPNENGVDLNENHEKAHFHALSVALRLLNENSSGALKIKGVELADGRNPDSLLA

AKIVQVLEGEPSLTAEIAVATSNSNEEAISAALGPDAGVRVISKNILKEPVDQNNHFVFG

VDVLSRPDTAVLVNSLASIKDNGFLIFEESAYGYRNSGRNLLTEFGLVVASEQSCGNSRL

LVMARRPVDLKQRKSVVINVTEKHFDWVEELKAALAKAAENEQYVYIVCQGEEAFGAVGL

MTCIRRENGGNFARLVFVQDGNAKPFSFNDAFYTKQLAKDLISNVLKNGLWGTYRHMKLE

TNAATLQVEHAYVNALVKGDLASLKWIESPRSTPNISNDFELCTVYYAPINFRDVMLSSG

KLSADALPGDLAQQDCVLGLEFSGRDSKGRRIMAMVPAKSLATTCVAAKIMTWQIPDKWS

MEEASTVPCVYSTVYYALMVRGQMKKGEKILIHAGSGGVGQAAISVALHHGLTVFTTVGS

KEKREFLKKRFPQLKDNHIGNSRDTSFEQMIMRETKGRGVDLVLNSLAEEKLQASVRCLG

LNGRFLEIGKFDLSNNSPLGMSVFLKNTSFHGILLDSVMEGEEAMQKQVVELVAEGIKNG

AVRPLPTSVFNDQQVEAAFRFMASGKHIGKVVVKIRDEEPGSKAIKPPTHLVNAIPRTYM

HPEKSYVLIGGLGGFGLELTNWLVFRGAKHIVLNSRSGLRTGYQALMVRRWQEKGVKVLV

DTSDVTNASGCKQLLTNANKLATVGGIFSLAAVLRDDILENQTAKDFKTVCDPKVTATKL

LDQCSRSLCTSLDHFVCFSSVSCGRGNIGQSNYGLANSAMERICEARQASGFPGLAIQWG

AIGDTGLVIETLGDNDTVIGGTLPQRMNSCLQTIDLFLQQPHPVLASMVVAEKRKSDQSG

GVSLIASVANILGIRNIKNIPEKSSLADLGMDSLMGAEIKQTLERNFDIVLSAQEIRQLT

FGALTQMDSGPARAESATSPQTTSPSAFGDGTQDVFSSELMPKETIVRLKSKAPVDSNKR

PLFIASPIEGFADPLKNLAELLDCPVYGLQCTANADIDTIQSLASFYLKEVRKIQAKGPY

VIAGYSFGAAVAFEMVINLQSHDIVSNYIALDGAPKYVSWNTSSFKDRRSMDDGDNYDYG

LAYFAMVVANMDYAFVVKMLLDKPTWQEKLAKCAEMVAQEIKKPIDLIKQAGEIFYKKLS

AGHNYKATTKITCPVTLVKPSENYVQLEEDYGFNELCQKPVKVFTVEGNHRTFLVEEASL

KTIESELNRLTA

>GMOY004931-PA NADP-dependent isocitrate dehydrogenase|protein_coding|scf7180000648342:114337-124015:1|gene:GMOY004931

MATKISAGPVVDILGDEMTRIIWDSIKEKLILPFLNIELHTYDLGIENRDKTEDKVTIDC

AEAIKKYNVGIKCATITPDEKRVEEFKLKKMWKSPNGTIRNILGGTVFREAIICKNVPRL

VNGWEKPIVIGRHAHADQYKATDFLVPSAGTLTLSFVSKDGGTKIEQVVNEFKGAGIALA

MYNTDASIIDFAHSSFKYALARKLPLYLSTKNTILKKYDGRFKDIFEEIYQKNYKEAYEG

ENIWYEHRLIDDMVAYVMKSEGGFVWACKNYDGDVQSDSVAQGFGSLGLMTSVLLCPDGK

TVEAEAAHGTVTRHYRFYQQGKETSTNPIASIFAWTRGLLHRAKLDNNTDLKSFAETLEK

VCVDTIESGHMTKDLAICIKGMNKVERLVRQHFIRIGWLLQIFLILLLLSVAETYPPNNN

YRDLDICNHWNGRRHFLELGERGDLHARNVTTSAFRSTLFSPSSSNNALKNRTSTDIWYQ

CNLELVTCAECVIRITFTHANFSKTCERIANSGVGKSSMCPCEHIQFSEPPYDTTISGQE

FCGDGKVFRSKTRTLLLKFFYRATNSHVFSLQYFSERNVKIISGSPRQTIISNYSNQKSM

QTQVISTPYFPMTYPRDYGIEHILTCEADNCHVRLDFTDFQLGLTSTLEIFDSNGQMLDS

YTGEHFRPPIIISSGKSLLLQFRGNGNTGAGFRAEVTFISAKQLKEDRLLPYTDCGGMVS

GPGGAITMMNMIENSTDVRFFDCIWIIKPGNNYMMMKTHISLRVQEFHGMASRSDLTIRQ

GTTSDALEIENVVWPNNGMSKESHVVPILTGYYIRLRGVFGMSSKLAIVYSVFNYLNCYI

GSEFLCGNNHCISIRLHCDGFDHCGDGSDEPDSCEEDWAHLQNDRRWYSHKPNYYFPKIE

QYPDLKTATGIFIVSTLGIFAVLSGWMVILYRMGVRARHQRELQNHLQTISELLDRQEEI

TPDEPPSYEAPPDYEEVIKIGMEQEMRQHMDRPRRHHRHRRRREREHCDRERSCSRAPSN

ATVQSSVPIHCHCAEERPTTSAAAAAATTTAPVEALSHEKPTENAIAGNSNQSVSQEITQ

RLETNAENCGATGTSQASEVSTSTSNDGCNRCKHYKRELKTKCASTSPMITNKINNNNNN

NSCATNTQLEENSRDFCDDSLNISLTLANNTNDSAGTSSRPKSSSNCTEQTYLKRSWIVV

SNGSHSYKMPRLRHTFSSPEPFQIYCNDWLSNDTQNYGSILPYERSFISSNNTSYFGSEL

SRDTSALSFSVKKQYQATTSTSPTNSDSGNIHRQNTLERRRKRMLMSELDSDDGSKISCF

GNVNKQLRVQSKLKQSTQTLRRHARSKSFSNLNQNRSRKVMKRSSSVDLVTSNQRLTSEI

FHLSEAKIFMI

>GMOY004957-PA hypothetical protein|protein_coding|scf7180000648344:50172-50529:1|gene:GMOY004957

MDAIKKKMQAMKIDKDAALERASLCEQQARDANLRAEKAEEEARQLQKKIQTVENELDQT

QEALTLVTGKLEEKNKALQNVSIYV

>GMOY004982-PA hypothetical protein|protein_coding|scf7180000648360:9145-11878:1|gene:GMOY004982

MESIDEERVNLNLNEIWKACRKIQDAIFRYGFDICAEFCPHDPKGKGLISESLFSAILGK

YKRIIGLSDFELREVTDYFRLRDGRVVYKQFCKVVCSENHDKSQKKDLATGLEWNDPMHV

NVIPRPEDRRQLCLVLIKIALVSHLPLKPYFQDYELISNNVGTVTVSHFSRVLHFLKIPV

SDNDFLLLLKRYLKDSYMVNYVAFITHIESIIEYLKQRNLTDNAQQIVKDFPGCIIDLEL

PELPPVEGTNMAQRIFKDYCDHPKENRTDCDVVVCIQHQVYKNRVRIREFLEGFDLLHTG

TVTINQFERALHNMGVGKFITQRDFQLLCYRYRDPIDTNRIMWRIFEDEVDRVFTVKRLE

KTPLMQVQSPPKNIKEMPRVGSLDWQEVSEKTRDLCEEALRKIQIRIRNRRLHLHPFFKN

YDKVNSGHIHCRQANQIFTTNGILLSNDELNALIDRYGNELGFNYTRFLEDADPAEYAIP

KLMPPASMQTACLSFKNNAADTENEEYVIQLLTKAKRQAIVKSIPVIDFLQDFDRHREGE

ILEVDFRRGVDNADIKMTPKEMDTICKIFRSPKRACCVLYRDFCKALDEIFVQIETVAGD

NEIIAVPLVHLANLDCVECFMNFEERTICSQALMKLARKPDEISNLSSVFKDFDRENCGT

IRKNQLIRGLTVRDMHYMISSREFEAVFKCFGVQRGLDLEFNYREFLNILSIIYQNGQTK

RNY

>GMOY005064-PA hypothetical protein|protein_coding|scf7180000648400:135771-137702:-1|gene:GMOY005064

MDDDDTDQSLISSETKEKRDGENKSATVSSNDKKAKSDPGISITCEGYQQAKRTRQERRS

IYADIDEKNLGRVYSVSGPVVTADNMVGAAMYELVRVSYFELVGEIIRLEGNLATIQVYE

DTSGVTVGDPVLRTGKPLSVELGPGIMGNIFDGIQRPLRSIRDVTELIFIPKGINVASLP

RDINWDFTPVTGVRVGCNLTGGDIYGVVYENTLVKHRLMLHPRSKGRITFIAPPGNYTLD

DVIMETEYDDIKTSHTMLQVWPVRQARPVTEKWSCNLPLLTGQRVIDSLFPCVLGGTTAI

PGAFGCGKTVISQALSKYSNSDVIVYVGCGERGNEMSEVLRDFPELSIEIDGVTESIMKR

TALVANTSNMPVAAREASIYTGITLSEYFRDMGLNVSMMADSTSRWAEALREISGRLAEM

PADSGYPAYLGSRLASFYERAGRARCLGNPKREGSVTIVGAVSPPGGDFSDPVTSATLGI

VQVFWGLDKKLAQRKHFPSVNWLISYSKYTRALDDYYDKNFPGFVPLRVKAREILQEEED

LSEIVQLVGKASLAETDKITLEVAKMLKDDFLQQNSYSPYDCYCPFYKTFGMLKNMIAFY

DLARNAVEQTAQMENRITWTKIQQFMGSLLYRLSSMKFMVSKG

>GMOY005107-PA hypothetical protein|protein_coding|scf7180000648422:30682-34285:1|gene:GMOY005107

MNISRSLLHFPRYCNWSVNLTQIFKNNSLQIPRHFLRADRYQLVTELCDIKRHVPEINKE

KTALLVFADDAEEILVATVTDILRRSGVKLTLAGLCDDEPVRCSRDVLIKPDTSLEEAKK

KKYDAVILPAGLECSRAMCQSVSLGQYLDQHEKEGRFLAAIGCGPIVLAAHGIAMTKCVT

AYPRCEGLENLKRFYKYVDDTPWMEDVQLLTSPGPGTAIDFSLKISEALVEGSGEILIAV

ISDILRRAGIEVSVCGLCDSEPTKCSKDVVIKPETSIYRAHKYKYDVVIIPGGLEGAKTM

AKNQTLGKYLKQHYKEGRLLAAICCGPLVLAANQIAAGCRLTSYPARKPDLEKIYKYVDD

EIIVQDGKLLTSRGPGTAMKFALKISEIVAGNVKAREVAKEILMNDDTCCK

>GMOY005224-PA hypothetical protein|protein_coding|scf7180000648483:9558-11168:-1|gene:GMOY005224

MLSTRNILCCLAEHHLLIGKRYESSNKVFQVYTERVNSGVLLADKSQKHTATLLDTLYGK

IQSYRPSSKSPNTGGHLLGKLFGRSTNGSSVQLVNKSSPRGLYIWGNVGAGKTTLMDLFF

NCCTDIKYKNRVHFNAFMTDVHARIHETKLQQGPSLRITDSENPQPFDPTLPVAKAIARE

SWLICFDEFQVTDIADAMILKSLFTHLFNEGIVCVATSNRHPKDLYKDGLQRSNFLPFID

VLLNRSKVADMDSGVDYRKIAQSGDTNYFVKSETKALVEMERMFKILCSQENDVVRERTI

THLGRDLKFRSACGRVLNSSFKELCDRPLAGNDYLQLAQVFHTVLIHDVPQLTLRMKSPM

RRFITLIDTFYDNHIRVVISADVPLDKLFNFSDKSSGLADDQRMLMDDLKISQDSKEAGA

SVFTGEEELFAFDRTISRLYEMQTKVYWEKWAKPH

>GMOY005336-PA mitochondrial processing peptidase beta subunit|protein_coding|scf7180000648542:10569-12331:-1|gene:GMOY005336

MAMRVLGISQNLRPLSKGVDMLKRFKASKAELMRKCLLNIPATQVTIMDNSLRVASEDSG

ASTATVGLWIDAGSRSETAQNNGGTSKRSQTDLELEVENMGAHLNAYTSREQTVFYAKCL

SKDVPKSVEILADIIQNSKLGESEIERERSVILREMQEVESNLQEVVFDHLHATAYQGTP

LGQTILGPTKNIKSIGRNDLQAYISTHYKASRIVLSGAGGVKHKELVQLAEQHLGKMDNT

YDGKPPSMDPCRFTGSEVRVRDDSLPLAHIAIAVEGCGWSDQDNIPLMVANTLIGAWDRS

QGGGVNNASNLARASAEDNLCHSFQSFNTCYKDTGLWGIYYVCDPLECENMLFNIQTEWM

RLCTMVTEAEVERAKNLLKTNMLLQLDGTTPICEDIGRQILCYGRRIPLHELEQRIEAVD

VKNIRDVAMKYIYDRCPAVAAVGPVENLPDYNRIRSSMYWLRV

>GMOY005442-PA hypothetical protein|protein_coding|scf7180000648591:115103-128414:-1|gene:GMOY005442

MASTAVVLHILICIFSLSKVIKAESNCKNSCAEDENLFKYRDGHVYEYVFDSAMAIGVKT

SDNSQNDDTSMRITGVAKIFTEPNCGYTLQIGAFKVSAAEAIQKKIMQNIQKPVHFTMVN

GKLNSELCVADGGENSYSLNIKRAIISMFQSNPEAKHEIDIFGECPTRSSVAKVGSIKIV

NKSRNLNSCVYRQIIKSGFLRNIANTKSGYGVNTNALLDANFAKESKIENGIISSIEVTE

EYTFGNKVGGKTIPNVYAKVKTGMRVKSLTGSPSTAPLYGSRSASIIFQEPETYTAKNIA

AVKAVFGELINHVEDHVKLGSANGFVELIRLMRASDTDVLMELSAFPHQKQDLARRVYLD

ALFRTGTSESARAIIKQFNKMTEKEKTIAMEALKLVEIVDRDTLNLAANLITPNAPKETY

LALGMLVSNFCTRNSCDQGEIDMIFKKITDVLKKTHCKANTRNEENRLLFLLKGIENAKH

LAKMVNSSLLECVSEGRSNRIRIAALQAFSSANCDISLQKKAMDLLSDHNEDSEIRIKAF

LAVIKCPSAEAANELSSIVNSEPVHQVGGFITSTLKLIRDSTDESRSLQREHFANIRITK

KFPIDLRRYSYHGEISENLLGSSVNYKLIYSQTGFLPRSSALNIKTNIFGIDLNVFETNL

RMENIENILQFLMGPKGIINENRNSMLKLDEYTHPASRKRRSIVDEAAKAAKRYKTYGSK

FSNDVNLDISLKFFGSEMMFLSLGDDLPNSFEDIYKQISSGIDKIKNELGSYGKEFINHD

LFMDTTFNYPTALGVPLELSFQGFAASKVNFGIGVDIDSIFVHGAYLNKKYKCKIEPSID

VNLMVGLGFNAYVLSTGVSTSVNMHSATGSAIELALIHEGAGFNMEFEMPREKIEFINIK

VAHSFYIQENDKPIDKRLILNRNNKDPNAIQMEGCYNQLESLGIKPCISTLGTLKQEDDA

TLPSEFIFSLHVMSVKKFNIKGYRNGAEGNVEQWKLDYSTPEGAYDTSLTVEIGTKSRFY

GRVCVENTKQHYGLEAGVSNDNHELVIYVQHEEGKDIKKSKVGFMKSGNEYRPVIEIRMF

NGGLSDELNGYKVDGRVVVASGDGEGATKKYNFDNLQIMDKENDGIIINGWANIGKTSLN

TQLLIKIPKDSTYAVEGNFEVSEVYSAGIFISDENSPDHIYGASTSIRVIDQLVNISILA

KYAKYQFAANSELEYIKQGDNPSISSSKFANSVSLKYSENNLIFLKLSGLTEGENKFEIV

ADMNIQGQKKKKKGSLSIKFAAHQRAKSDYSLSINGKLNDNFVELTALCDVTGNQYNLDN

SLSTSLGTLITLKGKINQSLKLIFLADSDVVVDLQGTTRFSNKDSQSKWNLKINGAEDKT

IVDFNFLKNKEEVVKLKFEKTISEEKLTAAEFNLLADDFLDVKIDFKVSKIGKGELAAVI

ESLKHKKQVEVNSNFHVQNPKYDVEVLLVCDKERKLFVKSDNVLDRTAQIYRTKNIIQSG

GKQTSFDANTIIKGHSYVDGDIDANFVFTFSDGRILDGEFKQKMNTNIKTGLTHGIAEIN

LSDNLSNSGPKRSLILTTKVDKLDIKKKEFLVDFDILYTNLHNQKLQIVAQTKNLAKQKP

NAAIHFSTKVSGDLVESPITASFIVDEFSPAHAVFKLDFIYGSEFQTYCSCNYHVSELDK

PATYAIQMQVQKVNSTWKTFELSSKGQYENNKQQTLFELTLDEKLGSGDFLRLNIASKIL

TNKSVNALLLFEIASNHMEPLKIEAQSERASVSNEGSTDGEVLLSINYGSKFGKITNKYK

FSKSEIIKHSYFLDTSFDAMKSLEININRVNGYHWGVEVKHNEEPYAVDLELFGGKPKRG

FDLKITLPNVNPILVVVIHEILDTHKAKLQLDIQNFLNLDFKLNTEASYNNFDEFYLVAR

WNSARLQLNNYDLMLRTENKVLNMELKNTQGTVFKGAISYAVSKENHKCIYEGQGQIEHR

GSIKTGNFKLISEVYELATDKEIGFAYTFSGNVGGAHGVSTVKITNKDLILKLSICEEKK

QCTNAQVLASNDMVSENSNIQSLLILLDLRELGFPYELELQSKNTRDGFKFRYILDMKVM

SNSNLNYQLLITLQPSNGKVQLKLPTREMLLEMHQQYPQEGQLMGHYKSSLSLYMNKSKK

PNEVVRLLAQADVSGSDWVSLNLKSLLKFEHPAIQPCSISTRLEADRNKELVDLNIVFDV

FKLPEYQVVISSRLENIALPNGFNVTGQQILSSEGFGIQYRSAGHAAFNLENKELSLGGE

LTNVAGGVKSTILFLGSPAKIEILAYGLNEELLKLIISVKQIKPSTKIDGTLQILGTKPI

EMTSELQFGLAKLNVEREEFLHLDVFLAMGKELKLKAHEAGKELISVTVNLNPNSFLDST

FICNKKDIEEFVERLQHKYQEESNKVLLKMKEHYDTSYITSEQALVNKIRANLIDFKELR

PDFDEILKELEHDSTLKQLLEIYYEFEGKFLKFVEESLKLLHEISEKMTKTVLDMRSKLK

EMLHDIIIPAGQKTAEDILIIFKQIFNTFADVVMNSFDIFSKVFSEYEPMVKEYGKVVAE

LLKPSYEALEELYKFSLDILDDLPKNLQEHLTIIKDLQLELKRMFKKLHFEEKVFEFLNR

ILEELSLLPLNADILDLLRKLQEYTSAKLKNDPVNDKSYAEEFLHLLLKILRSSLVVDGF

TISTSTLPWDNIFFASPSSFNIFDDLPNLLTFRLSVVNFIFNENFDYLLSRDFWRSFIFF

EGFHLSGHLTEGRHLFTFDGVYVNLNGNCKYILTQDSLNNNFSVIAQVNNKLKSLYLTDK

EGQFLELNDAGVLKFNANPVEFPLHENGMHAWRLHYTIYLYSEYGVSVMCTASLKVCHIE

VNGFYKSKLRGLLGNGNAEPFDDFMQMDGTIAKNTVNFLHGYGLGKCNVASLTVNANDMP

HTDICNDYFGYESPLAIGYLIKDPSLYQTACDQAVASAADKDKETAACNIALTYASGIKK

KLDHPFIFLPERCLKCGGAPGQRDLFEDFTVKTPESSADIVFVIDVDVSAMQMTNLIAPI

IPEIRKALKVRGFSDIQIVVIAFSSGQRYPAILTSDQGKLNYHGNLANDKKKLKGPKPLF

SDFNISETVLAADKKTYILELLEKVVKNLVPNSDEMAFNLALDYPFRPGAAKTIVAVYSN

ELPYDNFLTVLRAHLSNLVIDFNGALLHVITTVKGMSLEGVEPEKLIGFNSRLVATLDGK

DAKKRQKLQYESSKSIDFVLNKGGWIFNMQHFEQLKPPDHIKVLNQVANSIADTLFKTEM

ISKCSCMPIYGIHSQHKCVVKSTHFVANKKVKS

>GMOY005519-PA chitinase|protein_coding|scf7180000648628:20365-26049:1|gene:GMOY005519

MKLLLILLGALLAVLTIKRTSAVQGSNHLICYYDGTSYTREGLAKLTLNDLEPALQFCTH

LVYGHAAINPSSNKLVSNNEKLDLDVGTGLYRTITGMKKKYPHLKVLLSVGGDKDEVDAD

NNKYLTLLESSNARIPFINSAHSMVKTYGFDGLELGWQFPKITEKVHGSIGKLWKGFKKI

FTGDFIVDEKAEEHKEEFTALVRELKNALRPDGYILGLAVLPNVNSSLFYDVPAIVNNLD

YVNLMAYDFQTPQRNPEMADFPAPIYELNERNPESNVNYQVQYWLQNHCPASKINVGIPS

YGRAWKMTTDSGLTGLPPVSDADGPAAGGLQTQTEGLLSWPEVCAKLPNPANQHLKGADS

PLRKVGDPTKRFGNYAYRSTDDKGENGIWVSYEDPDTAANKAAYVKTKGLGGVALVDLSF

DDFRGACTGDKYPILRAIKFKFQ

>GMOY005527-PA hypothetical protein|protein_coding|scf7180000648631:9230-15802:1|gene:GMOY005527

MSLAKLISGTQVANEIREALKQQVCKWKSVLPTFAPGLRIVQVGAREDSNVYIRMKIKAA

TEIGIDVRLVQLPRSITQIELLDKIFNLNNDPSVHGIIVQMPLDCETKIDSHKITDAVSP

DKDVDGLHTINEGRVSIGDLGGFMPCTPWGCIELIKRSGVKMEGANAVVLGRSKIVGTPA

ANLLKWYNATVTCRAADILVVGIGVPEMVKGSWIKPGAVVIDCGINVKPDPSKKTGTRLV

GDVDFEEAKLVASAITPVPGGVGPMTVAMLMQNTVRSAFTAAENLLQKAWDLAPLTLKLI

KPVPSDIVIARSQTPKDISLLAQEIGLIGNEVSQYGNKKAKISLSAIDRLRPRGNGSYVV

VCGITPTPLGEGKSTTLIGLVQALGAHLHRNAMACLRQPSQGPTFGIKGGAAGGGYAQVI

PMEDFNLHLTGDIHAVTAANNLLAAQLDTRIFHESTQEDKPLYERLVPKIKGERKFSKIQ

LRRLQRLGINKTDPDGLANEEITRFARLDIDPDTIMWERVVDVNDRYLRQITVGQSPTEK

GLSRPASFSISVASEIMAVLALASDLEDMKQRLGRMVVAFSRAGEPVTADDLGVTGALTV

LLKDALEPNLMQTLEGTPVLVHAGPFANIAHGCSSIVADEIALKLVGKEGFVCTEAGFGS

DIGMEKFCNIKCRSSGRYPNAMVLVATVRALKMHGGGAPVTPGAPLNKQYTEENLQLLEK

GIPNLLQHISNGHKFGMPVVVAINGFRKASLNAGAVDAVICTHWSEGGAGCLDLANAVIK

ACEKSNNFKLLYQNEMPLLDKLNTIAQNMYGAAKVELTPDAEKTMQKLTKAGFGNLPICM

SKTSASLTGDAKIKGAPKGFTLTVQNFYVSAGAGFVVAMCGEISKMPGLPTRPCIYDIDL

NTETGIIEGLF

>GMOY005582-PA hypothetical protein|protein_coding|scf7180000648667:20298-20823:-1|gene:GMOY005582

MAPRKAKAQKEEVQVSLGPQVRDGESRCFGVAPIYASFNDTFVHVTDLSGRETIARVTGG

MKVKADRDEASPYAAMLAAQDVAEKCKTLGITALHIKLRATGGNKTKTPGPGAQSALRAL

ARSSMKIGRIEDVTPIPSDSTRRKGGRRGRRL

>GMOY005584-PA hypothetical protein|protein_coding|scf7180000648667:63426-74155:1|gene:GMOY005584

MPFPREQLESVVNEKLCKTETIKLRNKYIGQACQLFYRSDPLKIVRGQGQYMYDEQGTRY

LDCINNVAHDDLIISTLKICDLIKEECSSNWRENIGAKPLKPLKLEMIRDTAANQLHDFK

NNALKKTESITTSSGNPVGIQDASMTVGPKGPILLQDAHFLNKLQSFQTERIPERVAYAK

GSGGFGYFEVTHDISKYCAAGLFSEVKRRTPIAVRFSTFSGETGSNDTVRDSKGFAVKFY

TDEGIFDIVGQNCPVFCIRDPLLFPSLVHVAKRNPQTHLRDADMYWDFMSHCPETIHYMC

MIFGDRGIPDGYRHMNGYSVHAYKLVNDKTEGIFAKFHFRTDQGVQNLDDERALCLACRD

PDYCTRDLFNSIRNGNYPSWTMFAQFLTQQQAKNLNFDAFDPTKIWPYTEAPLIPVGKII

LDRNPANYFAEIEQMAFSPANMVPGIEPSPDKILQGRLFAYGDTQRYRLGTNYLQIPVNC

PFRVPVKNYQRDGKMTVTDNQGGAPNYYPNTYSGPETCLRARTLSTCCPISGDIYRHSAS

AAEDNYSQATDFWVLVLDDCARKRLVQSLAIGHCHPKVVTAGSLQMATISTNNRFLHDEL

VQCAKTLTSKLPSPLSVCFFVNSGSEANDLALRLARNYTKRQDVITLDHAYHGHLTSVME

ISPYKFNQPGGDPKPDYVHVAPCPDVYGGLYKDKDYQCSDMAEVYSTPIRDLCERLKLQG

KGVAAFIAESLQSCGGQIIPPTGYFEKVFEAVRSAGGVCIMDEVQVGFGRVGSHYWGFQL

QDVIPDIVTVAKPMGNGHPVGAVVTTPEIANAFYNTGVSYFNTYGGNPVSCAIANAVMRV

IDEECLQENARLVGDYLLKQCRELKYEFDILGDVRGVGLFIGIELVKQRDSREPATKFAH

WVVNRMKEMHKILISSDGPNDNVVKLKPPMCFSQENADEFLLAFRECLSLLSKQREGDTL

PSSNAAAITTTSSSMELLSNKKQIFERRDHLIKSV

>GMOY005682-PA hypothetical protein|protein_coding|scf7180000648704:105016-108204:1|gene:GMOY005682

MAEVPKREVENIEFVFEVMGSPGEGIDAVDLGDALRSLNLNPTLAFIEKLGGTKRRHEKK

IKLEEFLPIYAQVKKEKEQGCYEDFIECLKLYDKQEDGTMLLAELQHALLSLGENLEDEQ

VEELFADCMDPEDDDGMIPYSQFVQRLMSDPIVFD

>GMOY005690-PA hypothetical protein|protein_coding|scf7180000648709:36749-38542:1|gene:GMOY005690

MTKKTRQRPKLSKPTRKQTEYDVKLNLLTDYIFNNLSKGKNEKYTKREYSYKDDLFNTEE

TSTVKKPETPLIDKKVDGKHHIATDPNIKMQIFVKTLTGKTITLEVEPSDTIENVKAKIQ

DKEGIPPDQQRLIFAGKQLEDGRTLSDYNIQKESTLHLVLRLRGGIIEPSLRILAQKYNC

DKMICRKCYARLHPRATNCRKKKCGHTNNLRPKKKLK

>GMOY005703-PA hypothetical protein|protein_coding|scf7180000648716:31353-60252:-1|gene:GMOY005703

MPRPIASQEDEDPTPYLFVSLEQRRIDQSKPYDAKKNCWVPDEKEGFLLGEIKATKGDLV

TVNLPGGELKDFKADKVEKTNPPKFEKIEDMADMTVLNTPCVLHNLRQRYYSKIIYSKDF

KKDLLTQVNPPKYEKAEDMSNLTYLNDASVLHNLRQRYYNKLIYTYSGLFCIAINPYKRY

PVYTNRCAKMYRGKRRNEVPPHIFAISDGAYVDMLTNHVNQSMLITGESGAGKTENTKKV

IAYFATVGASKKDESQKNKGSLEDQVVQTNPVLEAFGNAKTVRNDNSSRFGKFIRIHFGP

SGKLAGADIETYLLEKARVISQQSLERSYHIFYQIMSNSVAGVKEMCLLTDNIYDYHNVS

QGKVTVASIDDSEEFQLTDQAFDILGFTRDEKENVYRITAAVMHMGGMKFKQRGREEQAE

QDGEEEGGRVAKLFGCDTAELYKNLLKPRIKVGNEFVTQGRNVQQVTNSIGALCKGVFDR

LFKWLVKKCNETLDTKQKRQHFIGVLDIAGFEIFDYNGFEQLCINFTNEKLQQFFNHHMF

VLEQEEYKKEGINWDFIDFGMDLLACIDLIEKYNGFEQLCINFTNEKLQQFFNHHMFVLE

QEEYKKEGIEWDFIDFGMDLLACIDLIEKFNGFEQLCINFTNEKLQQFFNHHMFVLEQEE

YQREGIEWTFIDFGMDLQLCIDLIEKPMGILSILEEESMFPKATDQTFAEKLVNTHLGKS

APFQKPKPPKPGQQAAHFAIGHYAGVVAYNITGWLEKNKDPLNDTVVDQFKKSKNALLVE

IFADHPGQSGGGEQAKGGRGKKGGGFATVSSGYKEQLNSLMTTLHSTQPHFVRCIIPNEM

KQPGVVDAHLVMHQLTCNGVLEGIRICRKGFPNRMMYPDFKQRYMILAPAVMAAEKQPKK

AAEKCLESVGLDPDLYQILSPAAIKGVADHKKASAILLESTSLDPDMYRTGHTKARYFIS

VVSLRTPSSLSLISLLSFFLLILWNAKPKMGKSNLTAYVCLKRLIYQILNPKGILGQGDP

KKCSKILLESTALDQDQFRLGNTKACSIFLFYKIMCPKKLLGVDDPKKATQVIINYIDLP

DDQFRLGNTKVFFRAGVLGQMEEFRDERLGKIMSWMQAWARGYLARKGFKKLQEQRVALK

VVQRNLRKYLQLRTWPWYKLWQKRLEEKAKKAEEAHAAEVKVRKELEALNAKLLAEKTAL

LDSLSGEKGALQDYQERCAKLQAQKNDLENQLRDIQDRLTQEEDARNQLFQQKKKADQEI

SGLKKDIEDLELNVQKSEQDKATKDHQIRNLNDEIAHQDELINKLNKEKKMQGEANQKTG

EELQAAEDKINHLNKVKAKLEQTLDELEDSLEREKKMRGDVEKGKRKVEGDLKLTQEAVA

DLERNKKELEQTIQRKDKELSSITAKLDDEQVVVSKHQRQIKELQARIEELEEEVEAERQ

ARAKAEKQRADLARELEELGERLEEAGGATSAQIELNKKREAELSKLRRDLEEANIQHES

TLANLRKKHNDAVAEMAEQVDQLNKLKAKAEHDRQTCHNELNQTRSACDQLAREKFLNYF

IQQTATKWVSVMQRAYQIFYPLHNMAEKEKNEYYAQLNDLRSCVDHLTNEKAAQEKIAKQ

LQHTLNEVQSKLDETNRTLNDFDAAKKKLSIENSDLLRQLEEAESQVSQLSKIKISLTTQ

LEDTKRLADEEARERATLLGKFRNLEHDLDNLREQVEEEAEGKADLQRQLSKANAEAQLW

RSKYESDGVARSEELEEAKRKLQARLAEAEETIESLNQKCIGLEKTKQRLSTEVEDLQLE

VDRANAIANAAEKKQKAFDKIIGEWKLKVDDLAAELDASQKECRNYSTELFRLKGAYEEG

QEQLEAVRRENKNLADEVKDLLDQIGEGGRNIHEIEKARKRLEAEKDELQAALEEAEAAL

EQEENKVLRAQLELSQVRQEIDRRIQEKEEEFENTRKNHQRALDSMQASLEAEAKGKAEA

LRMKKKLEADINELEIALDHANKANAEAQKNIKRYQQQLKDLQTALEEEQRARDDAREQL

GISERRANALQNELEESRTLLEQADRGRRQAEQELADAHEQLNEVSAQNASIAAAKRKLE

SELQTLHSDLDELLNEAKNSEEKAKKAMVDAARLADELRAEQDHAQTQEKLRKALEQQIK

ELQVRLDEAEANALKGGKKAIQKLEQRCRELENELDGEQRRHADAQKNLRKSERRIKELS

FQSEEDRKNHERMQDLVDKLQQKIKTYKRQIEEAEEIAALNLAKFRKAQQELEEAEERAD

LAEQAISKFRAKGRGGSVGRGASPAPRAMSVRPQLDGMAFPPRFDLAPENEF

>GMOY005746-PA hypothetical protein|protein_coding|scf7180000648740:10421-11266:-1|gene:GMOY005746

MLVTTYQHDYIPPYTKRYEFLRTKPTAKSAEPEKEVCECVEETKVDFQKAVEKQCAMEWT

GVAPMGRLVDPRILPTKLSPEQLDAMAGEAQTDCLKEQPNRFLKILRTAYPDLYERLKQM

PKDELNRRLERERMYTTYQIDFCDMSEYPEGIYKSLKDEDETKKLNASKLLQRQGPCAEF

RTNVMQELVKEGEAGVVNQDPCEKAYKPFKISFADSARFISSGNNSHWRSNIFAKRADFS

EYMDTINKIGCVIMRNNIHNHQKCSNKHCRHQLTHTCANLK

>GMOY005752-PA hypothetical protein|protein_coding|scf7180000648742:46720-49171:-1|gene:GMOY005752

MANTNNASGDFNTEISDVVSVDRKKAGKEGKIRAIIFDSSNKKYTLEEVQKNDPQQPELS

EFSLNKTSSKSKPRFLRRSGQQKCGSCPDRLSNIKKSVQALDSDSTEVESSTDEEERWKD

VARASEIPADYYNIQKLVKFIKAGNQTATIVSLCCLRDYDLTTQINQFAIQDIGGLEVMV

NILECNDAKCRLGALSVLADISLNIDIRKTIVDLDGIPLIIDILNSSMKDLKTVAAETLA

NVAKVRLARKYVRVCGGIPKLVDLLDIKLQILKTPREELTAEDIENLNQARAGARALWSL

SDSRHNKELMRKSGIVPLMARLLKSCHIDVVIPIMGTIQKCASEPKFQLAITTEGMIADI

VNHLNSTNLDLKKEGSSAIYKCAFDKTTRDLVREAGGLEPLVAIIKDKNVRDNKPLLIGA

TGAIWMCAASPDNVRKFDSLRTVNHLVTLLNDECDDVLTNVVGAIAECVRVQPNREVLRN

ANGLPALVCLLNSSHPPLLENLAKALKECAQDIPSMRILEELDAVRLIWSLLKNTNSRVQ

AFAAYAICPCVQNANDSGELVRSLVGAMELVVGLLKSKDVLVLSAVCAAIATIAKDTTNV

AILTDLKVIYKLANLVNTTDDLLRANLAAAIASCATFGNNTQELGRLRTVTPIVTYMTSE

DPEVHRTTAMALEKLSMDPQNCITMHQSGVVPFLLECVGSTNKELQLAAAGCLRNIRELA

LRAEEYLLKIDDD

>GMOY005771-PA hypothetical protein|protein_coding|scf7180000648749:2746-3453:-1|gene:GMOY005771

MSYRTLTFFGILLVIQSHFVTPKPSPVDDNKFDTNNKLYKGTLAAFINFATQWGQRYSQV

LEKVIEDLKAEGKDHENVDEIEQLQEIAELSKHLKGDSDEETLHNIMDFDEEETDTDEIL

EQYADMDHVLALVYKDGKDVVKKFYRNLVKFVHSFSKALEEYTKDMSEEEKAEHQHFFEW

FKNFEELKQKNDVVAQSHMFLDFFKFFNPNI

>GMOY005867-PA hypothetical protein|protein_coding|scf7180000648777:19399-22124:-1|gene:GMOY005867

MSIRSLKAFNVQLCRHAYVLASKQVRILSYPMLMPAQSSQKREAHNSLFQMVKKRTIEAR

TKTQTNNFPNNKPVFKNESSGSNLAASESNPSCTISKSLSTSTKASPFLKPDSSKKPDDP

RKSGYASKPSYNAEILKAPNCLGVKKSGLAAKAGSDSDYCRIPLPQDRSPERPMVPIGQG

GCRPPKGQDPCEKSLTSETFRDGASKGSSGPDGGGGKDGTDNYWKILAAILTALGLGGLT

WWLLGRRPTSQVSEKGPTFLRSSDLPKHVPYLLIGGGTAGFSAFRAIKANDAKAKVLMIT

DEYRKPYMRPPLSKELWYSAGPSELTKDYRFKQWTGTERSLFFEPEEFFISPTKLMNNPN

GGIAVAHGFTVKKVDPSEKKVILNDGYEIFYDECLIATGCSPKNLPIFLDAPPPIKEKVM

LYRTPDDFEKLKRYVDAKKNVTIIGNGFIGSELACSLANYRKSAGSRIFQIFPESGNMSK

VLPDYLSKWTMQKVEAQGVCVLPNASVVDIKRDNSSLKLILSTGDTIITDVVCVCVGCEA

NINIAKSSGLEVDKIRGGFVVNAELEARRNLFVAGDASCFYDPLLGRRRVEHHDHSIVSG

RLAGENMVGKKKAYNHQSMFWSDLGPEIGYEGIGLVDSSLPTVGVFALPTKIDRRTDNLT

ESREKEVEKPLNEKDLPDVKCDIDNADSYGRGVIFYLKEDKIVGILLWNLFNRIGLARTI

INQNQIYDDLNEVAKLFEIHS

>GMOY005869-PA Trehalose-1-phosphate|protein_coding|scf7180000648777:32730-43271:-1|gene:GMOY005869

MAEQKKAPLLKKEEDYVKALEGFINPEADQVALLLDYDGTLAPLTEELSVMPKDTEINIK

KLAGNEKIFMCVFSGRELEAIKNHLKFPNVTYAGNHGLEVEYPSGKKFKIEMPEELLEKH

NKLVEELKEKVVCSGAWVEDKKISVTYHYKGVNDKLKAKLIETAKGLIQGHGFQLIETPY

ALEGKPRVNWDKGEGAKMILEKHFNADWAKNLKIIYCGDDTTDEDAMKMLHGIGKTFRVS

ELPTLKTYANYQIKTVEEQRMDTKLEMMDDYSSQSLEGDMSIPAGTSRTKATIGSPDFNT

LDEPIKETILRDIRAVGIKFSHVLYPKEKSTLLKDWDLWGPLVLCTLMATMLQGSSDREY

DGGPEFAQVFVIVWIGAAVVTLNSKLLGGKISFFQSVCVLGYCLTPVALALITCRLLLLS

QQTNLLFVLRLITTTIGFSWATYASFIFLGESQPPNRKPLAVYPMFLFFFIISWLVISHN

Q

>GMOY005874-PA hypothetical protein|protein_coding|scf7180000648778:26343-28072:-1|gene:GMOY005874

MTLLDLVHERNQLTMKLCIIFTVLAVAANITTALRAFAVIKNMLDCHERLGINEEDLMVI

QDLSDIKAASEYTPGQQCSIYCQSEAYGFTRRGQLKKWFMRKQPRIAQKYNLDKVFQNCK

RYATDTCDGPIHLAQCAQQYPLQAGDRNP

>GMOY005875-PA hypothetical protein|protein_coding|scf7180000648778:29937-30407:-1|gene:GMOY005875

MKLCIVVLIVLVAVNSALAATGIFAAVRDMLECHEQLRIKEEDLIFIDDLSQIQPVSSYR

PGQRCSIYCQSEAFGFTKQGRPMEEFWRNYAKIANRYDLDEVFRNCEYLAANTCEAPVLL

GICAQRFPRNL

>GMOY005876-PA hypothetical protein|protein_coding|scf7180000648778:37776-38246:1|gene:GMOY005876

MKVFIIVMVLMVIGSTSAFRIIHKTFTKLFECQKREQVPIEAWFAANLPDSDSTKLDPNH

QCGVYCQNEAFGLTTNGILNPEAILRFLPELEKTYNVEEMVKHCRHAGASNNCEGALKLS

ICFENYRLPETSLQ

>GMOY005915-PA |protein_coding|scf7180000648792:256383-264447:1|gene:GMOY005915

MKPLIKTTFLLFEKCMPEFVNAAYQLQIEATNTCGEQGDNHFCVQTMNSNHKNCEFCRWD

DHNPAFLTDLHDPQNPTSWQSETMYEGIQHPNHVNLTLHLGKSFDITYVRILFRSPRPES

FAIYKRTSETDLWIPYQYYSATCRDTYALPDSRAIRKGEGEAHALCTSEYSDISPLRDGE

IAFSTLEGRPSGIHFERSAELQEWVTATDIRITLDRLNTFGDELFGDAQVLKSYFYAISD

IAVGARCKCNGHASKCVASTGMNGERKLVCECRHNTDGPDCEKCLPLYNDVKWKRATSTE

VNECKRCNCNGFADKCFFDAHLFNLTGHGGHCLDCRENRDGPNCERCKENFYMREDNYCI

HCGCDPVGSRSLQCNSQGKCQCKPGVTGDKCDRCEMNYYQFGPHGCQPCGCDSRGSFENV

PSCDTETGICRCKDNVEGKRCNECKPGFFNLDLTNRFGCTPCFCYGHTSECQTAPGYSVV

SIASNFNKHKERWSAVDLYNRDMDIKYNQYSRSIGTTAQGNEYVYFQAPERFLGDQRASY

NRDLKFKLQLVGQVGPSTSANDVILVGAGTKISLPIFAQNNGMPDQNLKEYSFRLHEHRD

YQWQPSQSGRGFLSILSNLTAIKIRATYSVQGEAILDDVELQTAHRGAAGHPATWIEQCT

CPEGYLGQFCESCAPGYRHSPARGGPFMPCIPCDCNGHSDICDSETGRCICQHNTTGDNC

DQCARGYYGNALGGTPYDCKRCPCPNDGACMQINGDTVICTECPVGYFGARCEQCSDGFY

GDPTGLYGEVQTCKSCDCNGNVDPNAVGNCNRTSGECLKCIHNSAGKYCDECLPGHFGDP

LALPHGHCERCSCYPPGTEQNEEGVSQCDQITGQCHCKPNVIGRDCGECQPGYFNIMSGN

GCENCMCDPVGSYNSTCDRFTGQCFCKPGVVGLHCDQCAVYHYGFSSEGCKPCECDDSGS

KGFQCDQNGQCPCNDNVEGRRCDRCKENKYDRHMGCIDCPDCYNLVQDAANEHRTKLKSL

SSTLDEIARTPVTNDEEFEAKLKSVQEKVDILLSDAKYGAGDGGQTYVEVLNDLHKRLEG

ISTHLDSADSLQDNANEEIEKGRQNHTKAQDIIDAANNEIKNAMNMLNDEGALALAKARN

KSVEFGVQSNQISEISREARALADRLESEAQFDLKNAKDANDAVEKAYELAKSAINLQQK

VSDELRTEVRLELDTVKLSLGTTAQTTKEALRKANEVYDTALTLLADVNGLTAPDIDIKK

LKQEALEANEQADELLKRVNAISNNNGELFADFEDEYSLAEVILERADQQKLDDIKLLER

AKDAYDKATKAVEQGDNTLKEANNTYHTLAVNKTKVKYLKSNFIEQGFQSDVQLSSERAQ

FALKTVPIIEEEIEKAETLIRQAETALAGANKNANEAKQNAQEAQQKYAEQASKDAEHIR

KRANETKVNARKLRDEADQLNHRVKVTEIDISKLEDSSSKDDNLVDDAKRKVGQAKADAQ

EVKKQIDKAVNELDAIKEELTNLKDINTEDLNKLENRLNIAEAELSRANLNERIDKFREM

RNTQKKWIDKYEKEVQELEAEVLNVRLISEALPMGCFQRNRLEPY

>GMOY005924-PA hypothetical protein|protein_coding|scf7180000648792:461230-470557:1|gene:GMOY005924

MTKFINYYNLCPIPEPKDFLGIAADKEKGNIITTLSKNIVIIITISTQKQVRSWSVVEKL

SSKVVYDKRSEKYVGVFGNKTLRCWDESTADVNKSKKLRFHKNISELVTNEKDTFVLYDD

GSFESLKTAIDTRKDCKQITSVPQHLQLVPDFNLSKGRIFTLPGGKQILTYFETNHTSGE

CHLVRLPLEDGNLRQRFPLKRGNLQTTVCGAAVIEGDGVPSLCTIWSDKRIFLLTLDDCA

QPERSPGTFVSMLNHLKVDSSLSLLGLSGHSVAIYGANYGQKKESEGASLLLYNLQYKVI

KVKQFFKVYFDFSKLWSIDNHILLALGQNLSVVQYRVSKELLSELVGTQVCNDYQTSVEG

DQINEDDCIQDYLQYTTSIGKCTELEYVPKRTYHINEADGKSVAFIGVESFEKDLNHMRH

INLHVDVRRASAASSDDQITLMSNHNDDGFTCRKIQIIASQMEKAGASEHEITEKLLWLL

IKAELLSDIGVCLKRYTNISEKMLSKTLNFILKQFAITNPAIKDAASNETLILNGHRNSV

MDIDINSEDKSEKDKKNKKKLHIPCSDRLAKEEGTLQTEIKDILNTLLSCNFDFTAIGNY

IRQDIAYSEALILLEHLYNMLTDPDMTEFEERPSHDHTSVEFELQILRWFGVFLNTHFQK

LALSKDVKLIEILLRWQELFIQYKHEIIELQETSALLYNIIQCKKLAEDRDYSKCGITTQ

YPAASQARATTTLHIPGQGSIVGGYATTSWSEQAFMQFLGIPYAESPREELRFKLSVKRK

PWTHILNASNYGRRCPSLMSVNKIEEEELKEDLEDCLNLCVYTKDLNAQRPVMFYIFGGG

FFNGSNEDHPAGFLLEKDVVLVVPNYRIGALGFLSLGNEDIPGNVPIGDLILALEWVKDY

IKFFGGDAKQVTVFGQSAGAAMLGALLLSPKTPPGLFHRSIIQSGSILSSWGVRKDNIKQ

IKLLCNTLKCDECTSNADIYKCVRNVSVAKLLEETKTACFGPVLGDYYGFMPHEPLELYK

DKTISVPLMTGFTKHDGSFVLAIYYDGWKKELPNLSNVTVRQFANGLFNMINDNGGIANN

LLMKLLFPKDLWRSDQHQKALAAYFDVANIAFMKSPTITFAQKMLEKHSAPVYLYSFDYD

GLNTRFGYELGHEHYPFSGGVHHSNENIYLFPTHLLNENDKKIAHKMVDLWTSFVINGRP

QTDGGPNILPMEGKF

>GMOY005926-PA Superoxide Dismutase 1|protein_coding|scf7180000648792:481967-484484:1|gene:GMOY005926

MPAKAVCVINGDAKGTVFFEQNDECAPVKVTGEINGLSKGLHGFHVHEFGDNTNGCTSAG

AHFNPCNKEHGAPTDNERHIGDLGNVESNGSGPTKVNISDSLISLFGEHSILGRTLVVHA

DQDDLGKGGHELSKSTGNAGARIGCGVIGIAKV

>GMOY006016-PA serine proteinase inhibitor|protein_coding|scf7180000648810:4903-12106:1|gene:GMOY006016

MRLLAVLVLSAIAVVCSGKKPPEFCKFTHAANTDDPSICDGGGQSLWSYVLEENSCVEFY

YYGCYGNNNRFFTKSQCEYICKKLCNFQHSANSEDPSFCDGGGQNLWSYVPQANSCVEFY

YYGCYGNENRFFTKAQCEETCKKKPGFRKKFCGPLSIQQSCTSLMYHKQIVKMVM

>GMOY006034-PA mitochondrial ADP/ATP carrier protein|protein_coding|scf7180000648818:59426-64290:-1|gene:GMOY006034

MRNVSIGTDISQNLIMGKDLTDPVSFLKDFLAGGISAAVAKTAVAPIERVKLLLQVQEVS

KQIPEAERYKGIIDCFVRLPKEQGFTSYWRGNLANVIRYFPTQALNFAFKDKYKQIFLGG

VNKHEQFWRFFIGNLASGGAAGATSLLIVYPLDFARTRLAADVGKGQDRQFHGLGDCIVK

VFKSDGFVGLYRGFNVSVQGIIIYRATYFGFYDTCRDFLPNPKETPFYISWAIAQVVTTI

AGITSYPFDTVRRRMMMQSGLKKEEMIYKNTMHCWYTIHKTEGLKAFFKGAFSNILRGTG

AALVLAFYDEIQKYI

>GMOY006145-PA hypothetical protein|protein_coding|scf7180000648863:10127-67228:1|gene:GMOY006145

MPMWDGEDENQLNGTGSTPKQRLLNWIHTKIPDLPINNFTNDWTTGKAVGALVDACAPGL

CPDWEVWDPKDAVQNASEAMGLADDWLNVRQLIKPEELVNPNVDEQSMMTYLSQYPNAKL

KAGAPLRPKTNPNRVRAYGPGIEPVGPVVGAPANFTVETFSAGKGLVDVEIVSPDGLIEK

ADVRFNNDKNLTYSVSYIPKLEGEHKVFVKFSGRDIPKSPFYVKVEGHAGDASKVKVTGP

GIQPMGVVLSKPTFFDILTKDAGRGVPEVIIIDPANHKTSVAAKVRQIENDVWRCEYVSA

LTGLHSVNVFYAGTPVPGSPFPVKIAPLSDVRKVKASGRGLQPNGVRVGDDADFKIYTEG

AGEGIPEVRIVGPGGMNENVVLTKLDAHTYECHYFPMKEGRYVAVVTFAGQEINKSPFEV

KVGPKKDSNIVAFGPGLRGGVVGYPAAFVVETNGETGALGFTVAGPSQAEIECHDNGDGS

ALVKYHPTATGEYSVHILCDEEDIPKSPYIAQILPRTDYHPEHVKAYGPGLEKNGVTLGE

PTHFTVDTSKAGSAPLDIIVEDNHGDRIPVEIKPQPDNTYKCTYTPTSNVPHTVEVNYGG

VAASNSPHRVNVGVPVDANKVQSFGPWLQPGVRPNVATHFNVDCRDAGEAELKVKIVHDE

TKVELPCRVIDNEDQTYTVEVIPPSKGAYTTTMTYGDQRVPIGQKVLVESQIDVSKIKVD

GLEQTAPLNSLQQFRVITNGLPKADLTVAITSPSGSRIKAHVIPTAEGFLVNFTPTQLGE

YLLSICFGGTPITPRPFRLQCLGVGDSTKVRAFGPGLERGLVGQPAEFMIDTRGAGQGGL

GVTVEGPCEAAINCRDNGDGTCNVAYLPTEPGDYTVNITFNDCHIMGSPFQPFILPLPNL

HNTRVSGIGIQPHGVVMSKPTDFMVDMSKVGSDIDAKKLSCSIFDPKGQVLPSEIVAGLS

NDVFRIMYTPFEAGRHTIELLYDNIPVPGSPFVVNVNSACDPTRCKAYGPGLNRGFVNEK

NKFSVETKGAGKGGLSVAIEGPSEAKMTCVDNRDGSCDVEYLPTEVGEYDVSIRFADKHI

PGSPFSVVVEERSDPTKVKVYGPGIEHGEVRENVPTHFYIDVSEAGPGRIAVKIQNSEGK

PLDNLRVEEKSDGLYCVHYSPPKEGSVLTSTITFSDIEVPCSPFIMSVFPKAEPAKVKLK

GVNEKKKTPASKPTEFEVDTKKAGEADIDVNIKNPKGKHIVPRLHETDTGTYVVSFTPDE

CGTYYVTVKYGGKEIGGSPFKLEAVAIGEAKKCKFVEQAPKIQPSGSKSHLTVDARDAGD

GAVTCKITSKAGKEIVDIDVIEKDGFFDVFYALNDPGDYDINVKFGGKDIPNGSFSIKAV

ESIEEYSAYFEEHTKVIKSTTQTTEHLVNGRSETTYRAVAFEKFPVPTTGGIVVEAPPAT

WEDFAYFYRSKFYHFTVSDRDHVEKTQFYYNEAFINENEGLNGCDFLPLNFKTAFSILTH

QDNNNKEYFFLKFLAEVKMPSGKVDKPVIQDNRDGTVSVKYEPKEEGIHELVVKYNGEPV

QGSPFKFHVDSITSGYVTAYGPGLTHGVTGEPCNFTISTKGASAAGLTMAVEGPSKVDIN

YHDNKDGTVSVQYLPTAPGEYHISVRFGDKHIKGSPYIAKVTGEGRKRNQISVGSCSEVT

MPGVITDDDLRALNASIQAPSGLEEPCFLKRMPTNNIGISFTPREIGEHMVSVKRMGKHI

PNSPFKVTVMEREVGDAKKVKVSGVGLKEGKTHAENVFSVDTRNAGYGGLSVSIEGPSKA

EIQCADKDDGTLNISYKPTEPGYYIVNLKFADHHVEGSPFTVKVTGEGSNRKREKIQRER

DAVPVTEIGSKCKLTFKMPGITSFDLAARVTSPSNVTEDAEIQEVEDGLYSVHFIPKELG

VHTVSVRYKDMHIPGSPFQFTVGPLHDFGSHLVKAGGSGLERGVVGLPCEFNVWTREAGG

GSLAISVEGPSKAEIEFKDRKDGSCDVSYKVTEPGEYRVGLKFNDRHIPDSPFKVYISPD

AGDAHKLEVQQFPEGNIQADAPYQFMVRKNGAKGELDAKIVAPSGTDDDCFIQAIDAEMY

SIRFYPRENGIHAIHVKFNGVHIPGSPFRIKVGKDVADPAAVSASGSGLETVKTGHKADF

IINTCNAGVGTLSVTIDGPSKVAMDCTEVEEGYKVRYTPLLPGDHYVSVKYNNMHIVGSP

FRVSCEGDKLADVGAQETSTVVVDTVAKFSKGGKNAGIHMPTFKSDATQVTSKGMGLKKA

FIGKQNTFTVNATDAGNNILYVGMYGPKGPCEEFTIKHTGRNNYNVSYSVRDRGQYILIV

KWGDENIPGSNISALVEYVDINADD

>GMOY006173-PA hypothetical protein|protein_coding|scf7180000648874:157338-167023:1|gene:GMOY006173

MSTVDKEELVQKAKLAEQSERYDDMAQAMKSVTETGVELSNEERNLLSVAYKNVVGARRS

SWRVISSIEQKTEASARKQQLAREYRERVEKELREICYEVLGLLDKYLIPKASNPESKVF

YLKMKGDYYRYLAEVATGDARNTVVDDSQTAYQDAFEISKGKMQPTHPIRLGLALNFSVF

YYEILNSPDKACQLAKQAFDDAIAELDTLNEDSYKDSTLIMQLLRDNLTLWTSDTQGDGD

EPQEGGDN

>GMOY006294-PA gamma-glutamyl phosphate reductase|protein_coding|scf7180000648948:31720-79429:-1|gene:GMOY006294

MNVFLRKSLQLTNAVKSSFSTNVWRAASIQAPRQPASTPEKRQTERKPTFSERSQLKYAR

RLVVKLGSAVITREDNHGLALGRLASIVEQVAECHLEGREVMMVTSGAVAFGKQKLAQEL

LMSLSMHPRAAAAVGQSGLMSLYDAMFAQYGVKIAQVLVTKPDFYNEETRTNLFATLSEL

ISLNIVPIINTNDAVSPPMFLKDDDIPPGTKKGIPIKDNDSLSAMLSAEVQADLLILMSD

VDGIYNKPPWEDGAKLMHTYTTNDSRSIQFGQKSKVGTGGMDSKVKAATWALDRGVSVVI

CNGMQEKAIKTIIGGRKVGTFFTESTDGFTTPVEELAESAREGSRQLQTLTPEQRASAVH

TLADLLVSKEQFILEANAKDLSDAQRNGLAKPLLSRLSLSTGKLKNLSVGLKQIADSSHK

NVGRVLRRTRLAENLDLTQVTVPIGVLLVIFESRPDSLPQVASLAIASGNGLLLKGGKEA

ANSNKALMELVKESLTTVGAEKAVSLVSTREEISDLLSMDDHIDLIIPRGSSDLVRSIQQ

QSLHIPVLGHAEGVCHVFIDKDADIQKALKIARDAKCDYPAACNAMETLLIHEDLMSGDI

FNDVCNMLKREGVKIYSGPKLNQLLTFGPPAAKSLKHEYGALECCIEIVKDLEDAVNHIH

TYGSGHTEVIVTENDTAAQHFLKSVDSACVFHNASSRFADGFRFGLGAEVGISTARIHAR

GPVGVEGLLTTKWILRGRDHTAADFADGGGSVWLHESLPV

>GMOY006295-PA succinyl coenzyme A synthetase flavoprotein subunit|protein_coding|scf7180000648948:77192-79180:1|gene:GMOY006295

MHRVAILRKRVAKKFTIKDLLFNSVINNGLYSARRYNSNSTKSVDVAEIRKKYMIVDYSF

DAIVVGAGGAGLRAAIGLAANGFKTAVICKQYPTRAHTVAAQGGINAALGNMEEDHWEWH

FYDTVKGSDWLGDQDAIHFMCREAPKAIYELENFGLPFSRTPEGTIYQRAFGGATLNCGK

GGPAHRCCSAKDATGHAMLHCLYGQSLAYKNLQFYAEHFVFELLFEDCKCVGILAWNLES

GNLCRFRAASTVIATGGTGRMYFSTTDGHCCTGDGTAMVARKGLPLQDMEFVQFHPTGMY

GTGCLITEGVRAEGGFFTNDKGERFMEKYAPKLKDLASRDVVARAMSLEIISGNGCGPEK

DHIHLQLHHLPKDLIKARLPGIQATALIFANVDVTKEPIPVLPTVHYNMGGIPTNYKGQV

LTLSDEGEDEIVPGLYACGECSCASVHGANRLGANSLLDIIVFGKACADCIASCNTPDKP

IPPLDEKTGMRGVVYLDNLRNSDGKTNPAELRLKLQRAMTKHAGVFRDGKLLTEGIEIVK

GISKEFNDIKVTDKGMIWNSDLMEIIELQNLIINALLTITSMEHRKESRGAHAREDFKTR

IDEYDYSKDIKEQTKKPLDEHWRKHILIWYEMNGKTSFKYRPIIDQVLDNSTNSIPPAPR

TY

>GMOY006316-PA hypothetical protein|protein_coding|scf7180000648956:15289-18142:-1|gene:GMOY006316

MFKSERPSKQNLNHSELLFVSEDNMKLLSNQVLSKLLARNVYSRISTNLCAASIIRRDFS

NAKSEKKQKLLSYPALKLDKTIEKFLKSCEPLLNRDEMEKTKCLAEKFVSEDGKKLQTLL

EEAARKEENWLSHRWLKAAYMQYRDPVSVWSSPGMTFPMKLFLKDNEWLTYASKIIMGMI

KYKRKVDCGEIPVVKMGKHELDNSQFKLVYGTCRIPHPKEDKMDYNPDSNYVVIIHKHHF

YKLPVYDKSGEILNADVLEGQLIRIISSEKERGIPYGVLTTNKRDDWADAYAELLKSPKN

AETIKTIQKSLFTVSLDACVTPYKNTILADRALQLIHGGNVCRNGGNRWMDKTIQLIVNP

NGMSGFCYEHSPAEGQPVALIAEYLSKIVSKEDEFKTGSSKDFECFTKLEFDTPTDCTVA

QIGEASSNLNELVNNFQLHVTQFKDYGKGLLKQYKLSPDSFIQMALQYAFYRLHKTPAAQ

YETAHLRIFYGGRTETIRSCSNESVAFAKAMLDGNMEDEQRAEALRKAVDSHRKYATMAL

MGEGIDRHLLGLKLMAFENKMPIPEFYNSPGYIKSMHFRVSTSQVASKDDAYMCYGPMFD

DGYSCCYNPREDDIFFGVGALHSNKETCAVGFSKSIEEALLQMKKVLGPPPKSDADKKAS

PGKPDANKKPKSKL

>GMOY006365-PA hypothetical protein|protein_coding|scf7180000648980:21290-56669:1|gene:GMOY006365

MFWEIISDEHGIDPNGMYHGENDMQLERIDVYYTEANHGKYVPRAVLIDLEPGTMDSVRQ

SPMGYLFRPDNFVFGQSGAGNNWAKGHYTEGAELVDQILDVIRKESEGCDCLQGFQLAHS

LGGGTGSGLGTLLISKIREEYPDRIMNTFSVVPSPKVSDTVVEPYNATLSIHQLVENTDE

TFCIDNEALYDICFRTLKLASPTYGDLNHLVSVTMSGVTTCLRFPGQLNADLRKLAVNMV

PFPRLHFFMPGFAPLTAKGSQQYRALTVAELTQQMFDAKNMMTACDPRHGRYLTVAAIFR

GPMSMKEVDTQMFNVQSKNSSYFVEWIPNNVKVAVCDIPPRGLKMSATFIGNTTAIQEIF

KRISEQFTAMFRRKAFLHWFTGEGMDEMEFTEAESNMNDLISEYQQYQEATADDDVEFDE

EQEEREAYEAEIQNGGI

>GMOY006372-PA hypothetical protein|protein_coding|scf7180000648980:83288-86883:-1|gene:GMOY006372

MKYVLMCMLLLFAAFHQIEGEEVNDEFPTENIDLDLGDFKEGSRTDSETVQREEEAIKLD

SLNVAQMKELRDKAEKFAFQTEVNRMMKLIINSLYRNKEIFLRELISNASDAIDKIRLLA

LTNKDEIETNPELNIRIKADKENKVLHIMDTGIGMTHEDLINNLGTIAKSGTAEFLSKMQ

DTTKSEGQDLNDMIGQFGVGFYSAFLVADRVVVTTKNNADKQYIWQSDANDFSIIEDPRG

DSLKRGTIVSLHLKEEAQDFLEEDTLRELIRKYSQFINFPITLWSSKTIEEEVDEDEGDG

DEVQPKEKLSTSEEDSADEGEDEAKVEEDTPDRKIKKKKVTKTEWDWSLINDSKPIWIRK

PSEVTEDEYKDFYKTLTKDSKEPLTHTHFIAEGEVTFKSLLYIPTVQPSESFNRYGTKSD

NIKLFVRRVFITDEFNDMMPNYLNFIRGIVDSDDLPLNVSRETLQQHKLIKVIKKKLVRK

VLDMIKKINEKEYENFWKEYSTNIKLGVVEDPSNRARLAKLLRFHSSNGKSMTSLAEYVE

RMKPKQEHIYYLAGANRAEVEKSPFVERLLAKGYEILYLVEAIDEYCISSLPEFDGKKFQ

NIAKEGFTLSDSEKNKDKFEKLKSTFEPLLKWLSEDALKNKILRAEVSERLSNSPCALVA

GLFGWTGNMERLAMSNAHQKSDDPQRTYYLNQKKTLEINPRHPLMRELLRRVEADEADDE

AKEMALMMFRTATLRSGYMLQETSAFADSIEKMMRQTLGISLHEQIEMDEDDEVEAEAGE

TAAADANVADEDDTAEHDEHDEL

>GMOY006406-PA hypothetical protein|protein_coding|scf7180000648998:777-1232:-1|gene:GMOY006406

MFAVKSIILMTLLALALAEVIDFKTCDTNDCDIHEVRVEPCPQAESHAACNIRRRKPANM

SFDFTPRFDADTLEASLNWVKSNDQELPLISMEKDACKYTKCPVKSGEKQTYQVDIPIES

KFPLNYYTIKWNFQAPNGKHCCFTHDIKLVR

>GMOY006412-PA hypothetical protein|protein_coding|scf7180000649011:10403-11098:-1|gene:GMOY006412

MNIIKDFLIYFLILFIINDCVSQSLNTPERERNAIKYFYNYVDIITDFKLNNMLKMTQTF

VEQLLDAIPYDDRGNTAELLQQYIDKAENLRYHGVSIEEKENMLLELQQLIATIRSGLAK

QEAEDIILKKSMLGMFELLARLSIEERRHSEKLSKASSLLRRRFTPEGIQRHQQLFDLLH

ELEQAQDIVNKEALFKHLKELRTQEI

>GMOY006418-PA odorant binding protein 21|protein_coding|scf7180000649017:13397-14825:1|gene:GMOY006418

MIRAEDEDWQPKTVADIKSIRNECLKEHPLSNEQITKMKNFEFPDEEEVRQYLLCTALKM

EVFCAHQGYHPNRIAKQFKMDMNEEEVLEIAEKCHDSNPDNSSVDVWAFRGHKCMMSSAI

GDKVKAYIKKRQEENAAKNA

>GMOY006519-PA hypothetical protein|protein_coding|scf7180000649084:8597-9273:1|gene:GMOY006519

MLSARISSNMKLLTASCVILLAVVVCLAEVQGPPRAKRSYGEPGRPGPYGGGGGGGGGPY

GGRGGDGGGGGGPYGGRGGDGGFGGSGSGQPGQPGQPGGWGGDGGNGGGGGSYGGRGGQG

GYGGGQPGQPGQPGGGNGGGGGGNGGGGGGNGGGGGGNGGGGGGNGGGGGGNGGGGGGNG

GQGGGAGQPVQPGQPGYGYY

>GMOY006600-PA hypothetical protein|protein_coding|scf7180000649181:50069-69459:1|gene:GMOY006600

MSAKAIREATGKDILNRHLNRHGAPFTPCRFATVNMQTDWSDLVAQQQWLSNTPLVVKPD

QLIKRRGKLGLIGVNKKLAEVKEWINARMNKDQKIGQAVGKLRNFIIEPFVPHTDAEEMY

VCIYSHRSADTILFYHQGGVDIGDVDAKALKLDIPVNSDVTMADINNKLLSQVPGQKKER

VAKFIYALYKAFVDLYFTYLEINPLVVTDDNIYILDLAAKLDSTADFICRPKWGDIDYPP

PFGRDAYPEEAYIADLDAKSGASLKLTILNRNGRIWTMVAGGGASVIYSDTICDLGGADE

LANYGEYSGAPSEQQTYEYARTILNLMTSSPKHPDGKVLITGGGIANFTNVAATFRGIIT

ALREFQPKLMEHNVSIFVRRAGPNYQEGLRKMREFGSTLGIPLHVFGPETHMTAICGMAL

RKRPIPQALNAEFSTANFLLPGGQNAQPELRTLVGDCSNGNEAQASKGLDSSSQVQQQQQ

QSIKLPPILADNIKSNDVHDNVANGTRKFFTNKTKAIVWGMQQRAVQSMLDFDFICRRDE

PSVVAMVYPFTGDHKQKFYWGHKEILIPVYKNMSDAVTRHKEVDVMVNFASLRSAYESTM

EVLDFPQIRTVAIIAEGIPENMTRKMILAANKKGVAIIGPATVGGVKPGCFKIGNTGGML

DNILHSKLYRPGSVAYVSRSGGMSNELNNIVSKATDGVLEGIAIGGDRYPGTTFMDHIMR

YQADPEAKLIVLLGEVGGTEEYDVCAALKDGRITKPLVAWCIGTCAGMFTSEVQFGHAGS

CANSDRETATAKNKALRDAGAYVPDSFDTLGELIQHVYAELVKTGRITPREEVPPPTVPM

DYSWARELGLIRKPASFMTSICDERGQELLYAGMPISEVLNKDVGIGGVISLLWFQRCLP

PYVCKFFEMCLMVTADHGPAVSGAHNTIVCARAGKDLVSSVVSGLLTIGDRFGGALDGAA

RQFSEAYDTSLHPMDFVNQMRKKGQLIMGIGHRVKSINNPDVRVKIIKEFVMDNFPACPL

LRYALEVEKITTSKKPNLILNVDGVIATSFVDMLRNCGSFTSEEAQEYINIGAINSLFVL

GRSIGFIGHYMDQKRLKQGLYLASSMCGFANEAESSNNFKHETHTQHIKTAQSQTTTEND

RSGDLSLELHESLRFEPPQLDFGAWAVGMVRSHTVTLVNQNLNRSVYLSSITGRTPAFYT

SFFEVKMIPPNSNTTFSVVFLPREQGAISTDLHIHTSFGVIGLWVRGEGRACPYRLKPII

GIKAPLNATLVPEIHMYNPHTNALQILEIYSSGGQFQLELPSGDQEAPQGLWEIPPHTTK

PIIRIRFQGLAAGNYSAYIRIKIAGPLLGEEHKDILVIPVEFEITSDYDLYAENPLLDLG

HVTVSSETKPIQRHIYIRHSKHEFRLNSLQCSNEQINKGFEYNSKNSTLTIKPQMLTHIA

DGASVSERLVLKLVTPSSGVAVQHYVQLLIRFELFKGSFHYDREINRLTLKDFINGQRRF

VFQNLFQQPLTVYNVSLHKEQSDTTLTIEMPTLRVDGSILLPHATFEVDFKLSKRDSKDL

NYKTTLHIYTNLTRFEIPVSLCSGHLHVYTQTNTIWRSSSSVYSTELNLGSKPQTSFVIA

QNRNPISVKITNWRMQSVRAVRCSIMFLGCVKASSATSLDGHTIVDLVNTNYTFDDNLDE

GDVAVFSVELHPHARSLLELSEYPINQMMVISTQYENITVNIKLFISNGRLEVDQEKLHF

ISCFPGKLCSSELSIRSTYKYPLHIRSINFTEPGFRFEDFQQYGSKISPEAVTTVGRIYF

EPALLCGSRCYISPYTDEVVTFLNNFEGNSHHIPHFDEVELRRRTELYRHFKWYFQNIDF

IMTTKDSVQFQMNLIIELEWPQLVGGAAVLPTTEVNKTQEYVVTITNPADTPILVDYFLA

DPSLAKQTQLSLPLEVIVIAPSCYLTDKAVFSLVNNPPEKPLLIPAGASVSVPVRFRSQT

AGTYCTLLHVRNNLTLYEAVWLSAKTVQSQFRLSNRKPGSLNPLLFEISELQMESVCSAT

QNPSKVGVDVAHKLLAKRVFTARNSGELPIWIKGIFIDDQPCTGYGFSVADCSPFELKTN

GTRRIEIAFQPDFTVTRITKPLILKTNHSFEFDYVLVAQLPNKGLERCIALLPRPFWESR

IRSAAIVVLSIMFALVLIAAHIDYGNICYQQSKLSLARDKGQMQPFNLRNIAMRSQQSTE

MSGIEAVTGKTKEKPNVSDGNGNTGLKKRNTNKNQKSFSNGETKSSITKRNLSLADMVNW

TFGSGESRKRLAKDKTETLLTLNGSNNSNGSNNNSSSNENTAKNLKPKLSEKTLLDKMDG

GLKKLTSCPKISNKKNKIFEATATEDLIKLSKTEEEVHEKESKKEKNSPKTNAKASIKAS

KTCMGALTPPNGKDLNVCAGRKLGKTPGRERRKNCSMEVGSSLHSSSSASSSSCSSSTCS

ASSMHSQHSSSHKARADRKAKLKASNSLNFSTNATNISTPSTDSIEKAEKQKESSTALND

CLYVGNCITSPWETNSQVTFSDVLQSQSSKSATTKHTFLDDLMPAPVVSALQSSPLENSE

LLATLTTSQTPAQKSELGPIGSRKSPSSTPVWETFHNGNALGSVSSYFPDLLSHTLGNPY

GFDDHSQQQQQHQRQEQQQSQQTNLLTNHLYEIHAQDELPCQWQQTSDYLRKLREQQMLG

HNNQQQQQQQQFLESTACSTVAKLPRNNWSQLNCTSWPSMVNSNAVVPATSTNAVNFDPA

NQNAHSWNLAATTTTTSNMIRPPPGLERHYILNQKQQMSTATMTTSGAVAINGSVNDLST

EATENIPIFDPFGLSSIWTDNWKQSLGQTQQKQQPQERQVSGPSQQQQYCYPSATYAHKS

TIIGGISQGKKRENLKTLRIANKRDKEQKSLNE

>GMOY006845-PA hypothetical protein|protein_coding|scf7180000649364:2012-2332:-1|gene:GMOY006845

MKVIFSIVLLTAFVFLAGSQVMAEDAKQAAPAVGEQKAVEAPPTETSDVAAPATTLAEFA

DTTTNATEPQKTKEEKPADNSTKTSRR

>GMOY006913-PA hypothetical protein|protein_coding|scf7180000649563:34445-57977:-1|gene:GMOY006913

MAANNMLRNCLERRKDVERKRNSVFLSICDREFGQCVDPTAPKCGKCCLFSLDAPVSDAD

KMLVHIYKKKTNKCKFLVGCTDMPIKGLFDKVMENFNVENPNWEETTKKHISNIPHPLEP

KPSQIVDNDCDDDSVGRREQMCPTSEMNKRLLPIFNLKGAQTGNVVLIIRLVANGPAIVS

YFPFARICQAGCGAKPPICGPKDRTKSCPDNDKDKCAGVDDILPQIGRCCGGNDSAFGAS

GTSNIDATSSAKEVASKEGSSKQDDTGSAALTYGNKSACGGRKVYRNFKDPDNGCIPLED

PCKKQEKKLRCLRYFACNADKGCPCDNVEDDCERGKQKHQACKPPKCGCPESIKDSCDDS

KGKDVSKSQTYSCDKCTDECETASGDERFDPCKVPDTSRREPCLMEPTKPSPGPSDYEEF

EACLNGSGLLIRVLKDTHQVQNICDGTENFDNAESGSDCDRNQHDSQNSQCNLNELLQRS

DFARNQIKRRTGGHIVNHPKLPKIRANIKYSGNDYCCPDSYHVPFSRFKEFCDNQENKVD

AYRRRKAGDRGVRPPLHPDENRSCCVQVNRDDIKNAVQGVNVDTRKKGIEVCYRTCEETD

SIQHTRITKHHFAMSKWYRPITNTGDNFVVSFWYNFSKILRKTITATIHISRNRNPNETI

VAKNNTDSQGRL

>GMOY006927-PA hypothetical protein|protein_coding|scf7180000649583:342931-343498:-1|gene:GMOY006927

MLKSLKNYQVFFLHFLYVQAIWAEVSEEEDGELSQPIEKVWDRTQLQAEIEAINKKLQAA

EQEIVESMESVRNVFNKVQEWAIRSQANVVNYAVTVCKSQVATTTEEASITTARVGPGTL

GFIPESTTVSVAEQHSMLDIVERLKAKLKEAYETALNEVEQMAN

>GMOY006928-PA hypothetical protein|protein_coding|scf7180000649583:355956-356592:-1|gene:GMOY006928

MLETVLSLNLRFVCCLFIFVEGAFGAHGTLSNHFTHDAEFVNNTSIDKLTMPVHAKIHVI

ESKKKTRVSSTKSNGVEVIRNDRSESSLRQNSDNNLEEEFNKSSKNFVVKVDNETVENLQ

DTSTTRSTERKAFDASNLFPGKKPAGDIQVKGDQLNPSLKQQTFSPPSTPSYNPRNIKQL

LTKQLYIGKLGL

>GMOY006948-PA hypothetical protein|protein_coding|scf7180000649694:2-4965:-1|gene:GMOY006948

MALVTSSLVQTPQSVRVVVIGSGPSGIAAATRLVEAGFNDVLLLEAESRVGGRIYTIPFA

DSVIDLGAQWCHGEKNNAVYEMLQRLNLLNLLETTGDTYFKFKCVRSNKDIVPDNVVSKL

TEIFYQIIPSREDELKSYEGSTGAIMTEMFWKEIDKLPEKPDRTVVKEYFETAKKSNLSL

EAADTMFEVSPSNSLQFEISEGDQNLNWKDKGYQTFLNLLLKTDDYKTNLGLLEDKIQVC

QRVKHIEWRHEDGIKITLFSGKIIVADHVVCTTSLGVLKESHKSLFQPSLPLSKIRAIEG

LELGTVNKFFLEFEKPFEPVDWVGFNFLWLEEDLMALRETERFWLENVFGFYRVAHQPRL

MQGWIVGRHARYMETLSEKEVLEALMWLLRKFFSRPVPEPIKFTRTQWYTNCNFRGTYSL

RTMYTDELRTGAWDLAAPLLDDDGKPIVQFAGEATNSHHFGTVHGATESGWREADRLIHF

YQKKSFTNLTIPCFETFNNKYETTMALVTSSLVQTPQSVRVVVIGSGPSGIAAATRLVEA

GFNDVLLLEAESRVGGRIYTIPFADSVIDLGAQWCHGEKNNAVYEMLQRLNLLNLLETTG

DTYFKFKCVRSNKDIVPDNVVSKLTEIFYQIIPSREDELKSYEGSAVHKGKRFLSPGNQH

ELEPVLKAPYSLLLYPSKTSVPLETTKQQIISGSVTPLGPPGTFTLVLIDGTWPQAKAIY

ASSPLLHRMRQVKLIAAGCSDYIIRTQPTEGCLSTLETAAQSLALLEDRPELRELLVKP

>GMOY006970-PA hypothetical protein|protein_coding|scf7180000649751:60406-70895:1|gene:GMOY006970

MARFIDLQLANYGLTGIMRISQLTNDLTFDDTFDIDQFEDVPASGTKLRSARDLNEQDLI

EDELQPVEAIESEDQNGGSWLMQSVKRVRRELGRLFGSDNKSQDIEKTKSKQHRRRSHGE

RLAGEKKNVNIPDGELKAKKQPKKLQKITGKHSKRQSNGDDNFEGSGDDDDLYETDQWQT

LFTLNEPWLDEYREGPSSPQYQNLGHQVETAFMEVISDMYGDDVDYSVKVKFVRVIPTSD

HYKIHCIVQLELPKKMKDFGDRLRDQIAKYRRIGQNLSAEVDEQFYFRTDVTDHEYVDNV

TPDGYEHGNNGFKYDCDQDGFFTCGDGQEIPCIYRCDNKFDCEDETDEAKEMCDTIFNED

ELSAEDERHRGNEDEHRERHSGEEGNDGGYTENRHSEEDQRYPVDDGRNSGREHQYPAGG

SDRSENEPRYSEGDRRYPESGHYPESDRRYPEEDSDHRGSDSRYPENEHYPEGNHRYPEN

ERYPEGDHRHSEGEQYPGGDHRYHENEHYPDGDHHYPENEHYPEGDHRYPEGEHYPGGDR

RYPEGEHYPGGDHSYPEREHYPDGDHRHPDGNVPYSGSDSRYPDIDPRYGSHHPDSTSSE

SDHGHPLGPHTDVSTSEEFNCLVNPNGEFLCQDGRILPCYLRCDGQPDCQDHSDEADEEC

SRTETDTDNGFDASTEGDIHIGGDNDFDGSGEGEVEPRHFNVTETSPVYTSPQHGGGCRG

DATYTCRHSGYVICDEHVCDGTEHCLDGEDEENCGSIEDNTSYEKVCAANEFKCDDRCLP

KEYQCNGVIECYDRTDEEHCP

>GMOY006971-PA hypothetical protein|protein_coding|scf7180000649756:2332-3365:1|gene:GMOY006971

MGRTQPNEMDAVVATAIDVGFRHIDTAPLHKNEKEIGAALNKQIQEKKVERDELFIVSKL

WNTFHEPDDVLCGCKKSLENLGLDYFDMYLMHTPMGFKPSDDLFPIKKGKVSFSNADYVC

TWQVMEDLVKQGLCRNIGISNFNTRQIGRLLKHATIIPQTHQIEAHPYLMQTELISFCKY

NDICITAYAPLGSPARPWAGKDTEDVLLKNSTVLDIARRHEKTPAQILLRYQIQLGNAAI

PKSATCQNMANNFDIFDFELHPEDMQNLDSLNYGMRLFKFSG

>GMOY006985-PA hypothetical protein|protein_coding|scf7180000649829:12331-20365:1|gene:GMOY006985

MIVQKVLKKSKLISLKMCDPCCAPCCSPCGPCGPCGPCSPCGPCSPCDPCCAPFECSPKC

YTGAQLDAVPQCAPRIAPPHPKCLTVQQPPRLICKKRVVFTEKVVPEPMVVNRCRQITIP

KVVDTTRVIKVPKLTWVSQMVREPRVIYYPSMVPDPYVVCYPKRICEPREVCQSILCQPK

PQTIDIPPPREYCCYPTGPINYKPSAACPPCPMGPSSPCGPCGPLPCFSTNQYPLCEPCG

PCGPCGPCGPCGPCGPCGPCGPCGPCGPCGPCGPCGPCGPCGPCGPCGPCGPCLAPNCGP

CGMTMPSGPYVPTPCGPCGPCGPCGPCGPCGTCEPSGPCGPCGPCGPCGPCGPCGPCGPC

RPCFPGCPYEAPECGPCYPCSPTPWNTHFVVDHLLKDNVIVALNDGFKPLAGIGIIKVSF

KREFTLISAPYQGGFHLETIHLGIPERQSWPDTNRSIPLFHRKLINSFLLFLQPKACRSR

ELRCGIMYTTCDCIKRNGLQDKCPRSLCQGRSACMCFPTPNCCPSSFPLRYANMTMGVIK

KKNKNNRRAGGGNGNGNGNGGCCSNGNCCSSNGCGCTCNPCGSSSDPAYCGLSNPPCGAP

APIPCSPTPRCCPPLPEGGIPDPRVWNSCNTPQVYPSHPFTGVAVFPIANKLNGLQETIC

CSTIDDEDYRRALIPFSNVEPKRPIFSTGNPCNNYPPLTDPLSCLCDDRLQQPNCLDGFQ

TTYSNNCCPPGKCNYIFLMNCNCGPIGCPNFSQYPQPSDDFVPLLDQERRNGLQKHFCDY

NKDEDVKEYERALIPFPDDGKINKRRPIFSACDNSLLQDPWWGPCCDNTQISANMDNMDC

FIPQTSVLCRDTGDKAGILDPCNGYPCLQEPCYDPCFGNYRMPCMDHCYGGFQKPSSCNS

LHQNMSCTQRNDKCMTLECNLQRSNFETGPTIAQADAIIMETKANLDEKLSDFTMYHEQV

SDSTMYHEQVAVSNVYREDVSSSNTYNENIIESNMYGDRNASVSNAYPNNR

>GMOY007011-PA putative metal-binding protein|protein_coding|scf7180000649882:73212-74590:1|gene:GMOY007011

MFSLSSKIPSTCGKLFVSRFSNSFISIARKNMGSKAESLSGNDCGNNSTPTIGTHSGTFH

CDEALACFMLKQLPEYENAEILRSRINEELRKKCSIIVDVGNVFDHERKLYDHHQPSFQE

TLSSLRPELGDKYKIRLSSAGLIFNYYGERVIDCILKKHGIDLSDENLRLTFIQVYQKFI

KEIDAIDNGLPMFLCPEGQEPLYTIGTDVSARVGRINLSWDDETGDCQDDRFREAMCFVG

NEFVEEVLYTGGSWIKARECVRTALQNAAKVYETGEILLLERALPWKEHLFDLEEECKVE

GRSKLVIFEDPLDNSWRVAGVPVTPQSFLGRQFLPIEWRGLRNDDLFQAAGVKDLLFVHN

NGFIGGAKNKEAALAMAIKSVQWPRKNETKEKQC

>GMOY007027-PA hypothetical protein|protein_coding|scf7180000649963:11264-13333:-1|gene:GMOY007027

MADEAEAPPPPPEGEPAPEGEPKPEGEPKPEGEAGAAAPAAAKEGEKPAVDMDDGEDYSP

LGSADDLEKQRNYKDYKRLVKEIKCQNETIERVKQQIQDIACKKCVTSCEKKDLKCLQEC

LEQELEKLRCLINKAMHLQNFGSRRRLKNVCGGGAGKVPGGSKSGGNADESELALLRNRV

SDYEEEQREFKCLLKEQSCQLEDYRDKYLGAQQKVEEQKATIDKLNMNNKTVEKQINAEV

KQIRAKFQEKLCELLQYPKLLENEQLKLAETCKEKEELEDKLVIVCKELKALKKKQTETG

GTDNCQSQLRESQKELEKVNKNLEEVQRQRDLFCEQLRSTEEDLNTLRCESAKIIARTKE

RAEVMREQMQNRIDRLEKDLAQCRAAACMSVNDRETVINEMRGQINTLSYSFDAAQKQIK

TLRNHIAYMSNENCFPIKC

>GMOY007028-PA hypothetical protein|protein_coding|scf7180000649963:17947-18798:-1|gene:GMOY007028

MGASRQGIGDGSNAEESELILLRTRVNEFKEEQIEFKCLMKEQAEQLEDYRNKYISAQQK

VEEQNALIEKLNMNNKRVEKQINMELKAIRAKFQEKLNELLQYPRLLENEQLKLSEACKQ

KEDMENKLMVVCKELKLVKAKLDNTAQDCRPQLQKCRLDLENATRNYEDMQRQRDLFCEQ

LKVTREDLDTLRSESAKIIARTKERFEVIKEQMQERMDRLEKDLAQCRATACLSVTDREV

VIREMQGQLNTLSYSFDAAQKQIKTLRNHIAYMSNENCFPVKC

>GMOY007029-PA hypothetical protein|protein_coding|scf7180000649963:18846-20124:-1|gene:GMOY007029

MADAPGTDDENERVAGTEGTAGTVSAPDTEAEPPSNLGEGTQDEGGEQEQKDEYTPLGSS

DDLEKQRNYNEYKRLVVEIKCQNEIIERVKTQIQDTACKPCITICEQKELRCLQECLEQE

MHKLRCLINKAMHLQNFGSRRHYKEIQLVTTFDEDQMAPISYYCDTMQVKTSKKSTAKQL

QSAGHWKSVVSSDTSEEEKPCSCQENPGSQDTSCTPEEDERKLMRDVLEAIRLCKKKSHE

ESSFEKLKNKIFGMQETVEELKEEICRREIQRKLSGPCPLRKKTRDVAPQPRSTSHIPSR

TPCPTAFPTPCPAPCPCADPCPNTCTNICQFPGHGPCHDDCHMVCEKTTLPPLEPCRSHK

PRKEDLFQKLQDNYVYLLTEFTKKDGQLKELNKKQVYFGKKKFAK

>GMOY007044-PA hypothetical protein|protein_coding|scf7180000649969:79227-87411:-1|gene:GMOY007044

MLCRTLLTSRYLRTRIYPSAVSSYNSSSNGNGTPNQRLLYKTTIPVNEQQQNHQTADTIN

ETLTADNNQTLASDAIAGLPSKHECSSCRNAVSHTNTLLMTNENHTTATKQTATTATTPP

PPPPTTTTTTTTTTVEYELSSNGIAAKLCKVSTSHIAPKQSSFSNLYSHTQPNCTVGQQT

LSSDEEILEKTDNKDYQIPLLSSSDLKVLCENLNAINTTNNRSLRSSFKAPALSYQQRAN

YTMDCNGINMDALRLAPRDRLGLWGTHNDLDIPGNISGLGRLRNKKYNKGLAFTITERQI

LGIHGLLPYVVKTDEEQVKHCTILLDRLENDLDKFMYLSDLAERNERLFYKLLASDIAKM

MPLVYTPTVGLACQKYSLIYTNPKGLYISIKDKGHVYEVLRNWPEADVRAIVVTDGERIL

GLGDLGANGMGIPVGKLALYTALAGIKPHQCLPITLDVGTNNETLLNDPLYIGMRGKRIT

GQKYDEFLDEFMQAVVRRYGQNCLIQFEDFANANAFRLLDKYRDQYCTFNDDIQGTASVA

VAGLLASLKLKNTKLTGNKILFFGAGEAALGIASLCVMTLMKEGLSEVEAKKHIWMVDSK

GLIVKNRPAGGLTEHKEHFAQEHPSVNTLLDAVKTVKPSILIGASAVGGAFTTEILELMA

EYNETPIIFALSNPTSKAECTAEQAYFSTKGTCIFASGSPFSPVEYNGKKFHPGQGNNAY

IFPGIALGVICCGMLTIPKEIFLIAAEKLADLCDENDLQKGSLYPPLEKITPCSVEIAKY

IMQYAYQKGLATVTPEPKNVIEFIINQMYKLDYPSALPEIYAWNNTKE

>GMOY007078-PA hypothetical protein|protein_coding|scf7180000650090:17283-22251:-1|gene:GMOY007078

MCANRAIGIRADLRYRGNAVHPDYPGQCYYEDLQQPIPVSQSFKPINRDGRCESIYCRND

FVLEIGICPRHNMQETDECSIVSDLTKAYPDCCPKAMLEGGGAATTEPVCSYVNSQGERV

FLKYFPLSKKGEDYVDFDSSGKCLKRAVCNEKYETKVENCAEYTVNCENKSHYKGVFPAC

CTKC

>GMOY007085-PA actin 5C|protein_coding|scf7180000650090:80493-84134:-1|gene:GMOY007085

MSENRRRIIIKRVYDKVTKNKERNTEKFFEDIVLQLLFKQLYNNYINEHRPKKMCDEEVA

ALVVDNGSGMCKAGFAGDDAPRAVFPSIVGRPRHQGVMVGMGQKDSYVGDEAQSKRGILT

LKYPIEHGIVTNWDDMEKIWHHTFYNELRVAPEEHPVLLTEAPLNPKANREKMTQIMFET

FNTPAMYVAIQAVLSLYASGRTTGIVLDSGDGVSHTVPIYEGYALPHAILRLDLAGRDLT

DYLMKILTERGYSFTTTAEREIVRDIKEKLCYVALDFEQEMATAASSSSLEKSYELPDGQ

VITIGNERFRCPEALFQPSFLGMEACGIHETTYNSIMKCDVDIRKDLYANTVLSGGTTMY

PGIADRMQKEITALAPSTMKIKIIAPPERKYSVWIGGSILASLSTFQQMWISKQEYDESG

PSIVHRKCF

>GMOY007176-PA hypothetical protein|protein_coding|scf7180000650230:62292-63347:1|gene:GMOY007176

MSCFINVKWVAKRSFLESFQKLLVASRRSRTSDCYAKTIPNLILNKETKVICQGFTGKTA

TFHCKAALEYGTKVMGGVSPKKAGQVHLGLPVFKTVKEAKDATQPHATIIYVPPPSAAKA

IFEAIEAEIPLIVVITENIPQHDMIRVQNCLMGQDKSRLVGPNCPGVIAPEQCKIGIMPG

HIHKKGLVGIVSRSGTLTYEAVNQTTFYGLGQTLCIGIGGDPFNGTNYIDCLEIFLADPE

CKGIVLIGEIGGDAEEKAAEFLIENNSGECRKPVVSFIAGLTAPPGRRMGHAGAIISGGK

GGANDKIKALEAADVVVTLNPTTIGKTLYKEMVFMEIIPDTMKDKLKPKSS

>GMOY007181-PA hypothetical protein|protein_coding|scf7180000650233:48296-49285:-1|gene:GMOY007181

MYYQVFGNVFYAYHLSLLEFSSNQLSDCLVFKMFNLKFIILAVILVARYSSAIPIPDDDD

FKLELVDKDMTQRIRQLIKGEYDKDDLSAKDKYMLFVINESKKLYEFFRDETTKVSEALL

ADTELTENEDEEVKEFLKKIQEYLDKSKDADNVEEKLTAVGFFFKLMDNYDAPNTKDPSK

AVVLIDGYLNKHGMEKFSEEFEKLSNDFLKEFIEKSEELKKDLTEEELEKYALFIEIIDE

LKKDLTIESRLDLLFDKVAVEKNHE

>GMOY007187-PA hypothetical protein|protein_coding|scf7180000650233:100222-139820:-1|gene:GMOY007187

MLMRASQFAKSITMACSVQKQTSSSAGPATPKSKAFGIPRGSLPTDMAQPPLEFQWPPPT

PASTHTNNVRLHMMNQDPKDLHTARAMIEELRSKVRCQAEHIMKWRKTYALQVQQHYRYQ

KEKNDQLNALTCQLLLLESRLKRKQKQIASLLNHREMTIQRQQKIIDTLSTRLVDHGLET

IEASYATELDSLNDSDSAVVLEDIDSDSNVTLTGNRRRSSGNIGNNMTSDGITIVRSISD

AIETNLTKYGVTRRNNCFLRRPEILETVYSVEEDPEPTSDVAEKRDKFKQRSEKALSSSS

TEGQIDNAEVNTSPSTPTPTTVNSAATVANGAHITTKEGDKKEDKPSICAIKNGHMKLPQ

LHNVKARSMEDPARASSIDDDDVNGNNLPKSNQVTNYNRVMSNHRSVTKPKDVKYKRINK

AKSKSLEELRGRLKNLVEKVDHQTSAAVHLPGPINASTGLSSELEFNSSKTKHAAPKRSL

TLPRVLVPQAALHNGGSGTTGTGNGSSPLPSSVNAQKFNTKNKLYKGTVAAFVNFATQWG

KRYGKVLDRVIEDLKAEGKENAGGIEHLKEIAELSKHLRAGSDDETLRNIMNFDGNEADL

KEIIDSFADPEHVVTLAYKDGKDTVQEFYITMLKFFDAFGKALEEFTRDMSEKEKAEHKE

FLEWFKNRKFSVKAITRIVSKMNYVTLSFVGMLLVLQLCFAIPKPDADSPASHHEFNTDN

KLYKATLEQFVKVAIPLSEQYGHLLEKVVEDLKAHEEAPNYETQIEHLEALVKACKHLKG

ESDDETFHNILKLQSDVAGAQEESKKSSKHHLVRDLFDKDGAKNLIEEYHKDFTEFFDGF

DDAFEEYAKDLSEEEKADHEDFLKWFKEFKEADGFDKKFEKFVEFFKFFNSENVLEIRNN

KLIITAFAMRSSTLVVLGLLLIIQHSSLSPVPEVDATDAQNELDRNSELYKNVASDFVKR

ALPYCENFRKISEQMLKDIEEHKTDSQYNDVKDRLEEFIEATDHLGNADQEEVLKYMLRL

VEHLNRKQNELYNIKFSWNAPRAPDADSPASHHEFNTDNKLYKATLEQFVKVAIPLGEQY

GDVLEKVLEDLKAHEEAANYETQIEHLQELIKGAKHLKGDSDDETLRNILKLESDLAGAQ

AASKKASDPHLVHDLFEKDGGKEYLEKFRKSMGEFFDGFDDAFEEYAKDLSEEEKADHED

FLKWFKEFKEADDFPYQFENVKFAVKTIIRIVSKMNYLTLTFLGMLLLLQTCFAIPRPDA

ESPASHYEFNTDDKLYKNTIKTLVEIAIPLSARGIVLLQRVLEDLKAHEEAPKFAHQIEH

LKNLIKSIKHIKADSDEKILRNIVGLKNDLAGAQKPSKESSNPHLVRDLVKKDGGKELAG

DLRKHLVQFFDGFVDAIEEYAKGMSEEQKDHHKDFLKWFKEFKNAHSYGKRLDTIVSKMS

YVTLTFIGIFLMLQSCFAIPRPDVESSASHHDFNTDNTLYKPTLRGLVKLGEQCGDVLEK

VVDDLKAHEEAPKYEHQIEHLRKLIEGFKHLKVHCDEETLHNILKLKDDISVKVIVKVVS

KMTCLKLIFLGMLLLFQSCFSIDKAAAEFNRDNKLYKATVEALIRFATPLREQYVTLLEE

VVEDLKANEEASKYEQQIEHMEQLIKDFQDIKADTDEETLRNILKLKDGVIDAGEPSKDW

SNVTVISELFKQDGGKELAEELRNDFAEFIVGFNHSFKEYVEELSEEQKADHEEFLKWVK

ELQETDGFDNQVKNTIFAVKAIIRIVSKMSYVTLTFFGMLLVLQSCFASHHEYNTDNKFY

KATTKALVKVAIPLSERYSEVLEKVVEDLKAHEEAPKYEHQIEHLEKLIKGFKHIKADSD

EEILQNIIGLKNDGAGAQEPSNESSNPHLVRDLFEKDGGKELAEEIRKDFVEFFDGFDDA

FEEYAKELSEEEKAEHEDFLKWFKEFKEADAFEKKLDKFFEFFNESVKRKKVNKHKMKAF

VIVVLVAQIILATEVLEKELNENNQFYKNTLKEYLEHSYTFAERFAGMCEKVLADLKQHD

EGSEFQSQKAELEDVLHYVAAMKKDENEKTLENMLKLHEILLSAAKEFKETHQAKQTLIH

QLFEKYGAKVIVHDFRKGFVEYLKNFETNFVEYEESLSAEQLESHGKLIQWFKEFKEEQS

FAKKFTSFVTFFKFFAPDLLKNEQKRSDEISNICENIFEMKLYILASLLLISSGQRSLAV

SVEAFTSQHEFNENNEFYKKSVNDFVKLNQPIAESFANILDKVVDDLQDLTQVDPYSATI

NSLAYSEMSKKLRNYQKNEDKANLETALDICGLFVTYIDVDKVAAKYSSATPVPESKTDL

KELDTEEKFVQHIKNKFNRDELTPKEILAVFLIREYKKFFNFFYAESVSLSEAILADPEI

LHNDLPAVKEFAKNLTDYLEKTKNVNDIEDELNLLILFGNYTDMYETKELEEPTKLDYLI

EGYLNKHGMEKFSEESKQRLDRAIIEFFVEFDKFKATLDEKDLEKYKKFIEFVNELKTKD

SIESRLECYLNFFTTDDDDDNEDKN

>GMOY007193-PA hypothetical protein|protein_coding|scf7180000650239:69648-71895:-1|gene:GMOY007193

MQCTLRNIINCSILKSPLRSNGSILGSLNGRNYSSEHEADLVVIGSGPGGYVASIKAAQL

GMKTVNVEKDPTLGGTCLNVGCIPSKALLNNSHYYHMAHSGDLANRGIVCGGVELDLGKL

MAQKSNAVKALTGGIAQLFKKNKVTQLSGLGTITSANEVQVKNKDGGVDTVKTKNILIAT

GSEVTPFPGIEIDEEVIVSSTGALSLKQVPQKMVVIGAGVIGLELGSVWSRLGAEVTAVE

FMDTIGGVGIDGEVSKTFQKILTKQGLKFKTGTKVLGASRSGNNVTVQVENVKTNEKEEL

TCDALLVSVGRRPYTDGLGLEAVNIVKDEKGRIPVNANFQTVVPNIYAIGDVIQGPMLAH

KAEDEGIICVEGMKGGPVHIDYNCVPSVVYTHPEVAWVGKSEETLKQEGVAYKVGKFPFL

ANSRAKTNNETDGFIKVLADKTTDRILGTHMIGPVVGELINEAVLAIEYGAAAEDVARVC

HAHPTCSEALREANVAAAFGKPINF

>GMOY007243-PA hypothetical protein|protein_coding|scf7180000650259:233335-234380:-1|gene:GMOY007243

MLANYISRSLQSVRNGVRAIATSTVQNSDNLFVHRDTPEDNPDVPFEFTAENQKRVDAIL

SIYPEGHKRGALIPLLDLAQRQYGWLPISAMHKVAEILGLPNMRVYEVATFYTMFMRKPT

GKYHIQVCTTTPCWLRGSDDILATCKKTLGIGVGETTKDMKFTISEVECLGACVNAPMIA

INDDYYEDLTSKDMEEILADCKAERVPRAGPRNGRFASEPKGNPTSLTEEPKGPGFGLQP

GL

>GMOY007256-PA phosphoribosylamidoimidazole-succinocarboxamide synthase|protein_coding|scf7180000650265:2705-5920:1|gene:GMOY007256

MTSIQEYKLGKILIEGKTKQVYDLPQNPGLCLLLSKDRITAGDGVKAHDLQGKSEISNTT

NGQVFQILNEAGVRTAYVKSAGPKAFIAKKCQMIPIEWVTRRLATGSYLKRNTGVPEGYR

FSPPKQETFFKDDANHDPQWSDEQIISAKFNLNGVLIGQDEVDIMKKTTVLVFEILEKAW

STRNCALIDMKIEFGIDEEGEIVLADIIDSDSWRLWPAGDKRLMVDKQVYRNLTSVTSAD

LDVVKSNFVWVSKQLQDIIPKSDHLVVILMGSASDLDHCEKIAKQCKALGLNTELRVTSA

HKGPEETLRIMREYESVMNNLILITVAGRSNGLGPVLSGSTTYPVINCPPLKAETMPIDV

WSSLNLPSGLGCATVLYPEAAALNAAQILGLSNYMIWAKLRVKQLNNFVTLKKADKQVRG

VRKA

>GMOY007295-PA hypothetical protein|protein_coding|scf7180000650279:376910-379007:1|gene:GMOY007295

MHVCDGLGVHLQFDPVPVDLALNEPPAKQRRFESNNKGRGGGAAKRAEASVGSGNSATYV

EDMYNAWKRDPGSVHASWNAFFASGAYECVPPEHTSQRNLVPLSQLCGHNPRSEVIEGHL

NAQSVVRSYQTRGFLTADLDPLGITQSKKITTPCGMELRATEAVLMMVSNFVKGEYLNI

>GMOY007296-PA hypothetical protein|protein_coding|scf7180000650279:379068-404022:1|gene:GMOY007296

MDCSFKLPATTYIGGKEEELTLREILKRLEKIYCGTMGIEYMYIYSDGKRDWIRQRLEKP

GCMEITKDKKLLIFKRLLRATRFEAFLARKWAAEKRFGLEGCDVMIPCLKEIIDVSSNLG

VESFVIGLAHRGRLNTLANVCRKPLDQIFSQFHSLDAQDLGSGDVKYHLGTFTERLNRVT

NKNVRLCLVANPSHLECVNPVAQGKARSEQFYRGDSEGRKTMCILIHGDASFSGQGIVYE

SMHLSDLPSYTVHGIVHIVINNQIGFTTDPRFSRSSPNCTDVARVVNAPIFHVNADDPEA

SVHLSKICAEWRAAWRQDVVLDLVGYRRNGHNEADEPMFTQPLMYTKIRAHKPVFDIYCE

KLLNEKVITKELLKQSIDEYDAILEKGFEDAKKITTMKYSDWLDSPWTGFFQSRDNSKVP

CTGVDEKILCHIGYKFSSPPPPEQNFVLHKGIERILAARKQLVDDRTADWALGEALAMGS

LLREGVHVRLSGEDVERGTFSHRHHVLHHQTIDKVTYCAMKCLYPDQALYSVCNSSLSEM

GILGFEHGYSMTNPNALVIWETQFGDFYNTAQAIVDTFISSGEMKWVRQSGLVIMMPHGL

EGQGPEHSSGRVERFLQMSDDDPDTLPEADCDDSNVPLKQLRTINWIVANCTTPANWFHI

MRRQVALPFRKPLVLLTPKTGLRHPLARSNFCDMMEGTEFQRIIPDYGPASQSPNDVCKL

VFCSGKVYFDLFKARAERRQECKVALVRVEQLCPFPYDLVKEQILFYRESELIWAQEEHK

NQGAWFYVQPRFKTAMTPDYDDGFIINYVGRPCAAAPATGNKNQFQRELNSLITDVFGEL

TEEDKEFIKATEEAARAKAQEAAKADAAKAKAQQAAKADAAKAEAQETAKADAAKAKPEE

GVKADAAKAKPEEGVKADAPKATPQEGVKVDAAKAKPQEVSKTDAAKAKPQEPLPKPPNE

GVPPPATPAGSSPMKTPTPQKESTSPGGGKSNVVLQIFNKYPEAGCSRVLILAEKKSIDK

IMFGLLISGRLPQTDFTPVDENKLLINVPDIDHVNYIVVFLTGVQPLPEGMSAAVYFSWP

DANSAPTWQYLGHISNAKPSAIFKISQLKKAPELEAQENAMVFGAQEISHIAQIGISVEP

DIAISQLTPAVSNANANLQFSQKMLENFYNYVSSFIVNVSNETLVPLSTLQNWYTNFQRR

MEQNPNFWKY

>GMOY007314-PA odorant binding protein 17|protein_coding|scf7180000650289:23868-24284:1|gene:GMOY007314

MKSWILVLLTVGAVTIIDGSNDKAAADNKVILLYHKRACLEMEGLSEEVFPGDDVHEIFA

TMFQLESEVVPYETKCFLRCWLKRIQVMGDHLTMLKKKMNPDGTCERAARAASRGDECEF

AFLYQKCDHLLDVNEFDY

>GMOY007353-PA glutamate dehydrogenase (NAD(P)+)|protein_coding|scf7180000650305:30603-33421:-1|gene:GMOY007353

MTFKCACVDVPFGGAKAGLKINPKQYSEHELEKITRRFTLELAKKGFIGPGVDVPAPDMG

TGEREMSWIADTYAKTIGHLDINAHACVTGKPINQGGIHGRVSATGRGVFHGLENFINEA

NYMSMIGTTPGWGGKTFIVQGFGNVGLHTCRYLTRAGATCVGIIEHDGSIYSQEGIDPKL

LEDWKNEHGTIVGYPNAKPYEGENLMFEPCDIFIPAAVEKVITNENAHKIQAKIIGEAAN

GPTTPAADKILIERNILVIPDLYINAGGVTVSFFEWLKNLNHVSYGRLTFKYERESNYHL

LESVQESLERRFGRVGGRIPVTPSESFQKRISGASEKDIVHSGLDYTMERSARAIMKTAM

KYNLGLDLRTAAYVNSIEKIFTTYRDAGLAF

>GMOY007369-PA hypothetical secreted conserved protein|protein_coding|scf7180000650326:202-17000:1|gene:GMOY007369

MNGPPQQLFKPDDYGTTPLNLAICVRLLISNKFTIITEKNPTLELQLIMTKGRVNIAFLL

IFHCILIALITCTEAQQNAMEPTMLGTDLLLGGNVDPEKVRLVTVKNADGSEFEILVGRN

TKKGRAKGTTPLNLAICVRLLISNKFTIITEKNPTLELQLIMTKGRVNIAFLLIFHCILI

PLTTCNEAQQDATTTSTTMTNLSSFTANIGKESIKALRPTILGTELRFGRNLNPDKVRVV

TVKSDDGSDVEILVGRDSRKGRAHPSETSFFVRKTDVARHPEKRTAEMKANASTLQFQHS

PVLLKQLELARQAKEYNQRKADADERLKKLQELKLIKAESFQEQQKQLLEPKKSRAARRI

HFETAANQFSPQHFSEEIYFTSPPETRRVERRQPTYIPLDRNNNGWQFPASELSNGKQLR

SWRWKPIESRNFQPQYRQEDAQQFYPSINRYNQGFPTDPQKKLEVSDQHNARHTRYIDSA

TRNSFIEDLTNNKYLRQNLITKNNFIPAQYKSLRLPEPIVITSSTVVKSDAVDNVGKAIN

SPNEYSDSNSFTRMTLAKTSNVMPKTSPSESKVADTGSIVPVIEGIRVPDTPEDELKTWR

NARVLNNQLVPYPVGYTPAKVEMQTFDR

>GMOY007382-PA reticulon|protein_coding|scf7180000650341:2361-13685:-1|gene:GMOY007382

MAGGRKFSRNNSSSNQQLPVRGPVESLIYWRDVKKSGIVFGAGLITLLAISCCSVISVFA

YLSLAILSGTIAFRIYKSVMQAIQKTSDGHPFKEYLDYDLTLSQDKVQHIAGVAVAHVNG

FVAEMRRLFLVEDLVDSIKFGVILCVMTYIGAWFNGMTLVIMAFVSLFTLPKVYENNKQS

IDTYLDLARSKVADVTEKIKAAIPLGTKKPIAAESDKDK

>GMOY007443-PA hypothetical protein|protein_coding|scf7180000650392:22142-24500:1|gene:GMOY007443

MSSLRLTNWLTRSLLRYSKVQQISRIQKRFYAAVDDPRVTRKNENMVYMSFKIILKYTCL

REKKEYVFARKILNTAINGLLKGLILGVYQKEGEKDPKLTPSGEKFDDRVQGKVSELVKE

CNITGTLGKGKVFNNIDQEYRSVAVIGVGREGAGFNELEMIDDGMENVRIATGVGARSLQ

LQGCSEVFVDSMEYPEQAAEGSALAVWRYNDNKQKKNRTNVPKLELYDSPEVDAWTRGLF

KAEAQNLARRLSDAPANQMTPTTFAQATVDALCPCGVTVEIRTMDWIEQQHLNSFLTIAK

GSCEPPVLLEISYCGTAPEDKPILLLGKGMTFNSGGICLRPADGMEEYRGAMAGAAVVVA

AIRASAALSLPINISAVIPLCENMPSGMAVKPGDIVTLINGRTLAIRNTDKAGVVVMADP

LLYAQNTYKPRLVVDVATLGNGVVKGLGGGATGIFSNSHYIWKQFQKAGSLTGDRMWRLP

LWRYYRQLVADNVSFDISNDGVGPASSCLAAAEFVPCVDWAHLDIRGVGMLTRYGTVPYL

LKNRMTGRPTRTLIQFLYQMACPDATPSK

>GMOY007466-PA hypothetical protein|protein_coding|scf7180000650411:128623-130101:-1|gene:GMOY007466

MFKRLNLNNLQVIVATARRNSSDKNVGEEKFRIETDTMGEVKVACNKYYGAQTVRSKKNF

PIGGKSERMPKPIVIAMGILKKAAAEVNKEFGLKAEISDAISKAADEVISGELYDKGHFP

LVIWQTGSGTQTNMNCNEVISNRAIEIMGGEMGSKNPVHPNDHVNKGQSSNDTFPAAVHI

SVGMELNKSLIPSLTELAQGLGDKEKEFADIIKIGRTHLMDAVPLTLGQEFSGYRQQLCN

GLARINACLPRVYELALGGTAVGTGLNTRKGFAEKAAARISELTSLPFETAPNFFEALAA

RDAMVEVHGVLNTIAVSLMKIANDIRFLGSGPRCGLGELMLPENEPGSSIMPGKVNPTQC

ESLTMICAQVMGNHVAVTVGASNGHFELNVFKPVIVSNVLRSIRLLSDGCRGFNKNCVKG

IKANKERIEKLMKDSLMLVTALNPHIGYDKAAVIAKTAHKNGTTLKEEALKAGVSEADFD

KWVDPKLMLGPN

>GMOY007471-PA alpha-mannosidase|protein_coding|scf7180000650411:202303-207950:-1|gene:GMOY007471

MLSIWRIVAYVCLTAALLHDVELRPQRTEGELLTNDQCGYESCPKVQPNMLNVHLIPHTH

DDVGWLKTVDQYYYGSETLIQKAGVQYIIDSVIQELLRDPEKRFIYVESAFFFKWWREQN

EELQEQVKMLVNEGRLEFIGGAWSMNDEATTHYQSIIDQFAWGLRRLNDTFGKCSRPRIG

WQIDPFGHSREMASMFAQMGYDGLFFGRLDYQDKTERLLTKTAEMIWRASANLGKSSNLF

TGALYNQYQPPPGFCFDILCADEPIIDGKHSPENNVKRRVDDFFKFVRKQAQYYRTNQII

ITMGGDFTYQDANINDFLAIVKKMAKSYRSRNVLITMGEDFNYQHAGMWYKNLDKLIKYA

NARQANGSEINLLYSTPSCYLKALHDVDITWPTKDDDFFPYASDPHAYWTGYFTSRPTLK

RFERVGNHFLQVCKQLTALPPTMYPEWSPHLSFMRETMGIMQHHDAVTGTEKQKVAYDYA

KRLEVAFRTCGANTRAALNILTTGDKPKPVEGHKPRPVKFEFKTCALLNISLCEVSEQSE

HFVLTLYNPLSSSTAEYIRVPVSDNNYQVLDDKVQYVPIPGGLMDLAYRRSETKYDLVFF

ADELPPLGYRSYYIKKTKERPLKPVPDSPNTSSMTSIGNEYIRLNFDTNGFLAAVTAEGM

TRIISQDFLYYEGAVGNNLEFRNRSSGAYIFRPKNDSMKIITTDVDITVYRGSLVEEVHQ

KFNNWISQVVRVYSQKNYAEFEWLVGPIPVDDDIGKEIITRFDTDIKSEGMFFTDSNGRE

MIKRLRNHRDTWNLEILEPAAGNYYPITTKIALEDDRARVAILTDRAQGGSSLRDGSLEL

MVHRRLLHDDAFGVGEALNETAYGNGLIARGTHYLMVGFSQKNDSPTQKTLERFAQLEVT

LPTWKFFSKTNYTFEEWMMSFNNSFTGLGKSLPKNIHLLTLEPWHNGELLVRFEHILEKD

EDPEYSKSVKFNIKEVLKNFDIQDVRETTLDGNAWLDEHRRMEFVSDPESIDFVNYATLS

EEHQSVHLLRARRPLTKSEYHKETLPDFDFESKSKQDPKPSIHLNTLRRNRLEQMKLDEK

DLKHPRGDSNNKYDVELNAMQIRTFVIYLRQNNSFASE

>GMOY007523-PA Scavenger Receptor Class A, Member 5|protein_coding|scf7180000650446:20021-25319:1|gene:GMOY007523

MGSPGIPGRLGGRGEVGKPGPAGPQGDAGEGGINSKGTKGNRGERGSDGSEGMPGYEGMK

GYKGDTGFSGAMGRKGEEGLKGYKGEMAEPADLQLQLLGHTGDKGEPGEGEGAEWKPGKL

VKGYKGDKGPIGFNGFPGIQGEMGEFGRPGLPGARGDIGEPGERGKPGKSGEPGFPGAKG

VKGAPGYNGLDGEDGLPGERGDDGFDGIPGVQGPAGPPGSYDPNLDISLPGPIGPQGEIG

MPGPPGLPGIPGKQGRQGTRGPTGIPGDAGLDGLPGRRGISVKGDDGDYGLIGFPGPMGP

PGYPGPIGSIGETGAPGKNILGPKGYRGNMGLNGLDGYRGDRGDAGLPGDKGDPGIGVNV

VGPPGPDGLPGPRGQPGDDGHPGYSGIMGDKGIRGDDCGICPSGPQGSRGIHGDAGYPGV

HGDRGLPGLTGERGPRGMPGRHGPIGFKGMRGPDGIPGEPGRSGPHGRPGIVITTGNRRE

AARGDTGDVGERGTQGQQGDRGLNGQPGLWGLKGEVGSRGDYGDAGRPGRDGSAGRPGGN

GRPGRNATTPKIYLIGDPGYDGRKGQTGEYGSIGNKGQKGEPNPGTFYDNRGDKGEKGYD

GLPGLRGLKGFQGDEGIPGMPGDMGYPGPTVQGPMGAKGWPGLSGDMGLHGTPGLNGLDG

EPGDDGLLGRKGQRGDPGPYVLPGDIGIDGQHGPKGEMGDIGFPGLPGPTGKPGVKGVQG

TKGDNGPQGLQGLSGSKGQRGDIQPGYSGLAGAPGRDGRVAPHGRKGQKGESGPPGPSGV

QGVKGSIGYPGRRGLVGEKGQPGMPGAQGMQGFVGIPGEQGDRGELGADGRHGDMGPRGA

LGLMGAKGVLGDFGAVGRPGNHGFPGRKGASGTRGIPGRTGPKGFPSRSGVKGEMGLPGF

VGAIGYDGLPGQKGMQGAKGDDGIGSDGLPGFKGETGVPGRNGLQGLAGLKGQRGDFGEL

GFPGEIGDVGEQGLPGYPGKHGAPGIKGSIGPLGSFGEQGAHGDIGEMGYIGYPGSPGDR

GEYGVVGEVGSPGEQGDEGLPGRPGVLIMSYAPQGDKGNQGYMGESGPVGERGYEGSPGY

RGRKGDRGDVGLVGLAGIDGIPGMKGEQGNKGYPGPPGQSSNYAEPGDEGDSGYDGLPGR

PGRVGPKGAPGDVGNDGIHGSAGQTGISVMGPQGMRGDVGYPGPMGRNGLHGMTGRKGDR

GDMGPRGIPGDMGMIIPGIRGDRGEPGPQGMRGQDGRKGDQGMSGRPGRSGPAGPPGPRG

PTGDAGWSGIDGLDGLVGEVGEPGVTFPFDFARKGDRGEPGIDGFKGEMGEFGLPGEVGY

QGAQGVRGYSGEQGLIGLMGPDGPKGDFGMMGHPGRTGLSGAPGRKGDMGNPAPPPPRPK

SRGFVFARHSQSVRVPVCPANTNKLWEGYSLAGNIGSSRTVGQDLGQSGSCMLRFNTMPY

LVCDINNVCNYAQNNDDSMWVSTSEPMNNAMTPIQPQEVIKYISRCVVCETTTRVIAMHS

QSMSIPDCPSGWEEMWTGFSYLMTTTDNTGGFGQNLVSPGSCLEEFRAQPVIECHGQGRC

HYYDPVASFWMAVIEEHEMWQMPRQQTLKADQTSKISRCTVCRRKNDSYITRLTPVDSAA

YEFRRGHERLAPAPPPPPPGPYRRPHNRRPYNRGRYPREDTTAP

>GMOY007550-PA hypothetical protein|protein_coding|scf7180000650478:7679-9485:-1|gene:GMOY007550

MLLSGLSKSCLSERNCKFLQKFMPITQQLELKRHQHEIPKHLEKMPEEATPEFSHMVQYY

YHAAARLMEPSLVKELEKKYPRADNKWREGRVAAILKFIGVVSCCIEVTFPLHKSDGTYE

LITGYRAHHCRHRLPVKGGIRYAMDVDQDEVKGLAYLMTFKCACVNAPFGGAKGGVRIDV

KKYEDKDLKTITRRYTMELLKKNMLGPGIDVPAPDVNTGQREMAWVVDQYLKTYGHDDIN

ALAITTGKPIHFGGVNGRTSATGRGMVLTKENADKVQAKILLEGANGPVTPAADQILRKK

KVLMIPDMYCNAGGATVSYFEFLKNINHVSYGKMSVKRESSMIHEIIESISQSVKQKIEP

TKKLENLRDCHSEAAIIDYGLQTVMETAGQGIKETSNEYSLCNDLRTAANIFAIQKIFNA

LESSGISQ

>GMOY007601-PA hypothetical protein|protein_coding|scf7180000650546:31707-37540:-1|gene:GMOY007601

MDTLPDTQCDKNTKASRFPNEIWAKIFSNLSHGDLLQVNLVCKAWYHVACMPQLKRKSKL

VITRHNVRDVCGFLDYKDLKYENVLVLDKYWGEASNVEYAYLFKIFDSLASDVACLTLYQ

PETLLALNNVLPNLRTLDIENMLIDDDVLVDFTKFPNLKSISMPQICDDYLMRRLVLSLA

QPPRLGLEKLSLFLSADCLDVLSMCASSLRWLTIGTSIWAQTIPTDRARLQETFAKFTQL

EVLDIYEIENIEDARLILETLPKENYLKTLRLNLSYDENLLELIVRKWPDSLECLELGCC

NLTQTNVKQQLSLISGKLRRFHLCEDGIGSEELLHIIAPKKNKKLNELKIHIFHLTGPLF

FLILVERLPNLTSLELERCASKLTDENLSYIFRYLTQLRILCVGPCSSELPITHLSSKPN

IANLKHLQALRSCFCPIKLRQFADKSAEYKPLRDEDRIFTNLYGRHDWRLNSAMCRGDWY

KIKEIIQKGREWILDEIKKSEEGERGKRRFPVFQKLENMKNKLPPTYLVVNGAEGEPGTC

KDRELMRHEPHKIIEGALIVSYIIKARVAILYIRGHYYNEACTMQMAINEACAAGLLGKG

IAGACFDFDMVIQRGGGRYVVGEETALIRCLQGQEGLPQRKPPFPADDGLYKMPTVVFNV

GTVASIPTVIRRGGDWYSSLGKDGDIRGSRVFCISGHVKRPCIVEELNAVPLRYLINRHA

GGVIGGWNNLLAVIPGGASTSLVPGGLCNNLILDAESLRKVNSNQGTGAVIVIKKGTNVL

KAMLPIMKFFRKGSCLQCPQCSNASLWYEEYLTKFIEGNAHPADIDFMWEIMQYSVGKTI

CYYTTANAAAMEGIIRHFGSIIREKMKQARLEMNRKC

>GMOY007615-PA hypothetical protein|protein_coding|scf7180000650553:365513-366547:-1|gene:GMOY007615

MSKRNLSMTVTLSSRVTIAGAVGGVGQALAMLVKTNTRVSDLGLYDLEDTKGLAADLSHI

DTYSAVHAFAGKDQLRKSLMCSKILVIAAGAVRADVKASRDSLFEKNVGVVGEIVMEAAE

VCPDAMFVIITNPVNSFVPFTAEILKSKNVYDPKRLFGMSFINSVRASTFVGDALIRNPQ

KTKIPVIGGHSRSTIVPVFSGIEPPIDMEEELKTEIYNRVVNGGEAVLKAKKGTGTCQLA

VAYAAKKLCDALLEGLENAPASGTALVPSELTKSPYFSSSFELNSKGIKKLLKLPELDAD

EKQYLEQCMRTLDKEIQKGVEQAKKIINAEDKKPKKKTGKEECN

>GMOY007617-PA hypothetical protein|protein_coding|scf7180000650553:396675-397784:-1|gene:GMOY007617

MVCVVEDEFGTVQDPLTGKALKVRRFTMTNCHQLSVSVITRGATVTSIKFPDKRGQIDDV

CLGFDDVQGYVSNRKSYMGAILGRVANRISNGKFEMACSEVCVSKNLSNKHQLHGGFVGL

DSVVWEVCKVRSNGLDLTHCNPSGHEGYPGELTVIVSFTLDDCNSFRICIEAESTDCTPV

NISNNIHFNLAGHAAGKESLYEHSVLIKADKVVDTDNDQIPTGMLMPVKNTPYDVRKFTN

LGKTIKKFRNCPITGFDKSFCVDVRPGNPETIAKIVHPYSGRWLEVITNQPTVVFYTANN

LPNMDATEYPVIGKDGAIYEKHGAFSFQTQQYPDAMNHKTFPDVILKPCDKYYHEVIFKF

GACKNCLKK

>GMOY007655-PA hypothetical protein|protein_coding|scf7180000650594:8972-19667:1|gene:GMOY007655

ADQLTEEQIAEFKEAFSLFDKDGDGTITTKELGTVMRSLGQNPTEAELQDMINEVDADGK

WKNGKTGKRNGTIDFPEFLTMMARKMKDTDSEEEIREAFRVFDKDGNGFISAAELRHVMT

NLGEKLTDEEVDEMIREADIDGDGQVNYEEFVTMMTSK

>GMOY007736-PA arginine kinase|protein_coding|scf7180000650651:465536-476826:-1|gene:GMOY007736

MGGCASKDKKDKVVDGDAVANATENTADGTKAADATTTDQPNGDATTNPEGKDTMVDAAV

LTKLEEGFAKLAASDSKSLLKKYLTKEIFDNLKEKKTPTFGSSLLDCIQSGLENHDSGVG

IYAPDAEAYTVFADLFDPIIEDYHGGFKKTDKHPPREFGDVNCFSNLDPNNEFIISTRVR

CGRSLQGYPFNPCLTEAQYKEMEEKVSSTLSGLEGELKGKFYPLTGMEKAVQQQLIDDHF

LFKEGDRFLQAANACRFWPTGRGIYHNDNKTFLVWCNEEDHLRIISMQMGGDLGEVYRRL

VSAVNEIEKRLPFSHDDRLGFLTFCPTNLGTTIRASVHIKVPKLAANKAKLEEVAAKYNL

QVRGTRGEHTEAEGGVYDISNKRRMGLTEFDAVKEMNDGIAELIKLEKENMVEAWFMDDE

TSDQRLQHHCDPPEYINMNELFKKTGVEYFQINADNYENDNVLQELRKKRNYSYEDEITC

SEKCLPDYANKLISFFTEHLHTDEEIRLVLDGSGYFDVRELLKNAKIVKKNSPSAQEKWI

RIAVTKGDLIIIPAGIYHRFTLDVNNYIKAKRYFVGEPIWLPYNRPADNMDCRKLYLKHQ

GEGGIVLTASHNPGGPDNDFGIKFNCDNGGPAPDAVTNHIYQLTNAIKDYKIVKDLQVDI

TKVGINTYTIDNQQEFVVEIIDSVENYVNCMKEIFDFVKLRKFLSGETTGKPLRILIDSM

NGVTGPYVREIFLNCLSASEDGVVHTRPLPDFGGLHPDPNLTYAKDLVQTVASGEYDIGA

AFDGDGDRNMIIGYKAFFVTPSDSLAVIAHHLECIPYFQKHGIQGFARSMPTAAAIDLVG

QKLGRQVFEVPTGWKYFGNLMDAGYLCLCGEESFGTGSNHIREKDGIWALLAWLSIMQHT

GLSVEDILKQHWSTYGRNYFTRYDYEECELQSCNDMMAYLEKTICDLSFVGREFTAEGKS

YIVKVADNFSYTDPIDKSVAAKQGIRILFEDGSRIIIRLSGTGSTGGTVRLYIDSYEKDN

TLGQANIMLKPLIDVALEISRLPQFTGRSSPTVIT

>GMOY007746-PA troponin|protein_coding|scf7180000650658:119763-135296:-1|gene:GMOY007746

MEEASKAKKAKKGFMTPERKKKLRLLLRKKAAEELKKEQERKAAERRRIIEERCGSPRNL

SDASEEQLKSLCKQYHDRICKLEDQKYDLEYVVKRKDVEKTTSLGALKKICQDYYDKILK

LEDQKYDLEYEVARKDLEASILFLKPSFIIFFLLKSKEKYIIIFLTIFLIIGDHMPYTHI

SELQTICKEYWNRLYKLEGDKFDLEHIQKIKAQESTFHKKPNIQLNFPGELQEICEEYFD

RMYQCESQKWDLEYEVRKKDWEINDLNAQVNDLRGKFVKPALKKVSKYENKFAKLQKKAA

EFNFRNQLKVVKKKEFTLEEEEKEKKPDWSKGKPGDAKEHAPETPA

>GMOY007757-PA odorant binding protein 4|protein_coding|scf7180000650660:4511-5002:-1|gene:GMOY007757

MFRVTLILLAIVTPALFSENRYMEFLADFKHCKRERGVGRFELDRLRVGNLAYPSYEAKC

FLGCLYERTGILKNGVLQNDVLKKNVGYIANRVLLDEVLPPCYAVSGTNKCDIAFELKKC

FKNVGFDKVWITVPWEDNTDPQYIAAMKLIDDLANVKYRVAFA

>GMOY007760-PA hypothetical protein|protein_coding|scf7180000650660:231141-231521:1|gene:GMOY007760

MKLLITLTVLGISAPATVGILKLFDCAAKLLLGGDCNEKTTPMQIQNTVHIQGGTPDYSR

QPPPGPQPPYQPPAAPPQYPPPPLYYPPPPPPLYYPLPPEWASYNNPPRDPQQDYSQNTQ

QNQYKK

>GMOY007767-PA hypothetical protein|protein_coding|scf7180000650670:104574-113797:-1|gene:GMOY007767

MINLMRQQKKKEKKIAIACIFMQSVATVKLDAQNRRLILDIKDQKTTNKQYYSSNFDTNA

YRYGYDIGKTGNFHHETRGPDGVTYGCYGHIDPNKILRATHYVADTRGYRTVEPQKPVVT

YPEADDPIKGPSSGILLQWDELYFPIGCGKFEGGVRPDIPLVFIDDVRPVFQGTDNHVFN

FSKFTHLTPKDSKQSGLNLHSNIGGRPVNSNAGLFDNSHTYEGSPGAQASTTHSIQSLHP

SGDNRDKNEASGQLNHFEIPQNKTAAGQGFDNFRGPGNKHTGNRENTGEHTDLDFKGFEG

SNTPASQLRPSTQHQNSPDYLLHGSSSSHHPVKTHSSGFQGSGGSAGSTGVASSVGSGGL

GSAGSSSSGSRGPGNTGGSGSFASFGGPSGSGGPRSPGGAGGSGGSGGLGVSDSSIGFPD

LGSSNSYASHVGSGGSDGPGGLSDSGNYASFGGSTNYVGSGGSTNYVGSGGSGKYSGPRG

SGNYAGFGGSGSSNNSAGTISSVNSTGYVGSVGDIGSVSSVSSVDSSGSMDSVISVDHVT

SVDATTSAGIADSADSVASAGFESYAGSGSSGSPSPHDLFKPASHNSIETQRPSGLFDSE

GSGKYEPSPSASFTEFTKYEANDLPAVSLDGAATLPGGSSDFLGKYGLDSRPGSGSPGKP

QQYISSENNGKYVPGKEGKYNHEKEFLVTGFPGAIAYNKESGKYKKTHDDGRYKGIQQGK

YYHDNSGKYVHIEGPTGPPAPPYVHIVGPNGGYGGAGGDGGAGGFGGKGSGGGAGPGGPG

GPGGPRGPGGPGGPGGPKGPLGPPGPPGPPGPPGPFGPTGPGPKGPFGPPGPPGPPGPDR

PGPFGPPGPPGPPGPQRPGPPGPPGPKRPGPPGPSQPGPKTPGFGESIDSVPIVPGKKPK

PKYPSDPRESALQVPGYLPPLISKNGKITQTNTTYSYQVDRVPPPITNKQTSFVSTYPKD

NINAQKKVTATTTKPTTTTTNTVSNKQFVDKKINTVLTYLPPSSTPSPKIQQQQQQRQQI

KTTATTTGTRKAQDIVIKQDKPSKTYTTTTNQNFGSKQGLNIEKTIVTTTNTQYSKPRTT

IDKVVTTNTKTTTYSRPIAIEKVTSTNISTPSQGQKLSKISTTTTYTKPETIEKISTISY

SKPQTAEKLITNNVSSYVSPKGFENTVNNERGKVNIVSTDYQNKRPEQKITTVTTGKTTS

YQQQSASPRPTSPPFQAQKANNYCSCPADTIQSTKSSSSSTTYTAPSSKGFATSPQSLPN

LVSHFQQEFITSSGFSGHVNFPALSQQIPVVPQVPGHPAFYPPDKIPNGATVAFLPIIIL

PEVAYGKCNDNANKSTSEHIHTVGVQPAPIPINFSLNSIFSNGSNKSQCMCPCSCTQNIP

EHQTKKRETNLATTVENMRKNRAVDNAEIEQTSQPDSKSVANDSSYVSIDDVKQI

>GMOY007817-PA hypothetical protein|protein_coding|scf7180000650671:1508803-1511091:-1|gene:GMOY007817

MWCSKIAFKAANFGIEDDDKPEAPSPCEEKVEIAPVCDDKKKDTSKKMKVPPPKDPSKKH

DASTRVGKIYSIIGPVVDVIFEHDVPEIMNALEVPDYPGGRLVLEVFHHLGEKVVRAVAM

DCTEGLCRGQKVIDSGYPIKVPVGKATLGRILNVIGEPIDERGPVVSDFYSFIHADPPVL

TDMNVSPELLMTGIKVIDLLSPYVKGGKIGLFGGAGVGKTILIMELINNIAKAHGGYSVF

VGAGERTREGNDLYMEMIESKVISLEDDTSKVALVYGQMNEPPGARSRVVLTGLSIAEYF

RDVEGQDVLLFIDNIFRFTQAGSEVSALLGRIPSAVGYQPTLGTDMGSMQERITTTRNGS

ITSVQAIYVPADDLTDPAPAATFSHLDATTVLSRPIAELGIYPAVDPLDSTSRIMDPMIV

GEDHYTVARSVQKTLQNYKSLQDIIAILGMDELSEDDKLTVARARKMQRFLSQPFHVAEV

FTGHPGKLVPLNKTVEGFKRLLQGDMDDLPEVAFYMIGDIDEAVAKAKELALQLKG

>GMOY007827-PA hypothetical protein|protein_coding|scf7180000650671:1615927-1617216:-1|gene:GMOY007827

MASTVVKTLRPTGRVLQSIAPLGLNRRGNAWFEDVEMGPPDPILGVTEAFKRDKNPKRLN

LGVGAYRDDSGNPWVLPCVRCAEARIVEKQMHKEYAPIDGLPEFCAKTIELALGKDSYVL

KDKRNATAQSISGTGALRVGSAFLAKFWRGNREVYVPNPTWGNHIPVFEHAGLPVKKYRY

YDSDTCGLDYKGCKEDICKIPSESIIVFHACAHNPTGVDPNIIQWCELADLVKHKCLFPF

FDMAYQGFATGDLDRDAQAVRIFESKGINFCLAQSYAKNLGLYGERVGAFTIACENEVEA

CRVLSQLKIIIRALYSSPPLHGARIVEEILTDPDLYDMFLKDVKTMADRIISVRQKLKEG

LIQSGSKRKWDHITNQIGMFCYTGMKPEQVERITKEFSVYLTRDGRISMAGVTSKNVEYL

AKAMHEVTK

>GMOY007834-PA hypothetical protein|protein_coding|scf7180000650674:308387-310995:1|gene:GMOY007834

MAYRLINSAQVMERCIRRSYLSHSAASTKEMKHSQGVAFLEQVPLVRYLLEQIRYFCKKP

PKGFEKYFDEGKKPGAKAAGGEDGAKKGDAPAQPSKNLPPSQTQSTPQQQQQRSDWNFGM

FTNKQQQGGRQGGNRNVGDGGSGEREKWIILGAIAAAALVGSFAFFEVGYKEIAWKDFVN

NYLNKGVVEKLEVINKKWVRVRLLPGHSKDAGGMLWFNIGSVDSFERNLENAQIEQGFEP

ANFIPVIYRTEVEASSLTGLLPTLLIIGFLVYMMRRSADMMGSGRGRKGGGLFGGVMQST

AKLINSNEIDVRFKDVAGCEEAKIEIMEFVNFLKNPQQYIDLGAKIPKGAMLTGPPGTGK

TLLAKATAGEANVPFITVSGSEFLEMFVGVGPSRVRDMFALARKNAPCILFIDEIDAVGR

KRGGKSFGGHSEQENTLNQLLVEMDGFNTTTNVVVLAATNRIDILDKALLRPGRFDRQIF

VPAPDIKGRASIFKVHLQPLKTDLDKQDLSRKMAALTPGFTGADIANVCNEAALIAARDS

NETIILKNFEQAIERVIAGMEKKTNVLAPEEKKTVAHHEAGHAVAGWFLEHADPLLKVSI

IPRGKGLGYAQYLPKDQYLLSKEQLFDRMCMTLGGRVAEELFFNRITTGAQDDLKKITDS

AYAQIVRFGMNERVGQVSFDAGIQGDPVFTKPYSEETAQMIDNEVRDLIKKAHEVTTVLL

TKHKEDVAKVAQRLLKNEVLSRNDMIELLGPRPFKEKSTYEEFVEGTGSLDEDTTLPKGL

QDWNKQKTPETPTQPAKPVPTT

>GMOY007874-PA hypothetical protein|protein_coding|scf7180000650697:39612-40848:-1|gene:GMOY007874

MVEKKPIVTDTKTPLTENVEKRFIDTIPCTYILSVLSATAAELITYPLDLTKTRLQIQGE

LAKTQEVKAMHRGMLATAFGVVKEEGPFKLWCGMSSIIYRHTIYSGVRVCTYDYLRTTFG

HDNLPVWKAALFGVLSGCFGQWLSNPADLVKVQMQMEGKRRLMGEAPRFLSFHQAFVDIY

RRGGIVGLWQGSVPGLQRAALVNLGDLTAYDMTKRFLIHKMEMEEGPLAHILASLASGFV

AAVAATPADVVKSRIMNQPTDERGRQVSGSLDCFKKTVEAEGYMGLYKGFVPHWMRLGPW

TLTFWITFEQLRSILGGAAF

>GMOY007910-PA succinyl-CoA synthetase beta subunit|protein_coding|scf7180000650717:22174-31310:1|gene:GMOY007910

MTISYSDFLDVREEYPEIDYEIRGILLARAQNGATIDEIRDDYQKLTGLRFPVKENITDF

LLSITHVMVFCNENGTRIFNIMPTDTTRHLLDLVLNQKISQRTGVYSTLQSNNQNQRNYY

QRPHQQFWRYKSSCIAKQRPEQHQLPEIQIYPDADKFRRPVNASLQQEAEDIGENNCYYE

DNCNYLFNRFGKQQKLDNQATIKLHPPQHPTTATTAGLAATISRDLRKTLMNDVSSNSKQ

DVCAIETIPLESSNLIPMSRSSLSTNNNYPILTSLIPPYSQHSSPNAAKSCQTTDSIFTD

SDYEAHLLDFLLLGDDFFLYMARMELGCKFKRNQRVLQSGLCVSGQTVSGAIKRLNQLNN

FTDKSIIINIGSVDIMQGRHLIQIEHDLRELVVIMLKKKIKPILTTIAPLANYAHNTEVK

TTLMRLNDYIKRLGRARQLMVIDIWKCLVNDKNHTLFDCYQRRQKEIMASIFTRTGALAE

IVNQKIGKKVLGSALGAVTNQQNRKLNVQEHVSYKLLNDAGIPTPKFGVATSGSEAKDIA

TGLKTNNLVLKAQVLAGGRGVGHFKNGLKGGVRIVHTPEEAETLAGKMVNQLLVTKQTGP

AGRICKKVMVAERKFPRREFYVAVMMERAFNGPVIIASSQGGVNIEEVAAENPEAIIYEP

IDIKQGLTKEQAKKVAQAIGVGAAHEEKTIQLLENMYKLFVQKDALLIEINPYAEDAMDG

FFALDAKFRFDDSAEYRQKELFALRDWTQEDPKEVEAAKFDLNFIALDGTIGCMVNGAGL

AMATMDIIKLYGGDPANFLDVGGGATAQAVKEAFKIITSDPKVLCILVNIFGGIMRCDVI

AEGIIAAAKELNMQMPIVVRLQGTNVNEARELIRKSGLRIIPRHDLDEAANLAVHLAKIV

ELAREVKMDVSFEIPDHAK

>GMOY007957-PA hypothetical protein|protein_coding|scf7180000650781:2022-3650:-1|gene:GMOY007957

MLGCTTRVFEHVRNYASQHLNQILQLQHTEICADPPSRALVLGVYADETDKTDTGVLTET

AWRYNLSRTNGRMIEVLRMSGPMPKRGEARLLFAMEPEKVPYYSAVAIVGLGRECLGYNP

YEIIDEQKEAIRRSVAQACMQLSMLDTDRIDIESCGHAESAAEGAALGVWAYQELRAPKD

RIAVPAIDLYATKDEVCDIEGWRIGLQKAAAQNLTRQLQEMPSNILTPTAFAQNVVEVLC

KSGVNVEVKVEGWAESQVMNAFLSVGKASCEPPIFLELSYYGTSPEERPIVLVGQGITYD

CGGLCLKPYEKLKVMRGDMTGAAVVVATCRAISALRLPVNIRGLVPLCENVMGCNSFRSG

DAVKCMNGKHIKIERTDYEDTLVLADALLYAQNFCPKCIVDIGTASWCLNHTLGEAACAI

FTNSEILWQQIKHASMHTGDRVWRMPLWNYYTTQVTSGMSTDVQNYGSGTGGIPCKCAAF

LREFVPCGQWMHIDATNVMTTGGDTFEYLRDGMAGRPTRTLIEFIAQTLCKDAAPKFPKR

EK

>GMOY008018-PA hypothetical protein|protein_coding|scf7180000650847:14282-22215:1|gene:GMOY008018

MKTPEILSKEEARTSARPLIVMHYNQFIYSRERRRFGRQCLFSDRTQLMFSINPNMKLRR

NYILRNPVSTGTQLSDQKAFSEVETENVTYAEHGMYHYEGGWPKEVNINDEESTLRYRKK

IEREDAWGQQVITLIADATAVIEQNNAVNIYQEFFIDLPAEICEQIRFDFESRQKNIFHD

PYTPHRPISVIDWAPNDVKRFLVLHTNIPLSRLEAIERLKTPKTRIVKFEGRLESTEGNN

FYIWDLENPLKPMVDFTSTEIVRKTFFCPKDENWIAGGLHSGKVCLWDARTGGARIFVCP

LEAAHREATSALCWVHSKTNTEFYTGSLDGSVKYWDGRDMETPIQEILLDPVLTDSQLRS

RSHGVTVLEFEYTIPVRFIIGSDMGSVFVGNRKGMTPSETLAAGNYRIFSGPIRTIERNP

FFVKNFLITGDAKGELDFWDLLLHQRKPIFTFKFSHAIVCAKFRPDGEYLAVALSNGDIQ

ILEMDPALKHSTSKDKALIAADIMNPDDPEEFLRIITRDTEFKEVLNTFQDQVYKVDKKR

QEREYVMEQTDFEADFEPSEIDERRYFGRPTAFENKDEVLFNENSNRELSKNFVLRNPVD

DGTQYGTRWSLSLANTERATYKHIGITHNEGGWPKDINMHDPEQTVRYKRKIEKDENYIN

EVMNLTKPMEQYILQNNAVNIYENYFENSEPAPLPEPCHSRTINVYRDPNPIKVPVTHLS

WSPDGGLRMAISHCDMKFQGDKTGQKCNSYIWEIENPNEPYITLEPKVPCVCLEYNQKDP

TSLVSGMYNGQVAAWDTRHGRFPVMISEREICHRDPVNSVLWNNSKSGTEFFSGASDGQV

LWWDTRKLNEPLDRLLMDPVRSDEQDLGRSFGVSVLEYETTIPTRFMVGTEMGMLFCCNR

KGKTPVEKIQIRMMCHLGPIYAIMRNPAFVKNFLTVGDWCARIWSEDCRESSIIWTNNSP

AMLTDAAWSYTKVSQFFITRMDGVLDTWDLLQQQNEPVLTVKVCDEPLYCLRTSESGKFV

TCGSKLGTTFLIEVSENMVTSNKNDKPLLTAMFERENRREKILEAKSREIKLKVKVNQAV

DQNDVTMVDGKFNLDAFKNIMEQVEAEYFAAVEQERLRRVPGNKQDAEESELSEAGSIKT

RD

>GMOY008072-PA mitochondrial F1F0-ATP synthase subunit delta/ATP16|protein_coding|scf7180000650885:82911-83524:1|gene:GMOY008072

MFVKNARLLARRGTHLMQARGYADEMKLTFAAANKTFYDNADVRQIDVPSFSGSFGILAK

HVPTLAVLKPGLVQVIENDGKTSKYFVSSGTITVNEDSSVQVLAEEAHEIKDIDVSEARQ

LLSKYQSAISSASSDAAKAEAAIAVECCEALVKAAE

>GMOY008085-PA hypothetical protein|protein_coding|scf7180000650893:154799-160712:1|gene:GMOY008085

MHVPVTLQTVRAATKASKFVKYFTTLPEPKEPTIMSATIPGPKSLELKKKLGGMQMVETV

QFFVDYECATGNYLCDVDGNVMLDTLMQISSMPLGYNHPRLLGVFNNAKSVKTLVNRPAL

GYFPGKDWATKLENILKEIAPKGLDNITTMMCGSCANENAYKNIFITYMRKKRGEDACFT

DEEIETAMRNCPPGSPNLSLLSFKGGFHGRTMAALATTRSKYIHKIDIPSLDWPMAPFPN

YKYPLEEYERCNKEEDKTCLAQVEELIEAYCKKEKHVAGIVVEPIQGEGGDNHASPEFFQ

QLQCICKKNDISLLFDEVQTGGGCTGKFWCHEHFDLPSPPDVVTFSKKLQLGGFFHTSDF

RPREPLRIFNTWMGDPARILLLEQIVTVIKEDKLLEQVEKSGKVLKTGLLQLEKEYPHLV

NSARGRGTFLAISVETPELRDTIVGNLRKLGIITSGCGQQSIRFRPALIFQETHANIVLD

RLRKVLTMAFGDYPAEYNPKVHGPYDPARYYGKPDTPFGQVKLSELGAWFSRRNKSPNAL

AGAMSRAWWRWQHKYVQPKRAGIAPFFQITVAAMTFFYVINYGKMRHHRNYKYH

>GMOY008087-PA hypothetical protein|protein_coding|scf7180000650896:39834-53432:1|gene:GMOY008087

MWVNGRYRWIKNGKKFDWQAYDNRMLQQPGRGTLVITSPKDEDLGQYQCFAENEYGIATS

NSVFVRKAELNAFKDEAAKTVEANEGEPFSLQCEAPDGWPKPTVNWLIQQSIDGGIKSIN

NSRMTLDPEGTLWFSNVTRDDASDDFYYACSATSVFRNEYKIGNRVLLDVKQTGISAGQN

KNAPKRQYVTRKNEVALRGKRVELFCIYGGTPLPQTVWSKDGQPISWSDRITQGHYGKSL

VIRHVNFDDAGSYTCDVSNGVGNAQSYSINLKIHAIPYFTVEPEIQNAAEDETVEFRCEA

VGFPEPTIHWIHNGKPISESPPNTRRTVETNRIILSDLVKKDTGNYGCNATNSLGYVYKD

VYLNVLALPPEIEEPPRKEATVDGKDITMRCRVFGAPKPQVKWIRNGLELTGGRYSVMPS

GDLKIEQVNFNDAGVYTCYAKNKFGEKSANGSLIVKEHTRITEKPQNYEVAAGQSATFRC

NEAHDDTLNLTIEWWKDSQQIDFEAEARFVKTNDNSLTIAKTIELDSGEYTCMARTELDE

ASAKANLIVQDVPNAPLLTHVRCLPRHADITWEPQGDNRSPILHYTIQYNTSFTPATWDV

AFEKVPSTDFSFMVDMSPWSNYTFRVIAYNKIGSSPPSGHSQTCITQPDVPYKNPDNVAG

QGTEPNNLVITWTPMPEIEHNAPNFQYRVSWKRDIPAASWENKDIYDWKQSSLLITDQPT

FERYVIKVAAINERGEANVAAEEIIGYSGEDRPLEAPTNFSMIQVTGATSAILGWNPVSP

KSVRGHFKGYKIQTWTEQEGEEGLREIHVKGDSNQALVTQFKPDSKNFARVLAHNGRFNG

PPSAVIDFDTPEGVPSPVQSLDAYPMGSSAFWLVWKKPLNPNGKLTGYKVYYEEVKNSYV

GERREYDPHITDPRITSMKMAGLKPNTKYRISITATTKAGEGTDHFIEKRTLAEDSQEPA

VPAFEFEQQPSENGLAKFRINWKPNTEGHAGTHFFTKYRIKGETEWTNAPEEKNRDYQEI

AGLDPDTVYEFRVVAVDGHFFTESATREIDTNGVEGPIKVPNENLANAGWFIGMMLALAI

IIILFIIICIIRRNRGGKYDVHDREMQNGRRDYPDEGGFHEYSQPLDNKSAGRQSVSSAK

PGLESDTDSMAEYGDGDTGQFTEDGSFIGQYVPGKLQPPVSPQPANNTPASHTTPTAQTA

PTLTNQTSTGTNTNPSATYV

>GMOY008106-PA hypothetical protein|protein_coding|scf7180000650913:57984-67973:1|gene:GMOY008106

MPLEINTPNKLEYQFHPVFGSLFTFKVRCANDAHLTLATNPVEENPTYEVFIGGWSNSKS

VIRKNRQKPDVAAAITPCIVDANEFRGFWIQWSNNIISVGREGEAVAFLSYEAQDLFPIN

FIGICTAWGASGTWLIDEMPAPVPSRITRAPLAVPRVPVIKEVNTPDKLEYQFHPVSGNV

FTFKVRSPNDAHLALTSNPVEEDPMYEVFIGGWSNSKSVIRKNRQKPDVAEADTPGILDG

GEFRGFWIRWYDNVITVGREGEAAAFLSYDAPDLFPINFVGVCTGWGANGTWLLEESTAP

SAPSEPAMGFAPPTSGGGPGCWVPAANGEIPPTSMEGGFDGSEQLYIARAKHEGDLIPGK

LHPSHGVCYVAFGGGEHGHNEYEVLCAGGGQWVPVQDGNIPPNAVPGGETAEGEPLFIGR

ATHDGTVTVGKVQPSHGCCYIPYGGEEVAYKDFEVYVAM

>GMOY008231-PA hypothetical protein|protein_coding|scf7180000651051:24977-27203:-1|gene:GMOY008231

MERRRSHHNVKSYKRSYRNDSRSRSRSVERHRRRQHRGAFSRSRSRSRSRGGYSSRHDRR

QSFRRHRDSRTRSRSRSRSHRRYQREMDDLSRRRSCSRSSSQLDDDRCRSSKKECRSSNK

IQNSDLHTHSKPCSLTNMAEDTRDDAGQHTNDLNELPLPERSYSSSESYQENSYYNFDTD

EPIDKERIHREMEEKLRQTLAKEGKVYPPPKPEASHPVFANDGSFLEIFKKMQEQQKQQA

STSSVAANVVPVVPVLAVANPTTVPYLAATAMGKSAPPPPIVGRRRGGKILKTGVVAKPK

VQNEPANDPKDFWSLYLAEVNKYKNTACESEAGDKKKENPAPVEMTVKLETAPFDPRFPN

QNQTRYCYQSYVDFHRCQKKRGESYEPCNYFKKVFKSMCPNAWVEKWNDQIENGTFAGRI

>GMOY008272-PA hypothetical protein|protein_coding|scf7180000651104:9461-16133:-1|gene:GMOY008272

MLCWQNEDKFLQKRYPLLYPKTETLKDLLQKSSVPIPWSMAGAPPCMEPVSGPSVPPRVG

TSYQTTHPHPWRPTLSYEMIEVKNLPEQPVTNQLVKPCFAPSGMTTEPLTFPNLVTGFLR

NPQHGARAALYTRYTSGEWNNNNLTKYAESNINRNQSERIRNDAVRLMRETDEKTSQGQR

DAGRRLGERITDVTFWRNELFTELEKLISESSAFTELKRKCGKAMLDLEAPLHIAQECLY

HREGRAGVEKVHDSVEKALLLEIDNLRNSRQRLQDLHQKITRQANDCRAAQHLLEEDVTH

KESTLGIDSVCHQLNNYSRGIAYYGGIEKYDPTVSTQESWAEASSQRVSRSQAERAKLSQ

LRSDAETTKAIQDKANPLKVAQTRLESRSHREGGELCKDYAQLRLIQEVQDIKDVVNSLH

TKLQEAEAQHQSLLKTRSTLEVDLRNKVNALFIDREKSYIFHMTFGVDPNNFKKCEQSSF

CRRCRKIAPGNSKFALVPGSMNTYSNAITVDLINKDTQHLYIVKLEALVNNSYRLIIDEK

TPLRPRYKVTHALVAEPQPENINIQKEDQNEVVLTSGYNKAVIVADPFRIDFYENNVLAV

SANAKGLMNFEHLRKKSFNPDENVVENADDVSETNEKAPVATPQEDDPGAWEENFNSHHD

SKPYGPEAVALDFSFPEAEALFGIPEHADSFILKSTSGTEPYRLYNLDVFEYLIDSKMAL

YGSVPVLYAHGTQCTAGVLWLNAAETWVDINTAERNVVSSIVSFVSGSRKADPQSAHFMS

ETGIIDTFIMLGPSPMDAFKQYASLTGTAPLPQMFALAYHQCRWNYNDAADVESVVTKFD

EFDIPMDAMWLDIEYTDGKKYFTWNSYKFPEPLEMIKNLTDLGRHLVIIIDPHIKRDNNY

FFHKNCTDEGYYVKTKDGKDYEGWCWPGAASYPDFFRKDVRDYYAKQYLLENFRTTTGDV

MLWNDMNEPSVFNGPEVTMPKDLLHENDWEHRNLHNLYGHMHIMATFEGLLNRDPKQRPF

ILTRSHYAGSQRFAAIWTGDNLADWGHLQHSIKMCLSEAVAGFSFCGADVGGFFGNPTPE

LFGRWYQTGAFLPFFRSHSHIDTARREPWLYPEATRLIIRDAIRKRYSYLPLWYTMFYEH

ELNGGPVIRPLLAHYPKDTNSFTVDNQFLLQNRLMVRPVMEQGVSKVSVYFPAIDDKKTS

DLWYDADDYQKYDQAGSQTIPVNDYKIPVYQRGGTIVPKKERIRRAATLMKNDPYTLIVA

LDRDGKAEGTLYLDDEKTYNYRQGKFIYINYKFDGRKLFNEFINRPKYVSKSWIERVVIA

GLTKKPKSATIIVDGLTQELEVQPKEHAYVVRKPGVAINKDFEIKLNF

>GMOY008287-PA hypothetical protein|protein_coding|scf7180000651113:22150-24713:-1|gene:GMOY008287

MLRIPGMPFLPGTVFRDISKSHYPKPQSLMNYQGISMLSDRKPPGLVDASGFVIDPNCPP

VSVPSIYPPKIGPKLPPWLAYDKQVLCFNAYFKETLQEVYQAPYQVRKVKILYYLEGGTM

QILEPKVENSGIPQGCMVHRQRIPKPPPCESDFISILDLNVDQTIQIFDRVYHITSCDKF

TRHFLNKSGIPVPDPVDAPTDPTTEMRKRADGKATITKEKKHPFAQFLEFDRVVLKFSAY

WDDRTEFGDVRRLEVCYYLSDDTIDIKEIFPRNSGREGPSTFLKRSKLPREFSGIPLPGE

QTPHSLLNVLGTNMRNVRYVVDPLDVGRKDIIFYKDKDLQIGTVLNVYGRAIVLTDCDEF

TQSYYRKRYGIEDFTPAPVPMRADDCMPSKIKERILPPYNGWGTYEDSEGNCLSVEPKPP

QADFKKFIQLDRFVLRYGAKLLSTIKENCERVFIVSYYLSDDTIQIYEIAERNSGFLGGE

FMKRTRIPLPGQEKFTSRRPQYYQPYNFYIGATISLKDHIFHIFPLADVKSIMSKIREAI

RPTYKNFVCNCLSESGGDQKEVRFIGYESLKRALVDALGDKITNHEIITLCRHFSAEQAP

PMTCNKENVLGAVRLELKRTLWNAMDELKEHLYHINPLNKPFLPEVKLRSTLKGCRLPFS

KELIDDMMSVLNQNDCGEIEVCDFLNFLDMSCGSGPDITPVNVAFELCPKIPFLHKGRLV

NFSCFLEHLGLEQDLKKDGE

>GMOY008463-PA hypothetical protein|protein_coding|scf7180000651334:79177-86109:1|gene:GMOY008463

MFLIKFPSNSLALSVVLLWFLLDSKCVADRVRVINLNRNWTLSNENGTITKSGVTIPSGV

YSQLYGEKILDSYNDVDLRWISYDNWTYTYSFSVKTEDFKSICFVNLTLHGIDTVAEVRL

NGDWLGRPENMFVRYSYDITKLLQEDNILEIEIKSPVYEALNRARALKRLGLDIPPNCPP

SQYHGECHMNMLRKMQASFGWDWGLAAPSMGIWKSVTLEYYDVAIIRDVDVMLTKYSTHW

TMENRVFIDCNGDFYAELTFYAVELFKNPVIITKHVKTPVSCKAPFITFDIDVPINEVTL

WWPNGYGDQKLYPLHYSLKAWSNPDGPTLRDNVNSEKSIRIGFRTIELIEDPVKEGTGNT

FFFKINGKEIFMKGSNYIPSHILPEYSRDSNRLKHLLQAAKDTHQNMIRLWGGGIYESDE

FYDLADQNGLLIWHDMMFACAMYPATETFLASVRLEISQNIKRIAHHPSIAILATNNENE

VALVQSWYGTSFEDERFRKDYRKLYLATVMHELKIVEHSSRPIALVSSPSNGKASANDNY

ISDNPQDPDYGDVHFYDVFKDAWDPSIYPRPRFSSEYGFQGLPQMASWLKTAAPHEKLIP

LIEHRQHHPVGIPVMTNLIRRHLPLPLPEDAPYLEALVYFSQIAQAMATKVETELYRSLR

DTEHRTMGALYWQLNDVWIAPSWSSIDFYGNYKLLHYWSQDFLAPISIIALYDRNSKAIN

VSLVCDEFEVDTNDLKVTLNIYKWSQLLPTNSTTWPVTLKSNGVHYDKIIPISEIITGEL

TTKNCLVEFVLRRGSTFLSHTFYFPGSIKESLAVKDPKITLEISHRYCSSSAYVKTNNYS

LTVSTKYPALFVYIELLRPDISKYKLSCNGFMQVTPVQAIHLEFEANNCINLTRHDIKLT

TVNQYLKTRPI

>GMOY008537-PA hypothetical protein|protein_coding|scf7180000651417:61398-62057:-1|gene:GMOY008537

MYKKIIRASLLGPPGSGKSTVAKRILEKFDLVHISPGDLLRANIQKKTDLGKQAEAYVNQ

GKLVPDNIIIEFITNHLRDLNCASFLLDGFPRNVTQAKCLSEVECLNAVIYLNIPHEELI

RRIEGRFVHLKSGRVYNTNFNKPKVPGKDDVTGEDLIKRDDDKPEVFAERLKTYDQTVKP

ILEYYEKEYPGILKTLNDTTSDEIVCKMEKLLADIPETP

>GMOY008545-PA hypothetical protein|protein_coding|scf7180000651420:23120-24752:1|gene:GMOY008545

MEIFAKFSATLQLISQITRRLTYSFSSRPAVSLERTHNCHSGLEDLNSQESHTANKLKDD

KQADFVNPLADSRLKLSNLTADLPTIAVVDITPLGSPGQMNRFLITVNERNVGSVVKNSS

RLIESCEALGNETKTNITLDNNTGLMPWNETNISAIAQFTERNKDIKCLSTKNQLLKLID

LVQPATEIKKNTEFPKKTQNALMLYVKPQVYSPTFEPRGRSYSTRTFAEKTAESEGRGKK

SASPQLEFGRSIKLSENSFINESAKKDTPEHNIPMLQKIVDVNKDLHMRSAAAKSMEENI

SKNVEKSISKTNALHPKEMHQELEKLIRKQQAGKQKTKSNSIVTPFKPSPTEKDRVYKAS

TRSKPTSRFRNAILAKDKCPPDPCKEEKKCPDPCAQEYEKLEHNRKVDEHMAKKKRKKIP

ESCISDKCKRKGGCAPPPKKLADSSGKFISILANKPARNGMRIAGVSMKAWPRFNPKSTT

KSKNKLKNTLNRFSSPNAVLAIKGKKGGKTKLQFTIFF

>GMOY008568-PA hypothetical protein|protein_coding|scf7180000651420:610508-612654:1|gene:GMOY008568

MRCILLWLCLLSLLGVLIRGLWADNKRTFAVDWENDCFLKDGKPFRFIAGSFHYFRAHPD

TWQRKLRTIRAAGLNAVTTYVEWSLHNPKDGVYIWQGIADLERFIELAKEEDLLVILRPG

PYICAERDFGGFPYWLLTKYPNIQLRVANVDYMSEIRTWYRRLMPKVSPYLYANGGPIIM

VQVENEYGSYACDKNYRLWLHEETLTYVEDKAVLFTNDGVNGLSCGHIDGVLCTLDFGAT

RNIKELWKELRRIQPKGPLVNAEFYPGWLTHWGEDMARTPISPIKDTFEAMLSSNASVNF

YMFYGGTNFGFTAGANNGGPGRYQSDITSYDYDAPMNEAGDPTPKYFALRDIIARYLPLP

DIPVPAPLPKNITEPYSYYAGRHHLAKGVVMADKPITFEALNQHSGLVLYETFLPLLKRD

PSVLHVPGLRDRAYIYVDDALIGILSRETPIYDLPITKSAGRKLQILVESQGRINYGYMM

DFKGLTNNVTLDKKVLRNWNMTKFPLESYEDIANLIKIDGPKWNDINESNGMLNKGPALY

YGTLNINGEIGPEDTYLDVTGWGKGLAFINGENIGRYWPLVGPQITLYIPKEVLRTGTNK

IVLIEYQRASQTNEVTFTDVPKLDG

>GMOY008628-PA hypothetical protein|protein_coding|scf7180000651460:5231-6850:1|gene:GMOY008628

MSIYENKIELIREQERFLYYHMQFLESNLYTMEYLSGLNKHCIARYRGNIPSVTSIKQTM

ELCLEMAKDNSKNLLNNAQRTVDHLNEYYVTTFLNVAAKIRNMRNDGLALLDDEVLKANE

YAHEIKRRFDNDLESAECQAGAFTKLALNCSLSVHFDAAIALVEVKSKIQSCLKGQEFCT

CKDTYACNNVKEVKPAVLNAKSILTSNEVDSSKKSKTCILLKTETEHDLINITRKAGQDE

KPEKLPSSLLAHQIQGPRVLASSNRPKQSFEPVTLPSSVLTQEIKAIEKSPTSLLAQASL

PGSRFGQQSQHSETVRSSLLAQPSLHKSLLAKQSLPAPLLAQQSSRSSLLTKESIPSSRL

ETQNLLSPLPTHQTAMLPKSPLTQQKKNSDSMVEDLPESDDEVSEIFWKSRFEQQRQAAE

SLWNSHGDFDFPDMDDGW

>GMOY008640-PA hypothetical protein|protein_coding|scf7180000651473:2776-4175:1|gene:GMOY008640

MTFKCARGNAPFGGAKGGVRIDVKKYEDKDLKTITRRYTMELLKKNMLGPGIDVPAPDVN

TGQREMAWVVDQYLKTYGHDDINALAITTGKPIHFGGVNGRTSATGRGVWKSGDVFIQDK

NWMDLLQLKTGWEDKTVICQGFGNVGSWASKFAVEAGAKLIGVQEIDCSLVNEQGINPEE

LMLYKQEKKTIKCFAGATEKEGSLLGHKCDVLMPCATRMVLTKENGDKVQAKILLEGANG

PVTPAADQILRKKKVLMIPDMYCNAGGATVSYFEFLKNINHVSYGKMSVKRESSMIHEIT

ESISQSVKQTIEPTKKLESLRDCHSETAIVDYALQTVMETAGHGIKETSNEYSLCNDLRT

AANIFSIKKFFNALKSSVIFKCDLA

>GMOY008738-PA ubiquinol cytochrome c reductase subunit|protein_coding|scf7180000651516:84649-103389:1|gene:GMOY008738

MISAVSRAYLRANVQAVPNSLKPAVLGTAQGKKVVVAPCTDKSLPVGNTRATMGLQTIHQ

VRLAHSDLEVPDFTPYRRENVKNANRRNDSAEQRKAFSYMLVGATAVGSAYAAKGLVNTF

VSSMSASADVLAMAKIEIKLGDIPEGKSVTFKWRGKPLFIRHRTGAEIATERNVAVATLR

DPQSDDQRVTKPEWLVVIGVCTHLGCVPIANAGDFGGYYCPCHGSHYDASGRIRKGPAPL

NLEVPAHTFPDEGTLVHKSAVNIHCVLLKKIGNMAPRRKLNKNILLMDKQIIDFVKEHQF

VYDRSHENFNNNIYKEDKWKQLADSLDEPVELIKKRWKTLKDRYFRLMKNGGVENYKYCH

DLNFLGPYEPRRRSIIRCKKEENYEDNSQMDESLDSSHSPVDAVDNDMFENNYTLSITSL

ATDDAKPTKLENHSIVCEEVDEIALEDVLQMQPKKRYADTYAESKPTKLENESVCSNLYE

HLLQSQQNFAQLFAEFPYIAFDRQYLVYIVTTMSTAFLTAIAVILCILFRILNVNSQPLK

PSVWCLDQQFLDTLYKIAPVLKEPYVPTRLWGFSGHMQTVLHSIIGRVKCPWPLGERVYL

SLEDGSTLTYDLYQPIKEFEDDITLAICPGIGNSSESVYIRTFVHYAQCHGYRCAVLNHI

GALGSVQVTATRIFTYGHTEDFADMINNLHRKYPGTSIITVGFSLGGNLITKYLGEVQTK

KPESIIGGISICQGYNAVEGTKCLLNWQNFRRLYLYIMTENMKSIILKHRQVLLSDESIQ

RHNLNEREIIAAATLPELDEAYTRRVHNFSTTQELYRWSSSENYFDNIDKPIIFINAKDD

PLIPEFLLEPVKNFVVSHQQACYIEVAHGGHLGFYEGGFIYPNPVTWLDRTLIAMIGALF

IVHTTPNKHKSGVPAQC

>GMOY008757-PA hypothetical protein|protein_coding|scf7180000651525:26578-58638:1|gene:GMOY008757

MSAQSYQRNALLGLDDGRDSATDGPDRLADGKKNSAGYGEGGTGARYRKGALGQAGALSV

HRMGRKQRRSSAKDDGDKSGKTGRDGYGKGGDGNGRDVKGRDGKGRDGEGGDGKGRNGRG

RNGKGGVGRDGTGKGRNGKGRDSKIGDGKNGDGKDGDGKSKGKRGQDDKSGAKKDKDGRK

GKSGKGKGEEEDEDGKAGGDKSKSDKSGQRKSGTDKDAYKWGRRNGKKDGKNGKGYGKSS

QKATRWGSKYGMGKLSCREGNIVDYLRVKMLEKDRCRCLPVCQQSPADCSYSSNMRCCAL

CYSTSYNAACLSPASYAYCQAPMQKICRRLVCDYNKAQCNERYSVFDGGNSIRLRVRRQS

LLDDECEEGVSRRGKGDESQKGRRSKAADDIGRLKGRKSGEYDLGDDKSKRRKGKTGTGE

DEEGVDARGRKKKKRGSKDGDGEDEDDIGKKKKRKGAKGGKGGDGDEDEDDTGKKKKRKS

GKGGKGGDEDEDDAAKKKKGKGGKGRRGADEDEDEDDTAKKKKRKGGKGGEEDEDDTGKK

KKGKGGKGGDEDEEDTGKKKKGKGDKGRKGADEDEDEDDTAKKKKRKGGKGGEEDEDDTG

KKKKGKGGKGGDEDEEDTGKKKKGKGDKGRKGADEDEDEDDTGKKKKRKSKKGDDEDEDD

AGKKKKGKDDKGRKGADEDEDEDGTGKKKKGKGAGGRLGADEDEEDATGKKKKRKGSKVG

KLGGDEEYDEKGTKKTRKGSKAAKGRPGEDWDEGDEDETGKKKTGKAAKGRTSLRGSTLG

RGSKKGDIRASADIYSKRYKTDREARIFDKLRAGMEEKDLTTPIDWEKCPYVPRPSLITD

PITAFGQYRSERNKRHTSILDPINRAAIKPDYEVLHQPIKPYIPARDKNRERILNMVRQH

IDTVDVGGNTAARTARDSLDIVLPRKHRAASESLPIKRETYRNEKSGALTPRLDLATDRP

GSHRTHGTSDYSYSYKSSVEKSNYDSSNPYSYRPERSSYQSSYESSARSGPGGAYNYSTE

RTSTTGGGPGGYSYSSTTSGVLPGGTRYRHYSYRYPVVTKVSRVYKTSYPIYSTTSYTRG

SRVIASPVRIVTSPARVYTRVIRSPSPVRVVRTTRVVTTPDRYSSSYTYTSPTLYSYASP

YIPTSYVSSYVPSTYVSSYTTPSSYVYTSTNTPSYYYTPISRLYTRSVTPVRVTTSPARI

TPSYLKRNLPLFGSRAISSYLGTEPYTVFHDEANRIRYKAQSLARDVHTPVTRYSRSTTP

FPVIGYEPTSKLALDAYVSRITNPVRHVAKEVHKMSMYPEPARKYVGKSHLASVRICGNR

SYDLRRPMFDSDRLRTDINLLSWYLRHPTVKSEKKVEVDEKVESVDTGNTLGDIWRGDPD

LDPYRPSRKFSAPRPLEEPEEDEEMKEKQRLRQERLMTLNEEVLDVVELEKKKAQRLDEK

KRREQKALEEEAKRKAEEIAEKKRAEAERKAKLAEESARLAEEEEKKRVEAELERQAEED

LKTAKEAEKKAKEEEAKRKAEEAEMTRLAEEAKKAEEAKRREEAKKAEEARLAEEARLAA

EAKAAEEAARKAEEAARLAEEQRIREEELSRLAEIEKQAAEEQEAELARQAAELAEIARQ

ESEIAAQELQAMQQEVKENAVAEPEQSTTAVTETAEPIVEEPVSPITTTTATAEEEIYQE

EPTIDLTVSGGDAGDYDNDMGSDDADEEDEYDE

>GMOY008764-PA hypothetical protein|protein_coding|scf7180000651536:25240-28076:-1|gene:GMOY008764

MSMISARLATSVARSLPKAAQQIGVKAAYPVATLAARKLHVSSAQRGAEISSILEERIMG

VAPKADLEETGRVLSIGDGIARVYGLNNIQADEMVEFSSGLKGMALNLEPDNVGVVVFGN

DKLIKQGDIVKRTGAIVDVPVGSEILGRVVDALGNAIDGKGAINTKDRFRVGIKAPGIIP

RVSVREPMQTGIKAVDSLVPIGRGQRELIIGDRQTGKTALAIDTIINQKRFNDGQDETKK

LYCIYVAIGQKRSTVAQIVKRLSDAGAMDYTIIVSATASDAAPLQYLAPYSGCAMGEYFR

DKGKHALIIYDDLSKQAVAYRQMSLLLRRPPGREAYPGDVFYLHSRLLERAAKMSPAMGG

GSLTALPVIETQAGDVSAYIPTNVISITDGQIFLETELFYKGIRPAINVGLSVSRVGSAA

QTKAMKQVAGSMKLELAQYREVAAFAQFGSDLDAATQQLLNRGVRLTELLKQGQYVPMSI

EDQVAVIYCGVRGHLDKMDPAKITKFEKEFLQHIKTSEQSLLSQIAQEGKISEGADAKLK

EVVTKFLSTFQG

>GMOY008788-PA hypothetical protein|protein_coding|scf7180000651573:19957-21979:-1|gene:GMOY008788

MADEKKKVPNYMKFIIGGAAGMTGTLFVQPMDLVKTRMQVGGVGTAQSEYKNSIDVIIKI

LRNEGVLKFYKGIGAALLRQATYTTTRMGVYQTINDMYKARTGITAPSTAASICMGLTAG

ATGAFVGTPAEVALIRMMVDGKLPESERRNYKNVVDALWRIHQEEGIKGLWTGCLPTVGR

AMVVNMCQLASYSQFKNALHTSRLQMDGILLHIGASCLSGLLTSVASMPLDITKTRLQNM

KTLPGGIPEYSGTIDVLIKVAKKEGIPALWKGFTPYLLRIVPHTILTFVFLEQYNRLYRL

YVLRSEGDSTIALRKNAVGANRKLLASR

>GMOY008793-PA hypothetical protein|protein_coding|scf7180000651585:38945-40744:-1|gene:GMOY008793

MLRLNASAKCIANIINPMRRYAHQAINQMLQLQHTEICADSPSRGLVLGVYADEEDKTDT

GILTPAAWRYNVSRTNGRLIEVLRMSGPMPKRGEARILFAVEVDKTPYYSAVAVVGLGKE

CLGYNPYELLDEQKETIRRSVAKACMELGRIDTSRIEVEDCGHAESAAEGAALGIWAYQE

LKSRAARITIPAIDLYTTKESVCDLEGWRIGLQKAAAQNLTRQLQEMPANLLTPTTFAQN

VVEVLCKSGVNVEVKVEGWAESQVMNAFLSVGKASCEPPIFLELSYYGTSPEERPIVLVG

QGVTYDCGGLCLKDVPTLKWMRGDMTGAAVVVAACRAVAALRLPVNIRGLIPLCENVMGC

NSFRPGDTVKSMNGKHIKINCTDHEDILVLADTLLYAQNFCPKCIVDIGNTSPWSHDVLG

EAASGVYTNSEILWQQIKHASMHTGDRVWRMPLWDYYTSQVTKTDSADIANYGAGRGGRP

CKAAAFLREFVPCGQWMHIDSTNVMNTKGTAFEYLRAGMAGRPTRTLIEFIAQTICKDTA

PKFPKKQI

>GMOY008908-PA hypothetical protein|protein_coding|scf7180000651686:33518-35317:1|gene:GMOY008908

MADEESAPEEPKQDAPKEEEAPKKEDAPKEEGAPKEDALKKEPASKEGVEGAEKKAEEEP

EGEDKDKKKPVIDCPKPEPKKTLKEFQKEREKEKEEKAKEKEKKEKEKKEKEKKEKEKEK

EKKEKAKAKGTDAKEETPPKEAVDKEKPVKQEPTKEEEKKEKKKKKKKKIPKKEPPPVYR

RLDWTFYPTLCEIYNWMDVIVEKCSKVVTKFEIGRSHEGRLIRGLKISFKSGNKAIFIES

NIHAREWITSSAATCIVMELLFSKDPDVRRIAAGIDWYIVPVFNVDGFVFSQEKERLWRK

SRRPLEEQETPQSPESPACIGVDLNRNFNHQWGVIKNEPCTDQYGGSEAESEPEVKQLAQ

FIDTIPEGTLKIYIALHAAGQAVITPWAYTKEEQPKDHKELMEVAKAFVEAAYPRFRTKY

IYGPAAEILKVGEFCGTSTDWAYGVKNIPISLGIELPGKGRYKPFELPTESILRVSIELL

DGLVGMIKAVGELGFIAKIPEPPKKEIKPKYERPKKAAKKKEGEAEEKPKPKWITD

>GMOY008910-PA hypothetical protein|protein_coding|scf7180000651687:51071-53853:-1|gene:GMOY008910

MSFFGSNRDPTIVKKEPFPPALIHQRLLHMPVIGALDRDAMFYMLCFVLNLLRLSPDSQK

DVLKKFRERNMIDNEILIYMQLISRHPNTGLTNEQRDHVIRITQDIINEHSTMSSNNYFN

PDHIFKKSFVEIDPYELEYLKSIMHTTEDLTKQQFLYLTMLVERLQEMPLAKRDQYLKLL

HSKGVLPSEKFTTLSWYLSDKAKRFDSTRLKTLFQELETLMDIAETMPPIRPAEIPKKYI

STTEIKTDAYKVTQTLEALMKGAVENIVRLSELKNFLKNLMSLTAYELESTLNSLKNSYV

LDKNQFQFLIDFIADGRIRGDPKKLQLLTDMLEKVSGTPIGTQVPTNENAAMGQGQGGAK

KKLTVIKAAEPIIAAVASKQRMLAMWSIQSLNQQGATGTDPTVTGIGEAAVAGKNLEQAL

AEIDPTSSAAKAKAPAATKIFEKTWAETDATHTEKDEAAAADKHLKQTLAQRSTTSTGKD

AATPGKGRSRKEVEKGSAIAKQENISLDEYSTDISAYKEDVGGDGDVKRDDDAKAGHASK

HQNFKKIGAGDQKKKGDKGQVKRGIPVTHGLPAPRIVKSEMSAASILSAMKLDTTKNVKK

EKDKSQRVDYFEYPPPLYRSTHKEFMLEGPNGPIPLIRNRALFFHFCTRSGKTNRIVSIK

NGVLRFR

>GMOY008955-PA Thiolester containing protein IV|protein_coding|scf7180000651715:109449-119569:-1|gene:GMOY008955

MKLSFIEDLEILIDTSTAGLYITGSFFLKYSVIGPGTIHSDGKYTVAVAVHHVAEPCQIQ

VGLTGPSYNDSKIVELSGFEVKNVDFDLPVLERGDYNLTAEGLNCMKMFKNSTKLNYSKF

HTNVRVQTDKGLYKPGDVINYRVIFLDKNLRPDKPIKEAKIYVEDGKRNRIKEIKDFNVV

QGVYTGKFQISEYPVTGGWRLGVSNGGRYDHMVYFDVDKYVLPKYVVKVESTERVSVKDG

DMQVIVRANYTYGKPLNGKVTLLVNLNVNRYYYRGDSETEETPKNPPTIIKTAPMIQGKS

KIDLDVKEYEAFMDSKTSPSYLSIVATVEEEFTGVKINATSGSTVYPYRYSMNCISYDTC

SVFQADKEAEVEFQIVYVDGTHLNDTKSPVELIYTEVLNKYRVWYPDSDEENSKEADTEP

VSENRTFHFRSHMNESSIAVFKVSLPDLRDYRKHAHFYKMELKYRDEQRELYSTYQYREP

KNLDPLSAEENDKLKEFFQLEYKRYDDKIEINKESQFTVNSSQPLSYVVYNVVGRGNILK

SDRIDLPDKPKFHNISLTPTEMWAPNFALYVYYVDEKGEYHYAEQRYYVQYRLQNQINIT

APEQVKPGENVSLKIKTAPNSFVGLTAVDQSVLLLRSNNDLRPHEFDWVLSSYTTTTPHQ

GGYSDYPGWSSGVVTLTNADYFYNWTKPEYLSTPLSAQLDSELNNRIFTKSGVQESGILG

AAGRPAMAEADSGSGFSASSAEVQVRKDFSETWLFDNIESTNEEEFTYVTKIPDTITSWL

ISGFSMNPNKGLGITADKTKVVTFQPFFISIRLPYSVKRGEVINVPALVFNYLNKDLDVE

VILDNNDGEYEFMDITNEVHNDEKQVKKVVRVPAHGAAGVSFMLRPKIIGNVMLKYLAKS

PLAGDAIHKTMKVVPEGVTQYANRAYFVNLKKESEEKTNFKLELPDDVVPDSQYVEVGVM

GDLLGPVLNNLDNLVRKPAGCAEQTMSKLLPNYLVMKYMQHINQLTPGLEKRLLYNIESG

YQNMLNFRLKDGSFSAFGLPQYYRDEKKPTNGSTWLTAYIIRSFNQLKEFVNIDERVINE

GLEYMVKNQAKNGSFIDKGNFYYGGSRDVISLTSTVLLAFLENKTIADQHKDVIQKGLDF

ISKNIDKPKTLKDHILGTYTLHKAQHPQAEEELIKIKNLAKTEGDRMWWSESDDRPKTYY

FFSNDVEITAYNLLTLLDESSTTVDDVLPIIKWLIAQRNSYGGFSSTQDTIVGLQAIIKF

AEKADYKAAKMDIEIEAKGDMPKKETLHLNEENGILYQTLELPAKTSNIEFTVKGAGSAL

VQISYQYNIFEKAPQPSFSIDTQKHDSSLSGKLLMDVCVDYIGEGDSSNMALLEISLPSG

FVIDEDSLENLKQIEGASMKPYFTVFFKQNIEVKNSASLLVIYFDHLHKNRQKCVPIEAF

KSHAVAMQKPASILLYDYYDTNKKVTSFYEVASKLCDICDGEEECTNCEWQLNAAKRHHM

ETDYLWMMSIPNFPVFFTNAIEQSNQLKRTYVKKLLRVIDDDDIIADDADDAS

>GMOY008974-PA hypothetical protein|protein_coding|scf7180000651717:275627-313125:-1|gene:GMOY008974

MTDCHPVITTTHGCVKGLWKCSVYNDNYYSFEKIPYAQPPINNLRFKAPQPITPWAEVRE

CYQLDVKPLQQNYFKKIVEGSEDCLYLNVYAKKLISSKPLPVMIYLYGGAFELGDATTNN

LGPDYFMMEDVILVTMNYRLGPLGFLSFEDPSLDIPGNAGLKDQLMAIKWFKENCLNFNG

DPDNITLFGESAGSAAAHLLMMSEKTRGLFHKAILMSGTINNKWACVPPGNYAYRLAKAT

GYEGEDSDPLILRHLQQIPSAKLTSVDVLTEEEIFNDVAFVWGPIVEPYDSPSALITRSL

DKLFTTSWSNEIPLILGGTSFEGLLKLPSIKKRPQRIEILNEKPELIVPQDIRLKQTLEE

SQSLGRQILKMYFHNKDLNIENVLNYLEILSYTRYWIGFQFTIFQRLRYATRSPTYMYRF

DFDSPTFNLYRLKYCGREVRGAAHADDVSYLFYCRRAWKLPLDSLEYLTIRRMIGMWTSF

ANTSNPNCKEIQSSEWTCVSDKQPFAALNISDEVQMLKELPETENSGSVLRKLLSAMSAN

IYQVITLPVGQIRGIQQQTIYNDEYFSFEGIPYAQPPVGELRFKAPLPAKPWSGVRDCAD

FQVKPMQKDPKTGLAVGSEDCLYLNVYTKQLHSSESLPVMLYIYGGGFYKGEGTRYLYSP

DYLMRANVVVVTFNYRLDSLGFLSLKDRELQVPGNAGLKDQVLALKWVKQYIKYFNGNNE

NITVFGCSAGAASTHLLMLTSQTENLFHKAICMSASALNVWACKPANDYAYRLAKCYGYQ

YENNDRLVLEFLKSLDAEKLVLHNIYNEENLGQELAFTPTIEPYHSDNCIIDIEPEAMLP

TAWGNQIPLIVGSVADEGLIIYRSLKNHLKKLKISQEEPESLIPQIIKSKKNSELYKKLI

EIHFGHEGPKENMMEEFIKFYGCFIINYGVQRLITSRLTYSKAPTYRYRFAFDSPTFNHY

RTLFCGNDLKNGVPHAEDLSYLWFSSSSWKLDSNTLEFKMIKTIVDIFTSFSQTSDPNCF

ALQPTKWKPLNPSDNYLALLIDEDIKFTSTVELSISKMSRHLTASFSHFGNLRAVQFNAY

SLVKHLKFAIKQFHLTNSKKTTSLVMQSGTNDEILEISQGLIKGLKCTSLYDQIYYSFEG

IPYAQPPVGSLRFKSPVPAGGWLNVRNCTEFKVKPVQKNNLGIIEGSEDCLYLNVYSKDI

KSSQKLPVMVWIYGGGFSTGSCVKDKFGPDYLMHEDVVVVTFNYRVSVLGFLSLSDPALK

VPGNAGLKDQVLALEWVKKNIVHFGGDSNNITLFGESAGAASIHYLLSTNRTRHMFHKAI

CMSGCMLNNWAFSYDAPVLSSLVACSKGYKGPKDNERLILEFLQHLPANELIDVDAINDE

VRARGYLYAFMPSFEPYESDDSIITQPLWASMKTAWGNEIPLVIGGTSFEGLLMYPRLKK

RPEIMSRIAQKPEKLLPQDISLIGQRKQVKGLSETLRETHFGDKELNEENILLFLDYFSY

RLFWHPLHRVIAARLKYASASTYQYCFDFDSPTFNHHRNMFCGKDITEGVAHADDLSYLF

YSYYSRPLNNDSMEYLTIQRLIKMWTTFAKNSNPNCVYVNDWEEVTTAGIYKWLNIGSDL

IFGDMPTCVKNKFQVWDNLYGENLVEFIVYRVQQYYLTTNEHEIVNTLSGRVKGVKRKTI

YHKFYYAFEGIPYAKPPIGKLRFRAPQPAEPWKHVRDCTNCRSKPTQRNVLERFVQGSED

CLYLNVYTKKLNSENPLPVMVWIYGGGFQLGEATRDFYSPDYFMFKDVVLVTVNYRLGVF

GNFNDPELDIPGNAGLKDQVMALRWVKDNIQNFNGDPNNITLFGESAGAVSVHFLMLSEQ

GRGLFHKAILQSGCTLNSWVSTALADRNYRFACSLGYRGNNNDQDIYRFISKLEAKRLTN

PEPRLLNKAERFNNILAIFLPVVEPYETHQCIISRSYKDCLQEAWSNHIPLMIGGVSSEG

LLCKNVVELYPFLIDDLGNCSSLLMPETIISRSPQEIEKMSLELKRAYFETSKLSAKQNL

YEYLHLVSHRSFWHGIHRVILARRKYASVAATYCYRFDFDSKFLNQIRIIKCGSKVNGVC

HGDDLCYLFYNVLVTGLDININEYKTIQRMIAMWYNFALNSDPNCPEIQPTKWEQLHKHP

EAYRCLNIGEELEFIELPDYNKLKKEYVTLELPQGRIRGYKAQALYGDCYYSFEGIPYAE

PPLGSLRFLSPVPVKKWSGLKDCLDFANKPLQKNEWGLIEGSEDCLYLNVFTKNIQPVKK

LPVIIWIPGGGFVTGGCPKEYIYGPDYFMMEEVILVTFNYRLSVFGNIIFTTICISKYIL

LFSRFLGFLSLCDPAAKVPGNAGLKDQVLALKWVRANIELFGGDERNICICGDCAGAASV

HYMLSTEHTNNVFQKAICQSGSFLHNWTVGYHNIEMSYNLACRKGYKGPRENDVLILKFL

QSVPAEKLIDIDDLDMIARYKGYLFAFLPSIEPYECEDTIISKPVWEVVKKAWGNKVPLI

VGGCSFEGLYFYPILKKLPDFLDHIIKNPVKLLPSDMPMDCEEKECAERCEELAKTLTKV

HFANKLVGRENILPVLDYFSYRLSWHGLHRFVLARIKYAKKTPTYQYIFDFDSKDFNHHR

DLYCGGDITEGVAHSDDLSYLFYSFYSGRRLNEKSNEFLTIQRMVKIWATFAKTSNPNCE

YISNWEDVGSAGENKWLNIGSQLKFVDVPTKILEKFQVWDNLYAQGCIRGLKLQSIYGDW

YYSFEGIPFAEPPVGRLRFKSPQPIGCWSGVKDCLQFKHKPLQKNENGEIEGSEDCLYLN

VFSKNISTKEKLPVLVWIYGGGFGTGGCIRDYIYGPDYFMMEDVVLVTFNYRLSMIGFLS

LGDPDVEVPGNAGLKDQVLALKWVKCNIGCFGGDPNNVTLFGQNAGGSSVHYMLCTERAR

NLFHKAICQSGCMLHDWAISHDAIELTYLLACRKGYKGPRDNDKCILEFLQAVPAEKLID

IDDLDTVGRYKSYLYAFMPSIEPYENEDSIITRPIWDLIKSAWGNSIPLIVGGTSFEGLF

MYPLLKKLPEVVERLKNKRVKLLPNDVPVECTNMAKILTKLHFGDKQTIEESVCPLLDYY

SYRMIWHGLHRFLLARLKYAKAPTYQYCFDFDSPIFNHHRELLSGGDFTEGVAHGDDLSY

LFYSYYSSKLDKYCQEYFIIRRKDDSYVHRCESMKLDEDEFGENTEVVLTPSGSVRGLKS

ISIYGHEYCSFEGVPYAEPPLGQLRFKSPVPAKAWAGIKDCRYSKSKAMQKNSQGVIEGS

EDCLYLNIFSKDVHSKTKLPVMVWIHGGGFSTGSCIKDYICGPDYFMMEDVVLVTFNYRL

SMFGFLSLCDPSVCVPGNAGLKDQVLALKWIKNNISYFGGDANCITLFGQDAGAVSVHFM

LSTDRACNLFHKAICQSGCMLNDWAISHDAIEMSYLLACRKGYKGCRDDDICILKFLQSV

PAEKLVDIDDLDTVGRYKGYFSAFMPSIEPYESDDSIIAKPIWELIRSAWGNNVPLIIGA

TSFEGLCIYPVLKKIPELLERLKNKWERLLPNDVPAWDEQTKLIDSLMAVHSIDSDISRE

NMCALLDVILLLSLILAWNASFLLSRYKYAKADTYQYRFDYSTDNFNHHRMIYCGNDVSS

GVAHGDDLSYLFYSYYTIKPKPETCEYSMIKRMIKMLTAFAKTSNPNCECICNWSNICEA

GIQKWLNMSDDWSFVSMPYDVQEKFAAWNNLYGEYLVGERSQILKTPQGMVQGLKLESVY

GDSYYSFEGIPYAEPPVGKLRFKSPVPSKKWFGVRDCCRFTCKAIQKNQHGIIEGTEDCL

YLNVYSKNIHCAKKKLPVMVWIHGGGFTTGSGIRDFICGPDYFMMENIVLVTFNYRLSMF

GFLSLSDPCVKVPGNAGLKDQMLAIRWVKDNICYFGGDSENITLFGQDAGGASVHYLLSS

EHARDLFQKAIIQSGCFLHEWALSYDAVQLSYLLACRKGYRGSKENDECILEYLQSVPAE

ILVDIDDLDVLGRYKGYLYAFLPSVEPYESEDSIITKSVWDLIRTGWGNNIPVMIGGTSF

EGLCMYPILKKIPELLERLRKKRERLLPNDIPCGCDYLNLTEELISAHFRENQISEEDIL

PFLDYYSYRLFWHGMYRLLQARSKYGKASTYLYCFDFDSPTFNHHRTMYLGNDVYRGVAH

GDDLSYLFFSCYSSVLDTTAREYFMIKRMVRIWSAFAKNSNPNCEYVCTWDDINTSGLSK

WMSIACELRFIDIPANVQEKFKIWNNLYGSRLIE

>GMOY008993-PA vacuolar H+-ATPase v1 sector subunit A|protein_coding|scf7180000651745:221590-223660:1|gene:GMOY008993

MSNLKMFDDEEHESKFGRVFAVSGPVVTAEWMSGSAMYELVRVGYYELVGEIIRLEGDMA

TIQVYEETSGVTVGDPVLRTGKPLSVELGPGIMGSIFDGIQRPLKDINELTSSIYIPKGV

NVPSLARKQSWEFNPLNVKVGSHITGGDVYGIVHENTLVKHKMIVNPRGKGTVRYIAPAG

NYTVEDVVLETEFDGEVTKHTMLQVWPVRQPRPVAEKLPANHPLLTGQRVLDSLFPCVQG

GTTAIPGAFGCGKTVISQALSKYSNSDVIIYVGCGERGNEMSEVLRDFPELSVEIDGVTE

SIMKRTALVANTSNMPVAAREASIYTGITLSEYFRDMGYNVSMMADSTSRWAEALREISG

RLAEMPADSGYPAYLGARLASFYERAGRVKCLGNPEREGSVSIVGAVSPPGGDFSDPVTS

ATLGIVQVFWGLDKKLAQRKHFPSINWLISYSKYMRALDDFYDKNFPEFVPLRTKVKEIL

QEEEDLSEIVQLVGKASLAESDKITLEVAKLLKDDFLQQNSYSSYDRFCPFYKTVGMLKN

MIAFYDLARHSVESTAQSENKITWNVIREAMGNNMYQLSSMKFKDPVKDGEAKIKADFEQ

LYEDLQQAFRNLED

>GMOY008996-PA hypothetical protein|protein_coding|scf7180000651751:1544-10805:1|gene:GMOY008996

MKIEITLLIIACTASMVLARPQEPIAIVSQESNQEPDGSYRYSYETANGIKGEETGTLKK

ATSADTSDVIVASGSFSYTSPEGEQISLNYAADDENGFQPQGAHLPTPPPIPPAIQKALD

YLLSLPPTKRR

>GMOY009019-PA hypothetical protein|protein_coding|scf7180000651758:461419-462940:-1|gene:GMOY009019

MSSIESKRVQYRKYLERAGVIDALSKALIKLYEEQNKPDDAIRFVRKFMCESCPDDTQFD

AMKADLEEANKTISKLEQDLERLKDQIKKSPEEIAELLEEGFKKLVEDEEHTGSLLRKYL

TREILDEYLKLTTPPPVEASLLDCIQSGLTFHNSSCGVYAADLESYEMFNKLFDPVIRDY

HGQLESDKEQLQPEAEFGNPDEVENMDPEKKYILSTRIRVARNIEGFPFFMKLREKQFLE

IEEKVKAATDAFDGELAGAYHSLKDISPETQEEMVKRHILFRRGDEYLQAAGCYRFWPVG

RGIFYNPAETFLIWVNEEDHLRIISMAKCGDLGDVYNRLIKGLTDLEKTINFMRHPLYGN

VTVCPTNLGTTMRVSVHIRLPLLSRDQDRLNSMAAELHLQIRGTGGEHTAIEDGIMDVSN

RRRLGVTEFELIKTLQEGILTLIKAEQELEAKE

>GMOY009073-PA hypothetical protein|protein_coding|scf7180000651758:1032630-1036649:1|gene:GMOY009073

MAKETPGPASLYGLPPTVGYENHDVRKQRLPKYSFGVKTDQKILDVGPGPARYNVEKLVR

YGVSRANHFTIAPKTKIIDKVKSPGPQAYAVHKFPIFKGSRAPAYSMGKVNTFEFKKCAP

GPNKYGIKDNFIRQRAPAYTLGVKKYLPELVRSPGPAGYPAASLNKVKPQSPKYTLTPNN

KFIRKLYGPGSNYYNRMNYKPGKRAPIYSFGIRHHSKTRPMIVPCSEVKAKRNRSSNYSI

IVPPSDKPRSPGPAGYGPSNISLIRRSSPQFSMSSRFGPRWNKSWVPASNHYNCMNYQPG

KKAPSYSFGQRISTRSPPLIIPD

>GMOY009095-PA coracle|protein_coding|scf7180000651771:252201-263590:1|gene:GMOY009095

MPAETKTPTANETESPSKGKKSSSSSVSKAALARVTLLDGSILDVTIERKAKGRDLLNSI

CAGLNIVEKDYFGLTYETPTDPRAWLDLEKPVSKFFRSDPWPLHFAVKFYPPEPSQLQED

ITRYHLCLQVRNDILEGRLPCTFVTHALLGSYLVQSEMGDYDAKEMPDRSYLQDFKIAPN

QTAELEQKVMDLHKDHRGKSPAVAELHYLDNAKKLAMYGVDLHPAKDSEGVDIMLGVCAS

GLLVYRDKLRINRFAWPKILKISYKRHHFYIKIRPGEFEPHEVTIGFKLLNHRAAKKLWK

SCVEHHTFFRLMTPEPPSKSSLFPRFGSRYRYSGRTMYESKKNPVDREAPKFDRSLSGRR

LTSRSMDALALAEKEKDAQKRHTMAHPPDHIPDLDSPRSRSPIKKDKKEKVKLIRESSTG

TASASSQSSLEGDYETSTTGAAADVASAAAAFLADNQEAEKEAKLREKKQKEKEEKERKE

KEKKEKERKEKEKKEKEAREKASKEKPEKFTAGTATEGLNGNENLNDSQKSGKQKGGGLF

LSGRRSKSGSPTKEVKDKERSAKDKLGKEKEMVPVVASDTSAPGYVKPYEYTDGDGEISP

TRKSYIPGGFRYDHDPDSAKRASEGQDQLSPTAQQKKIGLAFNYAPGNEEALKKTAEKLK

SGQLSPRTRDKLNKGQLSPKTREKLLKEVNLSPTTRAKLQGSAVDAAAEPLRDTQKRSYS

PTKGQMQGYSSGAPGSYKPLMADPTKAFLDSERYNREQGIDTSTPSREGKSATASPKSIG

KGSKPVAPPVIPAKARAATPAKPKKKRVKIMVITSKFDPSTKRIDSESGAIEHSTGILDP

ATGLIDTKYGVIHPQKGTLLALNTKTGENETYQGEIDPKTGNIHLINGVSDPQTGRLNES

LGQIICITPQEDPVVELTVITSRIDPNTGKIDIVNGEVERSLGVLNMETGLLDTKYGEIN

TRAGELKIVDPKSGKMVVTKNVKIDPATGQISILGIIDPKTGKLDPNQGRLIEAGQQIDP

IVEVTSLSGKYDAKKNVIDSKTAQLETSGGQFDPKAGKVDTKYGQIDLVKHTITFTDPKS

GKSVTRDIKIDPSTGQIVLKNQINPKTNKPDKDYARIICLRIVQQRVDPKTKNPISQIST

AKDKDVIVDPKSNQIWMPTGAVDPNTKEPQYVSSSVDPKTGYVITIYGYLNPKTNEVKKQ

TKLDPNLFKVEPSSGKIYTATGDVDAATGEPVYATTQVDPDSGEVYTKLARVNPKTGKIV

LIRILLISKVDERGRPEELDPETCEIDPVSGRVLKFFNKTVYVYNMIDPITGEIVQVDPN

DPRFAGARTTVTHTMTLTGEIDPVTGRIKSEYGDIDPNTGDIDPATAIRDPVTGKLILNY

AQIDPSHFGKQAQISTTTETVPITRQQFFDGVKHMGKNALRRDSEASDSEDDMTHEYETE

NVKDIVIGGTKQSAAQNKYVTTPTVVKTTTKQVLTKNDDGVTHNVEEEVRNLGTGEVTFS

TQEHKADTPAADITSGAYVTATAVTTRTATTHEDLGKKAKTEQLEEKTVATTRTHDPNKQ

EQRVVTQEVKTTATVTSGDQFQRRESISSTSSGDSGTPIDGPYDESGTVQHRTSPNSYKQ

PLIHQTSATAGTASMGPHVEQTRVVLGEHTPGYSGHGEIVSTQTVSSKTRTVETITYKTE

RDGIVETRVEQKITIQSDGDPIDHDRALAEAIQEATAMNPDMTVEKIEIQQQTQ

>GMOY009161-PA Chitinase-like protein Idgf5 |protein_coding|scf7180000651771:2339108-2340567:1|gene:GMOY009161

MRNKMIYFNFHLFVIIFANLQIFQVQAANIFCYYDTQRITDVNAAINYLEPALQFCNFLI

YGYAGIDGETYQVKSLDYGLNYDIYQAITSLKLKHNRLKVLLSIGGDRDQTEDLAEDNKY

LKLLENLSSRNAFINSIQSVIRTYGFDGLDMAWQFPKNPPKHEHSGFRKYLDKLMNLFRR

SPVIDENSAFHKEQFVSLLTELRQSLNPMGAIMTMTVLPHVSAELFLDVKPIVNHVDFII

LATFDYLTPYRDPTIAHYTAPIYAVSEHDPSHNINYDVQYWLNHTTATSKLVLGVPAYGR

SWTMIKKSGITGHPPITAGGPGRAGHRTLTAGLLSWPEICVKIHQNKELEGDAARFRKVS

DPTKRFGTYAYRSVDENNEYGIWVSYEEPKTAANKAEYAHARNLSGVALFDLSMDDVTGE

CGDGTYSILKSIHNAFKKFK

>GMOY009277-PA hypothetical protein|protein_coding|scf7180000651837:12368-19419:-1|gene:GMOY009277

MKLMLVFGVLALAACAIAAPQKDVEVLEYEADNIGIGGYKFSYKLSDGTTRTEEAVVNNA

GTDNESLSVRGSVSWVAPDGQTYTVNFGQKYGSHNSFGDAVGMITADFLSRTDGRGFDIA

QLRYAICLVAFGAFHIYPETVLNSTIFEEIMKRGLLLCSASQKDNHQLVSPPDVYGPHEQ

EVLRDLGFNEFKFHVELVNIPYKTLIHIMKRSDGGDMRGTVLVKRIKSALASAVRGHTYY

LLCVNESYLMIWLNERVYFIFDVCGRRTTDVTTDEDEGVPMLIGLKTLDNVNHLILNLSG

LNEVDPCSIRGLKVIHLVSPSGNIIQRSYGRSPPGKINNLYKKFCFIF

>GMOY009348-PA hypothetical protein|protein_coding|scf7180000651840:1262560-1264989:-1|gene:GMOY009348

MLSEKFKNKWNKKNVGESMSFSKEQADGVLREQDKIAEFLSAKDVAPWFRGTSVFHLEGA

NRLFNAIRDDTEQSTSHRVRACLSLLGLDPIISSKQIKFLMRLSRGNDLAFLWFLMELHY

HTPKPHFSVNEQLICSAICHLDMITTLRELDRILPENHKIKRRKASSRSEKPKSISTDTK

PKKKYILPYFERLRKPKPYGKSLVLKLPNFSVDVNIYKPYTDPNYVVPNESNRWYANYQF

QTSKRIANRIIRDEIEEMFKDLEGAKLNASQIHVICDYHKTLKEIEVKMHDELQVKLRDE

CLLKYFGSLKKDKERAEAMKKKLEEEIEKQMEIFRKDARTIRKQMTLKTLTSECEIIDVM

LGEEDVEICKKTGEELQAKKKCEVAPEKCKDSSDKTKKISLLAKQSHTKGIIDEADAMSI

LSKEQLINVENLNPRVSGRSSRGRLSHVSNVSTFASTYMRPLDYGHICVTPTESQTFFQA

PKNHKPFSFDYRKIFEFKDKTSADEMKLKNAFLEALDEDISNLTELQKAKMNVEESARKC

ADKIWLDSVKERLDKDADDLLFRESLGKPLVQYPDNAYYDAKDKVLMEKMLFDAFEYLRK

NPKFVWAQLPQAHKVPMLREWIARRYGKVYTTQERWRSYKISLKIFQALYTLDMSVPPPT

ATKLGHNMFLSYNCRDYIDKKGRAIKNAYNRRLNENIMEQSRVFWFAMRGHLCSSGPPRN

TFFAYMPSRFRDIQRFRLWKPNEYRNYKRAYDKRRINK

>GMOY009357-PA hypothetical protein|protein_coding|scf7180000651840:1319933-1321145:1|gene:GMOY009357

MERYAPVAKDLASRDVVSRSMTIEIMEGRGVGPEKDHVYLQLHHLPPEQLAQRLPGISET

AMIFAGVDVTREPIPVLPTMHYNMGGISTNYRGQLLTIDNNGCDVIVPGLYAAGEAGCSS

VHGANRLGANSLLDLVVFGRACAKTIAEVTKPGEKAPTLKDNAGEMSVANMDKLRHSNGK

IKTADLRLKMQKTMQAHAAVFREGSLLKEGCKKMCDVYQQFKDIQVVDKSNVWNSDLVET

LELQNLLANARMTIVGAENRKESRGAHAREDFKQRIDEYDYSKPLEGQQKKPLEQHWRKH

TLAWICDDKGTVEIKYRKVIDETLNESVKSVPPAIRSY

>GMOY009456-PA hypothetical protein|protein_coding|scf7180000651846:508780-514484:-1|gene:GMOY009456

MEYQVIPVKRFDEVIEHLRQNFFADEPLNKAVNLCKRGEGHKYLEEHSLKTLEANLSVMA

VSDANEIAGVVLNGILCPGDLQTAKKKLQTKDDEKYRKIFQLLYDHNLQTDIFEYFKIDK

AFDMSILSVDEKFRGKGIAKHLVENSECLAKKHGFKLLKADATGVFSQKIFKSAGFEVLH

ELYYNKYVDNDNEIILPIAGVAINYIVCSGDLENARDQLHTINDENYRNILKLLYDYNIE

ANIFEHFKVDAFFELHMLSVDAKFRGEGIAKHLVNDTENLAKKDGFKLLKADATGLFSQK

IFKSAGFEVLREVLYNEYVDNNNKPIFPVPPPHTKMQLVCKRLD

>GMOY009493-PA heat shock protein 70A|protein_coding|scf7180000651846:1400359-1402281:-1|gene:GMOY009493

MVAIGIDLGTTYSCVGVFQHGKVEIIANDQGNRTTPSYVAFTDSERLIGDAAKNQVAMNP

KNTVFDAKRLIGRKYDDSKIQEDIKHWPFKVISDGGKPKISVEFKAEQKCFAPEEISSMV

LTKMKETAEAYLGHTVKDAVVTVPAYFNDSQRQATKDAGAIAGLNVLRIINEPTAAALAY

GLDKNLKGERNVLIFDLGGGTFDVSILTIDEGSLFEVRATAGDTHLGGEDFDSRLVNHFA

DEFQRKYKKDLRSNPRALRRLRTAAERAKRTLSSSTEATLEIDAIFEGIDFYTKVSRARF

EELCADLFRQTLQPVEKALNDAKMDKNQIHDIVMVGGSTRIPKVQNLLQQFFCGKSLNLS

INPDEAVAYGAAIQAAILSGDKSSEIQDVLLVDVAPLSLGIETAGGVMTKIIERNSRIPC

KQSKTFTTYADNQPAVTIQVFEGERTMTKDNNLLGTFNLTGIPPAPRGVPKIDVTFDLDA

NGILNVTAKEMSTGNAKNITIKNDKGRLSQADIDRMVHEAEKYADEDEKHRQRIAARNQL

ETYVFGVKQALEEAGDKVNSSDKNRLMEKCTETIKWLDSNTTAEKDEFEYKLEELTKICQ

PVMTKMHQQQGAGSGDGSRSANCGQEAGGFGRGPTVEEVD

>GMOY009538-PA hypothetical protein|protein_coding|scf7180000651846:1955551-1960318:-1|gene:GMOY009538

MNETRIFCNSSGALVLDLENGKKGGKGGKKDKKDKKGKDKKGKGKKGKKKAQVKPVVKEE

PPPPPPPPPPPVEVKIKIEEPCVFNCAQDKVTYRPNSYPGLLLHPKTLAVVGSECGSYDS

LCKLLHAGFRCADRVYTMVPWGNYVVFRSQDGDNYLYFVFDGCTCNVNRFRYLDLTCGTA

SLLYFEELHKAINYIIMSRSRREELKNLKRSMVDDVCSEMYGTQHI

>GMOY009539-PA hypothetical protein|protein_coding|scf7180000651846:1968477-1969202:-1|gene:GMOY009539

MKNCIFIYLGVILALVFQTCLTSPVPDSGDSPSAEDKQLNEHNKLYKEVLKEFTDFTIEM

SEDFLEFTTKITEEIKQNNELPDIEHPNRRQLEFTKALEHVKSSEGDHTLLNMYKLTEDV

LEATRTLDDPKFLTEEREKLIEKYKIREFMNKIRERYMKFYESLSKAVDVYADELSETQK

QEQHKLLDWNKDFSETKDFRSKTDKFADFFDFFKPCCEDL

>GMOY009618-PA hypothetical protein|protein_coding|scf7180000651846:3826897-3835184:1|gene:GMOY009618

MNNTRTAVLCVYECHTGLELKRPKSGIYINETEPPNALPSDEIPYSTYNPRVLQEQFDSQ

NLKRIDLIDGPQADLWTWNLGVQEKEEVLDKTFIDAAIAKFDCSNAELVVFKIPTDLDKT

FSAGIVFGNKAVKAAEPTLKPEFYYRPKEMACLYVAFVEAFRLNADFWTAETMDLIIDMG

GKLVEKSKKMFYKSPDLPFDIIPEITERDALISLKIHFTGPLKSQPNIYKALSLYFSKYN

AGILCSKKLYLLIWKRCKNYYYVYDPNGRAENCERDFENGKCALMSTHFIEHLVHLIVNI

SQTNMDDEFSLYGIILENYGKISDPLPATPFKKLTRKQWATINEAYALIPSCNYGLTQPN

IMPEPNPSMLIAVMALLYNYIERPNTWDTNSVDQIIRIGTAYYKSLRRSMKMKEKDHVNI

LDVPDKYVLGQYKASMKKKPFFYTGTVTDYCKKFVDSLLACGLKELFSSEWEAALIQIDS

SVVAVWRDKELYYLFDPFKRGRSGQVIDPDDYKTDGAACLQMHANFDSLLWLLCQNALKM

RRGAKFFIHAVKVGCIKPILDGKYRKMRYTGLKLMPDVINRDAGGEDINAAGGDKAGKKK

KDKKKGKKGKDKKVQKVCSIVTVPDNDRLPGFENIDIVDGVLEDILCELLRNLPEEDYAR

PRLYKSANRVLLRSDKEYLESLKYMVTHDQDFDNYNAAFPTQRDQDTPILTLEDELAIPS

NFQNLPDGTWIIFGKQSLPRVDDEQTKLKGLLSALVTAALVTKYKISTWTSELVDYAIQA

VDSFSEEFQVYQYALGAFLSKKLPRVTIGQRTYDIRVHKTIKSNIQKSLRQVLLESLINY

NRMIVICQRFCCLIVKRYNFLYMFVGFPVNAVGYRKNNAGPACLLRFVELDSLIRRIEYG

CNPQGCDITHYVVVALKVVDSTPEPFGRYRAWPSDQQENLYKTELDYYKKAEMGKLAKMQ

FLDSELSKENARIKEFLDAKAEYQEDRKGKRKAVRKDKPKPRPPPTDESQLDEEAREEGG

QDEEGQEETVAEEDPCDIKPKHKFGTKIPKSKDEFVRPRPVVYGYRLREKDYLYKIQGSK

ALSNRTECLLDKIKPCFFASTMAILYTILRPLNEWTSQRIDQLIDSATILTEKLEDMSTS

YERVLKNVSVDEYTFNIMIRVFEPMGMGINLRKQLERATTMRKYMMLHTANCTYTIFRDE

YYHLFDPYPSMETTSQETEKEEEGTTDASKQKMPKLPRGTKKYGERNTASWILFADIQSM

LQYMDKRSCSPTWKEDYEYTFHVMDIISYKKSPQNTHVLTLLTGMAPCQTNDVEETARCA

HNESIAWLEHCLPVWSRLNRRNAAGRYRSMGISKFKKYDIEIEARLWSLWGTLHPSAPVF

ELSTRGKQFLACSVVALCAARLYRLIDWSPQLLDSIVINGNRYHQQSLGDIKCEDYNFSI

EDLNLECWLDNLRFVVHIEHVVYGKLYSRPYYNRMNLAEGLMYFFTYFQFGILQCLNKCL

AIGYIPGHDGGYFMFDCQSRDHPIFPKGQGASYVLRTKHLQVLLYCIVVTLNVPYYNVLF

TIHKVEMLADGASVEEDKTKEETAEQTEEI

>GMOY009632-PA hypothetical protein|protein_coding|scf7180000651846:4080106-4083389:-1|gene:GMOY009632

DVRAIDPKEDEHPKPGFLCKQFWKNHWQGVFSVVIPVVCLPIMLLIEGAEFRCLYLITIC

CLLWITECIPLYVTSLMPIFAIPMLGLMDSDKTCIQYFKDTLIMFAGGLIIALSVEYCNL

HKRLALKTILVVGCSPRRLFLGVIMITTFISMWISNSATTAMMCPIIKAILEELEANNVL

DIYMDESKEPAVDGKKHPTRAALAFYIGAAYSSTIGGCGTLIGTGTNMVFKGIYEGRFPN

AKDKIDFPRFMMWSIPMVIVQAGLMFFFFNITHLGLFRPKSAVGQQVNAGAKVAPAVRAI

VADKYVELGPMSAHEWQVAFWFTVMVAALFCRSPGVFTGWADLLNRVKIKTSPCVMFPVL

ALFTMPAFWTCFKHFKKKGPYPSEPIKSLLSWEFTHKNVPWGLVFLLGGGFALSTAAQDS

GMSKMMGQALAFLTTWPRVGIQFMACVVAVFMTNFSANVPICNILIPVLNEVALVVKVHP

IFLLFPAGIACSYAFHLPVGTPPNAIVSGYANIRIKYMAEAGILPTIFCIITVVLNSNTI

HRLVYPEIDLPEDMI

>GMOY009635-PA hypothetical protein|protein_coding|scf7180000651846:4114881-4118730:1|gene:GMOY009635

MESAQQCGCNDCLRTVPASEMPRPLDGECHGFNLWFASFLTAKKEKNENYNSLGSPIGLV

SSNVNFKNDGKGPDIAEVRYATYLKAFARLHVNPETVWNSLVIDEVMKDGLLLCSASERE

KNPPISSPDIYASHERHILREFELVGCNFQVELMGKFRLVKSSTDKPGEAGDGNNPDLPL

VENIKSALKAFFRKHKYCLLSVGQFYVMIWHTRGVFFIFDVGGRRLSDFNSDKEKGVAML

ICLKTLDNVRHLILNLSDLSKNDPCTMREMKIVKLVTPSGNVIQREYGPRQHEYEIASDD

YAYLKGKLHLSLNRGELLRNRSALPAGVTAMLVSKIDHPATWNRKMVDKIICFGVNFCQA

CWIKCTSTDPIDVDEFPSYFNIGQFRAHIELIPRKYEGIWRCVPGYKFTDLAQTLERAFE

MGDTKLLFQINFQMYAIWKQNDFIYLFDPFRHRIVGMPNQPSKVEDMEKHATVRMFRSFD

VFMTVFNAILLDSNRSSPFYLHVLKIRNIQLKEKKDGTPAPEEEPQLGPDGEVKSLNEVI

CFEESEDMCQKMLGEISDYEDEDLFSAVFELELKTSSSEVEIMEEEEEGGGGPLEELEGL

ESSSTDDEEGAKKGGKYGKGGKGGKGGKGGKGGKGGKGGKGGKGGKSGKGGKGKKGKGAQ

DVDGDIGDEDDKKGTGGKGKKGRKGSARAKSISADESKVPQRDLDKKKEGERTAELDKQK

QTELDKLKQGEMDKQKQTESDKLKLAELDSQKQQTVGDKLRQAEQDYLKQLELDKLKKLE

QDKLEQSEQDKLKQAELDKSKQAKLDKQKKSPELDKQRKAEFVDLKEAELDNKQRLADLD

KMKQSELDRQRQAELDKQKQAELDKQRFAEGSKLRKEEMEKLKQAELDKQKQAEIAKQAA

KSKEDKKPELDKTKRTEHEGLDIYKQLKAELEEQKKAEQELQREENFAKQKQAELDKQKI

EELERLLKEGKEKQMEMEKERQKDLEKLLQLEREKERKLKDKAKRCAERRDEATEADDEC

EDFFKEGKRFLAAPNPNRYPGFAPKAADIVVVGSESGSYNSLCKLISAGFRAADRILVMT

PWGNFVLFRCENKENKVKNYFLFDGCTCNVNRFRHLDLNVGTAGLLSFKQVNDLIEYIRR

VRKQRHRRTLTKCAANEICKQYCV

>GMOY009671-PA hypothetical protein|protein_coding|scf7180000651846:5510140-5511551:-1|gene:GMOY009671

MLPNMRTRRITGLHLTGTSACPISKIPPKYSNHDWDYNNKIKFRITCDQERLAERIVEDS

RRVVDDVNDTTRNWQREVEHHLRERASEIRFLCDELNKQKKTALLEDEALTTYRNRILNA

IGFLKEKSLNICQRCLVLREGRLAVDLCEDDVDRNLRRELKVIKGCQCLMDKALKETHEQ

MRKLRAAMYLLDKDLAQKDKSLLIDEKNLKLRDSQHDLGSSDNANQPCLYTLGEWQQKTY

LNIEGNGKELNSASQLRAYIDLILCQVCEDMQNQTDRTNEAFNCRITEVKHIKKCLENKH

NDTMNHIIEVQRNIQLLEGEMSDKNRAHQLCLTRLSNRAARPGLEMTCDEVQQSLYNELD

ALKASICKLQQKIRENKASLRYLMHVQVMQEEEINIKNNSIKIDEVDCMTLRQCLKYQAF

>GMOY009723-PA hypothetical protein|protein_coding|scf7180000651859:16000-35036:-1|gene:GMOY009723

MRHLWVLTFSLMALSYVKPFTAIASKMYTCSSEFPEALKFFERENELMRLRKRKENLAAF

KYSTDLTEENRQAMIEVASQNAKENKELAESIKKIFKYDQITDPCLKRQAVILSDIGADV

LDPEDFVTLQNAISKMHTNYASAKVCSFENPNLCSMSLEPHIQGKLGQSRDPQELEHYWT

EWHNKAGTPMRKQFTKYVELTRMAAKLNGYDSYADYWKHYYEDPDFENNVRAVYKAILPF

YQQLHGYVRHRLFQHYGPDVIAVKGNIPIHLLGNMWGQQWDNIMDLLTPYPNAPTIDVTG

EMKRQGYTVKKMFQLGDEFFQSMGLRALPSSFWELSMLEKPDKRSAVCHASAWDFYGDSD

VRIKMCTEVNAHYLYVVHHELGHIQYYLQYEHMPTPFRGAANPGFHEAVGDVIALSVGTP

KHLHAIGLSNVDRSNEQSRINELFRLALKKVVFLPFAYAMDKFRYAVFRDELREEYWNVN

FWQIRSALCGLEPPIERTERDFDPPAKYHISADVEYLRYFAAHIFQFQFHKAMCLKAGEY

EAGNPEKPLDNCDIYKSKEAGNAFSDFLSSGNSKHWKEILEDFTGDTEMNPSALLEYFDP

LNKWLIAENRKMSIPVGWDVTDTIVNTKMNMKLLLLAFLASVALCSAGVKEEIVAAEYLQ

NLNAEIARRTNIETEASWDFASNITDENEHKKNEISAEVAKFMKEVAKDIAKFDWNHFQS

DDLKRQFKMFSKLGYAALPDDDYAELLEVLSSMESNFAKVRVCDYKDNNKCDMSLDPDVE

EIITKSRDPEELKHYWLEFYNKAGTPTRNRFERYIELNTKAAQLNNFTSGAELWLDEYED

ETFEQQLEDIFEDIKPLYHQLHGYVRYRLNQHYGNDVVSKTGPIPMHLLGNMWAQQWSDI

ADIISPFPEKPLIDVTSEMVKQGYTPLKMFEMGDDFFTSMNLTKLPKEFWEKSILEKPTD

GRDLICHASAWDFYLVDDVRIKQCTRVTQDQFFTVHHELGHIQYFLQYQHQPFVYRSGAN

PGFHEAVGDVLSLSVSTPKHLELVGLLKDYVHDEEARINQLFLTALDKIVFLPFAFTMDK

YRWALFRGEVDKSEWNCAFWNLREQYSGIEPPAVRTEKDFDAPAKYHVSADVEYLRYLVS

FIIQFQFYKSACIKAGQYDPNNPQMPLDNCDIYGSVEAGEAFHKMLSLGSSKPWPDALEA

FNGERIMTGKAIAEYFEPLRVWLEAENIKNNVNIGWDKSDIGWFLHRKDILDWNVSLSGR

FSTCAGCFKLLPHYKVTCGSGSLRTLKYLRWSSLSAKERAMSGLKVSLITILSSKICSAF

DATSSAWCEMKILFSYLRDIFILFITLSLSTSLPHSEVALQKFLDEVNNRLAIVYNRNVL

ANWQIEIKGPNDLVALLQSEVSAQDIMRYIRSISSKVKYFRNQNITSENSRRQLSLLPQV

SYEILPREDLEFLHAITSNLTLVYKNAKLCSFQNREQCNLTLVPDVQNILENSKNVQEIE

YYWREKHRKPALTNKEDFQTLVDLYRKTAKLNGFSKPSDFYRILEEESTTLLDLFEKLML

ELRPLYEQFHAYIRGQMRQNYGSSLILFNKPYPQHLAEVFISNAYKPSGSKRYMSLPYRD

PAYNINITEGLWKAKITTAFENFHSAKTLFKSMGFLELQDSAQTKADLDEASCWHKVWKF

YNLRRVNVTYCPHVNEQTFFNMFEALTDVYYYKAYENLTTLYQEEAWQNFDETLGKFFSL

VATSPKFLGSLQVVGSSYLNKETRINRLFTQGLRSILFLPLLYVHDRYRIDIIDKSHDIN

ANCFYWQLIENYTGVAAPIQINKQDFDAPAKLLIDFENQYGSQIISIVLQYQLFEYFCSL

TKEYIKEDDEKPLDLCDLSQKAIVGEKQVMSLGSSKSPKEIMKILFGKNEISMNGLLAYY

KPLNDWFFEQNRLNNYEIGWQRSESCLA

>GMOY009744-PA hypothetical protein|protein_coding|scf7180000651870:149212-160976:1|gene:GMOY009744

MQLYLVGTLALIIFSTRTQAAEKNADYNFDEYEEYENYDDDEGVDNVQFFPVMIAVMQMQ

MVSPQTISGGSCSKSVETVADFDVNKYTGRWYEHEKYPAIFEFGGKCIYAEYHDLGNNEV

SVFNFQKNKLSGLGSSITGTAKVVSSGKLEVRFDGFAALAGPGNYWVLGTDYENFSVVYS

CSDFVGVVNTKVIWILTRQRFPEEKIVAQARDVIASHGFSLTNLVKTDHSHCD

>GMOY009777-PA hypothetical protein|protein_coding|scf7180000651886:372-8096:1|gene:GMOY009777

MEQILFYSIIVGRFLILFIVMETVTGGFVNIKTNCKTIQKLQRAWSLCPTTFRGCLEKLK

HIMHKYLILIVVMAFMIGQSLANIDCAVVGELEGAWNLCAGSFKDCLKRLKDEYGKAYSI

QSKICKWCAAVDICKGMKENDGDSALENAPDVLLGVA

>GMOY009778-PA hypothetical protein|protein_coding|scf7180000651886:12616-13850:1|gene:GMOY009778

MVKILTCYVMCRVIPSSLVVSSATIIGKFLTLTILMVSMVPAMCRYSCNTLLRREALWGL

CRPFSNKYLRSEVKSDYVTAIGLAYLCKRDLSSCVKTEFTNGFPDNLIYAWCNGVDYCKR

FR

>GMOY009830-PA hypothetical protein|protein_coding|scf7180000651900:184959-188579:-1|gene:GMOY009830

MFMSIDFAINARHQHVEHNNQARQCTDTFNKPEESQRVLDFLRAHYYPEEPLNVSIEPKR

QEDADEEYTMSMVKYGMSLMAVEPISQNSKDRIVGALIVGPKDANEANNLFKAANRAATR

KWYHMTQLLACVERDANVYERYKVQRVLHNHAIAVDSTMRGKNIGSRLITEMIKVAKDAR

FEAITADCTSFYSAKLYERLGFECINTIYYSHYKDTNKQQVFRPEPPHTCIKTYGYRL

>GMOY009834-PA hypothetical protein|protein_coding|scf7180000651905:94272-120383:-1|gene:GMOY009834

MRPSFSALLFVYIVLNVTLTSRCQAATVPHFHDKEDESFVANPFYHSNPELEDLFARFLK

NYPNHAQVYTIGHSTEGRPLLVLKIFQHPRYTNLLTPSVKLVANMHGDEVVGRQMLIYFA

QYLLMNYQTNLEISQLINSTDIYIMPSMNPDGFQIAQEGNCESLPDYVGRNNAAGEDLNR

DFPDRLDNEHHAQLRTEKRQPETAAVIDFILSKPFILSANFHGGAVVAGYPYDNSIAHHE

CCEESVAPDDIVFKHLAHAYADNHPSMRLGNSCNETFPGGITNGAKWYELDGGMQDFNYV

FANCFELTIELTCCKYPLAVTLPDEWASNKRSLMNFVKQAHIGIKGLVKDVNGYPIHDAR

IFVSGFEGKTVRTTQRGEFWRLLVPGIYDVQAMAFGFQNSEVQKVEVTNDNAVAQRVDFT

LSPISNNIDGNFRKVIVERADKAEDVEKNGGFLTLTEFVHHNYAAMKKFLHDIADNYPSI

TRLYSIGKSVQNRDLLVMELFAKPGEHLPNVPEFKYVANMHGNEVVGKEMLLLLTKYICE

NYMYDDRITKLVNNTRMHFLYSMNPDGYEIAREDDNTNAIGRENANNVDLNRNFPDQYGT

DRNNRVTEPEVKAVMNWTLSIPFVLSANLHGGSLVANYPFDDNANDFNDPFARLRDVKVS

RKLNPTDDHELFKHLAKVYSKAHPTMHFGSPCPKFKQEIFPDGIVNGAQWYSVTGGMQDW

NYVRAGVMEITLELGCVKYPKASELLKYWKDNREPLLLFIEQVHNGLHGFVRSSIGNPVP

QAAITMDGSRHTVYSDTYGDYWRLALPGRHNLTILADGFSPERETIEISAEKRSLRLDIT

LMRDDDQHWASANDYRIIENVVHTRYHTNPEIRERLAEFETHNDNGQIATFGYAENEFGL

YYNSIKLTSDIGAPEENKFKILILSSFFDTTSPLGREISLNLVRHIIEGYKLKEPRILKF

LQNASEKRRDLLLQFLDNERFDLMLTFTAGHSELSYPKDEQIYERFAHYIDDVEFSFSPL

QCPVSSTRTVHRDITERLTNLLYKTYNIPMFSLGAKGYVQDEKGSPLREAFIRLIDHKPV

YNVTKNLARFQLMLPIGLYALEISAPKYQSYVAKVEVIQGKISDLGVIKLSAYTLITGHS

EIVPLGRDRHPATVLTGFVLDLSNHPVPHAKVSVIAPITKHYLRNFTDSIGAYTINNIPN

GDITLKVEAPRYLEATRLVHVTDDGMPIKGVVFRLKRNEHVMGMPRFIFIVFASIAIIIV

VVMCILCAQFFLARRHRTDKPYYNFSLLPQKGKELFEDDDLGDDGETELFRSPIKKGMII

KPYFDDDDDDLKHIMHSDNDGDDSDDYSDVTDKHAIMDNQIKAKHVYNDDSGEEVIMLHK

QQNH

>GMOY009852-PA hypothetical protein|protein_coding|scf7180000651917:18271-20036:-1|gene:GMOY009852

MRKLIPNIPFQMLLRGANAVGYTNYPDNVVYKFCELAVQTGMDIFRVFDSLNYLPNLILG

MEAAGKAGGVVEAAISYTGDVSDPNRTKYDLKYYTNLSDELVKAGTHVLAIKDMAGLLKP

QAAKLLISAIRDKHPDVPIHIHTHDTSGAGVASMLACAEAGADVVDVAVDSMSGMTSQPS

MGAIVASLQGTPSDTAFDLGSISEYSAFWEQTRTLYAPFECTTTMKSGNADVYMNEIPGG

QYTNLQFQAFSLGLGDFFEDVKKSYREANLLLGDIIKVTPSSKVVGDLAQFMVQNKLTAD

QVLEKAEELSFPKSVVEFLQGSIGTPHGGFPEPFRSRVLKDMPRVEGRPGEKLPPLNFDK

LKKDLKETHPNVNDRDVMSAALYPAVTEEYLHFREAYGPVEKLDTRIFLIGPKVGEEFEV

NLQKGKTLSLKTLAMAEDLTPNGEREVFFEMNGQLRSVLIRDKEASKELHIHPKASKSNK

NEVGAPMPGTVIDVRVKEGDKVEKGQPLLVLSAMKMEMVVQSPKAGVVKKLEVSNGMKLE

GDDLLMLVE

>GMOY009975-PA hypothetical protein|protein_coding|scf7180000652007:44856-54005:1|gene:GMOY009975

MKGFGTDEKAIIEILARRGIVQRLEIAEAFKTSYGKDLISDLKSELGGRFEDVIVALMTP

LPQFYAQELHDAISGMGTDEEAIIEILCTLSNFGIKTICQFYEQSFGKPLESDLKGDTSG

HFKRLCVSLVQGNRDENQGVDEAAAIADAQSLHDAGEGQWGTDESVFNSILVTRSYQQLR

QIFLEYENIAGHDIEKAIKKEFSGAVEKGFLAIVKCCKSKVDYFAERLYDSMHGLGTKDK

TLIRIIVSRSEIDLGDIKEAFQNKYGKSLESWIKVSLDFIYFAIYVAIAFGYGFLQLIYK

MVLDFFLILEDLSGDYCKVLVALASY

>GMOY009976-PA mitochondrial F1F0-ATP synthase subunit OSCP/ATP5|protein_coding|scf7180000652007:68083-72872:1|gene:GMOY009976

MAKALRETADKADMTPTSGNLLSLLADNGRLNLLDGIANAFRMIMAAHRGEVVCELIVMR

IIE

>GMOY009985-PA hypothetical protein|protein_coding|scf7180000652007:172458-174909:1|gene:GMOY009985

MTGRGKGGKALGKGVAKRHRKVLRDNIQGIKKPAIRRLARRGGVKRISGLIYEETRGVLK

VFLENVIRDAVTYTEHAKRKTVTAMDVVYALKRQGRTLYGFGVRMPPKTSGKAAKKAGKA

QKNITKNDKQKKRKRKESYAIYIYKVLKQVHPDTGISSKAMSIMNSFVNDIFERIAAEAS

RLAHYNKRSTITSREIQTAVRLLLPGELAKHAVSEGTKAVTKYTSSK

>GMOY010053-PA Serpin 12|protein_coding|scf7180000652037:24026-25697:1|gene:GMOY010053

MFIKQSQAQNLDRNLRDFAMKTYQEMTEGSRKFLFSPFLIHMTGAVLHLGAKGSKSGVEI

DKCFHFDPNKNSEIEIADYFYRRLSYLQTSKFVRFANRIYIDEGNEMKDKFNTLLNEKFL

LDVESVTFSKPRKVLARINEYTASKTGNLMHPTVYEDEFFAFEKLLAVNVLYLRGKWKTE

FPPKYTSIKDFYLKKNIVVPIHMMHLKAELNYASLPQIDATVLELPYEEGDEKWSMSMLV

FLPDKHTTLENVESKFYKLYFGDVLEAMFSTLVSVELPRFSHEERGDFTYHLEEMGLNRT

LQRTNDFDKMVKSPTELELAAMGIQFFIDVHEEGANQAPEIIGDGKQFIANRPFMYAVIC

KSHILDEPLPLLMGRYTAFYDVILK

>GMOY010090-PA AICAR transformylase|protein_coding|scf7180000652062:35649-37552:-1|gene:GMOY010090

MASKRIALLSVSDKTGLIDLGKSLAALGFQLVASGGTATALRNAGLNVKDVADITGAPEM

LGGRVKTLHPAVHGGILARTTPNDVADMKKQNFDYISVVVCNLYPFVNTVSKPDVTVAAA

VENIDIGGVTLLRAAAKNHDRVTVVCEASDYIKVLTEIKQHGDTTLETRKILALKAFTHT

ASYDDAISDYFRKQYSGGISQINLRYGMNPHQKPAQIFTQLEKLPLTVVNAAPGFINLCD

ALNGWQLVKELKKALGLPAATSFKHVSPAGAAVATPLSKEQAKLCMVDDLYSSLTPIATA

YARARGADRMSSFGDFVALSDVCDVVTAKIISREVSDGIIAPGYSPDALEILKKKKNGGY

CILQMDPNYEPSALERKTIFGLTLEQKHNDAVIDAALFSNVVTKNKNYLNRRNSVCYARD

GQVIGIGAGQQSRIHCTRLAGEKADNWWLRQHPRVAGMKFKAGVKRAEISNGIDNFVNGT

VGKDMPFSQFEAMFEEVPAQFTMAEKAAWLKQLNGVALGSDAFFPFRDNIDRARLSGVSF

IGSPAGSTNDAGVIAACDEHGIIMAHTNLRLFHH

>GMOY010097-PA hypothetical protein|protein_coding|scf7180000652063:50963-54131:-1|gene:GMOY010097

MSSVIKRLLEKAPATKRYLSKNQPTLKADGLLKRLLDKVGFPKRELSKKTTAKKPQGVQE

MAHPLEHATGLEKKEMLAFLSGKCDPYHSDVIKRGSGTAENPTLIPSAFNGRIVGCICCD

NRFVNYMWLEKGCPKRCECGYCLTCTNMTTNDTNDGQPKIQLGDYIILQRQKYTKLVKFS

SISATAALGKEQLELGNILDKPYFSTFKMCPKEDLGKSHRGRQRLHTLEICTDVELPNIR

EAVSREALGISSSGVDNRNITDDGDSQSLKSDDIEQLRDEYNKRKVFEEKEKKYFEFVQI

KRPNIRLIAEIYYRQDAEKIMSIRMDTLSQIMSYSGVSAYGNYLMYESGTNGLLPAAFLN

AIGSSTEACLVHMHPGNVPQQQALLALNFPEEQGRRCISVNIYSVLRDYYQGEDSKEDEN

TDCNEHLEPCIKRPKLQSEIEKEENQKEENAGQQSISINNIVEEIVATRTENDSDECKQT

QETTAVNNDCNMKPLNKVHAQQKWQLENRRACLLMREKFDSLFIAAKEHPANLVRELLQF

IKPSRPVVIFSLCREVLTELYVDLKTNNSKVINLHLTSNWMRMYQILPNRTHPEVNMQTN

SGFLLTGYTQMAKHHPDLIFCRKQPGVAIGRLCEKCDGKCVICDSYVRPCTLVRICDECN

YGSYQGRCVICGGPGVSDAYYCKECTTQEKDVKHQRWLSENCESWQLQNRLVLRTQKIWV

QTKLLTLFRSSFLRPINTFHLIRKHLL

>GMOY010107-PA hypothetical protein|protein_coding|scf7180000652064:120228-121390:-1|gene:GMOY010107

MASLIRKLISTGNAARVIAPYNQAVVADRTVYVSGCLGMDKTTMQLVPGGAAAQTEMALK

NLEAILIAAGSGIDKVVKNSVFLKDLNDFGEVNEAYKKVFCKDFPARSCFQVAKLPMDAL

VEIECIALTGDVKTVSAQ

>GMOY010111-PA hypothetical protein|protein_coding|scf7180000652065:329568-342951:1|gene:GMOY010111

MKSFVAKLGLLVFCACIINGEKDNPATEAEIKIYKRLIPADVLRDFPGMCFASTRCATVE

PGKTWELTPFCGRSTCVQNEKDPSKLLELVEDCGPLPLANDACKLDTNKTNKTAPFPYCC

PIFTCEPGVKLEYPEIPKEEQSKKE

>GMOY010173-PA hypothetical protein|protein_coding|scf7180000652081:10527-191284:1|gene:GMOY010173

MSGQQPPANQPPYMGGPRGWNPRPNSPWSAPAPSPPPLASGARRPSLPSPVAMGSPTLFG

PPISPGGSNLRGTRGSTNMPHPRPQWPQPLSPLAAKQLQSPYPGPAFQQQSPQMCPSSPV

QQASGTGVNGPKPFRPLHVQQQRSYGDGGPTSSFQLSPTPSPIVCHTPVSDFMYGSNRLT

THNYETLPQQHYTRQQSLSSMSASPSTYHRPPQYEFVGVPLEPPQPKSYVIYDDDDDYGP

TTAEIIANQSQDYIDEKLAEYQMTILQLQGDRSVPKPAVRKRRLRQTAVEPNDTSPDVSP

TMDIDETNAADETASTVTRTVIKTIKKTTKEVVRSTSSSTEYLLDSGAPAWNRTPTSTSP

HRLRDKVAMYEKACSSGGGGRKRSTEHISDERTSRSHTPQRMFDFTDLETQRKDEIPFEM

DVYDIEKRLREERQRGLAEAEGAKLAFQQVQLKATPQPKPRKVEIHEEHTPSPFNVTLKT

TSKISPGAVSGRIELDEQSNSPFNVTLRTTQRYRRAPPHLEEISSSPFNVTLRTTNRHSS

LSPTSPNRIDPKLSTARFLEGEKTVREIQAADGVKTIVTSSMTTDGRSHVEKIFRHGEGY

LSPRGSPHREFHRGVTPQRVIDVTAANPRILIKVDETDEQTKDHKESIDMTDYIQPADIG

SVAPETSTGNIDITVGGSKQSYNNNNLMFVETKHPVMPWQTETYTTTTTEKTTTQIKTLN

RTQNENSIPEDNVSYTKTATSQTTTKSPEKQKFEANEPDVLAKEDIIVKKHYKRVGYDCC

ALHKDTTTGGGGPVKTKKLSSTTSEITDDSDTEGTAPSSIVIVPVQASALNASVSASVSA

SASASASPTKITGLKLVSELVTSPSSGTLSKGLSPSQTQQAQSINNDYQEFQSTMAAIQF

NRSNSQYDSHIKEKREEQERVQKKTFTNWINSYLSKRIPPLRVDDLINDLRDGTKLIALL

EVLSGERLPVEKGRVLRRPHFLSNANTALQFLASKRIKLVNINPADLVDGRPPVVLGLIW

TIILYFQIEENSRNLEYLSQGISGSVTSLDSVDKASGDARAEKWKQGARKTLLNWVTNAL

PKDSGVEVRDFGVSWRDGVAFLALIDGIKANLVNLVELRKTNNRHRLETAFDVAETKLGI

AKLLDAEDVDVPKPDEKSIMTYVAQFLHKYPEPKGASKDFSHINQELDELRRFLIEKSDE

YEPMVKLNSFPRDFSEYLIARSEIDAHIPTYNHLKQFVDTQSGFLQVPRSTWEDINSLWQ

QVQYQIMYWLWLLDSELPGEFGIVGKWLAEAEKILMDNEIPSAMNEETAAIISRKLEEHK

LFFADLPRIIAMFDNAKRTPLAQQIPLEQLRNMERRLQELGPKAAERRIRLKFLEHKCCL

VAFLNLVENKMKGWTGKYGTQERVSQQLEQYKNFVSRNKIFQEFQKAFVDMQQVVEEYKR

EGNILRKEVVDIDRFMYDIEERWKRVSMELKCCQNSLEEVVNCWKTWHQLAPACEQWLHI

AEQNIKRSEDERLEFFKDIGVWKDKFDALANAANYLMASCEEPISKNLRQQYENLSERFE

RLFVNTKKYMHAGDIIRARQEYKTSIEKLSQWLRYAETILSRKLILGNGEQIEKYGEELQ

QLASQIDDNEELFKNISRNIQGMIQDLSRDEVDGMMKCLKQEKESLVRIRAQIPAKLHLI

HQLLIQQESLESGKKEIHQWLNEAEAVLSNHTVIGGREKINEELNKHKTFFSRIVYYRSM

LESKNKVFQNLLKNIVSDESIDVEQASQEVQQLNERFNYVIESAQQWEQRLSDAIRNWNR

FNEDEHVVSEWLTQAETMLNEKHFESKTSIETQKYFFENVNERWMQNLIESAHDLLKMLP

AEEQKLVVEPIEVLQGRWQTVLAQAPLHLIKLEFRLDETAFYSTLQDIEKELHLEQQALN

RNEDVDSILQRNQQFFLQQNYTPRIEKFLQNMQRLNQVYKQQKPTDVSLEEAYNEARSQW

SHISHKISEMRETLQQIPAQWDSYREKFTEMVEWMNIVDKSLKNIVNEVNSMEEFEKEKV

VFQKICQEADSKREDMKWLVKTLDHLLGYAGEDEANVEQKKLEDLIARYKNLIPTIEITM

VKTEVFSKCYTYRREVHEVVNLLSKVREQAVNIPAPDSLERVNKLIEEQQYAINQLDHQR

PHIMSMLQRGRDLSKDVHAPSFVQVEVKNLETGWNQAYTETSEKLHALKGTQTIWNEFAD

QKSEIVSMLHVAETELRALTPLQTDPKNVSQDLKAKRELNAQLQQASHQLLPKLHTLKAE

LSPLAATEKRPILEKEVTEVEKMFFNTMEHVKDRVSYLEDYSGKWNNYKARLAELQEWAN

KIAPKSIEALHSEDLTPEERMVKVNVFKGVLAERMKQLDVLAADASELAPKEGNVAEAKR

LKGEISKLQEVLSAISRNVDHQAQAAQEDLLSWQQFQTGLQQLKPVVEECEIKVNTIVPK

PLSLEEAVALQQNAEHFETLCLEQIDRLEGISNISHKMLCKSNAPDEVDAMHSRWTAVYE

NAKQSSGRLEKLVSNWKSFEAEIHKLEEWVEKGEKMAAKKLIALSTPHIDKLENELVRLK

SFNNEISEQQAKLVALGQTADQISLHLAAEGAGVLKDNINVLKTKLQKLSEATRTNINEL

SDAIIARQDFNAKMVNFSNWMDQLRNQVAQVEDVNPERVEISLHVIHALMQEHADKKPSF

NAIYDEVKQLSLSASPDEAMALNEAYSALVLNYQNIENNMQQKKVCLEKWSELLNWKHET

ESHLNYLKHQIDKHDAPGSQELNKLIMEIDTIGQSMPYWKSQAKEIEENPVVQLKDALSR

RPLIATQIVNDIENKLENLQLRSQNQKHQLEQIQMRKNKFHSLESDLSQALQSDRLKLTE

ILKRKPSLASIDQIINDLLALNDALKLQADLKNRLHDEGAQLMREDITSMPAIQESLLHL

DKDYESLQSEIDDRIQKYQLINQALREYADIKNKFSQELQKAADLYNAIPGQPRDEVKLH

QAAEKTRKTTEQLRKVKTILDEFERKGNYVAKLFNAIGEAVPQEVPDDLTAAKQQCHDLH

DKTVKNIHMYETEAVFWSQIEDAKKDLIPWLSETNQGLCDAADNSIEIEFAPVRLTKYHT

ELPSYQALRDTVIEKSQELIRINDNNEIPALTALNQLLNDQFREVENNANRLNAITRMFN

DHEQELRQKIKYVGENVNKFREQLIKCDDLSGDNAKVVERLQKCRVLNRELSKTGNEIDN

IKLKVDELRALYPTFSDSIVPKELNNVQKRYEGVEMSAKKIENTLLQFLKKFHIDKVGML

KRLIVTQREKVAWCQPETSSDKYNLDVKKSSLHEVSKALDDCRKRQAEVQNSFEMLKAID

TPQNIAEMTKDVDLLNAEMKDLENSFNKIKNILEENVDLWSQYERANEEISSWLRDIEAR

IKVETSSQVNLQNIPQKLQELTRLNQDIKNHEPIITDLEKTSHQLVEKNPEARIGQFVNH

LVQRYQTITKSLAAYVDKVLATERAYNDYNKALEEATEWLSESKIEFQELARMGSPGSSS

ATAQQLQTIKNYLNTFDNGQKLINNAVDIGESIYPNVMPENREKIRTELRGLREKFDYLR

DEANALLQQVEAVLIQKTTIEESYTQVSHYLNESKAKAKATDELYPTLAAKKSALQNYKI

QLQEATLHKNALKQLQEKAVALYDDESERKTEESIHEYNDLSKHIAERISVVSDQVVKHE

AYDQVLEKAQDWLNTIKSEAFDILNETTFEKEGAEEKLIIVENLLQHKPEGDSIFDACHK

LLQTVLSQTHPSGHPALLKSFQEPKNAWDEFVILCQDSLVKLKQLCSRWDEFEAVISEID

SWMKSVESNVKNQSLKNTASAKRLHLQQLQSIAGDIEKHAKSINDLMDRCREIEGETDLN

LKLSRLNTRYQTLKNLCKESIAKYQIYAKDHESFDQDYETFKKDLEMSVEELAQCKEVIG

DQNILQERQNKLRDLLDKRINDGTVFENLIDRGEKLYGHTSPEGREIIRQQLRSLRSIWD

GYTEDLNAAAQTIDQCLQYFNEFAIAQEQLTKWLKDVDKAMQSHTEPKTTLQEKRAQLQN

HKMLHQEITTHNVLVDNVCDKAQVLIDKLNDNSLNIYLQSIKQLYNGIVQKSAQILENLQ

DCVDQHAELNNKITSAKSWISGEKEKLLECDDAYGEKTDIKRKLEILNNLAQNKSQALTL

ISSIRDQYDKIEPATSQKGNDVLKKEISELEIIMKSHFDDIEGIESKQKEVLQQWNNFEN

KLEDLTKWCRTAEAIFREQQLQSTLHEKVEELEKYKIQRDLILQKEKEVDAFADAAHILL

SNCGADRLKNLTAQFTNRYQLLQVLSKEVVNRWTSLVDDHQMYQDKHNEVDLWLQPIEKQ

LQQTTQEDTTQISNILQVLLAEREHADSMFAALNAAGEKALPETSTQGREKLRKEMRDIR

DRWDKLDEGIRNLQKRQEAQSLQLSNYHEILGQVLNWLDQAEKAVENENPASWTNAQDIR

SKMYKFKAISQDINSHKRIVEAVNEKAAILCGSASSANATEIQKSVDDINKRYSKVATAC

NNLLRQLEEAFEVYQQFSELQKSQQDYQKNLWDRLTGYSDYSGNKPALQARLNKVIEIQN

SMPESMHKLQTLSDHIDQNAKLLPARSKETMARDLSNLYADYEKFSAALSDVKSGLENRL

QQWGDYEVNLDQLIAWLGETENALKNYNLKSTLEEKEDQLNRFQTLVHNLRQREMEFEKL

KDESSELIQSSGETRIAVNVQQVTSRFQSIQATIKEIMKKCEQSVHDHAQFNEKYKQCSD

WIANAQAKFDDTLDLSLVAAREDLLKKQLAVQELLAQQSNATLLLNNTIELGEKCYASSA

SEGREAIRLQLEELQKAFDQLFDNLNSHARKIQDKMSKWSGFDEIAEKLKSWLIELERAL

PAEIELKTTLDEKRGKLLAYRDALNDINNHKAEIGNLQEIAGNLPEKTEHVDEATKQIVD

KYEKLKKRAESYVRSYEEFVSAHQQYCKAVMDTQEFIESTHDTIDYWGNLDLEQVSLHTN

LDRLRNLKSTLADEFPRVDQVRVLGEKVIPGTVASGQINIKTQIGNTQQEWEGLITALNS

AVDGIETRLQNWADYEQLRDNCLDWIREADNKLHSIDLKPNLAEKKTQLEELKTLQGEVR

AKELEVDNVSEKAQLLMKGPSGLRTSGPELVTKYQQLFHKVKELNSRWQQYVASHQEFEN

SISECTAWINSIKEKLEYCADTGSMGQKELDKKMTIIHDVILLKDEGSVKVQGVVELAQN

VLANTAPIGHDAVNKQLTNLQDLWSSIALRIVDVKSQLDDSITQWSGFLEQVQNVSKFND

WLESTLKELSEHQTSMTEKRAQLDRVRTTEEKVRLEKIDVDALKSQAKEIVVTGQQSQTA

FQAQKVFERFDELATRSQKLLSQRQDQYRDHRLYKEAYDDLLSWISRAREKFPSLKQGSL

SDKLAIESAVQATENMLNKQAQGELLVEHLVHTGEVVLASTSPQGQEIIRNDIRSLRDSF

EGLFRDINQQKENLEATMQQWRAYKEEYERLIEWLQQIDILVKNHKLNLQPNLAEKEKQV

ADMRDIMSRLEKGKDDIDRFNNSAAGLLKSHLDSYVNNQLRHLNSMYQVQVNLAKDVLKK

VETNRDQHREYKSNMEAAKSWIDHAKDVIRECSEASSARSKDVLEQRLEKVQELLRNREF

GQNLVHTAINNGEKVVRNTRSDGREPINNEMKELQNDWERLVKKLSTAKVQLETNLLQWA

DYSSSYSQLQQWILDKEAKLQQVSEPKVVKSKRNQQGLSSGLSERKANLRQTNNIVQDIV

SFEPMIQSVTSKASDLQQGAPASEISSKYQTLTKQAQDIYEKQKNTIDQYQAFIDSGNDF

ATWLRNAKERLSKCSEPTGDKQALAEKTHQLKILQGEVPQGQKKLEAALTQAEIACRNAE

PDDCEIIEQEVALLQEEFDTYVDNLQKTKVYLEVGIVKWSDYQDQYSEALDWLTKTETLV

QSYNKLQESLAQKKIVLEEFQGHLQTLFDWQKTLDHLNMKAQMLLETCSDTRISNAVMQL

TTKYNALLTLAKEVMRRLEMHYQEHQQHNTLYQECQSWIEQTREKLTQCEIIPGTLSEVQ

IKLNTVKNLRQGFESGQNKLRYLLELKEKVIINTEQTGAAKIHEDTESLKQDYDNLLIRI

NDVRQKLMNRLSHLEEIYKLYKALEEWLEEIRPSLKTSGEFLNDLSEKRASLEKFRSIQR

DFNSHNDIVEKISNSLKSDDGLDNKDFQKGLSEFEELQMRTNEIIESLENHVNNHDKYKQ

ALLEIQDWLRKAKIEIELCSDCHGEQQHVEDRLNKLGEIDESMVEGKSFLDACKELGQAV

IATSGNEGQDAVTQEIKHLITEWEALQTMSRDARYNLELCLTSWNTFQQKINKINALIEE

FNKRFARANETENKTPEDLINATKLLQEVMAEKENVEELNDTCELLMERSACSRVRDQTV

DTQAKYTKLLTNTQGLVARIEKNLSDHTEFLNYKNEMDAWIMKAQEILDGCNGKGDMQSI

TQQIETINALSSRLPEGQHLLGIVQDTYNRASNVLPEDKQEKLREMMSKVRESWDALGLA

VKQKLNDLKQNQNRWAEFLHNKDKLEKWLTEMEETLKLKPNTKGELSEMKTQLERYKVHQ

NDIKLKGSDLEHLCSEAKNLNADMDEVQNLQELWGKVKNDCNALVNRFEDEVNEYNAYHQ

TLQDVEKWLLQISFQLMAHNSLFIANRQQTQEQIKQHEALLDEIQKYQTNIDDLNAKGQC

RIKRYETSAPAVRLTVETQLKNIQDSYSSLLQTSVQIRNRLQESLAKFQEYEDTLESIMR

NLQEYEPIIKTELDTPPSNLEMAQKQLKCAQNMQNKLNFEKSRLAIAVQACEAATASISR

PSSPLETAMQVIPERELMVRSELEDLLDQIPNKRTQNSTTDDNFDDDVELNAVTDYLLDA

VANERLKKLKNVYANRELVQKLCTLKTKVQSHLSGLIASVGELEQQQKQRAELADWISKQ

QVIVSDWMMRPCKLRPEAAKQELMAMDDLLNAVGDKRSQLMLDLTGSLADDENDLIGKID

KLETQLMDAIAKKKAGQNVIDNYRQSAQDMNNWFDSLIKRMDVLDKGSSLNCAQKMSAIT

DVKKEYDEQGPQKLQDLKSKAAQVAEIISNLDGQQVKEQMKSYDRRYLDLGKRLDRKAQL

LDVTNKSVEAIKAETDQLQNWVKETLKSLQTPALLGYEPKSGEARQQTIKSIMKEAESKQ

SLTDALEKRIANMQSELEPAEYAQLGSALRNLNTEQKNLSAALKLEMEKALEATRLRKVL

ENDLEKARNWLKAKISKVRQLPGYHPLTALEVENKLQENKRFDDEAKQFNDTVLSDLQRQ

AINIMKDCPETERASLQKILDEIEADYKILKDECGSRAKTLGDLLQGRKAFEKSMKQMTD

WLNEMETATEGDLRTSNLPVLEEQLAHYHKLLKKAENMDDLLNDINEQGKGVSPSLSNPD

KLKLNDDIKSMKDRFGKVKQTLNNRVSTLGDHIKKYKDAKSKLAEFMEFLNRIQQRLREL

NKPIGAKIEDVQDLLRAYEGILKELKDSKHNMGDMEVDDLPELQSILAQQDDMIKLIEDQ

LAHLRQLLLLREQFIALINEIIAFIMKYTDVLVDIENAPDSLEEKINKYDDVIVKIQECE

GLLASANDKGQKIASEGTAADKNSITEQLQSLKNQLGNLRKAVEQQRQKHQIQLEHHKKM

ATDLMEILDWLHNTEGVIKSRPLLDRDPDSVEHELQKHHKLCSETQEYLDKFNKINGSVK

SEIGMPGALLEMLSEGRSLVSSLPPEMAEREKYMINNRDSRLAYMKLVAEFNAWLHEAED

RLQCGQHGIDYEHLMRDLEEHKLFFGNEALMRSLVHKQIQEAADKIWPSLNNYEQTELSG

ELAQFQTKLTNVLARAKTQQADLEREHERFREYQQSMERVKTTIECTKFPDEPVNNLAGL

HFNIQKLTHAMGNVMEVLNESEELKSSHKDIEALSKSIVTFLGEVHQPSAEAIQAKLDNL

VQQQSKLNDSLHDKDLQVNRDLEEIETIFRSISSLQDKFNALLEEIQAVHAFEPNITKTE

NELDKLNRESKATVDESNRLINKTKETYLSKQNLIPSDIAQEFTALELLSERVQSNMETK

QKEFKRAKTVRTDYLAGVDEIQRWLMQAEVEVQDRSLAPAQMKELLQRINHEISGIYERF

TLVKTNGQLIIDNCRNNEEKILVQNTVEQLGQQLAQVRSWLDEKKQQVGDSLDAWTRFMN

LYQIVMSWAAEKQAFIDQNIVLRTLPEARNKSTDYQAAVKSIKPIVKHLSEMDKELEHIS

QVTTVGDLKEKLEEAEEAKVTVEAILLQRNALLLEACEEWDQCERKIKEIRNWIEKTKQS

LESAQHKKKPLRDQLGYCEKTMADINVQKTKLSLSIEKLEVHFKNGMGGDPRLSENVDEL

LTILDSLADMVKEKCSHLEETLNQIDVYQKQMQTLRQKIIQEEQQLRLVLAPTYLPHDRD

RALAEQQALRSSIDEMQQSYDVIAALSYTLDPHSVVTTSSAVSMMLKPSMLGLTYAEVLS

GNTSPEIHVKPKKLMAQNYEDKSLESTLTQAATTSHGIDGQNVRPKRKDSKEELSELHSA

HRAITTTTYQTLDSVVTLENNPESVITFKKENLGNYEDIPNTAPSSVVTQDFLIKERKEK

HMNVPKEQKRGRSPRRKAHYPHVENVTAAIVEDIPDQIATPSPTSNTTVYLSATSAITDR

TRSQSPIWVPGSTSYAEVLRGIELERLRQQNLQTANEVILVPQAALAPELVHSQMTNKAS

KFYSNAQSEASASIISYPSGSPTPVYEQHVSPTSTSAYIRSYKNDTPMTAEEIQATYLQP

DPQLYQSMYENLADSGQWSYVTQTTTPAVVQSTQENNFYEQMMQVQYTQGTALQNQPTYT

TGYQYQSNAPQYQMITGYYQAIPAQPQTQIYAPSESTPMYYSPETSQTYNQQYQEETNVN

LYEDEDTTEKAQRTNIQAVYEISAGSSEKSLDMTTTVLATSAASTPSLVQQIQDQAESMT

LYEPQEIAVEEPVTDITFGQFDVNEIESIPIENSPIHCEGDVLDTYETTSERDKEEDNNL

YQQEETIYVDESIEQAQIIEPKEGQIAGSTYAEVLFGLKNDKHFQPSSQKVHINVTTSSP

LNHKTKNETISSTTAITTSNKQRFKSPATEYHKTASRRDDQIKSSRSQTGDKRKPIREEK

VALEQQKDSIVYGEISVESHTPILSTGETTDTQPTTKIKQTKAKKSKKPKDPLERNSLED

FLNNEKQFSSASFTKPDKKFEKKEKKELRKRKSSEAIEDNPLKKLEKTDEIEGKIYQEET

TATTTLKKDKKDKKKTPNEENTSPVMKSIKADQNAFVHGLDQTGDTLKAESSSKRSKSKA

KQKIRSKHSNESTEKSLATDEITQQYIVFEELQKVRDGKGNTPSLSDEKTNELLIGNEKS

KKRSLKDKSTENTLVVVDQQHNETKTDLLDFIEAEKQTVKTIVNKKEKLKSKKTTGGDVS

KSVENDVKKVKRKTKQKVNAAPTREVEEEIIIAEKMIPAISSNEFPEIVASRQNFENEWL

SDLDGNDRTEQEIMISEISEGLDTKNDIENVSASLCEEIILVESEKTATVIQEDERTTKR

LSQESPKEIFSAPLTQPPKHSSVFAEVTENVREPIGKVKNIYVSCERKLTDQVSESKIFE

PAKDPLNTVESKQLQNQPFAAPLAEFASAPTHDEEDLGTIVSEEILIIICTKILEKIVTF

GSILNTITRTSTVTRRMVKSFINGSEQILDESVDESAPHEYVTTEKLDSTTYPGGIITKT

VTTTTRRTIKRRTESGKEVTEVFINEEPQAISDISKEITCETRQEHMKSAQRESGDSVKT

NVSTDTQKEATRNEKYFDEETTESIKKDGRVIQESVGLESQVSQKLDQSIDPTEGNSATL

QVLRGGTISRTLTTLTKRLLTRVNERGEEVIEEVIDDEPQIVQEITELPSETTLVHPGDF

DENLTSAQVIRGATITRTLTTTTKHIVKRINEQGEEVVEEVYADEPLVQQQIDQVPMKTN

EILLVSPPLNNADLNTTSRIIQGGTITRTLTTTTKRVVKYIGEDGQQIVEEIGADEPVIS

QEITILPSISQINSEKEAATSAVELGRGGAITKTLTTITKRIIKRINEQGEEIIEEVVGE

NPVTTQEVTAVPADAEVSDKALSESGITKTVTTITKRIMKRSDGKGDIDEETGEQTECIE

EPDKLYYSPLQPYDDVKKEDSHRTEYTKEQTSFENDEQLLKSPPVEKITEPLTVTSATMS

SPTKEVNDGEVVKTTTIKTTKVVKKITQDEKSDNENELKTIEAPIALEVSKEVTEDITPQ

HQLEIPSKDEDISIQAHTAQLPENHANEPIGEGVLKKAIIRTTKIVKKISQNSDSAIVSA

AQAPENLHKVSSADTAIEDRECENNSKDTGVVKTTTVRTTKIVKRKPQISEIAEDTAVGY

IDNSITEQEPSSAITEEPKEVVSMANQTDGTIMKTTTIRTMKIIKKVPQDDIIPEETNKL

IPVPSIAPQQTFTNELETKTKSSVEPSSPIENSNLKGIELTNVEGTLTKSDLETATTTSL

TKPKTVQNQRNVEQEHNMNYRSICQTGPLHKIELITHEEKFKTPVVKIHLEFPEVSLHVT

ETITKSLETPEEIITVDQALGNEGKTEFDFTNSGEIIENLEEAIVERKALENVREIEKEV

LSASDMTPEDQELFEEIQRKLSKKDKKKRPAIPEEFLKVESYKESLSNSPLAFAQTTNNL

IAEMQSVERTIVTPSASITKSGPQNQEEVQVDTRILTNEIVNTKQFEPVKLSDVMFGAIS

ELQTSVKPATNLINLCPSASYFEIPKYYLPQLFIVERNYHILKNLSTSAIKTQIEDTAVP

STIAAATEAQPHVRNSSQETCYNFEIPKYDSKDISRAENKLISLFSSSENKVENISEKRE

EQNTQVVLDKPYSAPLQPFKEIKSSPRSDDDYDNAEISLQADGDIEMELVELEDNLEKVP

GNSYYNFEIPKYDSIALNKAENEMATNLESGQDVSECKSAISLGEKLTSLRNESSESSTT

ISKKGIIIPDKEENEEHLMTKGLCSPSLQPSKLHTFVPEIVQEVDIPLRDTPKFNYIASE

NLNKEKSNAQSEIADLIENRSKQKREFDGTTDHYKFEFPKYNTFALQQAEGEFVKLSYLE

KANDSLNERPSSFEQVVNIPENEKVGVEQSVSEMPHDTLQRLALQEINVTNKQVEVVEIL

KQDDTENYINFEIPKYDMKAFETLENEIFSTLQQIENKATKIVQEEENDQKFNMIPEKLA

IVPKSSTTKSEKVLLDNNHFNFDIPKYNRFVLNKAEKELYLNQSRDVDLIKLPTDMPNWR

HLVEETREKQSLSDMCSVDGGIHSEADSTEIKPSAFIKELGKKISRVQGVDLNYEFMCNF

EVPKYNVRDLIDAEKQAITVEVNVFKQTEEQVVPEKPYSAPLQPYFQREEGILQEHSSKV

DKEIASESIETFVNSPRPSADESQHHEQGLEIIIYDTKGKMELSATSRETSEIFLTAQQI

KEIHERGDPNEANELSTKDEEKPSKSLYAGLPIDETTNAWMEVFDEPMVFSDEDEDEPES

CAVPLEQCVALKEISSLDKEDEENNFALTDQTPYKKTQSITEEISKQSSETDTTYVMFTD

LPVTDAPQPRSWTEASSDTSILSRNHSQMHDEKAVIDTWSSVLEDSVPSIATIALGSKLS

PNAPEFMPSHMRPSHLDQTNTFLANEKYYNEFVPKSKQSKDGDLKQKQLSKSKKGQKSSK

RLHTPKNIKPDEEVNVLDSNEIVEEKDKASKPPESMKTEQAPGRAEDLEKSTGNTYAYVV

QNNILEREQETLSFHDTVAVTESLGASSIQGNANVQKQKPEYVCHDRSEQDKGTSEKKSP

NKEVSTIGSEKSEPETTDKDIEQSLQCTEEPATRFSWASLVQKPGEWIDNTLTKTKSKLA

PMEKRNPPDSKAKKKEKKSKEKKSEKSGSPEKSVSPEKSHSPEKISALTSTTSEEEDHAD

EKGTIVAEQCKKEELLKTSDDNKPTSSWAALVKRPGEWTDEVASRAKSPKELVTPKVTEE

NKEYKQRANKKKLNSKTDQPKQQPKDLSVVDKDKSQSGNLKLQAEPQTLLFADSRDTKQS

LKETGDDSRPKVADEPHVDQDVNDFSWASLVKKPGVWIDDLVSRKKSPVHLPADIKGEKP

EKSLTSIKHSGQRKLRPQRKQEKEANVDEFSLKIDQTFDSSSQPKIIEQTAVDIKEDVQQ

DSKPLTWAKIASQIDYMTDTVKIKPPKEMKIPLIEPNTPSPKIKKKKETPKIKATLEKEQ

PEEQEERLPSISGTFQSKFTADFIQAERNYQLPKKFVNTNKTAPGSKDEDSKFSSWAEIV

DEPIETMTWKDIVDEEYGDEEEIEILLPESGMQITSETPSPGTVTSEISKNERTPCDVLT

DLVNNREQNVVATVTQEMDLHKKTIIESAPEPSGLFDETSLRSLKETEEEKQVETQELVL

KKDLKIAADRSEGFNTLQTDQYCIQNRAVEQSLESSKIHQTASVENIILIHNDEQVFSTR

LEPFPQNIANIGEKQQSDLDKKMITEPEETVPELVRSCSTEEQNKEHVARGVDENIPTIQ

SNRRSKGLIITRQEQVEEKSVETELTDKRSDTELRIESTIGWTNIMNAPIAYTNIGEQPP

ILQGVVRTETAASSSETILPNAETFILDPIVRDIAKEKLEEDRSVPYAGLPIDESSNAWM

NIDESMAFSDDEDIDSKDLQNQELLEVCSHETNTTKIIGSEIVIKPDNKPSLDTLPKQTV

VAEFSEETNKPPSAYAELLVNGSTIKNIPDESMIFSDKGEQRDPQLALNEENVCEKKIIE

EDSIGGEEIIPVFSHQHIITFEEQTKSDYAGLPLDESSNAWISVLDEPINFSDDESEETK

EMPKSSENTEKHVYDYEVEKPYQTQLQPIGTTVKAVDDNEGMSDAASINTSRQVWTPAAV

VNATLSTSNTFEKNQETDIETITEDNYPRTSETDKLSSSQDAPAGGVPILSAFELPQLDV

PWREITKVEAGVEFCPSQKELLSQRSNKTYASYADILKQTTAFIDLEKMIFKRQKPWKNK

KNLNKREKEKQRAKKSEIKECAASTGITCTGQTTTTCTLSWSDIVSKKTEDFMINDQHDV

SAKEKFEKRSDKNLLGARMDKKSEETITSSKNKKSKTRRIKKDMLEETCKLSDITQSQFE

SPSESAPKKEFSEKISLEQSWAVLTDEEQFSEEVPSNTDSQLTFNTYGAINAGAEEQAKD

TSWSDVLRKDNYKTSSDAVAKQTFAFIKAEIEQSANSSNMQRINKKSTKLPNSEKIERSY

DDKSFKQSGSLALTSDGTESEWDQFVKPLEKEEIAEELDRYECVELDGRKNVMEVAPETS

QNYAETKTFERSENESASKKWSDIVLEEDMEASHNGKLSEAEILVTPMQSLDSFEILDPE

NEISFPIEEKGEKKLKTIEMENERTVSEISDLTHRFGDAERKEEETFVKECEDIDLTLSI

CKKSTSLHVPNTKSWSDVLKENIEKPFASSAVTQPVDMKQKTIDFVQQEQALLKASMKND

NTKTPKQLQDVRFHEQEHLSHLRSEPFKSPSTSRETSEESMHSPSAPAIKRDDIEKTPTI

SEKLPAADTKDQRDVKKKQPKRKKPKTQRSKSPIEEKQGLDATNETNRNLTDASTIEPIP

ALSLMSWSAILRNNMPSNQDEKKLQETTDSKQKTLEFIENEVVSVEADAIQNERVENVEL

EKSNEFATNNIGAEKIVEFENLQCSDDISNSNIYLEVTGKIPESTTANLSDKKKKEKKSK

KVRASKLAKDEEVEGGAISLSKKTLDIPDTMSTSQPSVWSLSKTYAEVVKKSGMSDSRGK

ISYVADDAARSPSPEVVEIAAKTSDEEVFVSELKEIPRVLEESDYETINESTTTPEEMKL

PYVSEPKDSFEIEDTLNTQESSISWKDLVDSDIECDTMDQYVPLNESRVGLESTIEKDGG

IQNIPSFTSLTTSCQENKENSVLTQWKFISSRSRSNSPRSRSRSNQSRKQKRLERSKLQT

ELRASPSNEPIKKIVLQNSRTVEIGWSSADTDDLNKDERYREKHFENEKLDAPEVTVSGP

LPISSTNAAQVGISWAAIAAQNVPQPAQKISVLNEAECALYPDKVVGDVGELKSLVPINL

EVERKEKKPKHKSKKKSVEAEPSYKNISEEALKDFKPMVSGVRISGEELPMLNVRVTQLV

ASEKAAPATEIPVAASSITAGVESSPIVVAKDLASVKFSEVSIGGTVTQIPETIRLSIGS

NEEEPETILCEQEKVHGDINKIPEITQLTIEDTEEKPREIIMKEKVHIPQLVASEEVVPG

TEYRDEESVIPAVVESSSNVVTEDFASVISSAVGVGGTVAQIPEIMQLSIDDNQEKRDTT

SSEQEKMHDNVHKIVNITEQTVEDTQEKTQEIVMEEEVRLSQLVVGEDAAPATEYRVEES

VSPAVVESSPIFVAEDFSHVMCSELAVEGYATQIPETIQAAIGGNQEVEPETTLHEQETM

YGDIHKIPEMTQLGPQEKPTEDVVPGMEHRAEESDIPAVVESSRIVVVEDLASAISSEVA

QMPPREIAIKEIVCDSKVRSYETQSNECEEQNIQVLKKAKSEKDFAFVNSTLIQQFIEAE

KNSGVSSVSAAIPRTTEATLGEVEEQIAAPDSKELLLERSIAAEGKISVCEISLKAGQEV

PAQSFEDIPKSFLIPIKNEEFELPEPVATDGTTKLAKFTSGTVPDIEKRKIVQNAEIKGG

TSKDSNESTIIVSVAKSFIPELDDQYNKVNENELNEMKLDELKQNLRQELREYSLDKSKI

YEDAIDELRSKTTIDKTCDNCVIVQEHPWDSSTQDLLAHNLHLESQSNLRPYHSFQERQL

VLLSSRAITVLEDHIASQPSTKTLNQSPKTVETALRDVGSSVYALDEPQPKAMAAETFAN

CAIIEMHPSGDITQDLLAHNLHLENQFDLKPSYSSQEGLLVLLSSCELTESKPRIASQLP

KSEEITLNEIASNDDKLWLTNLQRDDFSKLTFDDRKNLLRSEVSLAVIQTMEAKDEVTMR

VEKNIPDDFKTSKVVSESVKDMLILNLFKDSWSEIESKAVEKEGVYEQQKVNVKECKEDK

FRNLEGKDYQSDDDDDDDDDDNDQPNIFPDTQKYQDDDDRDPGSDSSGCITSSPKPDQLS

GSYQRSCSTQYMSTDLPGGLGHWRDQSTYLALEEHGKVEEALEPGEVEASKTLAEIELTA

IATTPLIESQNLSFPEPTNMSHSIPEFHNNNSSSLNLVSNTTPLASVATITTKLFNKAVQ

ASATVQGTAAVHEAASAMKTSVAASVLPTIEATSEVTKSPATVETITTITTSTTAAEDKA

PLASAPVAIPTQRLPINYTQTTTTTTTVTDELLQPSMIITPQDPALIRRTTTTTTVTTPG

RSGNGLDNDHHILQQRQPMQKELINQEVDELLQSLTTVEGAIAYLPQDSLDGMLQGLKLI

QENLEYQGKEAQRLKSISQTLPTDPPTERLLREIIDRIELLLRRTQQGITMIAGAVHAQK

KRKQELEEYQMHLTQIESWIQAVAEELKSLEITEQGSNDEHMLRGQVERSQNLLRTLKER

QQSLEDLVDKTKPLLTHEDVASLANNLIEQLQYVITILREQITIATKKIYTIETIIVDLK

KAKQEDELRKRIQTESLIMPTQASVESNASTVDSSSMPEEEIEPVNYAAVETQTSASLDK

PWQPLAEVYTVEAQTSFPLIESKPPNIETADIAMQTQKERKPTENITVTQIVQQGEETIK

IEAAPNADICDQPQNVEIEARYHRKPQGDIDRTTELVLKNVPQVFETTFVEPDETTTEVV

VGPDGTKHIILKKVTRTRQQIVQQQHLSSFPTISHVDSSVLPEPINLESVQPAALPRGTL

PERLIVEEVEGAPTMEEYEEYHSPNVLVHGVPLHEGDVAYVDSRNLQHMQIVPTEEVTHG

SIRAVVQQVTRKVIRKTRKIIKRVVVIDGKEHVTEEILEEPEEVEVTEEEIAPHVNVNIV

RTINGRIVSQDEFDRISTEPGVTIQELSTDVMQPDAQQQTPQQVFDIDSSKFITTTITTT

QQTQQPTTTKTATVIETTESRTKAPVEFVDMPARKVVVEQVIDTVDNKTPDDAVVSTSIN

TVTVTKINQQDQQKGEQSMIEDIKGIWPVQQHVQPTDVEKSMIEHVEDIYPVQQHLEPID

IVQPLAEDIKDIWSLQHSLQPTDIEQAMVEDIKDIWSIQEPLQPTDIEQAMVEGVKNICS

VQQHMEPADIVQPMSEDIKDIWSIQQPLQSTDIEQAMTEDIKDLWSIQQPLQPTDIEQTM

FEDITNIWPIQKPPQSTDIEQPMIEDIRDIWPIQHHLQPTDIEFNLPSEPPTEPLTSTRD

ATAIIWPLNLDTGHPVCLESYQYDRKILPESQEKSVIESPHSKKLPEFTDLQEPATLEDT

KGIVSQFLEKESTPLKPLAPVGKASITIVKAFVEPLHTDTTKPTESCVQSEGSETPELVE

EEPAENKMEIAVVEREDMEEIKELEYIEAEKLEKCAEPSPTKILMQALPSDETKFEKIKD

SKQTLLEFLEKEKYTKTPDTVKEYGTHITSIQDEDAMPQSVSFEPPAQPYKGDNDDRYEK

VHQTEISEMKEHSLDSPIISDPVVCAKTQKPREKVSITIVKSCVESLPSEKQNDNIVEIT

DGQESQQQFAQQVAETPKLLRSSKEELVQYTAIEKYAKPTVVDKEKLAIIDQTITSAIEE

PLEEVEIKKSVEEDPVTLRSLNDLKKTTSGPVEIESQEEPTKPFTQSLIETVETAAPSKK

VEKERKLEKGKIQQGPPSPDKIVEIKKSVPQTPERTTVKIGNFVVETSEPEITAIVSEIV

ESSGKESNLILETPTPYEVAEDSTRKMTTLTDTGIAEALAPEDNQSVSEGDQSSVPTHAF

SISEQSLPTGTLQETSIIENTFLIAQPKREIPHDIKATTQLFIAGEAVASPVSIVAPSIE

GNGASVLKMVMSEQPRPNDMNKVTMTIVETSAIVPEIPETQENIKRSRKKKKRKDPIPTS

STEESLKEEYPKDAEIQPEEKSTSEDLKEEKYIDVGETIPVTSPSEIESPHETGYEPEEK

TADDVHDIKEGKKKRRKKKKQIEKVQPDDETLQSVSPQYSDVSKPSSQSIGSEDKLHEEM

IAEISAESVASSITIDKPVKVVEEGVISPEILEMSKPTHKEVIIPVEIVELSFVRDEQQQ

TTPREKLIQMDDLEKIAEPEKSFKEVQTESLPSVPDIELEDRSLQTTPEILPEIKETESQ

TTPVILSDTLIQTEALEVHLPLDYQGDTAEQSTEGVQTDDFVATISPEIKNTCSQTNVIS

TVEKELQTTPKESPRRPDASIKDIVDPLVKEFIADVTIELPSQDVETRESATLTEESPIE

ESLTLAFEPKIEKLYAEHEKPKEQRDQQTSTVEFVFGSNRDTQTSPREEAVASSLEQTTS

LEPMSMEDSSITISEPYNFEVQTTITIPADSDTSETLPTVYEHTEMVEIPKSGKKYKTKS

EFVEQVLPELNVHVEFDEERESEPKVSVHPVTGETFTFAEVPQLVEFQIAKTTIGDEFAD

LPVQIRQPGKTSLTTTQTSKSRSRPTSTVTIEEVTSPIEEVVVPITPGPDEEITPTNEQS

IWMSSAAIHLPKERTGKESSRDLIMSEKLIRSIPDQQQNIIIIQQPNKWHQTQKTIGERV

KQLKESETHQTPLSNIMHLATLSQQIKEAPTEQRIRDVNEKLEELDKAVRDGNGATVQAV

VITVIEEISTWLETIEYRVYLIRQQANEGPSERKLQHYVELNEELTTIDHNVKQLEEQFG

KSQFAEPEIQQCLVTLKTHIDAVGEKTHDNQVQDVKDLEKWNHFVVLVHQIVTLLDDLQE

RYEIIVSQDGSLQGKLKAFDELESQNNGALAEISQLVVDARAFQRDFPSKKIPQDIYNAH

EVCLNLNNNVMAERDRLLQLQALADEYEQTLQEFTNITVLADKLVDSPIVTNSLEQLNNE

VQKHRKFFVNLSHCRAMLESLEENIDSETREKHSTLHKDLYNRATVLLDKASERSSKLVQ

AASRWTVLEKGMKDELQWLQVAQQRVPDLSAVTSADYEQYTILYQSLSQDISQHYVKMTQ

LSNIANKLQDLIQAPNLVEETNEALIVLLKLREEVAVYLHRLLLFKDIWTQYVAQTDKME

IFVRQSEKELKNIQIPEYPLEQPIEHMRQFWEIKAQFEMHNNIRTEAGQSFEKSLQIIPL

ADEMLQRQFHAQLEDRCNAVADSIERIQNQIMSTLASEDVAPEDKLKLVERELQEIYLNM

TNMKGVIKNEEELCLYIERIQVLKTRVGFIGNELGRIGLQEPAFEPEKVGELFALSHKIT

TQIAEELESASVLRQQLQSIQEGTSNLRKRQAKLSVILDECEGAEKLSNEAIEKALQDCQ

GVAEELITVWQEIMRLRQMLHTLPMHLKMSLSPVKLERDISQLQDDHAFLESKCTNIMTI

LRNRLTMWLRYERQLEMVHNSVQETDFMMELLKVHGQIDYERLRKATERLEGLADDLQNR

ESLLDDLQSSAKPLIETCDLNIVEQIESAVQEAVLAWNDTTDNLQSLCTRYQRAVELWHK

YRNASDQVKNFIEQQMDTAKSLKQQPLDSMQHAKVCEDNLIAQNDKLFELRDIVTKIAAD

IGLDASNLMQGELEELGQRLENCKETITTLANVAETQERERKEIERNCSEAKTYLNNVQQ

DLTRPAQTTKESEEQLNVLRSHLQTLARTEEQLKELRERNLDGSITNGNTNNNEDSAIVD

VLELWQKVFQDTFQEYHRLSSRLVRTQNSAEALRLWRQYLQHVQSFLSSAIPEDYGALKE

QQHLCEIHQNLLMSQQNVLSSTQSEGEQEAEPEINEQFKQLTNLHNETLARITQRNGELE

RRMTIWHNYRSELSELLEWLKEREKDRSALQLRYIHLKRLPRLQQRLDSLLDQIPVGEQL

CAKIRNQQDELFKFCDDALATSVRMEQASVTQRINNLKAALQTWSDFLQKIKHLEEQYEQ

KVDQTQADLSQSQRLVLKTEESLPTSSQDIQECLSTLRSQRLQLANLSSQLETLNVLQEE

LKECISPHDMKSIRQTIWILWQQHADVDYELSTLINSIEERLMLLTNFNNRYDRISKWLG

MLVERLEKSSDISAIANPEEAAKQLEKQINTELALRERDREWLLSSARELIDLYSEQTTH

GNAIRCEVQDKSDMLIDRWERVKYLSKQRSNKVNDLRMTLQRLEERIALLRSWMFEVETE

LSKPITFDSYTPPVIETKLKEHEKIQRSIEQKSSNVGEVLNLVEMLLNDADTWRTHLNTA

NLALAAHNLEQRWKHVCSQSTERKKRILNVWNMLQDLIKLTTENKKWLSTQESALNGLER

DLKKLSKDQIGERQEMVEVKLQELSTYEPNLRRLEHIYGKITSSSGVDPENIQKLTLPTK

VMLSKWRQLEPRCHAIIEAINKNLALIKEFKRNQDETMHSLNCIQRDVSKLIESSPQTAA

EGKEALKRLDACERKIKKTEEQLENVNKLAAEAKNQLKKDEYKQILIWMEQVNRLWQQVS

TSINATKSEWLSKAGGSAAAAVAGVIAEATIKATSAKELDSAVQVDTLGRQRKTRKAQMV

RETSITAKDAYILELETAIKECKSSLDELQSTICDKTRRPGPQKISKLLGNSQSSTDLVK

HLSHLLINECQATENEAQIEVVAELTLRFETLLSQWKARQQHDQNASKLSAAYKDICPHE

IGRLTCPLCTQRNWQQIDNDLWRLEQWLQFAEGTQKAQNTPPSNIELLEDVVQDHREFLL

DLESHKSIISSLNVVGEHLATHTLDTEKAKQLRDRLEQDNKRWNTVCMNATKWQGLLQTS

LMGNSEFHAIIDELCVWLQQTEAKIKASEPVDLTEDRAILQMKFDEFKGLRGELERCEPR

VVSLQDAADQLLRTVDGSAGESSHTYARTLSRLTDLRLRLQSLRRLSGIYIVKLGAVLGV

DGDHLGVPLQMLPSDLLDQSVTTLPSSTSIQAAAPNTENANNDGDAAGGDAINTTVLARG

ARFLGRVARASLPIQALMLLLLGVATLVPHGEDYTCMFTNSFARSLEPVLTYPNGPPPT

>GMOY010298-PA hypothetical protein|protein_coding|scf7180000652119:521-11146:1|gene:GMOY010298

MSDDEEYTSEEEEEVVEETKEKPPQTPADDDPEFIKRQDQKRSDLDEQLKEYINEWRKQR

AKEEDELKKLKEKQAKRKVSRAEEEQRMAQRKKEEEERRQREVEEKKQREIEEKRQRLEE

AEKKRQAMLQAMKDKDKKGPNFTIAKKDSGVLGLSSAAMERNKTKEQLEEEKKISLSFRI

KPLAIEGFNEGKLREKAQELWELIVKLETEKYDLEERQKRQDYDLKELKERQKQQLRHKA

LKKGLDPEALTGKYPPKIQVASKYERRVDTRSYDDKKKLFEGGYDTLYKEVLEKHWQEKQ

ERFMQRTKCKLHFHIQIYLEDTVSVTVRQLSLQVLCVVIAIKNQDNGWEELGKEVNEKIW

NEKKEQYDGRQKSKLPKWFGERPGKKAGDPETPEGEEDAKADEDVLEDEDEVEEEVVEEE

EAEEGEEEEEEEEEEEEEEEEEEEEEEEEEEEEEEEE

>GMOY010365-PA hypothetical protein|protein_coding|scf7180000652136:186474-190856:1|gene:GMOY010365

MLGSNRKILSSYKSASCLIRHYAAAVPKAEPKHEKSSQKANQSFMANIFRGSLVSSQVFP

YPDVLTNDEKELVNSFIPPFEKFFLEVNDAAKNDENAKIDDATLNQLWELGAFCIQVPND

YGGLGLNNTQYGRLCQIVGANDLGLGITIGAHQSIGFKGILLYGNPEQKAKYLPAVSTEK

VYAAFALTEPSAGSDAGSIKCKAVKSSDGKHYVLNGSKIWISNGGIADIMTVFAKTEVQD

KKTGEKKEKVTAFIVERSFGGVTNGPPEKKMGIKASNTAEVYFEDVKVPAENVLGEEGDG

FKVAMNILNNGRFGMGATLAGTMKHCITKAVDHANNRLQFGKRLNEYIGIQEKIAQMNML

QYATESMAFSISQNMDAGSQDYHLEAAISKIFASESAWQVCDESIQILGGMGFMKDTGLE

RVMRDLRIFRIFEGTNDILRLFVALTGIQYAGSHLKELQNAFKHPTANLGLIFKEASRRA

ASSVGIGGTDLSPYVATELQLHGKLAGECIDLFGKTVESLLIKYGKGIIGEQCILNRLAD

SAIDIYAMIVTLSRASRAVRKNIPTAEHELNLTKAWCSQASERCKLNLKHATSGERLNLY

KEMSVIAKSVFENGGVKTTHVLDQN

>GMOY010455-PA hypothetical protein|protein_coding|scf7180000652144:132761-148324:-1|gene:GMOY010455

MLSEIPSIARRQRRIPLKRDNNSLTNLRDCLVYVKMGQGTRSRAAGSSPQRKDNCVVDNE

TSVEAHNSPGRQLTAEELRVFDVGTPQGRGRPLPPATGYGALAKKLPAAIMPAAKSSKIE

TPTNYDQVVAARARNVYDEAPDYPKSPQVTSDEPLIPGARGQVPYNVIHKSQGKKYYTPP

DGPPVTSVQCRTVRVKPPSQPLVELFSGPTAEPPLPQPGRSAAHTPGQTPMRLMLPTSAQ

ALQTPQRSQSGDKSRSQTGERGTCPGLARRQPLQAIGAPQGKSGTSARPSPCPIPLGTPQ

SRPQEDRGRQPAIEFSSDKPREQSSGRAPNENYGGYIVRRSPSPPRSAPAYPQKQLQIYE

GAGDKSRSQSALRALEMVLAEPSRSIGDFVTEKSRGASPQIEAGRSEANPAAASAINQAG

SSKASSPANKASPDNDPSRSESPTGDNTGWIVADQLIGQDAPECEEMIEQRIADLRNLPL

LEDDPVYHLGRTLNTDEHIILDEILEDEIREIPGYQMSPGQTARKERRTEDKSTSAQAEN

VCAPCPSSRPTEQELDKPNIFKSIAKKIGITMEILSKLVHGRQPPAIEGPRAESSSQAKP

EDSGPNIRNLYTEEEPDDDVELPESYKACANAKKCGRIKFDKLNHCDDVAVNTATRQMQI

KELVMDNYCPGAIQEAIDGKEDFNAVAFELARVPCTIDEKACTNAPAEEPLSIIEAFVMR

IDIEREGMKKGLKKDASEEEIKSSLRKYFETEHNPTDLNKSINSLLMAEALTDVAIAKAS

KTVTFEADVQKWASSIPDNPNIKLVSYTATTNPVILEESPEQPASASKTPDPS

>GMOY010501-PA hypothetical protein|protein_coding|scf7180000652149:376233-377041:-1|gene:GMOY010501

MKLLLFVTLCVLVALVSSAPAPQQEVQIVEYENNNDGTGNYNYRFALSDGTKQDETGQLK

DLQGEDGPVQAVVKTGSYEFTDLDGKVHKVTYTADENGFHPVVES

>GMOY010558-PA hypothetical protein|protein_coding|scf7180000652150:569552-570595:-1|gene:GMOY010558

MQAPQALANFRCQTSCKHKECQRHANLGIMDKNECECAKEELSGSTIFLENVIDLSHTLA

QIMVICGLHECKAKSAVDIITYAFLHYDLVCYCLDEKPKKRLRKEDIFHELGRKCLMNRV

EAHLAYTIIKRGFKAFYYSPQIDETAKDYNGRPSYSDECVYVHAAKKTAESYAQFDGLSC

DEQRGVEAKIKVVYKKFYDRMQSRQSKPDHDQCNCCFCAKKRGHLVYGKPPPCVTTKPPK

KPHTCPHCCKRKKKAGIVHQQAYVAKCPVCHRSRKYCTCAKYDFGIEWVQSIWNRPDMNI

YYQNTIEKPQDDSLPHCRPQGAEWQEQVQEEEEDEEPAAIDENPMEE

>GMOY010728-PA Larval serum protein-like 4|protein_coding|scf7180000652156:398565-400907:1|gene:GMOY010728

MSNPINKKALELLFERPLEPIFTARDDGKAVFDIPDSFYSEQYSDVKEDIQSRFSEDVDI

KIPLRELTKKPDLTFTQPVGKRKQFSLFNSLHRSIAARLIDILLNAEDEDLFIATCAYVK

DRVNPFLFQYCYAVAVQHRKDTKNFEIKPIAEMFPQNFVEPAVFKDARAEGELVRNTGDR

RHIDIPRNYTASDREEEQRMSYFREDIGVNSHHWHWHLVYPGYGDDEIVKKDRRGELFYY

MHHQIIARYNVERFCNGLAKIKILNNVREPIAEGYFPKIMSSLNNRTYPGRSAFTKLHDI

DREDAKLEIADLERWANRIIQAIDQGFVTDAKGNNIPLDPKKGIDILGDIIESTQLSVNP

QFYGSLHNEGHNAISCCHDPDSRFLEDFGVMGDVTTAMRDPIFYRWHGYIDSIFNRHKEL

LSPYEDADLAFQGIHVSKFEVRIQTASQRASPNTLLTYMEKSDVDLAAGLDFGPKGNIYA

TFTHLQHAPFEYAINVNNADNVPKMGTCRIFICPKSDERGTLLSLNEQRLLAIELDRFAV

NLVPGTNNIHQSSNSSSVTIPYERSFRKLGPKYQPTDERQLAEFRFCGCGWPEHLLLPKG

RPEGMAFDLFVMISDYTGDEVQQAKDQPSVCGDSSSFCGLKDKLYPDNRSMGYPFDRRLP

EKTLVELTNKFPNMSMIDVVIRYNDVIVDRKA

>GMOY010851-PA hypothetical protein|protein_coding|scf7180000652157:1429888-1433199:-1|gene:GMOY010851

MLRIPKFLPRILTQHQANLLGGRSSSIFENKLMQANSVLNNTLAAQFSQRSGEVKGAVIG

IDLGTTNSCVAIMEGKQAKVIENAEGARTTPSHIAFTKDGERLVGMPAKRQAVTNAANTF

YATKRLIGRRFDDPEIKKDLKNLSYKVVKASNGDAWVQSSDGKVYSPSQIGAFILMKMKE

TAEAYLNTKVKNAVITVPAYFNDSQRQATKDAGQIAGLNVLRVINEPTAAALAYGMDKTD

DKIIAVYDLGGGTFDISILEIQKGVFEVKSTNGDTMLGGEDFDNAIVDFLVSEFKRDTGI

DITKDSIAMQRLKEAAEKAKCELSSSHQTDINLPYLTMDTSGPQHMNLKMTRSKLESLVG

ELIKRTIQPCQKALSDAEVSKSDIGEVLLVGGMTRMPKVHSTVQELFGRQPSRSVNPDEA

VAVGAAVQGGVLAGDVTDVLLLDVTPLSLGIETLGGVFTRLISRNTTIPTKKSQVFSTAA

DGQTQVEIKVHQGEREMAGDNKLLGAFTLVGIPPAPRGVPQIEVVFDIDANGIVHVSAKD

KGTGREQQIVIQSSGGLSKDEIENMIKKAEEYATADKKKRELVELVNQAEGIMHDTEAKM

EEFKNQLPAEECDKLKKSIADLRALLADKDKSEPEEVRKATNQLQQSSLKLFEMAYKKMS

AERESAAGNKDSTTESTGEGGSGGTSSSEQAKSQEKKEEKN

>GMOY010873-PA hypothetical protein|protein_coding|scf7180000652157:2010533-2011348:-1|gene:GMOY010873

MSSLKLQKRLAASVMRCGKKKVWLDPNEINEIANTNSRQNIRKLIKDGLIIKKPVVVHSR

YRVRKNTEARRKGRHCGFGKRKGTANARMPTKFLWMQRQRVLRRNVFKNKRVLMEYIHKK

KAEKQRSKMLADQAEARRQKVREARKRREERIANKKQELLSLHAKEDEVAAQAATTGKK

>GMOY010913-PA hypothetical protein|protein_coding|scf7180000652158:299241-325671:1|gene:GMOY010913

MSCNCCCSVPILYYDDLSPQARSCYMLIKVLEIDVELKPINLINGEHFSEEYSKINPSCT

VPALIDGNLTLYDGHTIMIYLCDKFAHAYNPQLCPKRYLTRLEVLNLLFYEGCVLYRRHS

HLLTDILLEKFPNVDIDYHKRKIADCYNALNTFLIGHCFMVGNCMTIADFSMISTVGALD

LMFPINRNLWPNLSTWFDRLRIMPWYKLNEDGIKKQQKLLQAVGEFPFPSPLIITELMTP

HSSFRADGYESIINQVLDEDESIKRQPSSETGISQALHETETDERPRTDDQMFQRDEPAE

IQPISETTVTRSSREEAYEVTEILYQGETNEGQRSSGTIPALTLREENRDDELRTERLSV

DSRKEKSSGGSRIERLSIGSRAERSERMPVEQKAEQVQRLSVGSRTDKSIISERTERSAV

ETRSGRSSAEARSEKKEKLRDAERASAEYVQQGFIEIPTTQTSQRQAPQTIDIDEPDMIS

SVSKLSVDNREFSREINVEEFYDVKFKPSGEDSENEVEDKCGCIKDVRKSYFEIKNLLKR

QSEEFKKAIQKYPEFLTVKAKRHKTSCICPDCAIANIGQDRYDMQRMKLELEFYKKDCCE

PENLRIKNCDCNNNQEGGQDDGYYYDEDYEATTLVRLKERHQRPGGGQSMNYDEDYVENE

TYRDYFQTLHADQMYKLQTIVDQNEIDYIDDNDGNDDTEYYNDNVDNGDTEDNDDNDENF

MGVAEELQNHQNTFVLKGKQEFEGESTDSCSQDTADSNKNLYSKPKAQMLFCQHGETEVP

PELTTVNESLKNYNTHAFTNTFATSTASDNLDTEDLDCHPEFLSTTSAKESPSQQDDLVE

ENVSGNQIETPSNIHQKHQENKEPGRLKDPIKTQENERRHSTSAQTILNFGRALSDTILN

TAKYNSEGKFGFISNVSGIAITERPNPSFEHGPKKTKKVNSKEIIINGIDTIETTKKAVA

EHQENKESYAFKKDEESKSRIDDTKHKERCDYNKQYENAQKSISKNEKYIIRKQNDYNNQ

RAYNESENLKEMKNGQTKEKLTENVSQQPEKTKSNSQISTGKVLIPNSGNSESDQKVSTY

TKTEKNDEEKCICKRARTSSRHRQYRSPSCSLADFRKCTVCREKRKLQIDSQSKRYKIEI

EAKGCKEHIHNQFGQKPITEGKEIADKLKFKGNTGVDNKDNVHDCTIKNEKNVRLRAGNC

GSKHLQICYMDEDVLIKMPQDLKSLVEKTNSSESVDNCNSDSLCSLTSISNASEEIACVN

YYDHYKDQRPIDESNRVCFHHSKYLCRRCLKTLTSVIPKETCVLPHMYGSRECMGTAPLQ

AASGCHDLTKPGLFTIPEYSGCVCGCKCRKCLFKSVFNYPGDSSQLWSEYVRYPIHEKNM

NDFYELPKVNSTYLQIRDKVRRKAMGRQQESEDLKVKSSSSEINKFAKLKQHLTPGCKSL

FSLSDNDDSDSEKNNGFFNYPKTLKDRRPDDMYGTPYQQKLKKLTFSTSSENKIFSEPES

EVHVKCYDRDSATAQSFKSRIPILLMKKRVDCSPTRETTSNDEKASKIAGKRDPQMDKEA

QEWVEAILGEKFPGGVAYEDHLKDGQVLCKLINTLTPNAVPKVNSSGGQFKMMENINNFQ

KAIKDYGVPDLDVFQTVDLWEKKDIAQVTNTIFALGRACYKHAEFKGPFLGPKPADECKR

DFTEEQLKAGQTVIGLQAGTNKGATQSGQNLGAGRKILLGK

>GMOY010969-PA hypothetical protein|protein_coding|scf7180000652158:2131769-2132326:1|gene:GMOY010969

MVLKYGKMLIHHHLYKSLRFVPQLVVKRSTTSDHCNQMSGKREYVGYGINGSPVYMDLPE

YPMPAIRFREGGDELCALREKEKGDWNKLTLDEIKNLYRGSFCQTFAEMHAPTGEWKLAV

GIAFWAVAIAFLMTVLTHLSESLPESFEEDSRQAQLKRMIALEMNPITGLASKWDYEIGD

WKSRK

>GMOY010992-PA hypothetical protein|protein_coding|scf7180000652159:547546-549189:1|gene:GMOY010992

MLRLNAFGHAAKLVSQMRNYASQAINQMLQLQHTEVCADPPSRGVIFGVYADENDKTDTG

ILTAAGWKYNVQRTGGRMIEILRMSGPMPKAGESRLFFAQETEKIPYYSAVAIVGLGKEC

LGYNPYEVLDEQKETIRRSVAKACMDLALLDTTRIEVDNCGHAESAGEGAALGVWCYQEL

RDKKNRIAMPAIDLYTTKDDLCDMEGWRIGVQKAAAQNLTRQLQEMPANILTPTAFAQNV

VEVLCKSGVNVEVKVEGWAESQLMNAFLSVGKASCEPPIFLELSYYGTSAEERPIVLIGQ

GITYDCGGLCLNPMEKLYVMRADMTGAAVVVATCRAVAALRLPVNIRGLIPLCENVMGCN

SFRSGDTVKCMNGKHIKIQGTDYEDVLVLADALSYAQNFCPKCIVDVGTPSWDMRKTLDE

AACGVFTNSEILWQQIKHASMHTGDRVWRMPLWDYYTKQVTSGMSADVQNYGIGRGGKPC

KAAAFLREFVPCGQWMHIDATSVMFTTGKHFEYLRAGMAGRPTRTVIEFIAQTICKDTAP

KMPKKEK

>GMOY011014-PA hypothetical protein|protein_coding|scf7180000652159:1018271-1019242:-1|gene:GMOY011014

MFSTCARLIRKSSPAVLNYSTLSTAQSGRLSKLSRNFSLSAVENFHTSRSDLISPSSSHC

FTPVIIHKRNFAKMASFETNGIVPDVIDKAPGELMNVCYGDVQAKEGNVLTPTQVKDQPE

LSWNAEADSFYTICMTDPDAPSREDPKFREWHHWLVVNVPGCKLQNGDVLSAYIGSGPPK

DTGLHRYVFLVYRQKCKREFDETRLKNNSADGRGGFKIAEFAKKYELGTPVAGNFFQAEY

DDYVPKLYEKLGVKTEEPKKDPKAKGAKAGEKPKETKAEAPKDAKPEAAKEAKPEAAKEA

TPEAPKEATPEAPKEEEQKAAAP

>GMOY011142-PA hypothetical protein|protein_coding|scf7180000652160:3481178-3491236:-1|gene:GMOY011142

MQNITALGTPNNSYHYSNYNHHSSNDDSINANPNANANANKIANDNDNAYDNASDMCRGN

DNENDNDNDNNQDNNKDIDNYNDNEKHNNNGNDNANDNENRCDNDNDAGNDDDNDKDNDN

DANKFNDNAKDNIEGFYNISRMTLQCVYNRAIVILSGNPMEAIELKCLRPPFRMHRATTI

DNGYYEEFPWCPRSSLFFTYATADSTKEDVFTGVLCYDIRLSSTKVLYYEYVPWERRKIL

PPTVPATYVGEEHDNAVPKDQNLMLSRHYNNEEFRHWFHFGQFEYASTVPESVSEQLREN

LSSLFNIMWWSYLRMGNWKRYQDKLREYVGSSGGMKILSGTFGEVRVPVNESGIYNQFVM

SALKDDSGRKIFEYIWNDLESRQEGEKHVVIFGYNSPFYEFFPENAIVFCKSMCEEIEWL

AGIQETFRYPNMGVIFCCLHEDVVKLNLIEGLGKKMRSPPDFEKPKGCSSSDENNPSKHE

DDSQESNELDYPSDKSVNSSFEWGKLKLMKVFGKKMRSPPDCENPKRCSYSDENDSSEDE

DLDNSQERNQLDNGSAESVESSFEQGGRRKGCGKSCHDGHRFGKKYVKSGKKLHQSDHHG

KGCHKCHQKSCRSGNHPYRRDHHCRNSDESYEDSRERNQLDNEWDESTECSRERTHRHRD

CAESGKNSHRSGHHGKGYHKFHQESCRSGNHPHRRGHHCTNSDESYEDLSEEDRHIRHAH

KSGLRERNHHDRNSDESHKDLLGEDQHNRNAHESGEDSRERNHHGRNSDESHEDLLGEDQ

HNCNAHESGEGSRERNHHGRNSDESHEDSPKGDQHNRNAHESGEDLRERNHHGGNSDESH

EDSPKEDQVNRNAHESGEGSRERNHHDKNSDETHEDPPEGDQHNINAHESGEDLRARNHH

GRNSDESHEDSPEEDQRSIDAYETGEGSGERNHHGRNSDESYEDSSEGDQHNRNSDKSYE

DSREKGQHKRNAHESGEGSRKRNHHSRNSDESYEDSSEEYQRKRHAHESWKRNHHGRNWD

ESYEDSWEEDQYNRYLDESFEDSRELWQDNYWDELTEKSPSRGRQTKNLAKSRQERHQSA

NDSPRQAQDDEDWDESHKKSRKRHKYDNVRDDLRHLDEHDRKSHDRRLGTNSHRPNHHGR

DYDKSYRNLGRNDLNDRKRSKYAENRNQR

>GMOY011172-PA hypothetical protein|protein_coding|scf7180000652160:3801656-3803149:-1|gene:GMOY011172

MAFQSLLVLEKPLQHLALSDWNNRLNTLRNVADARRGDAFRIRHSSRNLRNETRIEGDWT

NYETNEALADRVSELNRWRDAIANTFERIEREMKLLSEEKCSTERELEAMQNPLSVIGEC

LTMRDCRLGAEMTYDDADTEIKNELCVLENNQLLLADQCQKAWEKLCRLEEVKFKLGLEI

GNKEEAEDLDMAQLALDKFAANITYKPDSTRIPKNSCSYQSWLEHTKNMKQLAENELADT

YAIREALFVCREKARNMLTSQQERAEHSIRKRIFETQRARNELEWQQLKMKEEMEKAVCE

IKTLEQALRDKTDGLKLAETRLENRAQRSGMELCLDDAHDQLCLEVHKLRDIRRRLIDKI

DEAKTNYNLLEEHAQKIDVDLENKQHSLMTDIRALDLRQRLKGGEFGAAKPSAQTDRNIE

LTKMEKEIPKN

>GMOY011236-PA hypothetical protein|protein_coding|scf7180000652160:6105352-6106673:1|gene:GMOY011236

MAINFHKIPSDLVIPEPPKYAHTVLDEIQARSALIDERQIKCKDIGPLYEKPKKRPQRYS

YKYYCVCPRYNPQYLCQHSGNIIIDRDVLTQEEHLVYLSTPKANFAAPRKMPRYYEKKAI

VPNCTAPSKKKPDVEYTDINTALAWLEEERRLTLVAKRLNKQRCKKLSKKIANKQRSQIK

KIICVLFEEMKDFLLNDQFIMDERSTLVAVILETIREFTAVWINKFISNLNIHVAQAPAS

PQRDQPFADSRRQQYSDKPSQALQNFLPVGDYISYSSASDEFMDDENEEFYSVKNPPTMP

SEDVEGETEKTLKTEQAEQAEQAEQAEAE

>GMOY011430-PA hypothetical protein|protein_coding|scf7180000652170:4696909-4697649:-1|gene:GMOY011430

MSNISQSWERVFCTIAPVRNFVRFISRKDCAKVLEPKCKPEHLEKQPPCPEKLRRLQQGI

KEPPTCGMWECPECCLEHCPEVQKRLDELYYKTSDKLNRKYQQTWIACPDLKIKEVEVCC

GDTEGMTVQKKAKRGKGVRPKTACPQPNKLKGLMVCKKAETKSKCPRFMLGNCPPARTPP

DCNQSRPPSKCQKDPAPYPCYSECKKCELDALPPVECKCLDKPAMCEVWAEFRRRLSFVK

SKESKE

>GMOY011451-PA hypothetical protein|protein_coding|scf7180000652170:5288482-5296892:1|gene:GMOY011451

MTSCWEGKKYKLEKSENFDEYMKELGVGMVLRKMGNSVSPTVELKLDGEKYSLTTSSTFK

TSTITFKLGEEFDEETLDGRKVKSICTMDGNTLIQEQKGDKPSKIVREFKENELITTLTL

GDIKSVRVYKAV

>GMOY011501-PA hypothetical protein|protein_coding|scf7180000652170:6104770-6119391:1|gene:GMOY011501

MPEVSAVLLECPKKYRSRSYQDNSESKHERKACEPQLMVNIRVNPSFHIEKLEKFLILDE

VYDPRKRTKAKILSPYLIELSRGEPVMMYPLQFSKTVKLEDINNKECVESGHHNIGNYMT

TDSANNGNSNDGINTGNNDVGTEINKEEHGPEEEEQCPHNEHTTKCDEMLESSGAQSTQS

TYASCKTSTVMPSTCCVPLTTQPPNKCPANRLSPEDESLNRVMQRIHQSFAPITKISENR

PSGNIKQNHKNKPRIGDPTDFSEVFKRPHDQFDNPFSNTAKSDKYPNVWNKDRFMIHRPF

NKKSKAKSKRPHNRVSAVSAMRSKQRKNLFQPKIFDDLSANVGVEKPFAKRLLGNKLAQL

GIITSPRAERKVHKKGRIDDIKNAFGDENDDDDDDTLTFSKDSDYDNLLEKTSEDINDMD

KEELSLRSSNTYEKEYQDAEERVDAILRDRKKANENRKRFQVGRKHHRYPDEKLEENAAD

KANFKSCDCKSAPRRGPEESNDRMELKNVGKFANSQDLDEDNDLKDESKVDMNENDALSS

SEMQIASLDEKPNKEASFYSRQKTKNGKRRRMKNFRKHKEDKKFDYNDNFMDLNVENNMR

EERAHNVYNRSPDTLRSPSSMPIIAGIPLDEPSPKNSKKDDDLDEDDELYYYLNGDHYRE

GQKGYWPSKYGEDQDKEKERAKDINFQQLKTHNEELYQPILYGNYFNNPTLMYHYQTEKS

QAAAKTTAATAINEDETTDNFAPDFMQPSKAEFDNMYKYPNAFRSMLPHARKMYSEYMET

TTQFDRDEDDLFTDKNLSIEDKMRVIDATTNIDDKIDFNKISAEIDKTASQEVTAFPYLL

SKPKTKHKRSFLFYDDMEMPDNIDENQGIYDHLLPAINNEGQPLVGDDGVQPCATCGGKG

RKPPPKSEPEQSSNEKYLNCNCRNDPSAICKCDKKRRAPTVRDACFQHECAFAVPYDSAE

DFPKTPRSLVENCSIDHLQNNQLLVPKDDRPTRDYNPSIASKYLNSSDGSENFKNLHFNP

ENLLAVDPSRLTSDVLQMDMIEFDKERQQIDSPALKTNIRNTLWTGLLGHIRPMRSSSYP

KSNTDNNNNNNDGIKKIIDFGLFPTSLNVLEYCPNAKNQLCLNMLDDRVPAFTMKVKMPF

RENSVIYYNMSKVKIAKIVTDATTKDALQVAVDLVNEGMQVREFSICVCNCPTACASAAK

TTVTKVLLPHIRETVTFLLPLIVEANNLKHKKFNCELIVRTISETPRIIEENLEILSSSH

HKMFKENQTDGNVSSTMEKRFASVKPKQKIVAIRKLSVDPDNRCLCVWECKCHCVAKLET

MVDFHICQELSQEEKRNAGLLQYADDLEISEPVEELSSPPSKEHHHIHIPLPHRPHFPSF

HIEYRGFLANAIFLLLIMILLGLIKAILGLCIKPINKCGYDYVQPARSYKRSSRLRRFCV

NTFFFIVFPFLFWCKCFTPSEEDLLVASTEWPCSHEHEQTSDKSRMKAGKLHRAFTFSND

DDDDEEDVILSQHLDLQSLKSKPDSRMGGGYDVPSLHDISDLDLAFDDDIFDDNDDEDDE

EANTKFILKALAECRESLKRLASQYHQDMSPNKVEALPEVPCERTKSAEEFVKKLKEAQI

VYRTFSQPLGNMRQIPPDFKYCIQGYFLPSLGTGYEFVNYNPLAQHRGLSADRKTMLTLR

PPLLLCPKDFSRRYNNKLDVLEAGDLSEQPPVGIPCINISALASVATSMAKLASENCTES

IIDQESISFSLPLSSKETSSTDAQSPYNLKDEQDLQNLLTSDLNLKNSCTSNDIEIIDDE

NNENVERAAIFAAPEGYKYREPVARLEQGNAQANAFQPIVVSDLSCKNNVESGTDTLHVI

NSQATGRVVNHEQQHYHGQLSGKLQTHNYNTIPQVIPTQLYHHSQEVNGQTGEVLNNLTP

PSVQLPINYSPAVQLTPQVPVQSFHNAPNTGADQTSTYFAQQAAGLSQSVNLNNQQHYFV

AGAQQQQHQQHQQQYQQQQQIDKGFAKPIHTNHLPIQSNINSFGSAHSLSSHHLTNNNFN

AIPTFGLNSSPLHTLDGQLSQVIQEAYSTAPLDPTIEKHIYVHVPPEELEETTSSLKLPP

QSLPIAPKKHYKIIFIKAPSVSTSSHYAHLAAAAAPQVEEKTLVYVLAKKPDEPSAEYLQ

QLQQTAYKTNKPEVYFIKYKSHNEQKTNGIINIGDSFTDIRTNNESNNVDVSVTSSSSSA

TSSSVTEDHKHQVYGVPLQ

>GMOY011527-PA hypothetical protein|protein_coding|scf7180000652170:6604280-6670865:-1|gene:GMOY011527

MAEDEKKKEKVDERPEFFWNYITKTMRLKQEKWTKCMTANDFREIINTFVNDAYQERLIF

TLNAAAVLVPSFNFPEKPTSKVVYFIRNDVPTNLTLQNMSSALMIGDILPNVLENLSVIC

DDVIFPLLNNPVNQNGWTSVIVNDMKTESQDLRNGIAQMKGLVINRTILPLPICIDEVME

SAPAIAKGDLGKVNHLMKHALEFMVVKWLDSVEDLVHVKARDKIFSKDEFPRPEHLMGFW

ETRLENLENLADQLGDKRIKTIGFVLERIRSVFESSYRRIVELVLEALAEARDITKYLTP

LRKVIDKFETADMDENRANVRPLLLTVGLVWGHSKYFHTLDNMVLLFQLLHNTLIECAIR

TIEPDAIFQGDVDEAYKKITTNINHLEYYRSTYKDTRGSLKKFKVGTEFNSQDWTWHPSE

IFARFDKFIARLETLGELFETGRDFLKLEKVTVGGLKGRQITMAIEKILEEYNGYYREWS

NIQYNPLDPDYQGSTFESDRVAFKEKTDILERKIAYQFEKALEDSHDLLLCGTLLLRPII

KAHIDPFMHILIDDFADEIVSVKVEFNEFQKTCETEGITALNTDMCFPPVTGALSWLNKL

QYRITWIRTDYELYDYPIFENEHGQQTLEIYEEMLDHIERLRKDILLYWVQEVKKHIKEG

MEFTLLAKDEKGDLSVNFNLNLKNALKDTKYILLMELAVPEDIKQFYEKEERLWSARIKL

QRISEWYNDINYRSKPSEKALILSELTSYEIFMEPLVSRITWNNFEQKFIVDVFKKIQYL

HKRLERCQANVDAIKRSINEWGKVPLYQRKDKNPKAFLETEPRMDILAERVAHAGITSVL

IDRIMYENAKLFFDIPRRLYLMDEVEEEEEEYPDDIFDDKKDDEGEEDDEEDFITAIHRH

ELLQTLTQEERDLFRAYETYVDQEITQQLLDAVITSLTYLKMEIENRYENDFPIFEILME

LQEPHVTYFLNLDPTSKAGFTFHVETLLDDMYHMMEMLPRTAQDPAGNDDELLDFGDEIA

DKAEIHKSRTDILTKVKFGLQSVRTHSKPFMDYSYLWMLDKQQYLAEVKKFGRPLTLSER

EAELEVEGASGVKPLKDEYPPLSVYKEQLDKFIALEEHISQWETYQDINVWLRLNKLGFK

NAVLNQVAKWISLFKTDLIDRVKNSLRELAIFVEEANEALKIELNKDDFDGLLKILSVLN

TINEKQFIYDYMFEPLREIVDLLKTYNYEFKDTELAMFFNAFCPNVYEIIDEADLEIVAL

EDRHRSLTSSAVLFELQGPDPTKIERCRRDLQLIKIMWDFNITIASTIEDWKKTPWKKID

IESMDQECKKFGKELRGLDKEMRKWDPYLQTENSLKNLMTSLRAVTELQNPAIRDRHWIE

LMQTTKVKFSMDDSTTLKDLIDLNLHEYEEEVKNIVDKSVKEMAMEKVLRDLHNVWATME

FQNEIHERTGLKLLKASEEMIEVLEENQVQLQNMASSKYIAHFQTEVTNWQIKLSNADQI

IGSWFEVQRKWVYLESIFIGSEDIRAQLPEDSKRFDTIDREFKAMLTQMNADRNVVRSTN

KPGSKLYENLEHLLHLLLLCEKALNDYLETKRLAYPRFYFVSSADLLDILSNGNNPRMVA

KHLTKLYDSLGSLNIAVGSKSAAGMVAKEHQEYVPFLEPCDCSGKVEAWLTRITDKMRET

LRDQFRRAVISYEEKPRHMWIFDWPAQPALVTTQIWWTTETNDAFAKVQQRYENALKDYN

KKQITQLNHLINLLLSDLTASDRQKICTICTIDVHSRDVVAKIITAKVEVVTAFQWQSQL

RHRWDTKFEDCFANICDAQFQYDYEYLGNTPRLVITPLTDRCYITLTQSLHLIMGGAPAG

PAGTGKTETTKDLGRALGIMVYVFNCSEQMDYKSVGDIHKGLAQTGAWGCFDEFNRISVE

VLSVVAVQVKCIQDAIKAKKVIFNFLGEIISLRPTVGMFITMNPGYAGRAELPENLKALY

RPCAMVVPDFALISEIMLVAEGFQEARLLARKFIALYELCKELLSKQDHYDWGLRAIKSV

LVVAGALRRDDRYRPEDQVLMRALRDFNIPKIVTDDLPVFMGLIGDLFPALDVPRKRNLD

FEAVIKRSAVDLKLQPDDGFILKVVQLEELFAVRHSVFIIGFAGTGKSEVWKTLNKTYSN

QKRKPHYNDLNPKAVTNDELFGIVNPATREWKDGLFSIIMRDQANLGGTGPKWIVLDGDI

DPMWIESLNTVMDDNKVLTLASNERIALTKEMRLLFEIANLKTATPATVSRAGILYINPQ

DLGWTPFILSWLNTRTNQSEISTLNVLFDKYVPPMLEVFRLRLKKITPISDIAMLQMTCF

LLNSLLTPQNVPADCPKDWYEIYFVFSIVWGFGSTLFQDQIIDWRNEFSKWFLNEFKAVK

FPGTGNIFAFYVDHETKKFEPWTKLVPDFELDPDIPLQANLVHTSETTRLRYFMDVLIQD

NYPVMLIGPPGSGKTIIMNSKLASLPTEKFAITNVPFNFYTTSEMLQRILEKPLEKKAGR

NYGPQGNKRMLYFVDDMNMPEVDKYGTVQPHTLIRQFMDYHHWYDRVKMTLRDIHNCQFV

SCMNPSAGSFTINPRLQRHFCSFAVNQPSPDALFHILNSILSQHLNNPIHKFAKSVISLC

GPLVQTAILLHQKVATTFLPTAVKFHYNFNLRDIANIFTGMLYANFETCPNPNLLLRLWV

HECNRVYGDKLVDYSDINAFNKVVMDMVRKGIEGFNEDAVFSKPLIYCHFAKGLSDIKYM

PIPDWERLHKLLEEAQERYNDFVGAMNLVLFEDAMAHVCRISRIVESSRGYALLIGVGGS

GKQSLTRLAAFISSLDVFQIQLTKDYGINDLKVNIASLYMKCGVKTSPCCFLMTDAEVAR

EQFLVLVNDLLASGEIHELFPDDEIENIINAVRNEVKQLGIVDNRENCWKHFIEKVRGML

KVVLCFSPVGTTLRVRARNFPSLVNCTTIDWFHEWPRDALESVSYSFLSEIDVLPKDLAK

PVSRFMAYVHKTVNDISNVYLLNDKRYNYTTPKSFLELISLYTKLLKEKVRANQDRRTRL

GNGLIKLASCSKEVDALQDVLKVQEVELRIKNEDADNLIKVVSTENEKVSKERAFATKEE

KNVRQIEEDVGAKAKLCEEDFRKAQPALIAAQEALNTLNKNNLTELKSFGSPPEAVVSVC

AAVLVLFSQKGKIPKDRSWKSCRSIMGNVDKFLNDLVNYDKKHIHPDIIKALQPYIVDPE

FIPEKILAKSAAAAGLCSWVININRFYEVYLIVEPKERALVEAEQELQDARDKLTALNKR

LNELEEQLNVLQSEYDEALAKKQKCQDEADKTAFTIDLANRLIGGLASEKIRWTESVKNL

LSSQITLPGDILLISCFISYVGCFTRPYRTELQVKMWHPAFKASDPKIPSSEGSDPFEMI

CDDAQIAEWNNQGLPSDRMSAENAAILVHSDRYPLMIDPQLQGIKWVKQKYSSNIVVLRT

TQKGYMEKIEHAVSNGNVLLLENIGENVDAVLNPLLGRMLIKKGTCLKMGDKEIDFNPKF

RLILHTKLANPHYKPEMQAQTTLINFTVTRDGLEDQLLAEVVKAERPDLEVLRTRLTQQQ

NHFKITLKFLEDDLLQRLSSAGENVLEDVSLVMNLEKTKKTADEIEVKVAEAKITAVQID

TAREAYRPASERASIIYFILNDLFKINPIYQFSLKAFTVVFSNAILRATPAEKLKDRVEN

LIDSITYCSFMYTSRGLFEADKLTFLTQLTIQVLISAGEVEPSELDFLLRFPYMVNVTSN

LAFLTNVAWGGIRALSNLPAFKGLDKDIEGSHKRWKKFIDSECPEREKFPGEWKGKSAIQ

RLCIMRCIRPDRMSYSMKVFIEEKLGSKYIDARSMEFAKTFEESSAETHIFFVLSAGVDP

LKDVEKLGKVLGFHGDHENFHSVSLGQGQEIVAENAIDTASKRGHWVILQNIHLVARWLP

SLEKKMEASLMDVNPSYRLFLSAEPAGDPAAHILPQGILESAIKITNEPPTGMQANIHKA

LDNFSDETLEMCSKETEFKAILFSLCYFHAVVAERRKFGAQGWNRVYPFNVGDLTISVYV

LYNYLEANNRVPWEDLRYLFGEIMYGGHITDDRDRRLCRTYLEEFMQPELIDGELEFCSG

FPAPGILKYAGYHSYIDENLPPESPNLYGLHLNAEIGFLTTVSERLFRTVFELQPRLAGA

SDAGGGEAQSQEDVVKGMLEDLIDKIPSPFNIYELMARVEDRNPYILVAFQECERMNILM

AELKRSLNELDLGLKGELTISSIMENLMQSLFMDQVPESWTKLAYPSTLGLQSWFADLQL

RLRELEGWVADFRLPSSVWLGGFFNPQSFLTAIMQQTARKNEWPLDRMCLNTDVTKKTKE

EISSAPREGAYINGLYMEGARWDMNMNTIADAFLKELFPAMPVIYVKAVQKDKQDTKNVY

ECPVYKIRSKASMEPPHACEYIWKGLEQKIELNFPHISVYMLILLNWLQNGQHRVLVVTL

NLNGTLIPTNSINEISQQYVIPNNTQKVALIAPNTSTELSTSLLAVSAAMNATACLLPIP

ATSSSFSTLDGVPRGKCVYFLRKQLNIPLINENFRKHVIFGDFPIHSKMETLSVIFEEVI

KPLLQCAQNRKFRSDMQNKDLDALVKDIHTNLIEINGVLKEKSEYNADARRVKNDIINVC

LPVHEINFWINRQENLQNIYNQLTTCTHKAVTEILEAINSTYCPPFKKIFEQLIYALKES

REISIWLKPLLQHTIQFSSVHFNNAHHLIVPLVHVIHLIWSGARYYKSTTRMSTLLRCIC

NFLVCRAQEDLEVANLFNLDADEGLQKISKTLEVLELFKLTMTQYKVKYSNNQTILPALT

SLAVEVIKDSNNAFPSRQLWHFSDTEVFGPTMDEFLDQLMQLREIFDVANLFLCLEKLEI

GGSRGKTLTRMLKKLFRREKNVFFQRIRVLKKVLVNVLIQAFNGYQNWEQISKLTIMFGN

ILHGDIISSEFKVILPHIFNIYADELKSIENSINPVLLGFELRGVDAISLLGNLPPVAGA

LMCIENYIKRCDALHAYEINDLISLLLKVDDDSGKSRYETLTSRRDILITKLKQLQLKIW

LDWKQKIDKNLEIGLRSKVFVLLSMIEENNENKQEDGFAFSDDDGMNNKMLNIHPSYELF

TLLAEINYLLAFRQLWSTEGNTFLNEFPQVLWDVYEERDDLWQRKIKLIKIIHYYNAVQE

QISNDGKFKLIASEIDSVNNLVAKACQTVTWQNYEYLYNEMNANNTTNPAERLPSVIGKA

QKNRPISEHSRNQWAMEYNASLCDQRTFAQTPLFEVELHLSDIKICFKPSFEENEKNNFQ

QFFNQLIADTKETYKYLLRSFDGDSLLSQFKNNNEIAILRAEVEAGEINSTASASVTKSD

LLKKRIIAIREKLNESINAANLYAAQFEIYSHLWNEKYIDVLKKNLRDARNFESTCYYIL

DVFKQSKYDNFIERGFSLLQLHIKRDDLGTLLQILTIIRQIHEHEAETDNIFKPLKEAVA

LLKSYGVCFNAEFLRKVDHLPAQWNQLKTLATSKSEALRETKLYQQERIATLTMIFSCYA

QSFAKRFHRMAFFKVPCPEVYSLCDRICQKLMYYFTRHQKISNCASVLEIEPPDYDVLQK

CKTQIKHIKQLWDYVNVIESSVNHWRATSWLKLDIDDIENYCKQFTRDLRSLDKSIRDWA

PYLYIVRLLKELNSSFRTVTELQNPALTERHWMEMMTELKISTTYSSDYLHSYLLQHFLL

NFQKLSLKINPNTTLDELSSLGLYKHEEEIKNFVDRAVKEMAVVKMLDEINKIWSSMEFY

IEPHHRRQEINMLKVSEEFIEILDDNQIQLQNMTALKHGTYISEKINYWQNILAKVEIFI

NCWLEVQRKWMYLESIFIGSPDIRAQLSQDTMHFEEIDENFRSLLKKVVAVRTAKAIVLE

HEDMFAALIHLQEQLTLCEKALYEYLETKRMTFPRFYFISSADLLHILSNGSNPQIIDQH

LIKLFDSILRLEYNVGTTQAIGVISKENDEYVQFISSYIECSGKVELWLTNLIEEMRYTL

HELFDRGLHAYAKKPRENWIFDWPAQVALCCSQIYWTAEVNKAFAWIEEGYEAAMRDFHK

QQVSQLNTLINLLMSDLKPGDRQKIMTMCTIDVHSRDVVNKIISSKVDNSSAFPWQSQLR

HRWNSNDNWKDTASAGYPMDCFANICDAEFQYAYEYLGNTTRLVITPLTDRCYITLTQVK

SSIFISSAVCLLRKKMFSSFTLKSLHLVMGGATAGPAGTGKTETTKDLGRALGVMVYVFN

CSEQMDYKSCANIYKGLAQTGAWGCFDEFNRISVEVLSVIAIQVKTIQEAIKMFKKKFMF

MGEEIALKPSVGIFITMNPGYAGRTELPENLKTLFRPCAMIVPDFALICEIMLMAEGFQD

AYRLARKFITLYTLCKELLSKQDHYDWGLRAIKSVLVVAGALKRDDRSRPEEQVLMRALR

DFNIPKIVTEDVSIFTGLIGDLFPAIDVPRKRRFDFEKTVRLAITEMKLQPEQRFLMKVV

QLQELLDVRHSVFIVGNAGTGKTKTWQTLYETYHMQGLKPVCNVLNPKALTNDELFGIVN

AKTREWKDGLFSSIMRDQANMTQNNPKWIILDGDIDPMWIESLNTLMDDNRILTLASNER

ISLKKEMRLIFEVGHLKAATPATVSRAGILYINTQDLSWSSYVSSWLETRSDLIERSILT

TLFEKYYPRLMQHQQNFKRIIPIPAIAMIQISCHLLESLLETSHKSNSILVETSRISQPC

NSQHNNNTAETMETQLALMFTYATMWGFGSILHEDQIIDWRREFHKWWTSEFKDIKLPSL

GTVFDYYLDVQAQKFTPWTDLACQTPCNLIDLDTPLQNILIQTAETTRLTYFLETLMERN

LPCMLVGTSGCGKGSVFRELFKKYTSYQDAITIKNSTASTVIQEIHFNFYMTSEILQKIL

EYPLEKKSGKFYGPIGSNRRLIYFINDLNMPKVDAYGTVQAHTIIRQFMDYRQWYDRQHL

RLKNIRKCQFVACMNHTAGSFTIDPRLQRHFCIFSLAAPSEETLHYIYGTILSSHLENSS

NNFSKEIRMIGDLTVRVGIYLHRRVEYLFPATALKFHYTFNMRDLTNIYQNVVNSVGVSN

AGSNAALTNFTGTICSKPSDLIRLYVHEAFRVYHDRLVDQFDIKSFKSSIRDVFKKDFED

FDEEYVFSEPLIYCHFTQSLVDQRYMPINGWKRLYESLTEALISYNDVSGHMNLVLFEDA

MKHLCRINRILESPCSNALLVGVGGSGKQTLTRLASFISSLSIFQIQIKKGFCSQDMKDE

MAALYMKVGLKNIATVFLISDTQIPDESILIYINDFLASGEIPDLFSDDQLESIMNTIRG

EVKQSGILDIKENCWRYFVMKVRRLLKIVLCFSPVGHSFRRRARKFPAIIKHTNIDWFHE

WPDSALESVSQKFLNEVSENVLPHNFIKPISKFMAYVHGTVNQISKVFKQNEKRFNYTTP

KTFLEYIFLYRKLLIDTSDEYTKRINRLESGMAKLAKCSLQVEILKGQLATQQIQLNAKN

SAADKLIGVISSESEKVRNEKNIASEEEQRVKIIEEDVLIKTKTCEEDLRKAEPALVAAQ

AALNTLNKNNLTELKSFGSPPKAVVNVCAAVMVLLAVNGKIPHNRSWKAAKLMMDPEFNP

DKVVQKSVAAAGLCAWVINIYRYYQIFLFVGPKQLALKDSQEELKNAREHLHYLKSKIIS

LEHKLGQIQAEFEEAVNAKQKCQSEADKTANTIDVAHRLVNGLVNENIRWKQSVQSLIAK

IATLPGDMLLICSFLSYVGSFTRRYRDELQQKLWIPNFKKLQPAVPHTDAENFLMLFTDD

AQIAIWNNEGLPMDRMSTENATILVNSTRWPLMIDPQLQGIKWIKNRFGEALIVIRLSER

NFLEKLESAITKGDTVLMEQIEETVDTVLDPLLSRFLIKKGHYIRIGDKELEFNPNFPQT

TLINFTVTPDGLEEQLLAEVVKMERPDLEEMKAKLTVQQNKFKIYLKDLEDDLLARLASA

KENILDDNELVRNLEATKRTTDDIEIKVNETRATAMQIDDARNMYRPAANRASLLYFILF

DLGRINPIYRFSLKSYMHVFKQAIKLAAPESRQNKRVALLVESITLQTFHYTIRGLFEVD

KLTFTTHMTLRLMANSKYICAEEIDFLLRYPHDPNTSAPVDFINSNSWGGIKSLSLLEDF

YGLDKDIETCPKRWRKFITSEAPELEQLPGEWKHRTRLQKLCILRAVRPDRVSNALRDFV

EQTMGKEYGTIKSYPLGEILKEISAYIPVVFILSPGVDPIRDVEYIGSKLGFTSEAGSLI

NISLGQGQEIVAEQAIDAALKTGQQWVVLQNIHLVKHWLPTLDKLIENISSERLPIAESN

FRLFISLEPAPDPQYHIIPQGILESSLKIVNEPPSGMAANLHKAWDNFSPDTLETCTQEA

EFKSILFALCFFHSVCSQRYKFGPLGWNKAYPFNMGDLTISASVLRNYLEKCSHIPWEDL

RYLFGEIMYGGHITDDWDRRLCQTYLEEILQQDLIDGDFELCPGFPSPPNLDFQGYHDYI

SEDLPVDSPTLYGLHPNAEIGFLTNMSEQMLKTIFELQPRQSDLSTNYGAPREELVRIMI

DDFLDKLRDEFNLQALLNRVESKTPFVVVALQESERMNMLIHEIKRSLRELLLGLKGELT

LTVEMERLDHAIFYDQIPETWMSLAYPSMLNLQSWFADLLHRIKELSLWLNDFKLPCTIW

LGGLFSPQSFLTAIMQESARKRDLPLDRMCLHCDVTKKDKDSITLPPIEGAFVHGLFLDG

ASWDCQLNSIVPLHPKELFCAMPIIYIKSVVQSKHDLLHIYECPLYKTRQLTLT

>GMOY011549-PA hypothetical protein|protein_coding|scf7180000652170:7375783-7380505:-1|gene:GMOY011549

MDLKTGATGGGGGLGVVGAATPPRPSATVRTSQAGADAKPKSKKDGIGFVFDIIVVRVDV

PNETFAKPDLLTVDVKFNNVDLKITSSRINVVEFRSGRSYEFIDTPLNLKEQLKKQPLAL

AVKYEDRVIGNGQIAWPDTFTSRINDCAGEITHCDETELKDDRGMKTGSIEVIIRIQTKC

KDYEDPDYNLSKRSDICSQMDKVIDPNDILFVVGEDKAACCCAQVGVIPASDSEKSVYPT

CLDLSNFRKVNGRTVTSPDILDSLNNGNCNQGQKVREQYKDLISGLDSIETSCCPRRKNT

SHQPTQKRSMPLCSSSGSSSRNPCENKCKARDQSCSGGKFCIACGRKSNFRNCRCPSGED

PFAAEKVVRLCPSLTAPELRQSVPRSRRTCPLCKEDVSWLPKMAACPKCGYKPIPYFDEK

AYNERICAEEILEEYLDKKSSCSDDIDQNSKDGSEEKKQCRCTCKPGKICAHCRIRKLCE

DIFQPEPKQGEQCNKCERQSSELYNICQSGAGVGDDCRPYLARVFSELRDLYDIKETKKK

KFEKNEKCEKQFSERAKRNIKSSRARSGRSDGDKSARSNFNVNATSTTATAEQADDNKIE

AQQSIEEVNRALRKRLRREARKKHQEVERIRKHFLQKNFKKSEKPCKSKNYNYRTVRKYP

GNQIGHKTCVNGFAAGRKNVPSNMGWLWNVEAKGSRAGWKPGAIRKPIKELMKYFLKDYP

ADTLRVSQYSYRKKGCNGKEKMDELVQKPTLHIFKKNGEYIITMRPLKDPEALKQCADPY

LDMKPIQFKIIKNPLMVELRKLKRCLKDMGFSKCTCHKPIICCFCRSFLDKKRLEYQCNK

ECRKRNLPNVADKLVLSDTTDSDVEFDFGVTPPAGVIKPDKGKKPDLVNHGTQYSEGDWN

VTPMYPREPNKYMKLYNCAIGERFGKAFGPYGPDGYYPGASPLPGGLYGPCGPGGGMGGR

GGGWGTGGEGGGVGGGGFGGRGGGKFGGKFGPGFGPGGGRGPFGGRGGPGGPGGPGGPGG

PDGGPDGKGGRHKGGGKGDGKVDMMKYILEKAKKDKAAGGKPKKKNLMEGPPPILCMIDR

RGKPCDPCAPCGPCGPCGPCLPCDPCGDTCYRHDPRIC

>GMOY011554-PA myosin light chain 2|protein_coding|scf7180000652170:7448153-7454961:-1|gene:GMOY011554

MLIKKDFPKLSKRLLERLTFGTAGLRVADEKKKVKKKKTKEEGGTSETASEAPSETATPA

PAATPAPASTTGSKRASGGSRGSKRSKQRAGSSVFSVFSQKQIAEFKEAFQLMDADKDGI

IGKNDLRAAFDSVGKIASDKELDAMLGEASGPINFTQLLTLFANRMATSGANDEDEVVIA

AFKCFDNDGLIDGDKFREMLMNFGDKFTMKEVDDAFDQMIIDDKNQIDTGALISMLTGKE

EEEEGEEAA

>GMOY011652-PA NAD-dependent malate dehydrogenase|protein_coding|scf7180000652170:11285211-11287331:1|gene:GMOY011652

MLKQITKQLAMQSLRNFSTTGQNNFKVAVCGASGGIGQPLSLLLKQNPLVSDLRLYDIVH

TPGVAADLSHIDTKSSTAGFMGPDQISGALDGAELVVIPAGVPRKPGMTRDDLFNVNAGI

IRDIVTVIATKCPKAMIAVITNPVNTCVPIAAEIMKKAGVYDPKRLFGVSTLDVVRARAF

IGEAVGADPQKVHIPVIGGHSGITIIPVLSQSQPAFKGDQTAIEKMTVRIQEAGTEVVKA

KAGAGSATLSMAYAGARFANSLLRGMNGEKNVVECSYVQSNVTEASFFATPLVLGKDGIQ

ENCGLPKLNDFEKKLLVTALPELKKNIQKGVDFANS

>GMOY011653-PA hypothetical protein|protein_coding|scf7180000652170:11297166-11299035:1|gene:GMOY011653

MQCHRIGINTATCNENILIYFEKNFRVAVCGACGGIGQPLSLLLKRNPLVSELRLYDIAH

VPGVVADLAHVDTKVRVCGFMGPDQICDALECVDVVVISAGSPRKPGMSRDDLFKTNACT

VMDITKVIAVKCPKALVAIITNPVNTCVPIAAEIMKKDGVYDPKRLFGVCTLDIVRARTF

IGEANGVDPKKVDIPVIGGHSGITIIPVLSQSNPAFKGDQAAIEKMTKHIQEAGTEVVKA

KAGAGSATLSMAHAALRFTNSLLKGLKGEKNVMECSYVESKATEASFFSTPLVLGKNGID

ANCGLPKLSEYENKLLEKAIPEIKKDVQKGIDFAKK

>GMOY011657-PA hypothetical protein|protein_coding|scf7180000652170:11332940-11338471:1|gene:GMOY011657

MSERENNVYKAKLAEQAERYDEMVEAMKKVASMDVELTVEERNLLSVAYKNVIGARRASW

RIITSIEQKEENKGAEEKLEMIKTYRGQVEKELREICSDILNVLEKHLIPCATTGESKVF

YYKMKGDYHRYLAEFATGSDRKDAAENSLIAYKAASDIAMNDLPPTHPIRLGLALNFSVF

YYEILNSPDRACRLAKAAFDDAIAELDTLSEESYKDSTLIMQLLRDNLTLWTSDMQADGD

GEQKEQIQDVEDQDVS

>GMOY011708-PA hypothetical protein|protein_coding|scf7180000652170:14105509-14106720:-1|gene:GMOY011708

MFGRVLYRSRNNILRYFCAKQTPPPAGGDKDSKTSCEGDKNKPPSESDAAKAAAAAASKA

AAASSPSKSAATAAPDKGPATPPGKAAATGASNKAPATAASGKAPATTAATQGAKTAKKG

EAQQPASGKGADKDKSGKGAPPPPPGQQTKKEPRIKTFQIYRWKPGEKPKMQTYNIDLND

CGMMVLDALIKIKSEKDQTLTFRRSCREGICGSCSMNIDGVNTLACLCKIDGNLAAPMRV

YPLPHMYVIRDLVPDMSQFYEQYRIIQPWLQRQNESKEPKGCAQYLQHPQDRLNLDGLVE

CILCACCQTACPSYWWNSDRYLGPAVLMQAYRWVIDSRDEATDKRLNILVDPYKLYRCHT

ILNCTNTCPKNLNPGKAIIKLKQLLAGYKKKTEPKLETAKLFK

>GMOY011773-PA hypothetical protein|protein_coding|scf7180000652170:18794781-18853236:-1|gene:GMOY011773

MARKRIYLTFPFKAINFKCSSSEFKCRSGNCIDGSKRCDRILDCPDGDDEDERCPDECSS

IEYQCRDGMNCISESKLCDGFNDCIDGDDEEHCDSIVPKLKYFCPKGKFTCRDLSCISIV

LRCDGHVDCPGDRSDEEGCPCLHDKWQCDDGTCISKRLRCNGNLDCPEDISDERNCDGGD

NDDYCKFNEFRCNNGQCIPYREVCNHIYDCSDYSDEIDCEIKEDHNVNRNYEDDELRRKY

DHYSYTTFNSLPPPRSSSSSSSSSSSSSPPSPSSSSLDGNVLGGIHELDVNDYSFYHPNI

YENANQRNPCPADKFRCANNICVPLRFRCDGLYHCNDLSDEEDCDNYVRGSQERPITDRP

PDNVIRTIPPTTMKPRWNNISNSYSPRKTTSTTIATPSTPRYRYMYPRTTTTQAPPKKTC

LSMEFMCENGACIPLESVCDGVRDCARYDDESYGLCNCSSDKFKCVQGGGCVPKTQVCDG

KPQCRDGSDEISCQDFDTDADTTFTECELYEFECDYSKCIPNDKKCDGFADCDDDTDESD

CQSYTEHCQPDEFECDQNYCIPINLQCNAHVDCKDGSDEQNCHYCHEGFACDTGECILNN

LHCNGKIDCTDGSDERNCGDCPELRFKCNNTCVDWNIRCNGKIDCYDGADEEGCQEPSES

DLNLSNNSCRSDQWQCDNLLCISKDYLCDGRTDCSDGSDESIKQCEKTVTTVLTPEDCRD

DQFFCDDDCHSTSLRCNGHYDCLDRKDEQNCPLGYPFPTRPPPIFPCPQHTCSNGKCFSE

NERCDGTPQCDDGSDEANCCAADQFRCRSGECVPAYAQCNGYRECPDNSDEDDCLDPART

CLSSQFRCDNGQCINILARCNGYTDCADSSDEKFCKISKESYIDYEGPLKGSLQCGGFNM

YQCDSGFCIPAYKRCNGVIDCPHDISDELDCAPLLNNEIDTRPTTASPHQLTLRTYPNNQ

VIKESREAVFRCRDEGMLRARVRWTRPGDRPLPAGSRDINGRLEIPNIRVEDSGTYICEA

VGYPRHVPGQQVSVQLTVEKSAWGNDRYHIPSNKILYGTVPHIGLEFFGLDNPRDERVPA

ACSSTQATCMNSECIDRSQICDGIPHCSDGSDEHSCSHGRKCHPNQFLCRNSKCVDRVWR

CDGENDCGDNSDEESCDPEPSGAPCRYDEFQCRSGHCIPKSFQCDDTNDCRDGSDEIGCM

APDKIRDPPPTQVLKQGDFLNITCVGVGIPVPVIVWRLNWGHVPEKCVSKSYAGTGNLYC

PDMQSGDSGAYSCEIINTKGSKFTTDTLVTVIPPDRPGVCPAGFFNMLARSPDECINCFC

FGVSKTCKSADLFTSVIQAPVSSHRVVDVELSPYSNIVINEAPASGMMSLLHGVQFRASD

VHYGGGRSAPYLALPSEYMGNQLKSYGGHLKYDINVVGSGRPTHHPDVIITGNGFTLTYR

ARLQPQVNVPNKMEVQFSPGTWKKPDGRLATREEIMMILANVDNLLIRLSYIEATEREVE

LTNILMNSAGAHDHGLGQASLVEQCQCPNGYAGDSCESCAPGYVRQKGGAWLGRCVPFAP

APCSPGTYGDPLRGIPCRECPCPQTGSNNFANGCSLGPDSEVTCNCHEGYTGRRCENCAP

GYQGNPILPGGHCYPVPESTCNAEGTYYPHPNGTCECKQLVVGPRCDTCAPDSFHLNAFT

YTGCIECFCSGLTNTCTSSSWYRDQIVSNFGQSRAPHGFALIRDYDADHPSNVDFVSTGS

SLSFSNAPTAEPLYWSLPAQFLGNKISAYGGKLNYTLSYSPMPGGLMSRSTSPDVVIKSG

EDLTMIHYRKTGVTPSSSSSYSVPIVESAWQRSDGQVVNRQHLLMALSKIDAIYIKATYT

TSTKEGTLTHVSFDIATPTNTGNAKAVEVEECRCPEGYIGLSCERCAPGYKRNPEAGLYL

GLCEVCECNGHSQECDAETGICKNCAHNTEGDNCDRCAYGYSGVATTGTPYDCTPGGDYP

PPPPPDNQTSCSYCNQAGTTSCDNGYCYCKPNVQGNRCDQCRPGTFGLSERNPDGCKECY

CSHKSTTCTSASLYRQLIPVDFFENPPLLTDEEGLIADTENLSHDFGANEYIYSYTSYTP

KYWSLRGSVLGNQLYSYGGALSYVLSVDSYGEYVPGNDIILIGNGMKLLWSRPVNEQESS

DYSIRLHEEENWHTIQRGVMQRASRTDFMNVLSNLEHLLIRATPKIPTTRTAIRDVILER

SVERPMPGSEHAIDVEVCSCPSGYVGSSCESCAAMFYRNNNRDCVPCPCQEEGSTSCNLD

DRGYVKCQCKLHYTGDRCQYPAEPVIKAPPDIVYDLRHGFCCNGYQFNIEANQSIADNEI

LQIYRGRENIGNITKLQYGCNVRDDYEPDRPTSRPSTPPDYDYRTQISVSIAPPEITIIP

VGGSITLTCTGRLVWNSSPVIVSWYKLNGQMPYNWEQDNGILHLYDLQIHDSGVYICQAR

NNDTQRIYEDKVSITITESARRTPAKIENLPPYATFDEYQPNQIDCEVSGNPMPTVIWTR

VDGQMSREAHTEDSRLIFESPRKSDEGSYRCQANNGVGYEEKYTTLRVRPTRPSPPTPPR

EVVYIEPPSFNGEPGSYVRLTCQPTTSVVLIYEWNKDGYPIYRTHNLIINANTLEIREST

QRDTGVYTCIGIDYRGRRNYTSDARVIIEEPRPPYDGDGSVGPVEPPLPGTVPPSVKRLP

EENLIIQGHDFSITCEASGTPYPSIKWTKVHESLGDNVQQTGSVLRIMNARPDNRGVYLC

IAENSAGHDQASTVVDIEPREHPTVDVEPKTPQTITAGGQAMLYCTANGIPEPRVQWRRV

DGQPLSSRHQIQNEPGYIIVNDITLTDAGDYECVAENEVGKVTSVTSIRVIVPPIVELEP

SLEVLSVTEGDEVKVLCTASGFPNPNVQWVELDAHPKLATPISEHYNQAYLEFYRVTPQQ

AKTYKCVATNEGGDDERYVILDVKPRRGDATDDSDVYYPPHQPQPQPQPPYQPHPQPPHR

QPTHYPPSYPTQPAYPPQSPYHTPTSLPENIYRTKSGEDVQLTCNLTSYGPPVQVRWTRL

DGSPLPANSYNDQNTLIITRVTDQNAGKYLCNAYDNHGSVITFHIAELVLVPIPHITLHP

RMPIYVTANENVDIYCEVEGEEPIHVSWHAENNRPLPSSVHIEGQHLRFISITPADAGLY

YCSASNRYGNTTEKAQVVVNRGHTYEPRPSSRFYELNEGDTVSMTCDAQPSHTPIRGEVM

YNWRREDNLPLPQTAQKRGHELLLYQVRKEDEGRYICESFTTGGDVSQPSYAELRISHVK

PLKPIKAKPLTTATSLTANNSSNRSYACQPSDFRCISHPHTCITKSMVCDGIHDCTDHSD

EFNCTRPLNNGNNNNGLKRWKKHSSYQSRPKPLATSQSPLATTQSTVSAPSRHNKRRKQR

LLKSSPAYKKYSSAKKAQSLMKKKIVGARPQHSYPPALHASSTALPRDLSLKLDQQHSKL

RVGESTEVECYSSDDTYSDVMWERADGTPLPPHIQQIGNRLVISHVTSVDAGTYVCKCKT

DEGDLYTTSYELEIEELMHEWKHPKIEYAKVGSMAKLKCDADNSQASPSYRWSRQYGQMQ

LGTDILSDKLVLRDVQANDAGTYICTVTDTNGESVDYPTILVVTGAVPQFDQNPLSYMSF

PTLRDSFIRFNFDITFRPELPNGLLLFNGQKRGNGDYISLSLKNRYPEFRFDFDGKPMIV

QAEHPITLNKWHTVRVNRFRRDGIMHVDDQHPVGFPTLSPSSSLDLIEDLYIGGVPSWDM

LPDDAVDHKVGFVGCISRLTLQGSIIELMKEAKVKEGINGCQPCTTNPCSNDGICMESQS

EMAYSCICQPGWTGRNCGVEGTQCTPGICGAGRCENTETGLECLCPLNRTGDRCQYIEHL

NENSLAFKRNSFAAYGVPRASKLNIKFRVRPNSLDDAVILYTAESEMPSGDYVAVVLRNK

HVELIINTGARLKPVIVRSTNPLPLNQWTEIEIARRFGEGILRVGSEPEQKAKATGAART

LYIRTPLYIGGYDNEKITLNYDVNVTQGFDGCISNLYEAQRQLNLIADIREAANIQNCGE

INEIDQNETFQTETTHDKHDHVDVAAPKMIIPDEGCDSDPCENGGTCHVVNDTVTCDCGI

GFVGPHCEDFILLKFDANFRGNGYLELDRSQFNDETNQKYSFAAMVFSTSDPDGLLLWWG

QPKGETYTGQDFMALALVDGIVEFAFRLNGEEAVIRNPDKRVDDGHRHIVLVKRTDNTAI

LELDHVLYADETRPTGKNTMSLPGHVFIGGAPDLDNFTGGRYKQNFTGCIHVVEGEESGT

IELGKVAISGINVDTCPAVDESLDDAEPPLQ

>GMOY011788-PA hypothetical protein|protein_coding|scf7180000652170:19942186-19953234:1|gene:GMOY011788

MSEIVHIQADEFFGVSSDKHCVGATGTCYNESDLQMEQVNFCCNEMESVKYVPCAILVDS

DPGIVASEHMFKPDNFVLGHSVACNNWRKCHGKEVGKLVDSVLDIIREEYKDCDYLQDLQ

LVYFLDANIFGSVDDLRMGTLLLAKISGESPSSKGFGNVAESRNATLRVYRHTGNTDDAF

CIDNEALYDVCFRALKVAIPPYRHLNHLVSATVSAVTKYLRFPGQLNMDLPKLAVNIITL

PRFHAFMPRFMLLNSRGMQKYGTVVEPEVTEKIFDAKNVMVDYYSRHCLFLTIAAIFRAR

KCKKGVDERVPSIQEQYRSFCIEWIPRNSETTIWDVPPDGLKMSVSIIGNSNTIQELFKG

MPEQFTVMFAHKLHSWYTEMREIVHIQAGQCGNQIGGKFWEVISDEHCIDATGTYYGDSD

LQLERINVYYNEATGAKYVPRAILVDLEPGTMDSVRSGAFGQIFRPDNFVFGQSGAGNNW

AKGHYTEGAELVDSVLDVIRKEAEGCDCLQGFQLTHSLGGGTGSGMGTLLLTKIREEYPD

RIMCTFSVVPSPKVSDTVVEPYNATLSVYQLMENTDETYCMDNEALYDICFRTLKLTTPT

YGDLNHLISATMSGVTTCLRFPGQLNADLRKLAVNMVPFPRLHFFMPGFAPLTSRGSQQY

RALTVPELTQQMFDAKNMMAACDPRHGRYLTVAAIFRGRMSMKEVDEQMLNIQQKNSSFF

VEWIPSNCKTAVCDIPPRGLKMSATFIGNTTAIQELFKRISEQFTAMFRRKAFLHWYTGE

GMDEMEFTEAESSMNDLVSEYQQYQEATAEEEGEFDEDEEEWPAIVKHLAVPACLVPGGI

ILNCTMSRKEMREIVHIQAGQCGNQIGGKFWEVISDEHCIDATGTYYGDSDLQLERINVY

YNEATGAKYVPRAILVDLEPGTMDSVRSGAFGQIFRPDNFVFGQSGAGNNWAKGHYTEGA

ELVDSVLDVIRKEAEGCDCLQGFQLTHSLGGGTGSGMGTLLLTKIREEYPDRIMCTFSVV

PSPKVSDTVVEPYNATLSVYQLMENTDETYCMDNEALYDICFRTLKLTTPTYGDLNHLVS

ATMSGVTTCLRFPGQLNADLRKLAVNMVPFPRLHFFMPGFAPLTSRGSQQYRALTVPELT

QQMFDAKNMMAACDPRHGRYLTVAAIFRGRMSMKEVDEQMLNIQQKNSSFFVEWIPSNCK

TAVCDIPPRGLKMSATFIGNTTAIQELFKRMSEQFTAMFRRKAFLHWYTGEGMDEMEFTE

AESSMNDLVSEYQQYQEATAEEEAGQCGNQIGGKFWEVISDEHCIDATGTYYGDSDLQLE

RINVYYNEATGAKYVPRAILVDLEPGTMDSVRSGAFGQIFRPDNFVFGQSGAGNNWAKGH

YTEGAELVDSVLDVIRKEAEGCDCLQGFQLTHSLGGGTGSGMGTLLLTKIREEYPDRIMC

TFSVVPSPKVSDTVVEPYNATLSVYQLMENTDETYCIDNEALYDICFRTLKLTTPTYGDL

NHLVSATMSGVTTCLRFPGQLNADLRKLAVNMVPFPRLHFFMPGFAPLTSRGSQQYRALT

VPELTQQMFDAKNMMAACDPRHGRYLTVAAIFRGRMSMKEVDEQMLNIQQKNSSFFVEWI

PNNCKTAVCDIPPRGLKMSATFIGNTTAIQELFKRISEQFTAMFRRKAFLHWYTGEGMDE

MEFTEAESSMNDLVSEYQQYQEATADEEGEFDEDEEGGADE

>GMOY011870-PA fructose-biphosphate aldolase|protein_coding|scf7180000652170:21996157-22003018:-1|gene:GMOY011870

MTTYFSYPNEALRSELTRIANAIVAPGKGILAADESVSTMGKRLKDIGVENTEENRRQYR

QLLFTSDPSLGESISGVILFHETLYQKTDDGTPFVELLKQRNIIPGIKVDKGVVPLMGSE

DECTTQGLDDLACRCLQYKKDGCDFAKWRCVLKIGQNTPSYQAIMENANVLARYASICQS

QGIVPIVEPEVLPDGDHDLERTQKVTETVLAAVYKALNDHHVFLEGTLLKPNMVMPGQSC

KKQYKPEDVGLATVEALRRTVPAAVPGITFLSGGQSEEEASVHLSAINNVSLLKPWALTF

SYGRALQASVLRAWGGKKECVEAGQKELLKRAKADSQAAVGKYVAGSVVGVGADAGLFVA

NHAY

>GMOY011879-PA hypothetical protein|protein_coding|scf7180000652170:22886587-22909851:1|gene:GMOY011879

MTEEETQQDEIESNRTIVEDFCKPFVLNEETSQQVSDLFLAEIKKGLCKYTQPKADIKCY

PTHVEKLPTRCEQGKFLALDVGGSNFRISLFIIQDVDNTKVESQDFQLPPKVLTGPGEHL

FEFFAECMSNFIQTHELQEEEFHLGFTFSFPLMKTSLKEAILISWSKDFQCKDVVGHDVV

AMLEAALSRRDNIHIKDIFVLNGTTATLISCAWKHKETKIGVTIDRDTNAVYEEKMKHIQ

LFSEERFNSTMLINTQWGSFGNHGALNFMRSPIDFALDESSTNPHEAIFEKMVAGMYIGE

IVRLTMIECINAGALLKGNLSEQIRKQMVFDTKHMSQIENEKGDAYQSTRQILETLGYKE

ATNEDCENIRYICNKVSTRSAELIAICLACLIDRIGDPYIVIGIDGEMYSSYPNYHERLR

KKTKQLKNSSILQTFSMAEADLNENLSETHHKIVKDICKQFVLSDDVYKQIKEMFLGEIK

RGLCKYTHESACVKCFMTFVEKLPSGCERGKSLALDVDETRCRVLYINLQGDRDFRMYSQ

NYPIPPQILVGPGRDLFDFFVECIADFVYDHNLQNDELSLGFNFGFPLNQKSIKKAILMT

WTRDISSAGVVGRDVVALLQDAINRRGGLRISNIVIANDTTGTLVSCAWKYREAKIGLVV

STGFNMCYLEKTKYLQLIRNNVNTSPTMIINCESGAFGNDGTLDFMRTPIDITLDKNSVH

VGEQLFEKMISGMYLGEIARLTMLECIKAGGMMQGDFSEEVRTPMIFDVEDMSQIEADGP

GNYAVTRKIFQKMGYSEPTNDDCENLRYICTVVSTRSANLIASCLACLIDRVGDPYIIIG

VKGSIYETYPNFSTRLERKLKRLVRPEYEFDLVPAEGDSGQGAALLAAFVILKMPKPDYT

QYIIEANRRIVKDICKPFILSDDTYKRIKDLFLVDIKKGLGKYTHKKASVKCYMTFVEKL

PSGCEHGKFLALEVSGDDCRVLCVNLQGDDDFRIEWQNFDVPPEIKVDTATHFFDFLAKC

LSDFIVKYDLQYDELSLGFTFAFPLKQTSIRRGILVKWTKGYNVSGVVGNDVIALLQEAI

ERRGDLPINNIVILNNTTGTLISCAWKHREAKIGVLISGGFNMCYLEKSKHIQLFKNIGN

YCPTMVINCEAGAFGNDGVLDFMRTTVDFTVNKTSMRPGQQVYEKMVAGLYLGEIARLTL

LDCINAGAMLKGIVSKEILTPMIFRIHDMSQIEHQEPGHYAITRKIFEKMGYRDTTNGDC

ENLRYICTAISTRSANMVAACLACLVERVGDPYLIIGADGSVYRSYPNYPSLLRNRLKKL

VRPDFKFDVVQAEDENIKKILIQNFYFAKLKMTEEDTKDKDSQINRSVVEEICKPFIVSD

DVYRKIRDVFQNEIKKGLCKYTHESASVKCFLTFVEKLPSGCERGKFLALDIGGINFRIL

LVNINSGENLKIEAANYELPESLMTGTGRDLFDFLAECLSAFIYKHELQKEELSLGFTFA

FPLKQTDLSKGVLITWTKAFSCSGVVNHDVVDLFKQAINRRDDIRINNIVILNDTTGTLI

SCAWNYREAKIGLIIGSATNMCYLEKTKHIELFKGGVNASPTMIINCESGNFGSDGSLDF

VRTPIDIALDQNSVNAGEQIYEKMISGMYLGEIVRLILLECVNAGAMLNGVQSEELRTSM

SLDVKHMSEIEAAEPGNDSASRKIFEIMGYKRPSDEDCEHLRYICNVISTRSAYMGAATL

ATLVNRVGDPFVVIGVDGAVYRMYPNYPERLRKKLRDLARPEYQFCLKVAEDGSGRGAAL

LAATLLKMSEENSSSEPLSNHAIVEEICEPFILNDDKYQQIRDIFLDEIGRGLCKYTFKN

AYVKFLMTFVEKLPSGCERGNFFGLHLDGTTLRILLINLHSEEDFQIEGANYELPESLLT

GPGRDLFDFLAECLSKFIYKYELQEDELSLGFTFSFPIKQNKLTRGVLLPRTKGFCCSDV

VGHDIVGLLQQAIKRRGDIHIPNIVILNETTGILISCAWQYREAKVGLIVDTAISISYLE

KTKHIDQFKRGVNASPTMIINCKSDNFGNDGLLDFVRTPIDFALDNNSVNAGQQIYEKMT

SGKYMGEIVRLIMLKCVDAGAMLRGNHSDQIRTPMSFNINFMSKIEAEDAGKDSIIRQIF

EIMGYRRPTVEDCQHLRYICNLVSSRSAYLIAATLAALINHIGDPFVIIGVDGSVYRHSR

EETRLNEILTPFDITKDEMMKIKDLILKELKLGLKRDTHNTADTKCYPTYIQSYPSGCEH

GMFLVTTIHAVKVHVLFFHLKGENDYRLDEESSDIPEGINQAVELFDFIVEKLHKLVKSL

NLEREPLPLTMVLPYPLLQINLASAILLKFTTKLKIAGMENKDVGQMMRESMRRHPNIRF

ELTAVINDVTSAFMSAAWRHKNVRISFIVGAATNAGYWEKVSNIESVIQTRKPEMLVNTD

IAEFGSSGQLEFLATEFDEALEKLSPTKGQNIFEKMASAWYMSELSRRVIIKCINENIIF

GGQSNVQLNRQDALKFANVQGTLVEADQYLYMSLMLDKLGINLPSETDCARIHHIMEKVV

TRSASLVAAAIVAMIEIIDEPDIKIGLDGEVCNSLPIYHNMIRSKIDSILKPEHTYELVE

ANDEHGRGGAITASLILQEDYIYENLNTA

>GMOY011898-PA hypothetical protein|protein_coding|scf7180000652170:23203839-23204460:1|gene:GMOY011898

MNSFSVFLVFALAAMVVAEPPSGYSYPRGGGGGGFGGGFRGGFGGGGGGGGYQAVSGGFQ

TSEGLNVDPQLLEQVRQILLNEESKSGGGGGGGGGGYPSPSSSYGAPSPQYGVPSYNGGR

VVGIDLEGVRQAIQVAQFAQQSTQAGGYPGGGKPSGSYGAPY

>GMOY011904-PA hypothetical protein|protein_coding|scf7180000652170:23339642-23345911:-1|gene:GMOY011904

MFGTLRSNSLRNTSKIFRQVRRYASQFINQVLQLQHTEICADPPSRALVLGVYADEDDKN

DVGILTPAAWRYNITRTNGRLIEVLRMSGPLPKRGEARILFAQEVEKVPYYSAVAVVGLG

KECLGYNPYEIIDEQKEGIRRSVAKACMELALLNTNRIEVENCGHAESAAEGAALGVWAY

QELRNRENRIAVPTVDLYTTKEEICDLEGFRIGLQKAAAQNLTRQLQEMPSNILTPTSFA

QNVVEVLCKSGVNVEVKVEGWAESQVMNSFLSVGKASCEPPIFLELSYYGTAADERPIVM

IGQGITYDCGGLCLKSRDQLYVKRGDMTGAAVVVATCRAIAALRLPVNIRGLIPLCENVM

GCNSFRPGDTVKCMNGKHIKIQGTDQAGVLILADALLYAQNFCPKCIVDVGTNSRYMREV

LGEAACGVFTNSEILWQQIKHASMHTGDRVWRMPLWEYYAQQIRGGMSSDVQNYGIGRGG

RPCKAAAFLREFVPCGQWMHMDATNVMTTRGTTFEYLRAGMAGRPTQCYGQMRCDAPANM

LMKFMLQLQHTEICADPPSRGLVLGVYADEDDKKDTGILTPAGWKYNIQRTGGRMIEILR

MSGPMPKRGEARLLFAQETEKIPYYSAVAIVGLGKECLGYNPYEIIDEQKETIRRSVAKA

CMDLARLNTDRIEVENCGHAESAAEGAALGVWAYQELRRKKDRIAVPTIDLYTTKDEVCD

IEGFRIGLQKASAQNLTRQLQEMPANILTPTAFAQNVVEVLCKSGVNVEVKVEGWAESQV

MNAFLSVGKASCEPPIFLELSYYGTSANERPIVLIGQGITYDCGGLCLKPKEKLYVMRGD

MTGAAVVVAACRAIAALRLPVNIRGLIPLCENVMGCNSFRSGDTVKCMNGKHIKVQGTDH

EDVLVLADTLLYAQNFCPKCIVDIGTTSWSMHHTLGEAACGIFTNSEILWQQIKHASMHT

GDRVWRLPLWNYYSTQVTSGMSADVQNYGIGRGGKPCKAAAFLREFVPCGQWMHIEASNV

MTTKGTCFEYLRAGMAGRPTRTLIEFIAQTICRDTAPKFPPKEK

>GMOY011932-PA hypothetical protein|protein_coding|scf7180000652170:24097376-24104331:1|gene:GMOY011932

MNLVTIFLLLLTVRLFSVFVVKTFYVPDEYWQSLEVAHKIAFGYGYLTWEWEQGIRNYIY

PLAIAGIFKILALLNLDTVYVLVLMPRVFQAMLSTYSDYRFFIWSGKKKWALFSIVVSWF

WFYMASRTLSNTLEISLTSIALSYYPWQGEGIVYFGPAALCCFLRPTSVILWLPLVLYHI

KKSKLSLTQLIFKRFIIIGAVTCVICISIDSLMHGQLLVTPYEFLKYNIYHNVGSFYGSH

AWHWYFTIGLPTVLGISFVPFLFGVIETIRHRKTYPTRKVLLITILIVMLVLSAVEHKEF

RFVGLLLPLCLYITSDTLTKWSYNASKVSLWCTALLLIIGNAVPALYLSTVHQRGPLEVM

NKLQDIAYRYRDEYKNPASILFLMPCHSTPYYSHIHQNVTMRFLTCEPNLKQKVNYKDEA

DQFYDSPMHWLRSHVPSYPPTAKPTHVVLYEPLVTQIKDFLVDYKRLYVVANAEYAQART

GKNILVYQRLRGGEQNLYNREEYKEEQQFKQLSDLKEHKNEDNLIDGEDEFGEKKKLAYE

TSKSEQATRIQKGILTMAASSRALLKVRDGLVTAVRYAAASTQYNKTRPNLLLTENSRVI

CQGFTGKQGTFHSQQALEYGTKLVGGISPKKGGTQHLGLPVFKNVAEAKKATDPHATVIY

VPPAGAAAAILEALESEIPLIVCITEGVPQHDMVRVKHALLTQQKSRLVGPNCPGIIAPE

RCKIGIMPGHIHKRGKIGVVSRSGTLTYEAVNQTTEVGLGQTLCVGIGGDPFNGTDFIDC

LEIFLRDPDTKGIILIGEIGGVAEEKAAEYLSYFNTGMKAKPVVSFIAGLSAPPGRRMGH

AGAIISGGKGGAGDKIKALEKANVIVTRSPAQMGKELHKEMKRLELV

>GMOY011978-PA hypothetical protein|protein_coding|scf7180000652170:25308788-25310753:1|gene:GMOY011978

MAYSTQKDFFHVPFMDEHVDFVCRPDCAKLKRYNSIIADSAWALDDKKQSKYLYDLVLCE

TPGLLNVCSSADMGMSGVGSAGAGDEGLLAQLGSISGGDSLLQCANTHLAEYQKLLNVKT

SLEKELNVSNERLKSATEENKKIKEILATKLDANTSKDFYNKVRQLVSSGKLNKNEENEM

LQIHKRIENMLKAYEILRAENYYVKRLVEKLANRCSLEKIKTEPEQSKDVGYLQKEVDKL

RKECIMLRDMEDGYQKLKQEMKDQHCAQKRLSDRDADNIKAIISDRNNLRDKCKSFNQKV

GDMQKKERNLNKDLENKAKCCANLEAEMQKMQKYYEDQMQQACFREECLKAQLEDLKEDF

MQVKCQAQKSDMLQMEVSCLRNEILKRDMALSDYDCQYKQLMYKAKRFKSAGYRILPPAE

PREDTMRAELFPESLTED

>GMOY011979-PA vacuolar H+-ATPase v1 sector subunit E|protein_coding|scf7180000652170:25313975-25317206:1|gene:GMOY011979

MALSDADVQKQIKHMMAFIEQEANEKAEEIDAKAEEEFNIEKGRLVQQQRLKIMEYYEKK

EKQVELQKKIQSSNMLNQARLEVLKVREDHVASVLEEARKRLGEITKNKAEYKQVLEKLI

LQALFQTMEQSVILRCRQADVDLIKEILPSVINYYKDIIGDDIEIAVDRDNHLSSNLCGG

IEIIALNGRVKVPNTLESRLDLIAQQLVPEIRNALFGRNVNRKFTD

>GMOY011989-PA acyl-CoA dehydrogenase|protein_coding|scf7180000649844:126212-131214:-1|gene:GMOY011989

MAFFNRLAVRSVRQLTSSNCRYASTSHVTPGGPSFGLTEEQKQMQEMARKFSREEVLPVA

AHHDKTGEYPWDIVKKAWALGLMNNHIPQEFGGLGLDVYTTCLIAEELAYACTGIMTALE

ASSLGQTPIILSANKEQKKKYLGRLLEEPLVAAYCVTEPVAGSDVAGIRTRAEKKGDEYI

INGQKMWITNGGVANWYFVLARTHPDPKAPASKAFTGFIVERETPGLTPGRKEINMGQRA

SDTRGITFEDVRVPKENVLIGEGAGFKIAMGTFDKTRPPVAAGAVGLAQRCMDEALKYSL

DRKTFGVPIAYHQAVQFMLADMAVGVETSRLAYRLAAWEIDQGRRNSYYASIAKCHAADM

ANKVASDAVQIFGGNGFNSDYPVEKLMRDAKIYQIYEGTSQIQRLIISRNMFEAAKASSS

>GMOY012011-PA arylphorin|protein_coding|scf7180000649808:31083-33780:1|gene:GMOY012011

MKIAYIFLAIFAAVSAFDSKHQKVTLNAYQHRFPIPSVFQQQRTLGQQQTAYNQKTSINQ

QAVHGYQNTAHNAQNAGYNQQYSAYNPLNKQNIVYGSQKNQNVKYASKEFLAKQKFLFEI

VYRVEDPLMFEEWIQLAQTATFNPSEYIQYDYYMQKFQQAYKAGALLPQGEFFGALVKTH

NKQLIGLFNFFYFAKNFETFQNNVAWARVHVNEHMFIYALNLAVIHRQDLQGMILPSIYE

IFPQYFFNSKFVYQAEKFDYDVWSKLVMYEKQYKDVLYAPYFTQQQQQQQFNQQQQQQQQ

VNQVNKNFYFYTKDFKTWQWWKLMGLGENWYSEDRFMLRDNIQQYNQDPKYVEAMQDVQM

FWMPVDYTRDIDIFNQESVLSYFTEDLDWNAYWYYYNLDYAFFLDGKTFGLNKDRRGENY

LYTVRQILARYYQERLSHGLGDIPQVSVNDEYEAGYDPQLIYHNGVGFSYRKNYYDIESY

NNDGLLKKINNFFTRLDDVISTGYYKTQDGTFIDLRKPQAIEFIGNIMQGNVDVYDQYFF

RQWNMFTHMYLADVEPQDTEVFPNIFVNYETMMRDPLFYSVYKRIADVYFQFQYYIKPYT

QQELLFPGVTIKNVQVTDLVTYFDLVDFDVTNLLNDKMTFVDGQFVWDKTLLARQARLNH

KPFALEFTIESDKPQPVVIRTFLGPKYDEFGRTISIMDNQQNFIELDQFIHTLTAGVNTV

QRNSQDFYLTIDDRTTYTELYKQVMLALEDKQQFPMDISQPHCGFPDRLMLPRGWAKGMP

MQLFVFVSPFTASYQPYSTYDTTYSCGIGSGVRYVDQKPFGYPFDRIVDELEFFVPNMYL

KDVKIYHADVLRKYADQPYLQFGQFDYNYYNYNY

>GMOY012074-PA hypothetical protein|protein_coding|scf7180000640693:15984-38012:-1|gene:GMOY012074

MENFTPKEVKILETVEDIQERREQVLSRYNEFKIETRQKREKLEDSRRFQYFKRDADELE

SWINEKLQAASEESYRDPTNLQAKIQKHQAFEAEVSAHSNAIVSLDNTGQEMINQNHFAS

ETIRRRLDELHRLWELLLSRLAEKGQKLQQALVLVQFLRQCEEVMFWIKDKEAFVTADEF

GQDLEHVEVLQRKFDEFQKDMASQEYRVTEVNQLADKLIQDAHPDRDTITKRKDDLNEAW

QRLKQLAIVRQEKLFGAHEIQRFNRDADETVAWIAEKDVVLSSDDYGRDLASVQALQRKH

EGVERDLAALEDKVSTLGAEAQRLCSIHADHSDQIRDKQAEIANYWQSLKAKAGERKQKL

DESYFLHRFLADFRDLVSWINGMKAIISADELAKDVAGAEALLERHQEHKGEIDAREDSF

KLTIESGQKLLEREHYAAAEIQEKLAALENDKSSLLSLWEDRRILYEQCMDLQLFYRDTE

QADTWMAKQEAFLANEDLGDSLDSVEALIKKHEDFEKSLAAQEEKIKALDIFATKLIDGQ

HYAADDVAQRRQMLLARRAALLEKSNKRRQLLEDSNRYQQFERDCDETKGWISEKLKFAT

DDSYLDPTNLNGKMQKHQNFEHELNANKSRIEDITTVGTELIEKGHYAADQVNTRMQEIV

VLWETLVQASDKKGCKLHEACQQQQFNRTIEDIELWLSEIEGQLMSEDHGKDLTSVQNLQ

KKHALLEADVMAHQDRIESIKVAANKFIESGHFDADNIRQKESNLSSRYAALAAPMAERK

QHLMDSLQVQQLFRDLEDEAAWIREKEPIAASTNRGRDLIGVQNLIKKHQAVMAEINNHE

ARLLNVISSGENMLKDQPFASDDIRQRLDALQEQWNNLKDKSNQRKQDLEDSLQAHQYFA

DANEAESWMREKEPIATSNDYGKDEDSSEALLKKHEALVSDLEAFGNTIQALQEQAKNCR

QQETPVVDITGKECVVALYDYTEKSPREVSMKKGDVLTLLNSNNKDWWKVEVNDRQGFVP

AAYIKKIEAGLSASQQNLVDNHSISKRQAQINSQYDNLLTLARERQNKLNETVKAYVLVR

EAADLTNWIKDKENHAQIADVVGEDLEEVEVLQKKFDDFTDDLKANEVRLANMNEIAIQL

TSLGQTEAAMKIQTQMQDLNEKWNNLQQLTAEKASQLGSAHEVQRFHRDIDETKDWIAEK

ENAINNDDLGKDLRSVQTLQRKHEGVERDLAALRDKIRQLDETANRLMQSHPDTAEQTYS

KQKEINEMWNQIITKATARKEKLLDSYDLQRFLSDYRDLLAWISSMMSLVTSDELASDVT

GAEALIERHQGHRSEIEYMFGNSTAKSFFHPTSKEEHRTEIDARAGTFAAFEQFGNELLQ

ANHYASPEIKNKIEDLGKTREELEKAWTERRLQLEQNLDLQLYMRDCELAEAWMSAREAF

LNADDVDDKGDNVEALIKKHEDFDKAINGHEEKIAALQVLADTLINQNHYAADLIDGKRK

QVLERWRHLKEGLIEKRSRLGDEQTLQQFSRDADEIENWIAEKLQLATEESYKDPANIQS

KHQKHQAFEAELAANADRIQSVLAMGENLIHKKQCSGSEDAVQKRLTQIADQWEYLTHKT

TEKSLKLKEANKQRTYIAAVKDLDFWLGEVESLLTTEDSGKDLASVQNLMKKHQLVEADI

ISHQDRIKDMNKQADSLVESGQFDSAGIQEKRQTINERYERICNLAAHRQARLNEALTLH

QFFRDIADEESWIKEKNCWWDPMTMAHKRLEAELGSHEPAIQAVQEAGEKLMDVSNLGVP

EIEQRLKALNQAWAELKNLAATRGQKLDESLTYQQFLAQVEEEEAWITEKQQLLSVDDYG

DSMAAVQGLLKKHDAFETDFAAHKDRCALICEQGNTLVEAKNHHGDSIGQRCQQLRNKLE

NLNALAARRKGSLLDNSAYLQFMWKADVVESWIADKENYVRSDEYGRDLSTVQTLLTKQE

TFDAGLNAFEQEGIHNITTLKDQLINASHAQSRAILKRHEDVISRWQKLRDASETRKQRL

LAMQEQFRQIEELYLTFAKKASAFNSWFENAEEDLTDPVRCNSIEEIRALRDAHAQFQAS

LSSAEADFKALAALDQKIKSFNVGPNPYTWFTMEALEETWRNLQKIIEERDGELAKEAKR

QEENDKLRKEFAKHANLFHQWLTETRTSMMEGSGSLEQQLEALRVKATEVRARRVDLKKI

EELGALLEEHLILDNRYTEHSTVGLAQQWDQLDQLSMRMQHNLEQQIQARNHSGVSEDSL

KEFSMMFKHFDKDKSGKLNHQEFKSCLRALGYDLPMVEEGQPDPEFEAILDVVDPNRDGY

VSLQEYIAFMISKETENVQSYEEIENAFRAITASDPQRQLIMNTEEEPEVVGTISNMNMT

PVIVAAIIGLIFIALFIILRKRSSVRRDFLLTGLSESGKSAIFMRLLHKKFPDTYVSVKE

NMGEYKSGGLAGRIIDIPGHYRVRDKCFDQYKRTAKGIIFVIDSVTIQKDIRDVADALYT

ILSDIATVPCSFLVLCSKQDVATAKSVKVLKQSLEKEMNLVRGTRGKLEVLGEKEANKPS

YLGKNGKDFEFSHLQQNIQFYECSAKEGQLSHLTDWLDRMLSPGGVWYCRT

>GMOY012164-PA Chemosensory protein|protein_coding|scf7180000652157:2283403-2283783:-1|gene:GMOY012164

MKYLTIVAVIATLSAVVVMGAEEKYTTKYDDVDVDEVLKSDRLFKNYYNCLIDQGKCTPD

ARELKKSLPDALQTECSKCSEKQKKTSEKVIKHLMDHKPEEWKVLQTKYDPEGIYYSKYK

ARDAKA

>GMOY012179-PA acetyl-CoA acyltransferase|protein_coding|scf7180000648022:91033-92265:1|gene:GMOY012179

MANFLRISSLFSTQSRSFSSKFNDVLIVSAARTPMGSFQSQLAPLSATQLGAVAIEAAVQ

RAGLSKEDIQEVYMGNVVSAGLGQAPARQAAIFAGLPKNVCCTTVNKVCSSGMKSVMLAA

QTLMLGQSEIIVAGGMESMSNVPYYLRRGQTPYGGINLIDGIVFDGLWDVYNKFHMGNCA

ENTAKKMGISRQDQDDYAVNSYKRSAKAWSDKVYDAEIVPVKIQQKRKPEIVVTEDEEYK

RVNFDKFGNLATVFQKENGTVTAGNASTLNDGASAVVLMTADAAAQKNVKPLARIVAFQD

AETDPIDFPIAPAFAVPKLLEKGGVKKEDIAMWEINEAFSLVVVANVRKLDVDLAKVNIH

GGAVSLGHPIGMSGARLVTHLSHALKPGEYGCASICNGGGGASSILLQKL

>GMOY012189-PA hypothetical protein|protein_coding|scf7180000648170:22175-22869:-1|gene:GMOY012189

MFPNGRSVRLFAVFAFFGLFVCIQANDVENAFEEHKVVPDVIDIAPQQFLDVIYDKGIKA

EKGVQLTPTQVKNEPTVMWAADEDAYYSLIMTDPDAPSRAEPKFREFRHWLVANIPGNQV

EKGEVIAGYVGSGPPKGTGLHRYVFLLYKQSGKITFNEKHVANNSREERPNFRAAKFAEK

YNLGSPIAGNFFQAEWDEYVPTVHKQLSGGN

>GMOY012285-PA hypothetical protein|protein_coding|scf7180000650885:84287-84511:1|gene:GMOY012285

MRVLSHLWRRVLMNNFNNLWSVVITLMVYITICRIVMNNFIDFWLSFNQSLMYHFYYWRR

FANVIKIFNVVARN

>GMOY012308-PA predicted (partial) protein lethal essential for life

MKKLPPPGSERVVPIAQTGPSSKEDNEKKVETTTA

>GMOY012309-PA lethal 2 essential for life|protein_coding|scf7180000652156:531402-531810:1|gene:GMOY012309

MSVVPLMFRDWWDELDFPMRTSRLLDQHFGTGLRRDDLMSSIWSSRPTLLRSGYLRPWQR

TGTGLQKLDSGSTLNVNDEKFEVILDVQQFSPNEITVKVTDRSVVVEAKHEEKQDEHGYV

SRQFTRRYMLPSKIFL

>GMOY012318-PA hypothetical protein|protein_coding|scf7180000648514:24395-26599:-1|gene:GMOY012318

MLWNIRWNNGVFKADYRQQLLATLSVTIISLSHGVALGWFSPMLMKMQSPETPLNFSLDV

HESSWLGAMISLGALTGNTLFSIILSRLGRKVAIYSLAFPHALLWLLLYYARSINYLYAA

RFFTGFTGGGSYVVVPIFVGEICNSSIRGRLTSLFGLTINLGTLFGYILSSHVRYHNIPW

IVLPLPCLFLFLVTRYPETPQFLLRAGKEKRAEQAFIFYQGRRCMLTLKENLRNQFEQLK

STYGNQTSAGKKVTYQDFLEGKSLKVLGIGLVLGIIHSYSGLFAFMSYMSNIFAATKTDL

HPDTNTIITGVVQVIGSYLAIGIVDRYGRRILMITSITGVGLGTAALGLYAFLVEKNDID

LSSFSYWLPVFLMSFIIFMANIGLNAIVYVIMVEILPSKIRSIGTMFFMVAWSISTFISL

KLFPMSMHFFGLSSTMWFCSAVSIFGVLFVAIFLKETKGISFDGVDNVILS

>GMOY012319-PA Major Facilitator Superfamily transporter|protein_coding|scf7180000648514:20131-21975:-1|gene:GMOY012319

MTSMKFRQLIDMSIGSPEPGHVNFYALHCLLSCIAEKLNIIDDTVDFSKYDTLVLYATSK

PNHQLAKLLTGSVRLAGGADEENPPGGDEHPTEPEEEEKIELEEKEEKELELEKPLAPEP

EQSGVIEVAVGEEEQEREPSEKSISSKVSLHELDFRVTKLEIIAQRQSVLEEYFTGVSVM

KDQIEFAITHLLHLTLLTLSKAPDAKRIRELYEMGKSLLQLRDVNPHIEGAKLFDVTAFP

SLHFGESGLQLTERMSEEGIFELQHEKKEGEDDHKMIEDHLCYSGEKLLEQLLELKSDFC

LLVNKVNEVSARVLKQESQQTVARTQELQEQMKEVKLFTTNLKTNQDRMEMRISHNINNI

ETIKSTLEDVLAEKVDKSELEILLADKVDYNQLQRKVSLDQMLELQCRIDKKFCEMLRQI

NDNDRKNNMMVEHLKETLGFAAIEGILSTFKGQIEKEIHHLQHMLQTYIDSTNDECAAAG

ARIKVLQDLACLSCDTTCVMRSMEKAKVAKLPNAHASVLLSPLITYELGSIRKSGIMGFY

RKDDFPHAPHAWMNRQNAGLANLKKCVPRHAGGSHTTNTARDRVEKITMNNKK
